# Dated Phylogeny of *Banisteriopsis* (Malpighiaceae) Suggests an Ancient Colonization of the Cerrado and No Evidence of Human Manipulation in the Origin of *B. caapi*

**DOI:** 10.3390/plants14071149

**Published:** 2025-04-07

**Authors:** Thais A. C. Santos, Bruno S. Amorim, Jefferson R. Maciel, Cassiano A. D. Welker, Scheila Cristina Biazatti, Regina C. Oliveira

**Affiliations:** 1 Departamento de Botânica, Universidade de Brasília, Brasília 70910-900, DF, Brazil; thaiscoelhobiologia@gmail.com (T.A.C.S.); reginacelia@unb.br (R.C.O.); 2 Faculdade de Tecnologia de Manaus, Universidade do Estado do Amazonas, Manaus 69850-000, AM, Brazil; brunosarim@yahoo.com.br; 3 Jardim Botânico do Recife, Prefeitura da Cidade do Recife, Recife 50030-230, PE, Brazil; jeff.r.maciel@gmail.com; 4 Programa de Pós-Graduação em Biodiversidade, Universidade Federal Rural de Pernambuco, Recife 52171-900, PE, Brazil; 5 Instituto de Biologia, Universidade Federal de Uberlândia, Uberlândia 38400-902, MG, Brazil; cassiano_welker@yahoo.com.br; 6 Departamento de Engenharia Florestal, Universidade Federal de Rondônia, Rolim de Moura 76940-000, RO, Brazil

**Keywords:** Ayahuasca, biogeography, Malpighiales, Neotropics, phylogenetics

## Abstract

*Banisteriopsis* is a genus in the Malpighiaceae family with 61 species, notable for including ritualistic taxa such as *B. caapi* (Spruce ex Griseb.) C.V. Morton, one of the main components of Ayahuasca tea. We analyzed 38 *Banisteriopsis* species, representing more than 60% of the genus, to investigate its geographical origin, diversification period, and colonization routes in the Neotropics. Plastid genes (*mat*K, *ndh*F, and *rbc*L) and nuclear regions (ETS, ITS, and *PHYC*) were used in our analyses. Divergence time analyses were performed using Bayesian inference with a relaxed molecular clock and ancestral area reconstruction. Our results show that *Banisteriopsis* originated in the Miocene approximately 22 million years ago, and its diversification coincides with the expansion of dry areas in South America. *Banisteriopsis* began colonizing the Cerrado earlier than most other plants, and the history of the genus reveals that the biome served as a source of species for Neotropical rainforests. Our results also indicate a probable ancient origin for *B. caapi*, with no evidence of human manipulation in its diversification, and they reinforce archaeological evidence of a millennia-old exchange of uses among Amazonian peoples.

## 1. Introduction

Ayahuasca tea has been used in spiritual contexts by ancient peoples and is currently utilized for religious, therapeutic, artistic, and recreational purposes in various cultures [1,2,3]. The ritualistic use of Ayahuasca has proliferated over the last century, originating mainly in Brazil and Peru, and it is now consumed on almost all continents [4]. However, the time of use and geographic origin of Ayahuasca remain intriguing subjects, still surrounded by uncertainties. The studies of [5,6] suggest that Ayahuasca has been consumed for millennia in South America, but without methodological support. Other studies [7,8] propose that Ayahuasca use dates back to between 500 BC and 500 AD, based on pottery supposedly used in rituals. Ref. [1] states that most religious groups in Brazil believe that the tea has been used since the Inca Empire. The arguments of [9,10,11] bring new elements, inconsistent within the ancestral origin of Ayahuasca. These authors show that there is a tendency to consider the use of Ayahuasca as a relatively recent cultural practice, attributed to Mestizos (mixed race) and linked to the origin and diffusion of Ayahuasca along the rubber route in the western Amazon.

*Banisteriopsis* C.B. Rob. includes the most widely used ingredient of Ayahuasca tea: *B. caapi* (Spruce ex Griseb.) C.V. Morton. This genus is also known for having other medicinal species [2,3]. *Banisteriopsis* comprises 61 species found in South America, seven of which extend into Central America, including Mexico, and one species is exclusive to Cuba. Most *Banisteriopsis* species occur in the Cerrado—which is the center of diversity of the genus, with 32 species—followed by the Atlantic Forest, with 14 species, and the Amazon, with 13 species [12,13]. According to [14], the fact that *Banisteriopsis* is distributed exclusively in the New World, with almost half of its species occurring in the Cerrado, suggests that this region is likely related to the diversification of the genus during the expansion of the savannas in the Pleistocene. The phylogeny, which includes more *Banisteriopsis* species to date, has not evaluated the influence of savanna expansion on the evolutionary history of the genus, nor its divergence times or biogeography [15]. Thus, the hypothesis that savanna expansion influenced the diversification of *Banisteriopsis* during that period still needs to be addressed, considering the current knowledge of the evolutionary relationships of the genus.

Some authors postulated that biome transitions were rare events among Neotropical plants [16]. However, Neotropical biodiversity includes a very significant number of biotic exchanges, with the Amazon serving as the main source of species for other biomes in the region over the last 60 million years [17]. Dispersal events from the Amazon to South American savannas represent the second most frequent type of biotic exchange among Neotropical plants after the dispersion from Amazon to Mesoamerica [17]. Based on this evidence, a possible explanation for the diversification and expansion of *Banisteriopsis* is that the genus may have had an ancestral area in Amazon forest, later colonizing the Cerrado and beginning its diversification in parallel with the expansion of dry areas in South America. However, this hypothesis needs to be tested experimentally. The ancestral range of *Banisteriopsis* and the role of biome transitions in the evolutionary history of the genus remain unknown.

Although the center of diversity for *Banisteriopsis is* proposed to be in the Cerrado of central Brazil, *B. caapi* occurs in the northwestern Amazon (Brazil, Bolivia, Colombia, Ecuador, and Peru), extending from the Orinoco basin in Venezuela to the Pacific coastal areas of Colombia and Panama [18,19]. Ref. [14] commented on the difficulty of determining where *B. caapi* is native, as most herbarium records come from plants cultivated by indigenous peoples. Ref. [14] also proposed that *B. caapi* has morphological affinities with species that occur in the Amazon and Atlantic Forest, including *B. membranifolia* (A. Juss.) B. Gates and *B. schwannioides* (Griseb.) B. Gates. Due to its long period of cultivation across this extensive geographic area, it is challenging to accurately determine the region of origin of *B. caapi* [14,20], although evidence suggests the probable Amazonian origin of the species.

Several open questions about the geographical origin of the genus *Banisteriopsis* and *B. caapi* (Cerrado and Amazonia, respectively) and the period of divergence of *B. caapi* compared to the ancestral use of Ayahuasca still remain. Here, we infer the most comprehensive *Banisteriopsis* phylogeny to date to test hypotheses regarding the genus diversification period and its colonization routes in the Neotropics. Therefore, we explore the following questions: (a) was the Amazon biome the ancestral area of *Banisteriopsis* and *B. caapi*?; (b) was the main dispersal route of *Banisteriopsis* species from the Amazon to the South American savannas?; (c) was the diversification of the genus influenced by the expansion of savannas during the Pleistocene?; and (d) was the divergence of *B. caapi* influenced by manipulation and cultivation by native peoples?

## 2. Materials and Methods

### 2.1. Taxon Sampling

**Figure 1:**
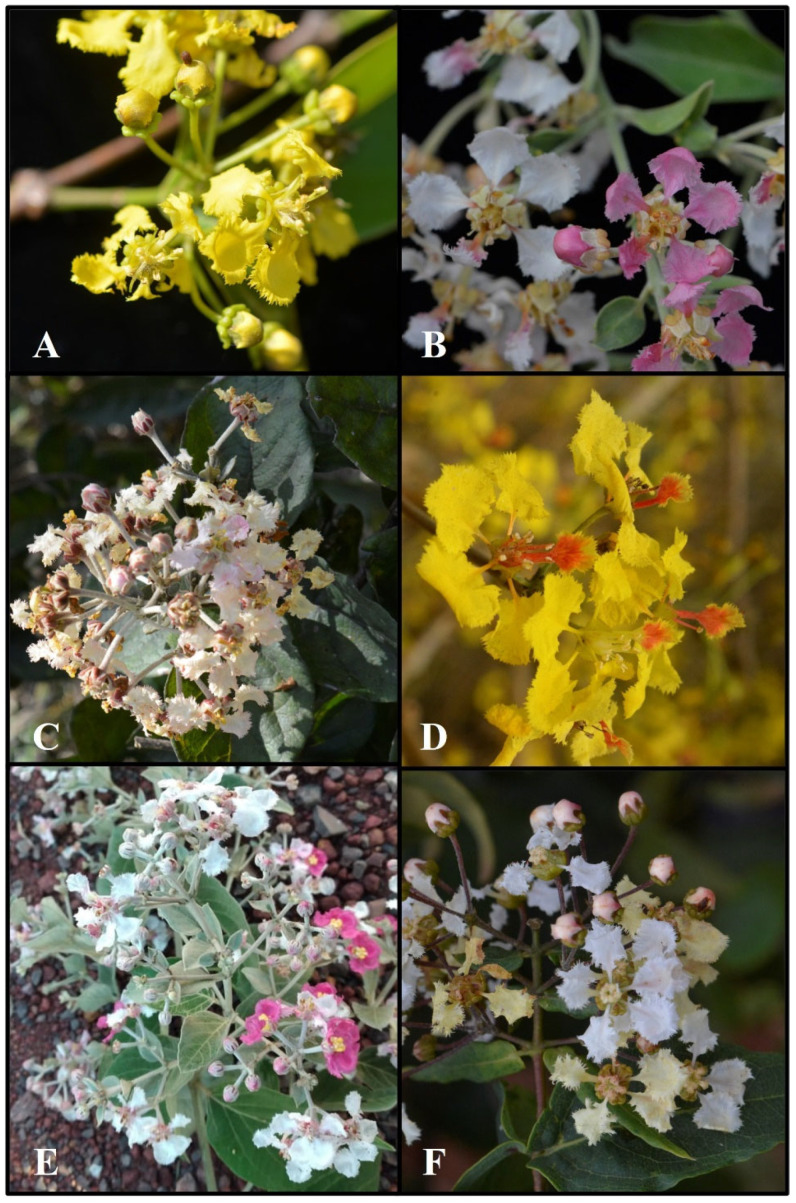
Some *Banisteriopsis* species sampled in this study. (**A**) *B. anisandra*; (**B**) *B. caapi*; (**C**) *B. malifolia*; (**D**) *B. martiniana*; (**E**) *B. oxyclada*; and (**F**) *B. stellaris*. Photos by R.C. Oliveira.

Thirty-eight *Banisteriopsis* species were sampled (more than 60% of representatives of the genus), 34 from previous phylogenetic studies of the family Malpighiaceae, plus four species sequenced for the first time in our study: *B. campestris* (A. Juss.) Little, *B*. *irwinii* B. Gates, *B. membranifolia* and *B*. *oxyclada* (A. Juss.) B. Gates (Supplementary Information S1, Figure 1). The inclusion of these four additional species aimed to maximize the geographical representation of the genus in this study. These species were selected to ensure broader geographical coverage, capture greater morphological diversity, and enhance the understanding of phylogenetic relationships within the genus. Fourteen species from seven other genera known to be phylogenetically close to *Banisteriopsis*, from the Stigmaphylloid clade (following [15]), were also included: *Bronwenia cinerascens* (Benth.) W.R. Anderson and C. Davis, *Bronwenia ferruginea* (Cav.) W.R. Anderson and C. Davis, *Diplopterys cabrerana* (Cuatrec.) B. Gates, *Diplopterys hypericifolia* (A. Juss.) W.R. Anderson and C. Davis, *Janusia anisandra* (A. Juss.) Griseb., *Janusia hexandra* (Vell.) W.R. Anderson, *Peixotoa cordistipula* A. Juss., *Peixotoa glabra* A. Juss., *Philgamia glabrifolia* Arènes, *Philgamia hibbertioides* Baill., *Sphedamnocarpus angolensis* Planch. ex Oliv., *Sphedamnocarpus poissonii* Arènes, *Stigmaphyllon aberrans* C.E. Anderson, and *Stigmaphyllon ciliatum* (Lam.) A. Juss. The species *Ectopopterys soejartoi* W.R. Anderson was used to root the tree (see [15,21] for more details) (Supplementary Information S1).

### 2.2. DNA Extraction, Amplification, and Sequencing

The nuclear regions ETS, ITS, and *PHYC*, and the plastid genes *mat*K, *ndh*F, and *rbc*L were used in our investigation. The sequences from [15] were obtained from GenBank [22]. DNA extractions of fresh samples were performed using the protocol adapted for microtubes [23], whereas for herbarium samples, the QuiaGen Mini Kit (Venlo, The Netherlands) was used. Extracted DNA was evaluated using electrophoresis in a 1% agarose gel.

The plastid region *ndh*F and the nuclear gene *PHYC* were amplified and sequenced using the same primers as those used by [15], but cloning was not performed for the *PHYC* region. Amplification of the plastid and nuclear regions was conducted through PCR reactions using a Top Taq Master Mix Kit (Qiagen, Venlo, The Netherlands), following the manufacturer’s recommendations, in a final volume of 15.4 µL, containing 0.2 µM of each primer and 25 ng of the DNA template.

The PCR products were purified using the method of DNA precipitation with polyethyleneglycol (PEG) 11% [24] and resuspended in autoclaved Milli-Q water. Sequencing reactions were performed on the sequencing platform of the Instituto Gonçalo Muniz, from the Fundação Oswaldo Cruz (FIOCRUZ), in Salvador, Brazil. Sequencing was conducted using BigDye Terminator V3.1, including precipitation with ethanol/EDTA, denaturation with formamide, and reading with capillary electrophoresis.

The generated electropherograms were analyzed and manually edited in the BioEdit program version 7.1 [25]. The initial and final portions of each sequence were eliminated to avoid artifacts near the annealing region of the primers. Sequence alignment was performed in the Mega version X [26] and PHYDE v.0.9971 [27] programs, using the MUSCLE [28] plugin with subsequent manual adjustments. More information on the sequence alignments and consensus trees for each molecular marker can be found in Supplementary Informations S2 and S3.

### 2.3. Phylogenetic Reconstruction and Estimation of Divergence Times

Phylogenetic reconstructions were inferred using maximum likelihood (ML) analyses performed with RAxML v.8.2.8 [29], using the rapid bootstrap algorithm with 1000 replicates to assess branch support, combined with a search for the best-scoring ML tree under default parameters. The Bayesian inference (BI) and divergence time estimation (DTE) were performed using BEAST v.1.10.4 [30]. The dataset was partitioned by molecular marker, and to select the best model of DNA substitution for each individual region in the combined dataset, we used jModelTest v.2.1.6 [31]. GTR-gamma was the best model for the regions ITS, *ndh*F, and *mat*K; GTR-invgamma for *PHYC*; GTR-inv for *rbc*L; and HKY-gamma for ETS.

To estimate the divergence times of the *Banisteriopsis* species, we used a calibration point for the Stigmaphylloid clade suggested by [21]. These authors used fossil pollen from *Perisyncolporites pokornyi* [32] at the crown node of the clade, dating back to 49 million years ago (Mya) (see [21] for more details). In our study, we applied an uncorrelated relaxed clock with a lognormal distribution of rates, in addition to a birth–death speciation model [33]. Although the Yule model [34] is recommended in cases when a single sequence per species is sampled in a phylogenetic hypothesis [35], it is a simple pure-birth process with a single parameter [34]. Its limitations, including the fact that it should not be used when not all extant species are included in the phylogeny, in addition to its other condition that assumes the birth rate of new lineages is the same throughout the tree [35], make this model inappropriate. Therefore, the two-parameter birth–death model has been suggested as an appropriate null model for species diversification [36] that can be applied to data on clade ages and diversities or fitted to the branching times in phylogenetic trees [37]. We also considered a standard deviation of 0.5 and a mean of 1.5 to follow the pattern of [21]. The Markov chain Monte Carlo (MCMC) was run twice for 150 million generation searches, with parameters sampled every 10,000 generations. The results of the individual runs were combined in LogCombiner v.1.10.4 [38], in which 10% of the burn-in was removed from the analysis. The maximum clade credibility tree, with a posterior probability limit of 0.95, was produced using the Tree Annotator v.1.10.4 program (see Supplementary Information S1 [38]). The RAxML, jModelTest, and BEAST analyses were performed on the CIPRES Science Gateway platform [39].

### 2.4. Biogeographic Analysis

Our sampling represents all major areas of diversification and geographical distributions of *Banisteriopsis*. We used literature surveys and data collected from fieldwork, herbaria, and online databases (https://specieslink.net/, https://www.jstor.org/, https://www.tropicos.org/home, and others) to define the geographical ranges of the studied species. Biogeographical areas were defined according to the biogeographical subregions of the Neotropics proposed by [40], as follows: A—Andes (South American transition zone *sensu* [40]), B—Amazon basin (Boreal Brazilian dominion and South Brazilian dominion *sensu* [40]), C—Cerrado (Cerrado province *sensu* [40]), D—Caatinga (Caatinga province *sensu* [40]), E—Atlantic Forest (Atlantic, Parana Forest, and *Araucaria* Forest provinces *sensu* [40]), F—Chaco (Chacoan province *sensu* [40]), and G—Central America (Pacific dominion *sensu* [40]) and the West Indies (Antillean subregion *sensu* [40]).

We carried out ancestral range reconstruction analyses testing the fit of S-DIVA, DEC, and BAYAREA models to our data in the RASP v.4 software [41] using the BioGeoBEARS v.0.2.1 package in R. S-DIVA to calculate statistical support for the ancestral ranges at each node of the phylogenetic tree by applying lower “costs” to vicariance events [42]. The DEC model simulates events along a phylogenetic branch according to the proportion of the branch length and the transition rates between geographic areas, assuming that dispersal mediates range expansion and extinction mediates range contraction [43]. BAYAREA uses “data-augmentation” to operate a continuous-time Markov chain for simulating colonizations and local extinctions [44]. All three models calculate the probability of each event for every node in a phylogeny.

We used 10,000 trees generated from the combination of each of the four Markov chains used to estimate divergence times. We randomly selected 1000 from the 10,000 available trees to calculate the mean frequency of ancestral geographic range. For each analysis, we ran two independent chains for 10,000,000 generations, sampling every 1000 generations. Akaike weights were calculated for alternative models using the function implemented in RASP v.4. To select the best model explaining the ancestral range of *Banisteriopsis*, we used the highest AICw value. In this analysis, we applied one scenario without constraints on dispersal. Finally, we calculated the dispersal rates and vicariance events over time in RASP and the rate of lineages through time in the ape 5.0 package [45].

## 3. Results

### 3.1. Phylogenetic Analysis

**Figure 2:**
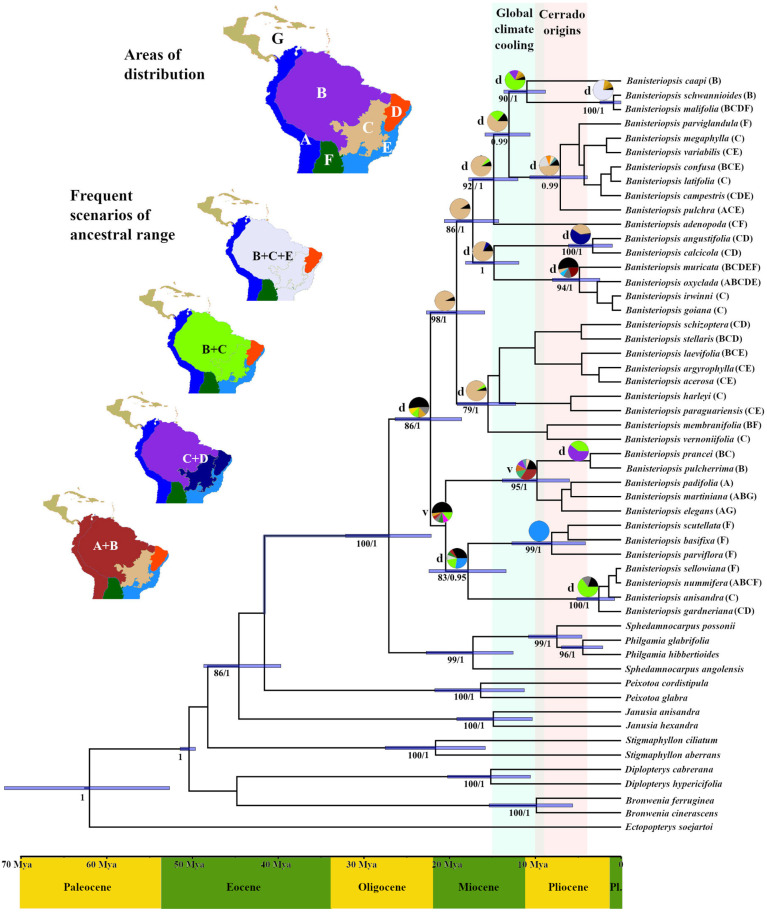
The BEAST chronogram from the Bayesian analysis of divergence times (Mya) of Stigmaphylloids using the entire molecular dataset and ancestral range reconstruction of *Banisteriopsis*, including 38 species from this genus, along with 15 closely related species. The blue bars represent 95% confidence intervals for the age of clades. The numbers below the branches represent maximum likelihood bootstrap (BS) and Bayesian posterior probability (PP), with PP < 0.95 omitted. The pie charts above the branches show posterior probabilities of ancestral ranges estimated using the DEC model. The letters after the species names and colors in the pie charts are coded according to inset maps that indicate actual and possible geographical ranges. The lowercase letters before the pie charts indicate evolutionary events: d = dispersal; v = vicariance. The black slices in the pie charts indicate indeterminate ancestral ranges. Geographical range categories: A = Andes; B = Amazon basin; C = Cerrado, D = Caatinga, E = Atlantic Forest, F = Chaco, and G = Central America and the West Indies. Pl. = Pleistocene.

The nuclear data matrix (ETS, ITS, and *PHYC*) had 1993 characters and the plastid data matrix (*mat*K, *ndh*F, and *rbc*L) had 3179 characters, resulting in a total of 5172 characters analyzed. The genus *Banisteriopsis* was recovered as monophyletic (bootstrap support [BS] = 86; posterior probability [PP] = 1). Within it, a poorly supported group of species composed of two clades is sister to the remaining *Banisteriopsis* species. Within that group, a clade formed by *B. anisandra* (A. Juss.) B. Gates, *B. basifixa* B. Gates, *B. gardneriana* (A. Juss.) W.R. Anderson and B. Gates, *B. nummifera* (A. Juss.) B. Gates, *B. parviflora* (A. Juss.) B. Gates, *B. scutellata* (Griseb.) B. Gates, and *B. sellowiana* (A. Juss.) B. Gates (BS = 83; PP = 0.95) is sister to the clade composed of *B. elegans* (Triana and Planch.) Sandwith, *B. martiniana* (A. Juss.) Cuatrec., *B. padifolia* (Poepp. ex Nied.) B. Gates, *B. prancei* B. Gates, and *B. pulcherrima* (Sandwith) B. Gates (BS = 95; PP = 1) (Figure 2). This broad group is composed of species from a wide range of origins, but mainly from the Andean and Atlantic Forest.

The remaining *Banisteriopsis* species form two main lineages, mostly of species that occur in the Cerrado. A clade (BS = 79, PP = 1) composed of *B. acerosa* (Nied.) B. Gates, *B. agyrophylla* (A. Juss.) B. Gates, *B. harleyi* B. Gates, *B. laevifolia* (A. Juss.) B. Gates, *B. membranifolia*, *B. paraguariensis* B. Gates, *B. schizoptera* (A. Juss.) B. Gates, *B. stellaris* (Griseb.) B. Gates, and *B. vernoniifolia* is sister to the most species-rich lineage (BS = 86, PP = 1). This latter lineage includes *B. adenopoda* (A. Juss.) B. Gates, *B. angustifolia* (A. Juss.) B. Gates, *B. caapi*, *B. calcicola* B. Gates, *B. campestris*, *B. confusa* B. Gates, *B. goiana* B. Gates, *B. irwinii*, *B. latifolia* (A. Juss.) B. Gates, *B. malifolia* (Nees and Mart.) B. Gates, *B. megaphylla* (A. Juss.) B. Gates, *B. muricata* (Cav.) Cuatrec., *B. oxyclada*, *B. parviglandula* B. Gates, *B. pulchra* B. Gates, *B. schwannioides*, and *B. variabilis* B. Gates (Figure 2).

### 3.2. Divergence Times and Reconstruction of Ancestral Areas

Divergence time analysis showed that the ancestor of the *Banisteriopsis* species originated in the Early Miocene, approximately 22.24 Mya (95% Highest Posterior Density (HPD) 18.6–26.3 Mya), within an unknown ancestral area (Figure 2). Around 19.2 Mya (95% HPD 15.9–22.7 Mya), also in the Early Miocene, an old divergence event within the genus occurred, leading to the diversification that originated the most species-rich lineage of the genus, with the Cerrado as its most likely ancestral area. Three other ancient lineages were also recovered for the Early Miocene. The clade composed of *B. anisandra*, *B. basifixa*, *B. gardneriana*, *B. nummifera*, *B. parviflora*, *B. scutellata*, and *B. sellowiana* has an Atlantic Forest ancestor dating to around 17.86 Mya (95% HPD 13.4–22.4 Mya), whereas the other two ancient lineages have their ancestors in the Cerrado, dating approximately 17.31 (95% HPD 14.3–24.6 Mya) and 15.53 Mya (95% HPD 12.3–19.1 Mya), respectively.

Other important most recent common ancestors (MRCA) for the genus diversified during the Late Miocene: the Amazon basin plus Cerrado was recovered as the ancestral area for the clade composed of *B. caapi*, *B. malifolia*, and *B. schwannioides* around 10.99 Mya (95% HPD 8.8–13.7 Mya); an Andes plus Amazon basin was recovered as the most likely ancestor for the clade composed of *B. elegans*, *B. martiniana*, *B. padifolia*, *B. prancei*, and *B. pulcherrima* around 9.82 Mya (95% HPD 6–13.8 Mya); an Atlantic Forest ancestor was identified for the clade composed of *B. basifixa*, *B. parviflora*, and *B. scutellata* around 8.1 Mya (95% HPD 4.1–12.7 Mya); and the Cerrado was recovered as the most likely ancestral area for the clade composed of *B. campestris*, *B. confusa, B. latifolia*, *B. megaphylla*, *B. parviglandula*, *B. pulchra*, and *B. variabilis* around 7.1 Mya (95% HPD 3.9–10.6 Mya). The Cerrado was identified as the most likely ancestral area for the majority of the *Banisteriopsis* lineages, with most of the *Banisteriopsis* species diverging around 2.5–5 Mya in the Pliocene (Figure 2).

The DEC model was selected as the best-fit model (LnL = −145.3, AICc = 295, AICw = 0.99) against BAYAREA (LnL = −157.3, AICc = 318.9, AICw < 0.01) and S-DIVA (LnL = −152.8, AICc = 309.9, AICw < 0.01). However, the level of uncertainty prevents a precise definition of the ancestral range of the MRCA of *Banisteriopsis*. The DEC model recovered fifty dispersal events and four vicariance events throughout the evolutionary history of *Banisteriopsis*. The main dispersal and vicariance events are highlighted in Figure 2.

**Figure 3:**
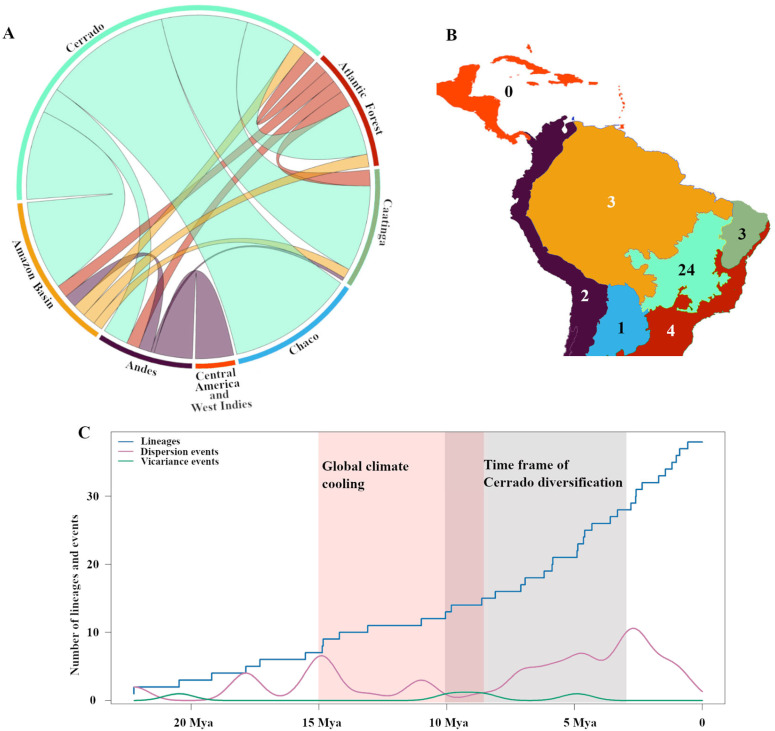
The dynamics of biotic interchange, vicariance, and lineage accumulation throughout the evolutionary history of *Banisteriopsis*, according to the results of the selected biogeographic model. (**A**) Biotic interchange between the areas. Line widths represent the proportion of interchange between the source and destination areas of dispersal. (**B**) Map of the areas showing the number of estimated events of speciation. (**C**) Dynamics of dispersal, vicariance, and lineage accumulation over time. Plot colors and area names in A are coded according to the inset map in B.

The Cerrado was identified as the main source of biotic elements of *Banisteriopsis* for the Amazon basin, Andes, Atlantic Forest, Caatinga, and Chaco. Dispersal events from the Cerrado were not detected only for Central America and the West Indies. Reconstruction analysis revealed that most of these dispersal events occurred from the Cerrado to the Chaco, Amazon basin, and Caatinga, all of which are biomes adjacent to the Cerrado (Figure 3A). The Cerrado was also confirmed as the main center of the speciation process in the evolutionary history of *Banisteriopsis* (Figure 3B). Dispersal cycles presented peaks around 18 Mya, 15 Mya, and 2.5 Mya, whereas vicariance events were concentrated around 9 Mya (Figure 3C). Dispersal events were associated with an increase in the number of lineages in the evolutionary history of *Banisteriopsis*.

## 4. Discussion

### 4.1. Origin of the Genus Banisteriopsis and Biome Transitions

*Banisteriopsis* likely originated in the Early Miocene around 22 Mya, with its diversification beginning to intensify around 19 Mya in the Cerrado. This period coincides with the expansion of seasonally dry areas within the diagonal of open formations in South America, in addition to the formation of the Atacama Desert [46]. Gates hypothesized a Cerrado origin for *Banisteriopsis* during the Pleistocene [14]. Although we cannot confirm or refute the Cerrado origin for the genus due to our lack of resolution, our findings indicate an earlier colonization of the Cerrado by the most species-rich lineage of *Banisteriopsis* in the Early Miocene, contradicting the previously proposed recent Pleistocene origin of the genus.

At approximately 15 Mya after the Mid-Miocene climatic optimum, a period of drastic reduction in global temperatures led to the expansion of dry areas in South America [47]. This expansion was further intensified by the orogenic activity of the Andes Mountain range during the Oligocene–Miocene transition, which resulted in the formation of a significant climatic barrier [46,47,48,49]. Thus, the evidence presented here suggests that the expansion of dry areas in South America, resulting from Miocene climatic cooling and the Andes orogeny, coincides with the period of origin and diversification of *Banisteriopsis.*

Our results demonstrate that *Banisteriopsis* likely colonized the Cerrado earlier than predicted by the most widely accepted previous hypothesis [17]. Indeed, the time of colonization of the Cerrado by *Banisteriopsis* predates that of other groups of plants, such as *Andira* Lam., *Mimosa* L., and *Chamaechrista* (L.) Moench (Fabaceae) [48,50,51], *Caraipa* Aubl. and *Kielmeyera* Mart. and Zucc. (Calophyllaceae) [52], *Eriotheca* (Malvaceae) [53], *Eugenia* L., and *Myrcia* DC. (Myrtaceae) [54,55], which diversified less than 10 Mya. It has been proposed that the Cerrado biota primarily resulted from processes of fire adaptation rather than the dispersal of lineages already adapted to fire [48]. Fire acts as the main environmental filter in the Cerrado, as many species are dependent on seasonal forest fires for flowering and seed germination [48,50]. Thus, the importance of fire and the process of adaptation of biota place the main events forming Cerrado biodiversity around 8–10 Mya [48,50].

Therefore, the evolutionary history of *Banisteriopsis* reinforces the body of evidence suggesting the need to revisit the current theory that the formation of the Cerrado biota is the result of recent colonization and diversification processes [48]. However, *Banisteriopsis* is not the only group that exemplifies older ages for Cerrado colonization than predicted by the current model. For example, refs. [56,57] reported a Cerrado colonization event around 15 Mya for *Stigmaphyllon* A. Juss. (Malpighiaceae) and for *Myrcia* sect. *Aguava* (Raf.) D.F. Lima and E. Lucas (Myrtaceae), respectively. Furthermore, earlier savanna-like biomes were also hypothesized by [58,59] for the Paleocene–Oligocene period. Ref. [60] uncovered a long history of niche conservatism in the Succulent Biomes of South America, although they highlighted that the dynamic exemplified by *Cenostigma* Tul. (Fabaceae) reinforces the recent assembly of the Cerrado.

The evolutionary history of *Banisteriopsis* illustrates several examples of biome transitions. In the vast majority of cases, species of the genus colonized biomes adjacent to the Cerrado, such as the Amazon and Atlantic Forest. This is an inverse process to that recorded for most of the Neotropical plants, where the Amazon Forest acted as the main source of biodiversity for colonizing other biomes [61]. In addition, biome transitions were rare events among Neotropical plants [16], as was the colonization of forest areas by savanna vegetation lineages [16,61], highlighting the non-standard trends found in our study.

The Cerrado occupies the central region of Brazil, covering an area of about 2,000,000 km^2^, with extensive contact zones with the two tropical humid rainforests (the Amazon and Atlantic Forest) [48]. It is considered a biodiversity hotspot, encompassing three of the largest hydrographic basins in South America and having an enormous importance for species conservation and ecosystem services [62]. One of the physiognomic types found in the Cerrado is a riverine humid forest named Gallery Forest, which is enclaved in savanna vegetation. The Gallery Forest has served as a refuge during periods of savanna expansion or as a corridor when the Atlantic and Amazon Forests underwent augmentation [63,64].

During the climate change cycles experienced over the last 10 Mya, the Gallery Forests expanded, allowing the establishment of two biological corridors between the Cerrado and the humid forests of South America: in the northwest, contact occured with the Amazon region, and in the east and southeast, with the Atlantic Forest [63,64]. These paleoenvironmental dynamics and the extensive contact zones must have been the causes of the most intense biotic exchanges between adjacent biomes and the ancestral area of the genus, according to the results obtained in our work. Such biotic interchange impressed an evolutionary signature on the history of several taxa that diversified in the Cerrado [48,50,51,52,53,54,55].

### 4.2. Wide Ancient Forest as the Origin of B. caapi

The clade formed by *B. caapi*, *B. malifolia*, and *B. schwannioides* diverged in the Late Miocene around 10.99 Mya, originating and diversifying in a broad area encompassing the Amazon basin and Cerrado. This period in the Middle to Late Miocene follows and overlaps (7.28–16.9 Mya) the Langhian (~14 Mya), known as the Miocene Climatic Optimum (MCO; [65]), when forests expanded. This supports our hypothesis of a broader forested area encompassing what is now the Cerrado and Amazon basin. This hypothesis should be considered, as until the Early Miocene (~16 Mya), the paleovegetation of the northern and northeastern portions of South America was characterized by tropical rainforests [66]. Over time, several forces influenced Amazonian Neotropical biodiversity, including the uplift of the Andes. One consequence of the Andean uplift was the emergence and drainage of the Pebas system in the western Amazon, a river system of lakes and swamps interspersed with mountains [67,68]. The drainage of the Pebas system implied the in-situ diversification of lineages that adapted to dry land and facilitated the dispersal of Andean taxa across most of the region [67,68]. This dynamic must have enabled the adaptation of the common ancestor of the clade containing *B. caapi*, which transitioned from the Cerrado and diversified into a wet forest environment.

Unlike species that are recognized as the result of human interference, such as *cupuaçu* (*Theobroma grandiflorum*, Malvaceae) [69], our results indicate an ancient diversification of *B. caapi*. Previous divergences of cultivated plants have been reported in some studies. Examples include *biribá* (*Annona mucosa* Jacq., Annonaceae) [70], Brazil nut (*Bertholletia excelsa* Bonpl., Lecythidaceae) [71], and *guava* (*Psidium guajava* L., Myrtaceae) [72], which do not support the idea that the origin of these species has been influenced by human manipulation. The first undeniable evidence of human occupation in South America dates back to 13,000 before present (BP), found in archaeological sites located between Belize and eastern Brazil [73]. Ref. [74] proposes a widely accepted hypothesis emphasizing the Mesoamerican influence in South America. However, there is abundant evidence of a network of exchanges, including the trade of entheogenic plants, among Pacific fishermen, Andean populations, and Amazonian groups [75].

Chemical evidence of harmine and/or tryptamine in mummies and archaeological artefacts suggests the consumption of *Banisteriopsis* in the Altiplano regions of Chile, Bolivia, and Peru [76,77,78,79], dating back 1000 years [79]. The presence of harmine and/or tryptamine alone suggests that the consumption of *Banisteriopsis* was not intended for hallucinogenic purposes, as this beta-carboline is a monoamine oxidase inhibitor with psychoactive effects but no hallucinogenic properties [76,77,78,79]. The use of *Banisteriopsis* for medicinal purposes or as protection against the evil eye, among other uses, is still documented in Amazonian states of Brazil [80].

The establishment that *B. caapi* originated in the South American Amazon supports these archaeological findings. Ancient populations [81] and non-Indigenous groups, such as Brazilian syncretic Ayahuascan religious organizations [82,83], recognize the diverse ethnotaxa of *B. caapi* based on stem and leaf morphology, chemical effects, and tea colors [82]. DNA barcoding analyses of 16 ethnotaxa of *B. caapi* [83] resulted in a phylogeny with three clades, without correspondence to ethnotaxa. However, one clade includes more native taxa, whereas the others comprise cultivated ones [83]. These findings corroborate archaeological studies that have revealed that, throughout the Holocene, complex societies in the Amazon region modified the environment by managing and domesticating plants [84].

Future studies using additional genetic markers and more comprehensive *B. caapi* samples, including herbarium vouchers, which were lacking in [83], for example, should clarify the relationships between some of the free-living and cultivated ethnotaxa. Such studies would contribute to a better understanding of archaeological networks and the domestication of entheogenic species in South America.

## 5. Conclusions

The results obtained in this study confirmed that the diversification of *Banisteriopsis* began to occur simultaneously with the expansion of seasonally dry areas in South America. However, *Banisteriopsis* started colonizing the Cerrado at an earlier period. The evolutionary history of the genus also reveals that the Cerrado served as a source of species for Neotropical rainforests, such as the Amazon and the Atlantic Forest, an inverse pattern compared to that recorded for the majority of Neotropical plants. Several biome transition events have been recorded throughout the evolutionary history of the genus, and many of them are associated with speciation events.

## Data Availability

The original contributions presented in this study are included in the article/Supplementary Materials. Further inquiries can be directed to the corresponding author.

## Supplementary Materials

The following supporting information can be downloaded at: https://www.mdpi.com/article/10.3390/plants14071149/s1.
