# Supplementary material for: Dated Phylogeny of Banisteriopsis (Malpighiaceae) Suggests an Ancient Colonization of the Cerrado and No Evidence of Human Manipulation in the Origin of B. caapi
Source: Plants (Basel). 2025 Apr 7;14(7):1149. doi: 10.3390/plants14071149 (PMC11990928; doi:10.3390/plants14071149)
Supplement: Supplementary file 1 [file plants-14-01149-s001.zip › Supplementary information 1.pdf]

Supplementary information 1

Taxa included in the analyses, along with collector name, collection number, herbarium acronym, and GenBank accession numbers for the sequences *ETS*, *ITS*, *matK*, *ndhF*, *PHYC* and *rbcL*. * New sequences obtained in this study.

**Taxon**	**Voucher**	***ETS***	***ITS***	***matK***	***ndhF***	***PHYC***	***rbcL***
Banisteriopsis acerosa (Nied.) B.Gates	Guala 1370 (MICH)			HQ247197		HQ246965	
B. adenopoda (A.Juss.) B.Gates	Villagra 45 (SP)	KR092987	KR093003				
	Silva 1109 (MICH)				HQ246750	HQ246966	HQ247438
B. angustifolia (A.Juss.) B.Gates	Carvalho 80 (MICH)			HQ247198	HQ246751	HQ246967	
	Almeida 605 (HUEFS)	KR092988	KR093004				
B. anisandra (A.Juss.) B.Gates	Kirkbride 4865 (MICH)			KM197226	KM197319		KM197468
	Almeida 611 (HUEFS)	KR092989	KR093005				
B. argyrophylla (A.Juss.) B.Gates	Sebastiani & Moreno 87 (SP)	KR092990	KR093006				
	Silva 3548 (MICH)			HQ247199	HQ246752	HQ246968	HQ247439
B. basifixa B.Gates	Anderson 13678 (MICH)			KM197227	KM197320		
B. caapi (Spruce ex Griseb.) C.V.Morton	Anderson 13791 (MICH)			HQ247200	HQ246753	HQ246969	HQ247440
	Sebastiani 248 (SP)	KR092991					
B. calcicola B.Gates	Lombardi 1690 (MICH)			HQ247201	HQ246754	HQ246970	
B. campestris (A.Juss.) Little	Lima 10 (UB)*				PQ871209	PQ871210	
B. confusa B.Gates	Krapovickas & Cristóbal 44854 (MICH)			HQ247202	HQ246755	HQ246971	
B. elegans (Triana & Planch.) Sandwith	Albert de Escobar 3294 (MICH)			KM197228	KM197322		KM197469
B. gardneriana (A.Juss.) W.R.Anderson & B.Gates	Azevedo & Lopes 313 (MICH)			HQ247203		HQ246972	
B. goiana B.Gates	Dias et al. 531 (MICH)			KM197229	KM197323		
	Resende 798 (SP)	KR092992					
B. harleyi B.Gates	Conceição 1713 (HUEFS)	KR092993	KR093007				
	Carvalho 87 (MICH)			HQ247204	HQ246756	HQ246973	
B. irwinii B.Gates	Kirbride 3184 (UB)*				ON109233		
B. laevifolia (A.Juss.) B.Gates	Francener 1359 (SP)		KR093008				
	Anderson 13631 (MICH)			HQ247205	HQ246757	HQ246974	HQ247441

B. latifolia (A.Juss.) B.Gates	Azevedo 698 (MICH)		KR093008				
	Francener 1187 (SP)	KR092994		HQ247206	HQ246758	HQ246975	
B. malifolia (Nees & Mart.) B.Gates	Mori et al. 21590 (MICH);			KM197225	KM197318	KM197455	KM197467
	Almeida 576 (HUEFS)	KR092995	KR093009				
B. martiniana (A.Juss.) Cuatrec.	Chase 90161 (MICH)			HQ247207	HQ246759	HQ246976	HQ247442
B. megaphylla (A.Juss.) B.Gates	Heringer et al. 6205 (MICH)			KM197230	KM197324		
B. membranifolia (A.Juss.) B.Gates	Kirbride 4832 (UB)*				ON109234		
B. muricata (Cav.) Cuatrec.	Anderson 13799 (MICH)			HQ247208	HQ246760	HQ246977	HQ247443
	Almeida 545 (HUEFS)	KR092996	KR093010				
B. nummifera (A.Juss.) B.Gates	Harley 54681 (MICH)			HQ247209	HQ246761	HQ246978	HQ247444
B. oxyclada (A.Juss.) B.Gates	Oliveira 3827 (UB)*				PQ871211		
B. padifolia (Poepp. ex Nied.) B.Gates	Matezki 320 (MICH)			KM197231	KM197325		
B. paraguariensis B.Gates	Ferrucci 1624 (MICH)			HQ247210	HQ246762	HQ246979	
B. parviflora (A.Juss.) B.Gates	Hatschbach 54117 (MICH)				KM197326		KM197470
B. parviglandula B.Gates	Lombardi 697 (SP)	KR092997					
B. prancei B.Gates	Anderson 9910 (MICH)					HQ246980	
B. pulcherrima (Sandwith) B.Gates	Maguire et al. 53669 (MICH)			KM197232	KM197327		
B. pulchra B.Gates	Dubs 2258 (MICH)			HQ247211	HQ246763	HQ246981	
B. schwannioides (Griseb.) B.Gates	Mori et al. 21590 (MICH)			HQ247212	HQ246764	HQ246982	HQ247445
B. schizoptera (A.Juss.) B.Gates	Anderson et al. 36421 (MICH)				KM197328		
B. scutellata (Griseb.) B.Gates	Kollmann 2511 (MICH)				KM197329		KM197471
B. sellowiana (A.Juss.) B.Gates	Menendro 278 (MICH)				HQ246765	HQ246983	
B. stellaris (Griseb.) B.Gates	Santos et al. 673 (MICH)			KM197233	KM197330		KM197472
	Francener 1123 (SP)	KR092998	KR093011				
B. variabilis B.Gates	Francener 925 (SP)	KR092999	KR093012				
B. vernoniifolia (A.Juss.) B.Gates	Cavalcanti et al. 1288 (MICH)			KM197234	KM197331		
	Hatschbach70734 (MBM)	KR093000	KR093013				
Bronwenia cinerascens (Benth.) W.R.Anderson & C.Davis	Nee 48570 (MICH)			HQ247216	HQ246769	HQ246987	HQ247450
B. ferruginea (Cav.) W.R.Anderson & C.Davis	Amorim et al. 3222 (MICH)			HQ247218	HQ246771	HQ246989	HQ247452

Diplopterys cabrerana (Cuatrec.) B.Gates	Burnham 1774 (MICH)		HQ247266	AF351039	AF500582	HQ247482
D. hypericifolia (A.Juss.) W.R.Anderson & C.Davis	Anderson 13638 (MICH)		AF344530	AF351037	HQ247042	AF344460
Ectopopterys soejartoi W.R.Anderson	Callejas 11806 (MICH)		HQ247272	AF351064	AF500565	AF344471
	Wurdach 2356 (US)	KR092910				
Janusia anisandra (A.Juss.) Griseb.	Anderson 13694 (MICH)		AF344553	AF351028	AF500536	AF344485
J. hexandra (Vell.) W.R.Anderson	Thomas et al. 11423a (MICH)		HQ247322	HQ246862	HQ247086	HQ247532
Peixotoa cordistipula A.Juss.	Gerlach et al. 26/01 (MICH)		HQ247370	HQ246907	HQ247133	HQ247567
P. glabra A.Juss.	Anderson 13636 (MICH)		AF344571	AF351036	AF500517	AF344504
Philgamia glabrifolia Arènes	Schatz et al. 4098 (P)		HQ247374	HQ246910	HQ247137	HQ247568
P. hibbertioides Baill.	Du Puy et al. M635 (P)		HQ247375	HQ246911	HQ247138	HQ247569
Sphedamnocarpus angolensis Planch. ex Oliv.	Brummitt & Pope 19597 (MICH)		HQ247381	HQ246916	HQ247143	HQ247576
S. poissonii Arènes	Dorr 3959 (P)		HQ247382	HQ246917	HQ247145	HQ247577
Stigmaphyllon aberrans C.E.Anderson	Rojas et al. 2347 (MICH)		HQ247387	HQ246919	HQ247148	HQ247581
S. ciliatum (Lam.) A.Juss.	Anderson 13739 (MICH)		HQ247390	HQ246923	HQ247151	HQ247584
