# Supplementary material for: Dated Phylogeny of Banisteriopsis (Malpighiaceae) Suggests an Ancient Colonization of the Cerrado and No Evidence of Human Manipulation in the Origin of B. caapi
Source: Plants (Basel). 2025 Apr 7;14(7):1149. doi: 10.3390/plants14071149 (PMC11990928; doi:10.3390/plants14071149)
Supplement: Supplementary file 1 [file plants-14-01149-s001.zip › Supplementary information 2.pdf]

Supplementary Information 1

Alignment in nexus format

```
begin taxa;
	dimensions ntax=53;
	taxlabels
	Banisteriopsis_acerosa[&description="Banisteriopsis_acerosa"]
	Banisteriopsis_adenopoda[&description="Banisteriopsis_adenopoda"]
	Banisteriopsis_angustifolia[&description="Banisteriopsis_angustifolia"]
	Banisteriopsis_anisandra[&description="Banisteriopsis_anisandra"]
	Banisteriopsis_argyrophylla[&description="Banisteriopsis_argyrophylla"]
	Banisteriopsis_basifixa[&description="Banisteriopsis_basifixa"]
	Banisteriopsis_caapi[&description="Banisteriopsis_caapi"]
	Banisteriopsis_calcicola[&description="Banisteriopsis_calcicola"]
	Banisteriopsis_campestris[&description="Banisteriopsis_campestris"]
	Banisteriopsis_confusa[&description="Banisteriopsis_confusa"]
	Banisteriopsis_elegans[&description="Banisteriopsis_elegans"]
	Banisteriopsis_gardeneriana[&description="Banisteriopsis_gardeneriana"]
	Banisteriopsis_goiana[&description="Banisteriopsis_goiana"]
	Banisteriopsis_harleyi[&description="Banisteriopsis_harleyi"]
	Banisteriopsis_irwinni[&description="Banisteriopsis_irwinni"]
	Banisteriopsis_laevifolia[&description="Banisteriopsis_laevifolia"]
	Banisteriopsis_latifolia[&description="Banisteriopsis_latifolia"]
	Banisteriopsis_malifolia[&description="Banisteriopsis_malifolia"]
	Banisteriopsis_martiniana[&description="Banisteriopsis_martiniana"]
	Banisteriopsis_megaphylla[&description="Banisteriopsis_megaphylla"]
	Banisteriopsis_membranifolia[&description="Banisteriopsis_membranifolia"]
	Banisteriopsis_muricata[&description="Banisteriopsis_muricata"]
	Banisteriopsis_nummifera[&description="Banisteriopsis_nummifera"]
	Banisteriopsis_oxyclada[&description="Banisteriopsis_oxyclada"]
	Banisteriopsis_padifolia[&description="Banisteriopsis_padifolia"]
	Banisteriopsis_paraguariensis[&description="Banisteriopsis_paraguariensis"]
	Banisteriopsis_parviflora[&description="Banisteriopsis_parviflora"]
	Banisteriopsis_parviglandula[&description="Banisteriopsis_parviglandula"]
	Banisteriopsis_prancei[&description="Banisteriopsis_prancei"]
	Banisteriopsis_pulcherrima[&description="Banisteriopsis_pulcherrima"]
	Banisteriopsis_pulchra[&description="Banisteriopsis_pulchra"]
	Banisteriopsis_schizoptera[&description="Banisteriopsis_schizoptera"]
	Banisteriopsis_schwannioides[&description="Banisteriopsis_schwannioides"]
	Banisteriopsis_scutellata[&description="Banisteriopsis_scutellata"]
	Banisteriopsis_sellowiana[&description="Banisteriopsis_sellowiana"]
	Banisteriopsis_stellaris[&description="Banisteriopsis_stellaris"]
	Banisteriopsis_variabilis[&description="Banisteriopsis_variabilis"]
	Banisteriopsis_vernoniifolia[&description="Banisteriopsis_vernoniifolia"]
	Bronwenia_cinerascens[&description="Bronwenia_cinerascens"]
	Bronwenia_ferruginea[&description="Bronwenia_ferruginea"]
	Diplopterys_cabrerana[&description="Diplopterys_cabrerana"]
	Diplopterys_hypericifolia[&description="Diplopterys_hypericifolia"]
	Ectopopterys_soejartoi[&description="Ectopopterys_soejartoi"]
	Janusia_anisandra[&description="Janusia_anisandra"]
	Janusia_hexandra[&description="Janusia_hexandra"]
	Peixotoa_cordistipula[&description="Peixotoa_cordistipula"]
	Peixotoa_glabra[&description="Peixotoa_glabra"]
	Philgamia_glabrifolia[&description="Philgamia_glabrifolia"]
	Philgamia_hibbertioides[&description="Philgamia_hibbertioides"]
	Sphedamnocarpus_angolensis[&description="Sphedamnocarpus_angolensis"]
	Sphedamnocarpus_poissonii[&description="Sphedamnocarpus_poissonii"]
	Stigmaphyllon_aberrans[&description="Stigmaphyllon_aberrans"]
	Stigmaphyllon_ciliatum[&description="Stigmaphyllon_ciliatum"]
```

```
;
end;

begin characters;
	dimensions nchar=5172;
	format datatype=dna missing=? gap=-;
	matrix
	Banisteriopsis_acerosa	----------------------------------------------------------------------------------------
	---------------------------------------------------------------------------------------------------------------------------
	---------------------------------------------------------------------------------------------------------------------------
	---------------------------------------------------------------------------------------------------------------------------
	---------------------------------------------------------------------------------------------------------------------------
	---------------------------------------------------------------------------------------------------------------------------
	---------------------------------------------------------------------------------------------------------------------------
	---------------------------------------------------------------------------------------------------------------------------
	---------------------------------------------------------------------------------------------------------------------------
	-----------------------------------------------------------------------------------------------------------------
	ATTTAAATTATGTGTCTGATTCACTAATACCTTACCCCATCCATCTAGAAAAATTGGTT
	CAAATCCTTCGTTACTGGATGCAAGATCCCTCTTCTTTGCATTTATTACGACTCTTTCTT
	CACGAGTATTGGAATTGGAATAGTTTTATTATTTCAACGAAATCTTTTTCCATTTTTTT
	TAATTTAAAAAGGAATCTAAGATTATTGTTGTTCCTATATAATTCTCATGTATATGAAT
	ACGAATCCATCTTCTTTTTTCTCCGTAACCAATCCTTTTATTTCCAATCAACATTTTCGG
	GGGTGCTTCTTGAACGAATATATTTCTATGGAAAAATACAAGATTTTGTAGAACTTTT
	TACTAATG------
	ATTTTAAGGCTAACCTACGGTTGTTCAAGGATCCTTTCATGCATTATGTTAGATATCAA
	GGAAAAGGCATTCTGGCTTCAAAAAGTACACTTCTTCTGATGAAAAAATGGAAATATT
	TCTTTGTCAATTTATGTCAATTTCATTTTTATGTGTGGGTTCATCCAGAACCGATCTAT
	ATGAAGTCATTATCCAACCATTCTCTTGACTTTTTGGGTTATCTTTCAAATATACGAAA
	AAATCTTTCAGTGGTACGGAGTCAAATGCTAGAAAATTCATTTCTAATAGATAATATT
	ATGAAGAAACTCGATTCAAGAGTTCCAATTATTCCTTTGATTGGATCATTGTCAAAAA
	CAAAATTTTGTAACGCAGTAGGGCATCCCATTAGTAAACCGAGCTGGGCGGATTCACC
	CGATTCTGACATTATCGACCAATTTTTGCGTATATCCAGAAATCTTTCTCATTACTATA
	GCGGATCCTCAAAAAAAAAGAATTTGTATCGAGTAAAATATATACTTCGACTTTCTTG
	TGTTAAAACTTTGGCTCGTAAACACAAAAGTACCGTCCGGGCTTTTTTGAAAAAACTA
	GGTTCAGAATTATTGGAAGAATTTTTTACGGAGGAAAAATCAATTCTTGATTTGGTCT
	TCCCCACGGCTTATTCTATTTCGCGAAGGTTATACAGAGGGCGGGTTTGGTATTTGGA
	TATTATTTGTATCAATGATCTAGCCAATCATGAATAA---------------------------------------------
	---------------------------------------------------------------------------------------------------------------------------
	---------------------------------------------------------------------------------------------------------------------------
	---------------------------------------------------------------------------------------------------------------------------
	---------------------------------------------------------------------------------------------------------------------------
	---------------------------------------------------------------------------------------------------------------------------
	-------------------------------------------------------------------------------------------------
	GGCTTCAAGGTTTCTTTTCATGAAAAACAAGGTACGCATGATATGCGATTGTCTGGCC
	CCTCCAGTCAAGGTAATCCAAGACAAGAGATTGCCTCAGCCGTTAAGTCTTTGTGGGT
	CTACATTAAGATCTCCTCATGGATGCCATTCTCAGTATATGACAAATATGGGGACAAT
	TGCATCGCTTGTGATGTCTGTGACTATCAACGAAGATGACGATACAATGGATGGTGAT
	CAGCAGCAGATGACAAGAAAATTGTGGGGCTTAGTTGTTTGTCATCATACTAGTCCTC
	GATTTGTTCCATTTCCTTTGAGGTATGCTTGTGAATTTCTGATTCAAGTGTTTGGTGTTC
	AGATCAACAAGGAGGTAGAATTAGCTGCTCAGGTGAGGGAAAAACATATTTTACAAA
	TTCAGACTATGCTATGCGACATGCTCCTTAGAGATGCTCCAGTTGCAATTATCACACA
	ATCTCCGAATGTGATGGATCTTGTTAAGTGTGATGGAGCTGCCTTATACTTCAAGAAC
	AAAACTTGGTTGCTGGGAGTAACCCCTACAGAGGAACAAATCAGAGACATAGCCGAA
	TGGCTGCTCGAATATCATAGCGGAAACACGGGTTTAAGTACTGACAGCCTTATGGAA
	GCTGGATATCCAGGTGCTTCAGCTCTTGGTGATGCAGTTTGTGGGATGGCTGCTATTA
	GCATTACTTCGAGAGATTTTCTTTTTTGGTTTAGATCTCACACTGCCAAAGAGATCAAG
	TGGGGTGGTGCAAAACATGATCCTGATGACAAAGATGACTTAAGAAAGATGCATCCG
	AGGTCATCCTTCAAGGCCTTTCTAGAGGTGGTAAAA-----------------------------------------------
	---------------------------------------------------------------------------------------------------------------------------
	---------------------------------------------------------------------------------------------------------------------------
```


Banisteriopsis_adenopoda
TGTTGGGATGGTAGGCTCCATGCTTTTTGCATCGAACTGCTAGACCGCAATCCTTTTC
AAGTCTCGAGCATTGCTTTCGTGCTTGAGTTATCGAC-------
CGTGTTCCTGTATTTGATACCCATGTCGGAGGAACTAGGCTTCTG-TTCTCCCC----
AGTCCACTCGGTGCCTCTTGCGGGCATCGGGCGGAGCACGTAGTT-
TGATCCGTCGAGCCTAGCATGCCTT---CTCGGCTGCAACGGTCGTCGGACCAAAGC-
TTGTGTTCTCGGATGCAGAATATACGATGGGTCCAAGGATTGAATCCTTG--
CATTAGCCCTGTGTGGGAAATCGTCCCTCGATAAGAACGACATCCGTTGTGCGTCGCG
ATCCGTCCGGAGTCCTCCCGGGCCGAGGATGCTTCGTGCGTGCATCGGTGTC-
CTCGAAGGAATGCTACCTGGTTGATCCTGCCAGTAGTCATATGCTTGTCTCAAAGATT
AAGCCATGCATGCCTGCCCAGCAGAACGACCCGCGAACCG-
TCGTCAAAACGCGTCGGGGGGCGGGCCGGGCCGATAACCCCGCCCCCTTCCCGACGC
AGCCCGCCGCGCGGCCTC-
GGCCGCCCGCGGGAACAACACCCACCCCCGGCGCAGAACGCGCCAAGGAAAAC-
AAACTAAGGAGGAACATTCCC--GTGCTGTCGGACACGGCTAGCACGCGG-
ACGTTCCGCCAAAGGTCG-
AAAAAAAACGACTCTCGGCAACGGATATCTCGGCTCTCGCATCGATGAAGAACGTAG
CAAAATGCGATACTTGGTGTGAATTGCAGAATCCCGTGAACCATCGAGTCTTTGAACG
CAAGTTGCGCCCGAAGCCTTTCGGTCGAGGGCACGTCTGCCTGGGTGTCACACAACGT
CGTCCCCAAACCCGACCCC-CTCTCCCACGACGGGGGGGAGT-----
CGGGAGGGACGGAAGCTGGTCTCCCGTGGCGATCCGTCGCGGTTGGCCGAATTCAAA
GTCCTGGCACGGAAAGCCGCAACGACCGGTGGTTGGAAC-ATCCTCGATCGATGC--
GTTGCGGCCGTTCC-CGCGCGGAGAAGGGCTCCGGACCCACGGCG-
CTCCGGCGCTTTCGATCGCGACCCCAGGTCAGGCGGGAAT-------------------------------------
----

-???TAACTGCATTTTATATGTTTCGGGTTTATTTACTTACTTTTGAAGGACATTTAAATC
TTTATTTTCAAAATTACAGTGGCAAAAAAAACAGTGCATTCTATTCAATATCTCTATG
GGGTAAAGAAGGATCAAAAATACTAAAAAAAAAAAATTCCTTTATTA---------------
TCTTTATTAACAATCACTAATAATGAAAAGACTTCTTTTTTTTTGAACAAAATATACAA
AATTGATGGTACTGTAAGAAATATGACGCGTCCCTCTATTACTAT------
TAATCATTTTTTCACGAAAAATATTTTTCCCTATCCTCCTGAAGCGGATAATACTATGT
TATTTCCTATGCTTGTGTTGGTACTATTTACTTTGTTTATTGGAGCTATAGGAATTCCTT
TCAATCAAGAGGGAACTAATTTGGATATATTATCCAAACTGTTAACTCCGTCTTTAAA
TCTTTTGCATCAAAATGAAAATAATTCTTTTGATTGGTATGAATTTATAACAAATGCTA
TTTTTTCAGTCATTATAACTTTTTTTTGGAATATTTATAGCGTCTTCGTTCTATAAACCTC
TTTATTCATCGCTACAAAATTTGAACTTACTTAACTCATTTGCTA------AAAAA---------
AGGGGTCGTTGGGATAAAATAATAGGTGCGATATATGATTGGTCCTATAATCGTGGTT
ATATCGATGTTTTTTATGCAATATT?GCTTCAAGGTTTCTCTTCATGAAAAACAAGGTA
CGCCTGATATGCGATTGTCTGGCCCCTCCAGTCAAGGTAATCCAAGACGAGAGATTGC
CTCAGCCGTTAAGTCTTTGTGGGTCTACATTAAGATCTCCTCATGGTTGCCATTCTCAG
TATATGACAAATATGGGGACAATTGCATCCCTTGTGATGTCTGTGACTATCAACGAAG
ATGATGATACAATGGATGGTGACCAGCAGCAGATGACAAGAAAATTGTGGGGCTTAG
TTGTTTGTCATCATACTAGTCCTCGATTTGTTCCGTTTCCTTTGAGGTATGCTTGTGAAT

TTTTGATTCAAGTGTTTGGTGTTCAGATCAACAAGGAGGTAGAATTAGCTGCTCAGGT
GAGGGAAAAACATATTTTACAAATTCAGACTATGCTATGCGACATGCTCCTTAGAGAT
GCTCCCGTTGCAATTATCACACAATCTCCGAATGTGATGGATCTTGTTAAGTGTGATG
GAGCTGCCTTATACTTCAAGAACAAAACTTGGTTGCTGGGAGTAACGCCTACAGAGG
AACAAATCAGAGACATAGCCGAATGGCTGCTCGAATATCATAGCGGAAACACGGGTT
TAAGTACTGACAGCCTTATGGAAGCTGGATATCCAGGTGCTTCGGCTCTTGGTGATGC
AGTTTGTGGGATGGCTGCTGTTAGCATTACTTCGAGAGATTTTCTTTTTTGGTTTAGAT
CTCACACTGCTAAAGAGATAAAGTGGGGTGGTGC?????????????????????????????????????
??ATAAATTGACTTATTATACTCCTGACTA
TGAAACCAAAGATACTGATATCTTGGCAGCATTCCGAGTAACTCCTCAACCTGGAGTT
CCGCCTGAGGAAGCAGGCGCTGCGGTAGCTGCTGAATCTTCTACTGGTACATGGACA
ACTGTGTGGACCGATGGACTTACCAGTCTTGATCGTTATAAAGGGCGATGCTACGGCA
TCGAGCCCGTTGCTGGAGAAGAAAATCAATATATTGCTTATGTAGCTTACCCCTTAGA
CCTTTTTGAAGAAGGTTCTGTTACTAACATGTTTACTTCCATTGTGGGTAATGTATTTG
GGTTCAAAGCCCTACGCGCTCTCCGTCTGGAGGATTTGCGAATCCCTCCTGCTTATAC
GAAAACTTTCCAAGGCCCGCCTCATGGTATCCAAGTTGAGAGAGATAAATTGAACAA
GTATGGTCGCCCCCTATTGGGCTGTACTATTAAACCTAAATTGGGGTTATCTGCTAAG
AATTACGGTAGAGCTGTTTATGAATGTTTACGCGGTGGACTTGATTTTACCAAAGACG
ACGAGAACGTGAACTCCCAACCATTTATGCGTTGGAGAGACCGTTTCTTATTTTGTGC
CGAAGCACTTTATAAAGCACAGGCGGAAACAGGTGAAATCAAAGGGCATTATTTGAA
TGCTACTGCAGGTACATGCGAAGAAATGATCAAAAGGGCTGTATTTGCCAGAGAATT
GGGAGCTCCTATCGTAATGCATGACTACTTAACAGGGGGATTCACCGCAAATACTAGC
TTGGCTCATTATTGCCGAGATAATGGTTTACTTCTGCACATCCATCGCGCAATGCATGC
AGTTATTGATAGACAGAAGAATCATGGTATGCACTTTCGTGTACTAGCTAAAGCGTTA
CGCATGTCTGGTGGAGATCATATTCACGCGGGTACCGTAGTAGGGAAACTTGAGGGG
GAAAGAGATATCACTTTAGGCTTTGTTGATTTACTGCGTGATGATTTTGTTGAAAAAG
ATAGAAGCCGCGGGATTTATTTCACTCAAGACTGGGTCTCCCTACCAGGTGTTATACC
CGTGGCTTCGGGAGGTATTCACGTTTGGCATATGCCTGCTCTGACCGAGATCTTTGGA
GATGATTCCGTACTACAATTCGGTGGGGGAACTTTGGGGCACCCTTGGGGAAATGCAC
CAGGTGCCGTAGCTAATCGAGTAGCTCTAGAAGCATGTGTACAAGCTCGTAATGAGG
GACGTGATCTTGCTCGTGAGGGTAATGAAATTATCCGTGAGGCTAGCAAATGGAGTCC
TGAACTAGCTGCTGCTTGTGAAGTGTGGAAAGAGATTAA-
Banisteriopsis_angustifolia
TGTTGGGATGGTAGGCTCCGTGCTTTTTGCATCGAACTGCTAGACCGCAATCCTTTTC
AAGTCTCAAGCATTGCTTTTATGCTTGTGTTATCGAC-------
CGTGTTCCTGTATTTGATACCCATGTCGGCGGAACTAGGCTTCTG-TTCTCCTC----
AGTCCACTGGATGCCTCTTACGGGCATCGAGCGGAGCACGTAGTT-
TGATCTGTCGAGCCTAGCATGCCTT---CTCGGCTGCGACGGTCGTCGGGTCAAATC-
TTGTGTTCTCGGATGCAGAATATTCGATGGGTCCAAGGATTGAATCCTTG--
CATTCGCCCTGTGTGGGAAATCGTCCCTCGATAAGAACGACATCCGTCGTGCGCTGCG
ATCCGTCCGACGTCCTCTCGGGCCGAGGATTCTTCGTGCGTGCGTCGGTGTC-
CTCGAAGGAATGCTACCTGGTTGATCCTGCCAGTAGTCATATGCTTGTCTC-
AAGATTAAGCCATGCCTGCCTGCCCAGCAGAACGACCCGCGAACCG-
TTGTCAAAGCGCGTCGAGGAGCGGGTCGGGGCGAGAGTCCCGCCCTCTTCCCGACGC
AGCCCGCCGCGCGGCCTC-
GGCCGCCGTCGGGAACAACACCCACCCCCGGCGCAGAACGCGCCAAGGAAAAC-
AAACTAAGGAGGAACGCCCTC--GTGCCCCCGAACACGGCGAGCACGCGG-
ACGTTCCGCCAAAGGTAG-
AACAAAAACGACTCTCGGCAACGGATATCTCGGCTCTCGCATCGATGAAGAACGTAG
CAAAATGCGATACTTGGTGTGAATTGCAGAATCCCGTGAACCATCGAGTCTTTGAACG
CAAGTTGCGCCCGAAGCCTTTCGGTCGAGGGCACGTCTGCCTGGGTGTCACACAACGT
CGTCCACAAACCCGACCCT-CCCTCCCAAG----GGGGGAGT-----
CGGGAGGGACGGAAGCTGGTCTCCCGTGGCGATCCGCCGCGGTTGGCCGAATTCCGA
GTCCCGGCACGGAAAGCCGCAACGACCGGTGGTTGGAAC-ATCCTCGATCGATGC--
GTTGCGGCCGTTTC-CGAGCGGAGGAGGGCTCCGGACCCACGGCG-
CTCCGGCGCTTTCGATCGCGACCCCAGGTCAGGCGGGATTATTTAAATTATGTGTCTG
ATTCACTAATACCTTACCCCATCCATCTAGAAAAATTGGTTCAAATCCTTCGTTACTGG
ATGCAAGATCCCTCTTCTTTGCATTTATTACGACTCTTTCTTCACGAGTATTGGAATTG
GAATAGTTTTATTATTTCAACGAAATCCTTTTCCATTTTTTTTAATTTAAAAAGGAATC
TAAGATTATTGTTGTTCCTATATAATTCTCATGTATATGAATACGAATCCATCTTCTTT

TTTCTCCGTAACCAATCCTTTTATTTCCAATCAACATTTTCGGGGGTGCTTCTTGAACG
AATATATTTCTATGGAAAAATACAAGATTTTGTAGAACTTTTTACTAATG------
ATTTTAAGGCTAACCTACGGTTGTTCAAGGATCCTTTCATGCATTATGTTAGATATCAA
GGAAAAGGCATTCTGGCTTCAAAAAGTACACTTCTTCTGATGAAAAAATGGAAATATT
TCTTTGTCAATTTATGTCAATTTCATTTTTATGTGTGGGTTCATCCAGAACCGATCTAT
ATGAAGTCATTATCCAACCATTCTCTTGACTTTTTGGGTTATCTTTCAAATATACGAAA
AAATCTTTCAGTGGTACGGAGTCAAATGCTAGAAAATTCATTTCTAATAGATAATATT
ATGAAGAAACTCGATTCAAGAGTTCCAATTATTCCTTTGATTGGATCATTGTCAAAAA
CAAAAATTTGTAACGCAGTAGGGCATCCCATTAGTAAACCGAGCTGGGCGGATTCAC
CCGATTCTGACATTATCGACCAATTTTTGCGTATATCCAGAAATCTTTCTCATTACTAT
AGCGGATCCTCAAAAAAAAAGGATTTGTATCGAGTAAAATATATACTTCGACTTTCTT
GTGTTAAAACTTTGGCTCGTAAACACAAAAGTACCGTCCGGGCTTTTTTGAAAAAACT
AGGTTCAGAATTATTGGAAGAATTTTTTACGGAGGAAAAATCAATTCTTGATTTGGTC
TTCCCCACGGCTTATTCTATTTCGCGAAGGTTATACAGAGGGCGGGTTTGGTATTTGG
ATATTATTTGTATCAATGATCTAGCCAATCATGAATAAGATTAACTGCATTTTATATGT
TTCGGGTTTATTTACTTACTTTTGAAGGACATTTAAATCTTTATTTTCAAAATTACAGT
GGTAAAAAAAACAGTGCATTCTATTCAATATCTCTATGGGGTAAAGAAGGATCAAAA
ATACTAAAAAAAAAAAATTCCTTTATTA--------------
TCTTTATTAACAATCACTAATAATGAAAAGACTTCTTTTTTTTTGAACAAAATATACAA
AATTGATGATAATGTAAGAAATATGACGCGTCCCTCTATTACTAT------
TAATCATTTTTTCACGAAAAATGTTTTTCCCTATCCCGCTGAAGCGGATAATGCTATGT
TATTTCCTATGCTTGTGTTGGTACTATTTACTTTGTTTATTGGAGCTATAGGAATTCCTT
TCAATCAAGAGGGAACTAATTTGGATATATTATCCAAACTGTTAACTCCGTCTTTAAA
TCTTTTGCATCAAAATGAAAATAATTCTTTTGATTGGTATGAATTTATAACAAATGCTA
TTTTTTCAGTCATTATAACTTTTTTTGGAATATTTATAGCGTCTTCCTTCTATAAACCTC
TTTATTCATCGCTACAAAATTTGAACTTACTTAACTCATTTGCTA------AAAAA---------
AGGGGTCGTTGGGATAAAATAATAGGTGCGATATATGATTGGTCCTATAATCGTGGTT
ATGTCGATGTTTTTTATGCAATATTGGCTTCAAGGTTTCTCTTCATGAAAAACAAGGTA
CGAATGATATGCGATTGTCTGGCCCCTCCAGTCAAGGTAATCCAAGACGAGAGATTGC
CTCAGCCCTTAAGTCTTTGTGGGTCTACATTAAGATCTCCTCATGGTTGCCATTCTCAG
TATATGACAAATATGGGGACAATTGCATCGCTTGTGATGTCTGTGACTATCAACGAAG
ATGATGATACAATGGATGGTGATCAGCAGCAGATGACAAGAAAATTGTGGGGCTTAG
TTGTTTGTCATCACACTAGTCCTCGATTTGTTCCGTTTCCTTTGAGGTATGCTTGTGAGT
TTTTGATTCAAGTGTTTGGTGTTCAGATCAACAAGGAGGTAGAATTAGCTGCTCAGGT
GAGGGAAAAACATATTTTACAAATTCAGACTATGCTATGCGACATGCTCCTTAGAGAT
GCTCCCGTTGCAATTATCACACAATCTCCGAATGTGATGGATCTGGTTAAGTGTGATG
GAGCTGCCTTATACTTCAAGAACAAAACTTGGTTGCTGGGAGTAACGCCTACAGAGG
AACAAATCAGAGACATAGCCGAATGGCTGCTCGAATATCATAGCGGAAACACGGGTT
TAAGTACTGACAGCCTTATGGAAGCTGGATATCCAGGTGCTTCCGCTCTTGGTGATGC
AGTTTGTGGGATGGCTGCTGTTAGCATTACTTCGAGAGATTTTCTTTTTTGGTTTAGAT
CTCACACTGCTAAAGAGATCAAGTGGGGTGGTGCAAAACATGATCCTGATGACAAAG
ATGACTTAAGAAAGATGCATCCGAGGTCATCCTTCAAGGCCTT????????????????---------
----

Banisteriopsis_anisandra --
ATTCCTTTTCAAGCCTCAAGCATTGCTTTTATGCTAAGTTTGTTGAC-------
GGTGTTCCTGTATTTGATACCCATTTCGGAGGAACTAAGCTTCTG-TTCTCCCC----
AGTCCACTTGATGCTTCTTAAGGGCATCGGGCGGAGCACGTAGTT-
TGATCTGTCGAACCTAGCATGCCCT---TTCGGTTGCAACGGTTGTCGGATCAAAGC-
TTGTGTTCTCGGATGCAGAATATTCGATGGGGCCAAGGATTGAATCCTTG--

CATTCGCCCCGTGTGGGAAATCGTCCCTCGATAAAAACGACATCCGTCGTGCGCTGCG
ATCCATCCGATGTCCTCGCGGGCTGAGGATTCTTCGTGCGTGCATCGGTGTC-
CTCGAAGGAATGCTACCTGGTTGATCCTGCCAGTAGTCATATGCTTGTCTCAAAGATT
AAGCCATGCATGCCGGCCCAGCAGAACGACCCGCGAACCG-
TTGTGGAAACGCGTCGAGGAGCGGGCCGGGGCGACAGTCCCGCCCTCTTCACGACGC
AGCCCGCTGCGTGGCCTC-
GGCCGCCCGCGGGAACATCACCCACCCCCGGCGCAGAACGCGCCAAGGAAAAC-
AAACTGAGGAGGAATCTTCTC--GTGCTCCCGAACACGGTGAGCACGCGA-
ACGTTCCGCCCAAGGTCG-
AACAAAAACGACTSTCGGCAACGGATATCTCGGCTCTCGCATCGATGAAGAACGTAG
CAAAATGCGATACTTGGTGAGAATTGCAAAATCCCGTGAACCMTCGAGTCTTTGAACS
CAAGTTGTGCCCGAAGCCTTTTGGTCGAGGGCACGTCTKCCTGGG-
GTCACRCAACGTCGTCCCCAAACCCAACCCT-CTCTCCCACG----GGGGGCGT-----
CGGGAGGGACGGAAGCTGGTCTCCCGTGGCGATCTGCCGCGGTTGGCCGAATTACGA
GTCCGGGCACGGAAAGCCGCAACAACCGGTGGTTGGAAC-ATCCTCGATCGATGC--
GTTGCGGCCGTTTT-CTCGAGGGGACGGGCTCTAGACCCKCAGCG-CTCCGCCGC-
TTCGATCGCGACCCCCGGGCAGGCGGGATTATTTAAATTATGTGTCTGATTCACTAAT
ACCTTACCCCATCCATCTAGAAAAATTGGTTCAAATCCTTCGTTACTGGATGCAAGAT
CCCTCTTTTTTGCATTTATTACGACTCTTTCTTCACGAGTATTGGAATTGGAATAGTTTT
CTTATTTCAACGAAATCCTTTTCCATTTTTTTTAATTTAAAAAGGAATCTAAGATTATT
GTTGTTCCTATATAATTCTCATGTATATGAATACGAATCCATCTTCTTTTTTCTCCGTAA
CCAATCCTTTTATTTCCAATCAACATTTTCGGGGGTGCTTCTTGAACGAATATATTTCT
ATGGAAAAATACAAGATTTTGTAGAACTTTTTACTAATG------
ATTTTAAGGCTAACCTACGGTTGTTCAAGGATCCTTTCATGCATTATGTTAGATATCAA
GGAAAAGGCATTCTGGCTTCAAAAAGTACACTTCTTCTGATGAAAAAATGGAAATATT
TCTTTGTTAATTTATGTCAATTTCATTTTTATGTGTGGGTTCATCCAGAACCGATCTAT
ATGAAGTCATTATCCAACCATTCTCTTGACTTTTTGGGTTATCTTTCAAGTATACGAAA
AAATCTTTCAGTGGTACGGAGTCAAATGCTAGACAATTCATTTCTAATAGATAATATT
ATGAAGAAACTCGATTCAAGAGTTCCAATTATTCCTTTGATTGGATCATTGTCAAAAA
CAAAATTTTGTAACGCAGTAGGGCATCCCATTAGTAAACCGAGCTGGGCGGATTCACC
CGATTCTGATATTATCGACCAATTTTTGCGTATATCCAGAAATCTTTCTCATTACTATA
GCGGATCCTCAAAAAAAAAGAGTTTGTATCGAGTAAAATATATACTTCGACTTTCTTG
TGTTAAAACTTTGGCTCGTAAACACAAAAGTACCGTCCGGGCTTTTTTGAAAAAACTA
GGTTCAGAATTATTGGAAGAATTTTTTACGGAGGAAAAATCGATTCTTGATTTGGTCT
TCCCCACGGCTTATTCTATTTCGCGAAGGTTATACAGAGGGCGGGTTTGGTATTTGGA
TATTATTTGTATCAAT---
AATCTTTATCTTCAAAATTACAGTGGCAAAAAAAACAGTGCATTCTATTCAATATCTC
TATGGGGTAAAGAAGGATCAAAAATACTAAAAAAAAAAAATTCCTTTATTA--------------
TCTTTATTAACAATCACTAATAATGAAAAGACTTCTTTTTTTTTGAACAAAATAGACCA
AATTGATGGTAATGTAAGAAATATGACGCGTCCCTCTATTACTAT------
TAATCATTTTTTCACGAAAAATATTTTTCCCTATCCCCCTGAAGCGGATAATACTATGT
TATTTCCTATGCTTGTGTTGGTACTATTTACTTTGTTTATTGGAGTTATAGGAATTCCTT
TCAATCAAGAGGGAACTAATTTGGATATATTATCCAAACTGTTAACTCCGTCTTTAAA
TCTTTTGCATCAAAATGAAAATAATTCTTTTGATTGGTATGAATTTATAACAAATGCTA
TTTTTTCAGTCATTATAACTTTTTTTGGAATATTTATAGCGTCTTCCTTCTATAAACCTC
TTTATTCATCGCTACAAAATTTGAACTTACTTAACTCATTTGCTAAAAATCAAAAA------
---
AGGGGTCGTTGGGACAAAATAATAGGTGCGATATATGATTGGTCCTATAATCGTGGTT
ATATCGATGTTTTTTATGCAATATT---

ATAAATTGACTTATTATACTCCTGACTATGAAACCAAAGATACTGATATCTTGGCAGC
ATTCCGAGTAACTCCTCAACCTGGAGTTCCGCCTGAGGAAGCAGGCGCTGCGGTAGCT
GCTGAATCTTCTACTGGTACATGGACAACTGTGTGGACCGATGGACTTACCAGTCTTG
ATCGTTATAAAGGGCGATGCTACGGCATCGAGCCCGTTGCTGGAGAAGAAAATCAAT

ATATTGCTTATGTAGCTTACCCCTTAGACCTTTTTGAAGAAGGTTCTGTTACTAATATG
TTTACTTCCATTGTGGGTAATGTATTTGGGTTCAAAGCCCTACGCGCTCTCCGTCTGGA
GGATTTGCGAATCCCCCCTGCTTATACAAAAACTTTCCAAGGCCCGCCTCATGGTATC
CAAGTTGAGAGAGATAAATTGAACAAGTATGGTCGCCCCCTATTGGGCTGTACTATTA
AACCTAAATTGGGGTTATCTGCTAAGAATTACGGTAGAGCTGTTTATGAATGTCTACG
CGGTGGACTTGATTTTACCAAAGACGACGAGAACGTGAACTCCCAACCATTTATGCGT
TGGAGAGACCGTTTCTTATTTTGTGCCGAAGCACTTTATAAAGCACAGGCGGAAACAG
GTGAAATCAAAGGGCATTATTTGAATGCTACTGCAGGTACATGCGAAGAAATGATCA
AAAGGGCTGTATTTGCCAGAGAATTGGGAGCTCCTATCGTAATGCATGACTACTTAAC
AGGGGGATTCACCGCAAATACTAGCTTGGCTCATTATTGCCGAGATAATGGTTTACTT
CTGCACATCCATCGCGCAATGCATGCAGTTATTGATAGACAGAAGAATCATGGTATGC
ACTTTCGTGTACTAGCTAAAGCGTTACGCATGTCTGGTGGAGATCATATTCACGCGGG
TACCGTAGTAGGGAAACTTGAGGGGGAAAGAGATATCACTTTAGGCTTTGTTGATTTA
CTGCGTGATGATTTTGTTGAAAAAGATAGAAGCCGCGGGATTTATTTCACTCAAGACT
GGGTCTCCCTACCAGGTGTTATACCCGTGGCTTCGGGAGGTATTCACGTTTGGCATAT
GCCTGCTCTGACCGAGATCTTTGGAGATGATTCCGTACTACAATTCGGTGGGGGAACT
TTGGGGCACCCTTGGGGAAATGCACCAGGTGCCGTAGCTAATCGAGTAGCTCTAGAA
GCATGTGTACAAGCTCGTAATGAGGGACGTGATCTTGCTCGTGAGGGTAATGAAATTA
TCCGTGAGGCTAGCAAATGGAGTCCTGAACTAGCTGCTGCTTGTGAAGTGTGGAAAG
AGATTAAA
Banisteriopsis_argyrophylla
AGTTGGGATGGTAGGCTCCGTGCTTTTTGCATCGAACTACTAGACCGCAATCCTTTT
CAAGCCTCAAGCATTGCTTT-ATGCTTGTGTTATCGAC-------
CGTGTTCCTGTATTTGATACCCATGTCGGCGGAACTAGGCTTCTG-TTCTCCCC----
ATTCCACTTGGTGCCTCTTGCGGGCATCGAGCGGAGCACGTGGTT-
TGATTTGTCGATCCTAGCATGCCTG---TTCGGTTGCAAAGGTCGACGGATCAAAGC-
TTGTGTTCTCGGATGCAGAATGTTCGATGGGTGCAGGGATTGAATCCTTG--
CGTTTGCCCAGTGTGGGAAATCGTCCCTCGATAAGAACGACGTCCGTTGCGCGCTGCG
ATCCATCCGATGTCCTCTCGGACCGAGGATGCCTCGCGCGTGCATCGGTGTC-
CTCGAAGGAATGCTACCTGGTTGATCCTGCCAGTAGTCATATGCTTGTCTCAAAAATT
AAGCCATGCATGCCTGCCCAGCAGAACGACCCGCGAACCG-
TTGTCAAAGCGCGTCGAGGAGCGGGCCGGGCCGACGGTCCCGTCCCCTTCCCGACGC
AGCCCGTCGTGTGGCCTC-
GGCCGCCTGCGGGAACAACAACCACCCCCGGCGCAGAACGCGCCAAGGAAAAC-
AAACTAAGGAGGAATCTTCTC--GTGCTCCCGAACTCGGCGAGCAGGGGA-
ACGTTCCGCCAAAGGTCG-
AACAAAAACGACTCTCGGCAACGGATATCTCGGCTCTCGCATCGATGAAGAACGTAG
CAAAATGCGATACTTGGTGTGAATTGCAGAATCCCGTGAACCATCGAGTCTTTGAACG
CAAGTTGCGCCCGAAGCCTTTCGGCCGAGGGCACGTCTGCCTGGGTGTCACACAACGT
CGTCCCCAAACCCGACCCT-CTCTCCCACG--GGAAGGGAGT-----
CTGGAGGGACGGAAGCTGGTCTCCCGTGGCGTTCCGCCGCGGTTGGCCGAATTCCGA
GTCCGGGCGAGGAAAGCCGCAACAACCGGTGGTTGGAACGATCCTCGATCGATGC--
GTTGCGGCCGTTTC-TTCGCGGAGACGGGCTCCAGACCCACGGCG-
CTCCGGCGCCTTCGATCGCGACCCCAGGTCAGGCGGGATC???TAAATTATGTGTCTGA
TTCACTAATACCTTACCCCATCCATCTAGAAAAATTGGTTCAAATCCTTCGTTACTGGA
TGCAAGATCCCTCTTCTTTGCATTTATTACGACTCTTTCTTCACGAGTATTGGAATTGG
AATAGTTTTATTATTTCAACGAAATCCTTTTCCATTTTTTTTAATTTAAAAAGGAATCT
AAGATTATTGTTGTTCCTATATAATTCTCATGTATATGAATACGAATCCATCTTCTTTT
TTCTCCGTAACCAATCCTTTTATTTCCAATCAACATTTTTGGGGGTGCTTCTTGAACGA
ATATATTTCTATGGAAAAATACAAGATTTTGTAGAACTTTTTACTAATG------
ATTTTAAGGCTAACCTACGGTTGTTCAAGGATCCTTTCATGCATTATGTTAGATATCAA
GGAAAAGGCATTCTGGCTTCAAAAAGTACACTTCTTCTGATGAAAAAATGGAAATATT
TCTTTGTCAATTTATGTCAATTTCATTTTTATGTGTGGGTTCATCCAGAACCGATCTAT
ATGAAGTCATTATCCAACCATTCTCTTGACTTTTTGGGTTATCTTTCAAATATACGAAA
AAATCTTTCAGTGGTACGGAGTCAAATGCTAGAAAATTCATTTCTAATAGATAATATT
ATGAAGAAACTCGATTCAAGAGTTCCAATTATTCCTTTGATTGGATCATTGTCAAAAA
CAAAATTTTGTAACGCAGTAGGGCATCCCATTAGTAAACCGAGCTGGGCGGATTCACC
CGATTCTGACATTATCGACCAATTTTTGCGTATATCCAGAAATCTTTCTCATTACTATA
GCGGATCCTCAAAAAAAAAGAATTTGTATCGAGTAAAATATATACTTCGACTTTCTTG
TGTTAAAACTTTGGCTCGTAAACACAAAAGTACCGTCCGGGCTTTTTTGAAAAAACTA

GGTTCAGAATTATTGGAAGAATTTTTTACGGAGGAAAAATCAATTCTTGATTTGGTCT
TCCCCACGGCTTATTCTATTTCGCGAAGGTTATACAGAGGGCGGGTTTGGTATTTGGA
TATTATTTGTATCAATGATCTAGCCAATCATGAATAAGATTAACTGCATTTTATATGTT
TCGGGGTTATTTACTTACTTTTGAAGGACATTTAAACCTTTATTTTCAAAATTACAGTG
GCAAAAAAAAACAGTGCATTCTATTCAATATCTCTATGGGGTAAAGAAGGATCAAAAA
TACTAAAAAAAAAAAATTCCTTTATTA---------------
TCTTTATTAACAATCACTAATAATGAAAAGACTTCTTTTTTTTTGAACAAAATATACAA
AATTGATGGTAATGTAAGAAATATGACGCGTCCCTCTATTACTAT------
TAATCATTTTTTCACGAAAAATATTTTTCCCTATCCCCCTGAAGCGGATAATACTATGT
TATTTCCTATGCTTGTATTGGTACTATTTACTTTGTTTATTGGAGCTATAGGAATTCCTT
TCAATCAAGAGGGAACTAATTTGGATATATTATCCAAACTGTTAACTCCGTCTTTAAA
TCTTTTGCATCAAAATGAAAATAATTCTTTTGATTGGTATGAATTTATAACAAATGCTA
TTTTTTCAGTCATTATAACTTTTTTTGGAATATTTATAGCGTCTTCCTTCTATAAACCTC
TTTATTCATCTCTACAAAATTTGAACTTACTTAACTCATTTGCTA------AAAAA---------
AGGGGTCGTTGGGATAAAATAATAGGTGCGATATATGATTGGTCCTATAATCGTGGTT
ATATCGATGTTTTTTATGCAATATT????TCAAGGTTTCTTTTCATGAAAAACAAGGTAC
GCATGATATGCGATTGTCTGGCCCCTCCAGTCAAGGTAATCCAAGACAAGAGATTGCC
TCAGCCGTTAAGTCTTTGTGGGTCTACATTAAGATCTCCTCATGGTTGCCATTCTCAGT
ATATGACAAATATGGGGACAATTGCATCGCTTGTGATGTCTGTGACTATCAACGAAGA
TGACGATACGATGGATGGTGATCAGCAGCAGATGACAAGAAAATTGTGGGGCTTAGT
TGTTTGTCATCATACTAGTCCTCGATTTGTTCCATTTCCTTTGAGGTATGCTTGTGAATT
TCTGATTCAAGTGTTTGGTGTTCAGATCAACAAGGAGGTAGAATTAGCTGCTCAGGTG
AGGGAAAAACATATTTTACAAATTCAGACTATGCTATGCGACATGCTCCTTAGAGATG
CTCCAGTTGCAATTATCACACAATCTCCGAATGTGATGGATCTTGTTAAGTGTGATGG
AGCTGCCTTATACTTCAAGAACAAAACTTGGTTGCTGGGACTAACCCCTACAGAGGAA
CAAATCAGAGACATAGCCGAATGGCTGCTCGAATATCATAGCGGAAACACGGGTTTA
AGTACTGACAGCCTTATGGAAGCTGGATATCCAGGTGCTTCAGCTCTTGGTGATGCAG
TTTGTGGGACGGCTGCTATTAGCATTACTTCGAGAGATTTTCTTTTTTGGTTTAGATCT
CACACTGCCAAAGAGATCAAGTGGGGTGGTGCAAAACATGATCCTGATGACAAAGAT
GACTTAAGAAAGATGCATCCGAGGTCATCCTTCAAGGC???????????????????ATAAATT
GACTTATTATACTCCTGACTATGAAACCAAAGATACTGATATCTTGGCAGCATTCCGA
GTAACTCCTCAACCTGGAGTTCCGCCTGAGGAAGCAGGCGCTGCGGTAGCTGCTGAAT
CTTCTACTGGTACATGGACAACTGTGTGGACCGATGGACTTACCAGTCTTGATCGTTA
TAAAGGGCGATGCTACGGCATCGAGCCCGTTGCTGGAGAAGAAAATCAATATATTGC
TTATGTAGCTTACCCCTTAGACCTTTTTGAAGAAGGTTCTGTTACTAACATGTTTACTT
CCATTGTGGGTAATGTATTTGGGTTCAAAGCCCTACGCGCTCTCCGTCTGGAGGATTT
GCGAATCCCTCCTGCTTATACGAAAACTTTCCAAGGCCCGCCTCATGGTATCCAAGTT
GAGAGAGATAAATTGAACAAGTATGGTCGCCCCCTATTGGGCTGTACTATTAAACCTA
AATTGGGGTTATCTGCTAAGAATTACGGTAGAGCTGTTTATGAATGTTTACGCGGTGG
ACTTGATTTTACCAAAGACGACGAGAACGTGAACTCCCAACCATTTATGCGTTGGAGA
GACCGTTTCTTATTTTGTGCCGAAGCACTTTATAAAGCACAGGCGGAAACAGGTGAAA
TCAAAGGGCATTATTTGAATGCTACTGCAGGTACATGCGAAGAAATGATCAAAAGGG
CTGTATTTGCCAGAGAATTGGGAGCTCCTATCGTAATGCATGACTACTTAACAGGGGG
ATTCACCGCAAATACTAGCTTGGCTCATTATTGCCGAGATAATGGTTTACTTCTGCAC
ATCCATCGCGCAATGCATGCAGTTATTGATAGACAGAAGAATCATGGTATGCACTTTC
GTGTACTAGCTAAAGCGTTACGCATGTCTGGTGGAGATCATATTCACGCGGGTACCGT
AGTAGGGAAACTTGAGGGGGAAAGAGATATCACTTTAGGCTTTGTTGATTTACTGCGT
GATGATTTTGTTGAAAAAGATAGAAGCCGCGGGATTTATTTCACTCAAGACTGGGTCT
CCCTACCAGGTGTTATACCCGTGGCTTCGGGAGGTATTCACGTTTGGCATATGCCTGC
TCTGACCGAGATCTTTGGAGATGATTCCGTACTACAATTCGGTGGGGGAACTTTGGGG
CACCCTTGGGGAAATGCACCAGGTGCCGTAGCTAATCGAGTAGCTCTAGAAGCATGT
GTACAAGCTCGTAATGAGGGACGTGATCTTGCTCGTGAGGGTAATGAAATTATCCGTG
AGGCTAGCAAATGGAGTCCTGAACTAGCTGCTGCTTGTGAAGTGTGGAAAGAGATTA
A-
Banisteriopsis_basifixa ---

ATTTAAATTATGTGTCTGATTCACTAATACCTTACCCCATCCATCTAGAAAAATTGGTT
CAAATCCTTCGTTACTGGATGCAAGATCCCTCTTCTTTGCATTTATTACGACTCTTTCTT
CACGAGTATTGGAATTGGAATAGTTTTCTTATTTCAACGAAATCCTTTTCCATTTTTTT
TAATTTAAAAAGGAATCTAAGATTATTGTTGTTCCTATATAATTCTCATGTATATGAAT
ACGAATCCATCTTCTTTTTTCTCCGTAACCAATCCTTTTATTTCCAATCAACATTTTCGG
GGGTGCTTCTTGAACGAATATATTTCTATGGAAAAATACAAGATTTTGTAGACCTTTTT
ACTAATGATTTTCATTTTAAGGCTAACCTACGGTTGTTCAAGGATCCTTTCATGCATTA
TGTTAGATATCAAGGAAAAGGCATTCTGGCTTCAAAAAGTACACTTCTTCTGATGAAA
AAATGGAAATATTTCTTTGTCAATTTATGTCAATTTCATTTTTATGTGTGGGTTCATCC
AGAACCGATCCATATGAAGTCATTATCCAACCATTCTCTTGACTTTTTGGGTTATCTTT
CAAGTATACGAAAAAATCTTTCAGTGGTACGGAGTCAAATGCTAGAAAATTCATTTCT
AATAGATAATATTATGAAGAAACTCGATTCAAGAGTTCCAATTATTCCTTTGATTGGA
TCATTGTCAAAAACAAGATTTTGTAACGCAGTGGGGCATCCCATTAGTAAACCGAGCT
GGGCGGATTCACCCGATTCTGATATTATCGACCAATTTTTGCGTATATCCAGAAATCTT
TCTCATTACTATAGCGGATCCTCAAAAAAAAAGAGTTTGTATCGAGTAAAATATATAC
TTCGACTTTCTTGTGTTAAAACTTTGGCTCGTAAACACAAAAGTACCGTCCGGGCTTTT
TTGAAAAAACTAGGTTCAGAATTATTGGAAGAATTTTTTACGGAGGAAAAATCGATTC
TTGATTTGGTCTTCCCCACGGCTTATTCTATTTCGCGAAGGTTATACAGAGGGCGGGTT
TGGTATTTGGATATTATTTGTATCAATGATCTAGCCAATCATGAA---
GATTAACTGCATTTTATATGTTTCGGGTTTATTTACTTACTTTTGAAGGACATTTAAAT
CTTTATTTTCAAAATTACAGTGGCAAAAAAAACAGTGCATTCTATTCAATATCTCTAT
GGGGTAAAGAAGGATCAAAAATACTCAAAAAAAAAATTCCTTTATTA---------------
TCTTTATTAACAATCACTAATAATGAAAAAGCTTCTTTTTTTTTGAACAAAATATACCA
AATTGATGGTAATGTAAGAAATATGACGCTTCCCTCTATTACTAT------
TAATCATTTTTTCACGAAAACTATTTTTCCCTATCCCCCTGAAGCGGATAATACTATGT
TATTTCCTATGCTTGTGTTGGTACTATTTACTTTGTTTATTGGAGCTATAGGAATTCCTT
TCAATCAAGAGGGAACTCATTTGGATATATTATCTAAACTGTTAACTCCGTCTTTAAA
TCTTTTGCATCAAAATGAAAATAATTCTTTTGATTGGTATGAATTTATAACAAATGCTA
TTTTTTCAGTCATTATAACTTTTTTTGGAATATTTATAGCGTCTTCCTTCTATAAACCTC
TTTATTCATCGCTACAAAATTTGAACTTACTTAACTCATTTGCTA------AAAAA---------
AGGGGTCATTGGGACAAAATAATAGGTGCGATATATGATTGGTCCTATAATCGTGGTT
ATATCGATGTTTTTTATGCAATATT--

Banisteriopsis_caapi
TGTTGGGATGGTAGGCTCCATGCTTTTTGCATCGAACTGCTAGCCCGCAATCCTTTTC
AAGTCTCGAGCATTGTTTTTATGCTCGATTTATCGAC-------
CGTGTTCCTGTATTTGATACCCATGTTGGAGGAACTAGGCTTCTG-TTCTCCCC----
AGTCCACTCGATGCCCCTTAGGGGCATCGAGCGGAGCACGTAGTT-
TGATCTGTCGAGCCTAGCATGCCTT---CTCGGCTGCAACGGTCGTCGGATCACATC-

TCGTGTTCTCGGATGCAGAATACTCGATGGGTCCAAGGATTGAATCCTTG--
CATTAGCCCCGTGTGGGAAATCGTCCCTCGATAAGAACGACGTCCGCTGTGCGTCGCG
AACCGTCCGATGTCCTCTCGGGACGAGGACGCTTCGTGCGTGCATCGGTGTC-
CTCGAAGGAATGCTACCTGGTTGATCCTGCCAGTAGTCATATGCTTGTCTCAAAGATT
AAGCCATGCATG---
GCCCTCTCCCCGACGCAGCCCGTCGAGCGGCCTC-
GGCCGTCGGCGGGAACAAAACCCACCCCCGGCGCAGAACGCGTCAAGGAAAAC-
AAACCGAGGAGGAACGTCCCC--GTGCCACCGGACACGGCAAGCACGCGG-
ACGTCCCGCCAAACGTAGAAAAAAAAACGACTCTCGGCAACGGATATCTCGGCTCTC
GCATCGATGAAGAACGTAGCAAAATGCGATACTTGGTGTGAATTGCAGAATCCCGTG
AACCATCGAGTCTTTGAACGCAAGTTGCGCCCGAAGCCATTCGGTCGAGGGCACGTCT
GCCTGGGTGTCACACAACGTCGTCCCCAAACCCGACCCC-CTCTAACACG----
GGGAGAGT-----
CGGGAGGGACGGAAACTGGTCTCCCGTGGCGATCCGCCGCGGTTGGCCGAATTCCGA
GTCCCGGCGAGGAAAGCCGCAACGACCGGTGGTTGGAAC-GTCCTCGATCGACGC--
GTTGCGGCCGTTTTCCGCGCGGAGACGGGCTCCGGACCCACGGC----------------------------
----
ATTTAAATTATGTGTCTGATTCACTAATACCTTACCCCATCCATCTAGAAAAATTGGTT
CAAATCCTTCGTTACTGGATGCAAGATCCCTCTTCTTTGCATTTATTACGACTCTTTCTT
CACGAGTATTGGAATTGGAATAGTTTTCTTATTTCAACGAAATCCTTTTCCATTTTTTT
GAATTTAAAAAGGAATCTAAGATTATTGTTGTTCCTATATAATTCTCATGTATATGAAT
ATGAATCCATCTTCTTTTTTCTCCGTAACCAATCCTTTTATTTCCAATCAACATTTTCGG
GGGTGTTTCTTGAACGAATATATTTCTATGGAAAAATACAAGATTTTGTAGAACTTTTT
ACTAATG------
ATTTTAAGGCTAACCTACGGTTGTTCAAGGATCCTTTCATGCATTATGTTAGATATCAA
GGAAAAGGCATTCTGGCTTCAAAAAGTACACTTCTTCTGATGAAAAAATGGAAATATT
TCTTTGTCAATTTATGTCAATTTCATTTTTATGTGTGGGTTCATCCAGAACCGATCTAT
ATGAAGTCATTATCCAACCATTCTCTTGACTTTTTGGGTTATCTTTCAAATATACGAAA
AAATCTTTCAGTGGTACGGAGTCAAATGCTAGAAAATTCATTTCTAATAGATAATATT
ATGAAGAAACTCGATTCAAGAGTTCCAATTATTCCTTTGATTGGATCATTGTCAAAAA
CAAAATTTTGTAACGCAGTAGGGCATCCCATTAGTAAACCGAGCTGGGCGGATTTACC
CGATTCTGACATTATCGACCAATTTTTGCGTATATCCAGAAATCTTTCTCATTACTATA
GCGGATCCTCAAAAAAAAAGAATTTGTATCGAGTAAAATATATACTTCGACTTTCTTG
TGTTAAAACTTTGGCTCGTAAACACAAAAGTACCGTCCGGGCTTTTTTGAAAAAACTA
GGTTCAGAATTATTGGAAGAATTTTTTACGGAGGAAAAATCAATTCTTGATTTGGTCT
TCCCCACGGCTTATTCTATTTCGCGAAGGTTATACAGAGGGCGGGTTTGGTATTTGGA
TATTATTTGTATCAATGATCTAGCCAATCATGAATAAGATTAACTGCATTTTATATGTT
TCGGGTTTATTTACTTACTTTTGAAGGCCATTTAAATCTTTATTTTCAAAATTACAGTG
GCAAAAAAAACAGTGCATTCTATTCAATATCTCTATGGGGTAAAGAAGGATCAAAAA
TACTAAAAAAAAAAAATTCCTTTATTA---------------
TCTTTATTAACAATCACTAATAATGAAAAGACTTCTTTTTTTTTGAACAAAATATACAA
AATTGATGGTACTGTAAGAAATATGACGCGTCCCTCTATTACTAT------
TAATCATTTTTTCACGAAAAATATTTTTCCCTATCCTCCTGAAGCGGATAATACTATGT
TATTTCCTATGCTTGTGTTGGTACTATTTACTTTGTTTATTGGAGCTATAGGAATTCCTT
TCAATCAAGAGGGAACTAATTTGGATATATTATCCAAACTGTTAACTCCGTCTTTAAA
TCTTTTGCATCAAAATGAAAATAATTCTTTTGATTGGTATGAATTTATAACAAATGCTA
TTTTTTCAGTCATTATAACTTTTTTTGGAATATTTATAGCGTCTTCCTTCTATAAACCTC
TTTATTCATCGCTACAAAATTTGAACTTACTTAACTCATTTGCTA------AAAAA---------
AGGGGTCGTTGGGATAAAATAATAGGTGCGATATATGATTGGTCCTATAATCGTGGTT
ATATCGATGTTTTTTATGCAATATTAGCTTCAAGGTTTCTCTTCATGAAAAACAAGGTA
CGCATGATATGCGATTGTCTGGCGCCTCCAGTCAAGGTAATCCAAGACGAGAGATTGC
CTCAGCCGTTAAGTCTTTGTGGGTCTACATTAAGATCTCCTCATGGTTGCCATTCTCAG
TATATGACAAATATGGGGACAATTGCATCCCTTGTGATGTCTGTGACTATCAACGAAG
ATGATGATACAATGGATGGTGACCAGCAGCAGATGACAAGAAAATTGTGGGGCTTAG
TTGTTTGTCATCATACTAGTCCTCGATTTGTTCCGTTTCCTTTGAGGTATGCTTGTGAAT
TTTTGATTCAAGTGTTTGGTGTTCAGATCAACAAGGAGGTAGAATTAGCTGCTCAGGT
GAGGGAAAAACATATTTTACAAATTCAGACTATGCTATGCGACATGCTCCTTAGAGAT
GCTCCCGTTGCAATTATCACACAATCTCCGAATGTGATGGATCTTGTTAAGTGTGATG
GAGCTGCCTTATACTTCAAGAACAAAACTTGGTTGCTGGGAGTAACGCCTACAGAGG
AACAAATCAGAGACATAGTCGAATGGCTGCTTGAATATCATAGCGGAAACACGGGTT

TAAGTACTGACAGCCTTATGGAAGCTGGATATCCAGGTGCTTCGGCTCTTGGTGATGC
AGTTTGTGGGATGGCTGCTGTTAGCATTACTTCGAGAGATTTTCTTTTTTGGTTTAGAT
CTCACACTGCTAAAGAGATAAAGTGGGGTGGTGCAAAACATGATCCTGATGACAAAG
ATGACTTAAGAAAGATGCATCCGAGGTCATCCTTCAAGGCCTTCCTAGAGGTGGTAAA
AATAAATTGACTTATTATACTCCTGACTATGAAACCAAAGATACTGATATCTTGGCAG
CATTCCGAGTAACTCCTCAACCTGGAGTTCCGCCTGAGGAAGCAGGCGCTGCGGTAGC
TGCTGAATCTTCTACTGGTACATGGACAACTGTGTGGACCGATGGACTTACCAGTCTT
GATCGTTATAAAGGGCGATGCTACGGCATCGAGCCCGTTGCTGGAGAAGAAAATCAA
TATATTGCTTATGTAGCTTACCCCTTAGACCTTTTTGAAGAAGGTTCTGTTACTAACAT
GTTTACTTCCATTGTGGGTAATGTATTTGGGTTCAAAGCCCTACGCGCTCTCCGTCTGG
AGGATTTGCGAATCCCTCCTGCTTATACGAAAACTTTCCAAGGCCCGCCTCATGGTAT
CCAAGTTGAGAGAGATAAATTGAACAAGTATGGTCGCCCCCTATTGGGCTGTACTATT
AAACCTAAATTGGGGTTATCTGCTAAGAATTACGGTAGAGCTGTTTATGAATGTCTAC
GCGGTGGACTTGATTTTACCAAAGACGACGAGAACGTGAACTCCCAACCATTTATGCG
TTGGAGAGACCGTTTCTTATTTTGTGCCGAAGCAATTTATAAAGCACAGGCGGAAACA
GGTGAAATCAAAGGGCATTATTTGAATGCTACTGCAGGTACATGCGAAGAAATGATC
AAAAGGGCTGTATTTGCCAGAGAATTGGGAGCTCCTATCGTAATGCATGACTACTTAA
CAGGGGGATTCACCGCAAATACTAGCTTGGCTCATTATTGCCGAGATAATGGTTTACT
TCTGCACATCCATCGCGCAATGCATGCAGTTATTGATAGACAGAAGAATCATGGTATG
CACTTTCGTGTACTAGCTAAAGCGTTACGCATGTCTGGTGGAGATCATGTTCACGCGG
GTACCGTAGTAGGGAAACTTGAGGGGGAAAGAGATATCACTTTAGGCTTTGTTGATTT
ACTGCGTGATGATTTTGTTGAAAAAGATAGAAGCCGCGGGGTTTATTTCACTCAAGAC
TGGGTCTCCCTACCAGGTGTTATACCCGTGGCTTCGGGAGGTATTCACGTTTGGCATA
TGCCTGCTCTGACCGAGATCTTTGGAGATGATTCCGTACTACAATTCGGTGGGGGAAC
TTTGGGGCACCCTTGGGGAAATGCACCAGGTGCCGTAGCTAATCGAGTAGCTCTAGA
AGCATGTGTACAAGCTCGTAATGAGGGACGTGATCTTGCTCGTGAGGGTAATGAAATT
ATCCGTGAGGCTAGCAAATGGAGTCCTGAACTAGCTGCTGCTTGTGAAGTGTGGAAA
GAGATTAA-
Banisteriopsis_calcicola --

ATTTAAATTATGTGTCTGATTCACTAATACCTTACCCCATCCATCTAGAAAAATTGGTT
CAAATCCTTCGTTACTGGATGCAAGATCCCTCTTCTTTGCATTTATTACGACTCTTTCTT
CACGAGTATTGGAATTGGAATAGTTTTATTATTTCAACGAAATCCTTTTCCATTTTTTT
TAATTTAAAAAGGAATCTAAGATTATTGTTGTTCCTATATAATTCTCATGTATATGAAT
ACGAATCCATCTTCTTTTTTCTCCGTAACCAATCCTTTTATTTCCAATCAACATTTTCGG
GGGTGCTTCTTGAACGAATATATTTCTATGGAAAAATACAAGATTTTGTAGAACTTTT
TACTAATG------
ATTTTAAGGCTAACCTACGGTTGTTCAAGGATCCTTTCATGCATTATGTTAGATATCAA
GGAAAAGGCATTCTGGCTTCAAAAAGTACACTTCTTCTGATGAAAAAATGGAAATATT
TCTTTGTCAATTTATGTCAATTTCATTTTTATGTGTGGGTTCATCCAGAACCGATCTAT
ATGAAGTCATTATCCAACCATTCTCTTGACTTTTTGGGTTATCTTTCAAATATACGAAA
AAATCTTTCAGTGGTACGGAGTCAAATGCTAGAAAATTCATTTCTAATAGATAATATT
ATGAAGAAACTCGATTCAAGAGTTCCAATTATTCCTTTGATTGGATCATTGTCAAAAA
CAAAAATTTGTAACGCAGTAGGGCATCCCATTAGTAAACCGAGCTGGGCGGATTCAC
CCGATTCTGACATTATCGACCAATTTTTGCGTATATCCAGAAATCTTTCTCATTACTAT
AGCGGATCCTCAAAAAAAAAGGATTTGTATCGAGTAAAATATATACTTCGACTTTCTT
GTGTTAAAACTTTGGCTCGTAAACACAAAAGTACCGTCCGGGCTTTTTTGAAAAAACT
AGGTTCAGAATTATTGGAAGAATTTTTTACGGAGGAAAAATCAATTCTTGATTTGGTC
TTCCCCACGGCTTATTCTATTTCGCGAAGGTTATACAGAGGGCGGGTTTGGTATTTGG
ATATTATTTGTATCAATGATCTAGCCAATCATGAATAAGATTAACTGCATTTTATATGT
TTCGGGTTTATTTACTTACTTTTGAAGGACATTTAAATCTTTATTTTCAAAATTACAGT
GGCAAAAAAAAACAGTGCATTCTATTCAATATCTCTATGGGGTAAAGAAGGATCAAAA

ATACTAAAAAAAAAAAATTCCTTTATTA---------------
TCTTTATTAACAATCACTAATAATGAAAAGACTTCTTTTTTTTTGAACAAAATATACAA
AATTGATGATAATGTAAGAAATATGACGCGTCCCTCTATTACCAT------
TAATCATTTTTTCACGAAAAATGTTTTTCCCTATCCCCCTGAAGCGGATAATACTATGT
TATTTCCTATGCTTGTGTTGGTACTATTTACTTTGTTTATTGGAGCTATAGGAATTCCTT
TCAATCAAGAGGGAACTAATTTGGATATATTATCCAAACTGTTAACTCCGTCTTTAAA
TCTTTTGCATCAAAATGAAAATAATTCTTTTGATTGGTATGAATTTATAACAAATGCTA
TTTTTTCAGTCATTATAACTTTTTTTGGAATATTTATAGCGTCTTCCTTCTATAAACCTC
TTTATTCATCGCTACAAAATTTGAACTTACTTAACTCATTTGCTA------AAAAA---------
AGGGGTCGTTGGGATAAAATAATAGGTGCGATATATGATTGGTCCTATAATCGTGGTT
ATGTCGATGTTTTTTATGCAATATTGGCTTCAAGGTTTCTCTTCATGAAAAACAAGGTA
CGAATGATATGCGATTGTCTGGCCCCTCCAGTCAAGGTAATCCAAGACGAGAGATTGC
CTCAGCCCTTAAGTCTTTGTGGGTCTACATTAAGATCTCCTCATGGTTGCCATTCTCAG
TATATGACAAATATGGGGACAATTGCATCGCTTGTGATGTCTGTGACTATCAACGAAG
ATGATGATACAATGGATGGTGATCAGCAGCAGATGACAAGAAAATTGTGGGGCTTAG
TTGTTTGTCATCACACTAGTCCTCGATTTGTTCCGTTTCCTTTGAGGTATGCTTGTGAGT
TTTTGATTCAAGTGTTTGGTGTTCAGATCAACAAGGAGGTAGAATTAGCTGCTCAGGT
GAGGGAAAAACATATTTTACAAATTCAGACTATGCTATGCGACATGCTCCTTAGAGAT
GCTCCCGTTGCAATTATCACACAATCTCCGAATGTGATGGATCTGGTTAAGTGTGATG
GAGCTGCCTTATACTTCAAGAACAAAACTTGGTTTCTGGGAGTAACGCCTACAGAGGA
ACAAATCAGAGACATAGCCGAATGGCTGCTCGAATATCATAGCGGAAACACGGGTTT
AAGTACTGACAGCCTTATGGAAGCTGGATATCCAGGTGCTTCCGCTCTTGGTGATGCA
GTTTGTGGGATGGCTGCTGTTAGCATTACTTCGAGAGATTTTCTTTTTTGGTTTAGATC
TCACACTGCTAAAGAGATCAAGTGGGGTGGTGCAAAACATGATCCTGATGACAAAGA
TGACTTAAGAAAGATGCATCCGAGGTCATCCTTCAAGGCCTTTCTAGAGGTGGTAAAA

Banisteriopsis_campestris ---

-------------------------GATTAACT-
CATTTTATATGTTTCGGGTTTATTTACTTACTTTTGAAGGACATTTAAATCTTTATTTTC
AAAATTACAGTGGCAAAAAAAAACAGTGCATTCTATTCAATATCTCTATGGGGTAAAG
AAGGATCAAAAATACTAAAAAAAAAAAATTCCTTTATTA--------------

TCTTTATTAACAATCACTAATAATGAAAAGACTTCTTTTTTTTGAACAAAATATACAA
AATTGATGGTACTGTAAGAAATATGACGCGTCCCTCTATTACTAT------
TAATCATTTTTTCACGAAAAATATTTTTCCCTATCCTCCTGAAGCGGATAAAACTATGT
TATTTCCTATGCTTGGGTTGGTACTATTTACTTTGTTTATTGGAGCTATAGGAATTCCTT
TCAATCAAGAGGGAACTAATTTGGATATATTATCCAAACTGTTAACTCCGTCTTTAAA
TCTTTTGCATCAAAATGAAAATAATTCTTTTGATTGGTATGAATTTATAACAAATGCTA
TTTTTTCAGTCATTATAACTTTTTTTGGAATATTTATAGCGTCTTCGTTCTATAAACCTC
TTTATTCATCGCTACAAAATTTGAACTTACTTAACTCATTTGC-----------AA---------
AGGGGTCGTTGGGATAAAATAATAGGTGCGATATATGATTGGTCCTATAATCGTGGTT
ATATCGATGTTTTTTATGCAATATTGGCTTCAAGGTTTCTCTTCATGAAAAACAAGGTA
CGCATGATATGCGATTGTCTGGCCCCTCCCGTCAAGGTAATCCAAGACGAGAGATTGC
CTCAGCCGTTAAGTCTTTGTGGGTCTACATTAAGATCTCCTCATGGTTGCCATTCTCAG
TATATGACAAATATGGGGACAATTGCATCCCTTGTGATGTCTGTGACTATCAACGAAG
ATGATGATACAATGGATGGTGACCAGCAGCAGATGACAAGAAAATTGTGGGGCTTAG
TTGTTTGTCATCATACTAGTCCTCGATTTGTTCCGTTTCCTTTGAGGTATGCTTGTGAAT
TTTTGATTCAAGTGTTTGGTGTTCAGATCAACAAGGAGGTAGAATTAGCTGCTCAGGT
GAGGGAAAAACATATTTTACAAATTCAGACTATGCTATGCGACATGCTCCTTAGAGAT
GCTCCCGTTGCAATTATCACACAATCTCCGAATGTGATGGATCTTGTTAAGTGTGATG
GAGCTGCCTTATACTTCAAGAACAAAACTTGGTTGCTGGGAGTAACGCCTACAGAGG
AACAAATCAGAGACATAGTCGAATGGCTGCTTGAATATCATAGCGGAAACACGGGTT
TAAGTACTGACAGCCTTATGGAAGCTGGATATCCAGGTGCTTCGGCTCTTGGTGATGC
AGTTTGTGGGATGGCTGCTGTTAGCATTACTTCGAGAGATTTTCTTTTTTGGTTTAGAT
CTCACACTGCTAAAGAGATAAAGTGGGGTGGTGCAAAACATGATCCTGATGACAAAG
ATGACTTAAGAAAGATGCATCCGAGGTCATCCTTCAAGGCCTTCCTAGAGGTGGTAAA
A--

Banisteriopsis_confusa --

-??CAAATCCTTCGTTACTGGA
TGCAAGACCCCTCTTCTTTGCATTTATTACGACTCTTTCTTCACGAGTATTGGAATTGG
AATAGTTTTATTATTTCAACGAAATCCTTTTCCATTTTTTTTAATTTAAAAAGGAATCT
AAGATTATTGTTGTTCCTATATAATTCTCATGTATATGAATACGAATCCATCTTCTTTT
TTCTCCGTAACCAATCCTTTTATTTCCAATCAACATTTTCGGGGGTGTTTCTTGAACGA
ATATATTTCTATGGAAAAATACAAGATTTTGTAGAACTTTTTACTAATG------
ATTTTAAGGCTAACCTACGGTTGTTCAAGGATCCTTTCATGCATTATGTTAGATATCAA
GGAAAAGGCATTCTGGCTTCAAAAAGTACACTTCTTCTGATGAAAAAATGGAAATATT
TCTTTGTCAATTTATGTCAATTTCATTTTTATGTGTGGGTTCATCCAGAACCGATCTAT
ATGAAGTCATTATCCAACCATTCTCTTGACTTTTTGGGTTATCTTTCAAATATACGAAA
AAATCTTTCAGTGGTACGGAATCAAATGCTAGAAAATTCATTTCTAATAGATAATATT
ATGAAGAAACTCGATTCAAGAGTTCCAATTATTCCTTTGATTGGATCATTGTCAAAAA
CAAAATTTTGTAACGCAGTAGGGCATCCCATTAGTAAACCGAGCTGGGCGGATTCACC
CGATTCTGACATTATCGACCAATTTTTGCGTATATCCAGAAATCTTTCTCATTACTATA

GCGGATCCTCAAAAAAAAAGAATTTGTATCGAGTAAAATATATACTTCGACTTTCTTG
TATTAAAACTTTGGCTCGTAAACACAAAAGTACCGTCCGGGCTTTTTGAAAAAACTA
GGTTCAGAATTATTGGAAGAATTTTTTACGGAGGAAAAATCAATTCTTGATTTGGTCT
TCCCCACGGCTTATTCTATTTCGCGAAGGTTATACAGAGGGCGGGTTTGGTATTTGGA
TATTATTTGTATCAATGATCTAGCCAATCATGAATAAGATTAACTGCATTTTATATGTT
TCGGGTTTATTTACTTACTTTTGAAGGACATTTAAATCTTTATTTTCAAAATTACAGTG
GCAAAAAAAACAGTGCATTCTATTCAATATCTCTATGGGGTAAAGAAGGATCAAAAA
TACTAAAAAAAAAAATTCCTTTATTA----------------
TCTTTATTAACAATCACTAATAATGAAAAGACTTCTTTTTTTTTGAACAAAATATACAA
AATTGATGGTACTGTAAGAAATATGACGCGTCCCTCTATTACTAT------
TAATCATTTTTTCACGAAAAATATTTTTCCCTATCCTCCTGAAGCGGATAATACTATGT
TATTTCCTATGCTTGTGTTGGTACTATTTACTTTGTTTATTGGAGCTATAGGAATTCCTT
TCAATCAAGAGGGAACTAATTTGGATATATTATCCAAACTGTTAACTCCGTCTTTAAA
TCTTTTGCATCAAAATGAAAATAATTCTTTTGATTGGTATGAATTTATAACAAATGCTA
TTTTTTCAGTCATTATAACTTTTTTTGGAATATTTATAGCGTCTTCGTTCTATAAACCTC
TTTATTCATCGCTACAAAATTTGAACTTACTTAACTCATTTGCTA------AAAAA---------
AGGGGTCGTTGGGATAAAATAATAGGTGCGATATATGATTGGTCCTATAATCGTGGTT
ATATCGATGTTTTTTATGCAATATT????????GGTTTCTCTTCATGAAAAACAAGGTACG
CATGATATGCGATTGTCTGGCCCCTCCCGTCAAGGTAATCCAAGACGAGAGATTGCCT
CAGCCGTTAAGTCTTTGTGGGTCTACATTAAGATCTCCTCATGGTTGCCATTCTCAGTA
TATGACAAATATGGGGACAATTGCATCCCTTGTGATGTCTGTGACTATCAACGAAGAT
GATGATACAATGGATGGTGACCAGCAGCAGATGACAAGAAAATTGTGGGGCTTAGTT
GTTTGTCATCATACTAGTCCTCGATTTGTTCCGTTTCCTTTGAGGTATGCTTGTGAATTT
TTGATTCAAGTGTTTGGTGTTCAGATCAACAAGGAGGTAGAATTAGCTGCTCAGGTGA
GGGAAAAACATATTTTACAAATTCAGACTATGCTATGCGACATGCTCCTTAGAGATGC
TCCCGTTGCAATTATCACACAATCTCCGAATGTGATGGATCTTGTTAAGTGTGATGGA
GCTGCCTTATACTTCAAGAACAAAACTTGGTTGCTGGGAGTAACGCCTACAGAGGAA
CAAATCAGAGACATAGTCGAATGGCTGCTTGAATATCATAGCGGAAACACGGGTTTA
AGTACTGACAGCCTTATGGAAGCTGGATATCCAGGTGCTTCGGCTCTTGGTGATGCAG
TTTGTGGGATGGCTGCTGTTAGCATTACTTCGAGAGATTTTCTTTTTTGGTTTAGATCT
CACACTGCTAAAGAGATAAAGTGGGGTGGTGCAAAACATGATCCTGATGACAAAGAT
GACTTAAGAAAGATGCATCCGAGGTCATCCTTCAAGGCCTTCCTAGAGGTGGTAAAA-

Banisteriopsis_elegans --

ATTTAAATTATGTGTCTGATTCACTAATACCTTACCCCATCCATCTAGAAAAATTGGTT
CAAATCCTTCGTTACTGGATGCAAGATCCCTCTTCTTTGCATTTATTACGACTCTTTCTT
CACGAGTATTGGAATTGGAATAGTTTTATTATTTCAACGAAATCCTTTTCCATTTTTTT
TCATTTAAAAAGGAATCTAAGATTATTGTTGTTCCTATATAATTCTCATGTATATGAAT
ACGAATCCATCTTCTTTTTTCTCCGTAACCAATCCTTTTATTTCCAATCAACATTTTCGG
GGGTGCTTCTTGAACGAATATATTTCTATGGAAAAATACAAGATTTTGTAGAACTTTT

TACTAATG------
ATTTTAAGGCTAACCTACGGTTGTTCAAAGATCCTTTCATGCATTATGTTAGATATCAA
GGAAAAGGCATTCTGGCTTCAAAAAGTACACTTCTTCTGATGAAAAAATGGAAATATT
TCTTTGTCAATTTATGTCAATTTCATTTTTATGTGTCGATTCATCCAGAACCGATCTAT
ATGAAGTCATTATCCAACCATTCTCTTGACTTTTTGGGTTATCTTTCAAGTATACGAAA
AAATCTTTCAGTGGTACGTAGTCAAATGCTAGAAAATTCATTTCTAATAGATAATATT
ATGAAGAAACTCGATTCAAGAGTTCCAATTATTCTTTTGATTGGATCATTGTCAAAAA
CAAAATTTTGTAACGCAGTAGGGTATCCCATTAGT--

GATTAACTGCATTTTATATGTTTCGGGTTTATTTACTTACTTTTGAAGGACATTTAAAT
CTTTATTTTCAAAATTACAGTGGCAAAAAAAACAGTGCATTCTATTCAATATCTCTAT
GGGGTAAAGAAGGATCAAAAATACTAAAAAAAAAAAATTCCTTTATTA---------------
TCTTTATTAACAATCACTAATAATGAAAAGACTTCTTTTTTTTTGAACAAAATATACCA
AATTGATGATAATGTAAGAAATATGACGCGTCCCTCTATTACTAT------
TAATCATTTTTTCACGAAAAATATTTTTCCCTATCCCCCTGAAGCGGATAATACTATGT
TATTTCCTATGCTTGTGTTGGTACTATTTACTTTGTTTATTGGAGCTATAGGAATTCCTT
TCAATCAAGAGGGAACTAATTTGGATATATTATCCAAACTGTTAACTCCGTCTTTAAA
TCTTTTGCATCAAAATGAAAACAATTCTTTTGATTGGTATGAATTTATAACAAATGCTA
TTTTTTCAGTCATTATAACTTTTTTTGGAATATTTATAGCGTCTTCCTTCTATAAACCTC
TTTATTCATCGCTACAAAATTTGAACTTACTTAACTCATTTGCTA------AAAAA---------
AGGGGTCGTTGGGACAAAATAATAGGTGCGATATATGATTGGTCCTATAATCGTGGTT
ATATCGATGTTTTTTATGCAATATT---

ATAAATTGACTTATTATACTCCTGACTATGAAACCAAAGATACTGATATCTTGGCAGC
ATTCCGAGTAACTCCTCAACCTGGAGTTCCGCCTGAGGAAGCAGGCGCTGCGGTAGCT
GCTGAATCTTCTACTGGTACATGGACAACTGTGTGGACCGATGGACTTACCAGTCTTG
ATCGTTATAAAGGGCGATGCTACGGCATCGAGCCCGTTGCTGGAGAAGAAAATCAAT
ATATTGCTTATGTAGCTTACCCCTTAGACCTTTTTGAAGAAGGTTCTGTTACTAATATG
TTTACTTCCATTGTGGGTAATGTATTTGGGTTCAAAGCCCTACGCGCTCTCCGTCTGGA
GGATTTGCGAATCCCTCCTGCTTATACGAAAACTTTCCAAGGCCCGCCTCATGGTATC
CAAGTTGAGAGAGATAAATTGAACAAGTATGGTCGCCCCCTATTGGGCTGTACTATTA
AACCTAAATTGGGGTTATCTGCTAAGAATTACGGTAGAGCTGTTTATGAATGTCTACG
CGGTGGACTTGATTTTACCAAAGACGACGAGAACGTGAACTCCCAACCATTTATGCGT
TGGAGAGACCGTTTCTTATTTTGTGCCGAAGCACTTTATAAAGCACAGGCGGAAACAG
GTGAAATCAAAGGGCATTATTTGAATGCTACTGCAGGTACATGCGAAGAAATGATCA
AAAGGGCTGTATTTGCCAGAGAATTGGGAGCTCCTATCGTAATGCATGACTACTTAAC
AGGGGGATTCACCGCAAATACTAGCTTGGCTCATTATTGCCGAGATAATGGTTTACTT
CTGCACATCCATCGCGCAATGCATGCAGTTATTGATAGACAGAAGAATCATGGTATGC
ACTTTCGTGTACTAGCTAAAGCGTTACGCATGTCTGGTGGAGATCATATTCACGCGGG
TACCGTAGTAGGGAAACTTGAGGGGGAAAGAGATATCACTTTAGGCTTTGTTGATTTA
CTGCGTGATGATTTTGTTGAAAAAGATAGAAGCCGCGGGATTTATTTCACTCAAGACT
GGGTCTCCCTACCAGGTGTTATACCCGTGGCTTCGGGAGGTATTCACGTTTGGCATAT
GCCTGCTCTAACCGAGATCTTTGGAGATGATTCCGTACTACAATTCGGTGGGGGAACT
TTGGGGCACCCTTGGGGAAATGCACCAGGTGCCGTAGCTAATCGAGTAGCTCTAGAA
GCATGTGTACAAGCTCGTAATGAGGGACGTGATCTTGCTCGTGAGGGTAATGAAATTA
TCCGTGAGGCTAGCAAATGGAGTCCTGAACTAGCTGCTGCTTGTGAAGTGTGGAAAG
AGATTAAA
Banisteriopsis_gardeneriana ---

-???????TTATGTGTCTGATTCACTAATACCTTACCCCATCCATCTAGAAAAATTGGTTCA
AATCCTTCGTTACTGGATGCAAGATCCCTCTTTTTTGCATTTATTACGACTCTTTCTTCA
CGAGTATTGGAATTGGAATAGTTTTCTTATTTCAACGAAATCCTTTTCCATTTTTTTTA
ATTTAAAAAGGAATCTAAGATTATTGTTGTTCCTATATAATTCTCATGTATATGAATAC
GAATCCATCTTCTTTTTTCTCCGTAACCAATCCTTTTATTTCCAATCAACATTTTCGGGG
GTGCTTCTTGAACGAATATATTTCTATGGAAAAATACAAGATTTTGTAGAACTTTTTAC
TAATG------
ATTTTAAGGCTAACCTACGGTTGTTCAAGGATCCTTTCATGCATTATGTTAGATATCAA
GGAAAAGGCATTCTGGCTTCAAAAAGTACACTTCTTCTGATGAAAAAATGGAAATATT
TCTTTGTTAATTTATGTCAATTTCATTTTTATGTGTGGGTTCATCCAGAACCGATCTAT
ATGAAGTCATTATCCAACCATTCTCTTGACTTTTTGGGTTATCTTTCAAGTATACGAAA
AAATCTTTCAGTGGTACGGAGTCAAATGCTAGACAATTCATTTCTAATAGATAATATT
ATGAAGAAACTCGATTCAAGAGTTCCAATTATTCCTTTGATTGGATCATTGTCAAAAA
CAAAATTTTGTAACGCAGTAGGGCATCCCATTAGTAAACCGAGCTGGGCGGATTCACC
CGATTCTGATATTATCGACCAATTTTTGCGTATATCCAGAAATCTTTCTCATTACTATA
GCGGATCCTCAAAAAAAAAGAGTTTGTATCGAGTAAAATATATACTTCGACTTTCTTG
TGTTAAAACTTTGGCTCGTAAACACAAAAGTACCGTCCGGGCTTTTTTGAAAAAACTA
GGTTCAGAATTATTGGAAGAATTTTTTACGGAGGAAAAATCGATTCTTGATTTGGTCT
TCCCCACGGCTTATTCTATTTCGCGAAGGTTATACAGAGGGCGGGTTTGGTATTTGGA
TATTATTTGTATCAATGATCTAGCCAATCATGAATAA--

-?????????????????????????????????????ATGATATGCGATTGTCTGGCCCCTCCAGTCAAG
GTAATCCAAGACGAGAGATTGCCTCAGCCGTTAAGTCTTTGTGGGTCTACATTAAGAT
CTCCTCATGGTTGCCATTCTCAGTATATGACAAATATGGGGACAATTGCATCGCTTGT
GATGTCTGTGACTATCAACGAAGATGATGATACGATGGATGGTGATCAGCAGCAGAT
GACAAGAAAATTGTGGGGCTTAGTTGTTTGTCATCATACTAGTCCTCGATTTGTTCCGT
TTCCTTTGAGGTATGCTTGTGAATTTTTGATTCAAGTGTTTGGTGTTCAGATCAACAAG
GAGGTAGAATTAGCTGCTCAGGTGAGGGAAAAACATATTTTACAAATTCAGACTATG
CTATGCGACATGCTCCTTAGAGATGCTCCCGTTGCAATTATCACACAATCTCCGAATG
TGATGGATCTTGTTAAGTGTGATGGAGCTGCCTTATACTTCAAGAACAAAACTTGGTT
GCTGGGAGTAACCCCTACAGAGGAACAAATCAGAGACATAGCCGAATGGCTGCTCGA
ATATCATAGTGGAAACACGGGTTTAAGTACTGACAGCCTTATGGAAGCTGGATATCCA
GGTGCTTCAGCTCTTGGTGATTCAGTTTGTGGGATATATGCTGTTAGCATTACTTCGAG
AGATTTTCTTTTTTGGTTTAGATCTCACACTGCTAAAGAGATCAAGTGGGGCGGTGCA
AAACATGATCCTGATGACAAAGATGACTTGAGAAAGATGCATCCGAGGTCATCCTTC
AAGGCCTTCTTAGAGGTGGTAAA?--

Banisteriopsis_goiana -----
GGATGGTAGGTCCCGTGCTTTTTGCATCGAACTACTAGACCGCAATCCTTTTCAAGTCT
CAGGCATTGCTTTTATGCTGGTGTTATCGAC-------

CGTGTTCCTGTATTTAATACCCATGTTGGAGGAACTAGGCTTCTG-
TTCGCCCCCGCAAATCCACTCGATGCCTCCCGAGGGCATCGGGCGGAGCACGTAGTT-
TGATCTGTCGAGCCTAGCGTGCCTTCTCCTCGGCCGCAACGGTCGTCGGATCAAAGCT
TTTTGTTCTCGGATGCAGAATATTCGATGGGTCCAAGGATTGAATCCTTG--
CATTCGCCCTGTGTGGGAAATCGTCCCTCGATAGGAACGACATCCGTCGTGCGTTGCG
ATCCGTCCGACGTCCTCTCGGGCTGAGGATGCTTCGCGCGTGCGCCGGTGTC-
CCCGAAGGAACGCTACCTGGTTGATCCTGCCAGTAGTCATATGCTTGTCTCAAAGATT
AAGCCATGCATG--

ATTTAAATTATGTGTCTGATTCACTAATACCTTACCCCATCCATCTAGAAAAATTGGTT
CAAATCCTTCGTTACTGGATGCAAGATCCCTCTTCTTTGCATTTATTACGACTCTTTCTT
CACGAGTATTGGAATTGGAATAGTTTTATTATTTCAACGAAATCCTTTTCCATTTTTTT
TAATTTAAAAAGGAATCTAAGATTATTGTTGTTCCTATATAATTCTCATGTATATGAAT
ACGAATCCATCTTCTTTTTTCTCCGTAACCAATCCTTTTATTTCCAATCAACATTTTCGG
GGGTGCTTCTTGAACGAATATATTTCTATGGAAAAATACAAGATTTTGTAGAACTTCT
TACTAATG------
ATTTTAAGGCTAACCTACGGTTGTTCAAGGATCCTTTCATGCATTATGTTAGATATCAA
GGAAAAGGCATTCTGGCTTCAAAAAGTACACTTCTTTTGATGAAAAAATGGAAATATT
TCTTTGTCAATTTATGTCAATTTCATTTTTATGTGTGGGTTCATCCAGAACCGATCTAT
ATGAAGTCATTATCCAACCATTCTCTTGACTTTTTTGGTTATCTTTCAAATATACGAAA
AAATCTTTCAGTGGTACGGAGTCAAATGCTAGAAAATTCATTTCTAATAGATAATATT
ATGAAGAAACTCGATTCAAGAGTTCCAATTATTCCTTTGATTGGATCATTGTCAAAAA
CAAAAATTTGTAACGCAGTAGGGCATCCCATTAGTAAACCGAGCTGGGCGGATTCAC
CCGATTCTGACATTATTGACCAATTTTTGCGTATATCCAGAAATCTTTCTCATTACTAT
AGCGGATCCTCAAAAAAAAAGGATTTGTATCGAGTAAAATATATACTTCGACTTTCTT
GTGTTAAAACTTTGGCTCGTAAACACAAAAGTACCGTCCGGGCTTTTTTGAAAAAACG
AGGTTCAGAATTATTGGAAGAATTTTTTACGGAGGAAAAATCAATTCTTGATTTGGTC
TTCCCCACGGCTTATTCTATTTCGGGAAGGTTATATAGAGGGCGGGTTTGGTATTTGG
ATATTATTTGTATCAATGATCTAGCCAATCATGAA---
GATTAACTGCATTTTATATGTTTCGGGTTTATTTACTTACTTTTGAAGGACATTTAAAT
CTTTATTTTCAAAATTACAGTGGCAAAAAAAACAGTGCATTCTATTCAATATCTCTAT
GGGGTAAAGAAGGATCAAAAATACTAAAAAAAAAAAATTCCTTTATTA---------------
TCTTTATTAACAATCACTAATAATGAAAAGACTTCTTTTTTTTTGAACAAAATATACAA
AATTGATGATAATGTAAGAAATATGACGCGTCCCTCTATTACTAT------
TAATCATTTTTTCACGAAAAAGATTTTTCCCTATCCTCCTGAAGCGGATAATACTATGT
TATTTCCTATGCTTGTGTTGGTACTATTTACTTTGTTTATTGGAGCTATAGGAATTCCTT
TCAATCAAGAGGGAACTAATTTGGATATATTATCCAAACTGTTAACTCCGTCTTTAAA
TCTTTTGCATCAAAATGAAAATAATTCTTTTGAGTGGTATGAATTTATAACAAATGCT
ATTTTTTCAGTCATTATAACTTTTTTTGGAATATTTATAGCGTCTTCCTTCTATAAACCT
CTTTATTCATCGCTACAAAATTTGAACTTACTTAACTCATTTGCTA------AAAAA---------
AGGGGTCGTTGGGATAAAATAATAGGTGCGATATATGATTGGTCCTATAATCGTGGTT
ATATCGATGTTTTTTATGCAATATT--

Banisteriopsis_harleyi
TGTTGGGATGGTAGGCTCCGTGCTTTTTGCATCGAACTGCCAGGCCGCAATCCTTTTC
AAGCCTCAAGCATTGCTTC-GTGCTTGTGTTATCGAC-------
CGTGTTCCTGTATTTGATACCCATGTCGGAGGAACTAGGCTTCCG-TTCTCCAC----
AATCCACTCGACGCCTCTTACGGGCATCGAGCGGAGCACGTAGTT-
TGATCCGTCGAGCCTAGTATGCCTG---TTCGGTTGCAACGGCCGATGGATCAAAGC-
TCGTGTTCTCGGATGCAGAATATTCGATGGGTCCAAGGATTGAATCCTTG--
CATTTGACCCGTGTGGGAAATCGTCCCTCGATGAGAACGACGTCCGTTGTGCGCTGCG
ATCCGTCCGATGTCCTCTCGGACCGAGGATGCTTCGTGCGTGCTTCGGCGTC-
CTCGAAGGAATGCTACCTGGTTGATCCTGCCAGTAGTCATATGCTTGTCTCAAAGATT
AAGCCATGCATGCCTGCCCAGCAGAAAGGCCCGGGAACCG-
TTAACGGAGCGCGCCGGGGAACGGGCCGGGCCGAAGGTTCCGCCCGCTTCCAGAAGC
AGCCCGCCGGGTGGCCCC-
GGCCGCCTGGGGGAAAGACCCCCACCCCCGGGGCAAGACGGGCCAAGGGAAAC-
CAAATAAGGAGGAATTTTCCC--
CGGGTTCCCAACGCGGGGAGGGCGGGGAACGTTCCGCCAAAGGTGG-
AACAAAAAAGACTTTCGGCAACGGATATTTCGGCTCTCGCATCGAGGAAGAACGTAG
CAAAATGCGATACTTGGTGTGAATTGCAGAATCCCGGGAACCATCGAGTC-
TTGAACCCAAGTTGCGCCCGAAGCCTTTCGGTCGAGGGCACGTCTCCCTGGGTGTCAC
ACAACGTCGTCCCCAAACCCGACACC-CTCTCCCACG----GGGGGAGT-----
CGGGAGGGACGGAAGCTGGTCTCCCGTGGCGATCCGCCGCGGTTGGCCGAATTACGA
GTCCGGGCGAGGAAAGCCGCAACGACCGGAGAAGGGAAC-
ATCCTCGATCGATGCTCGTTGCGGCCGTTTT-
CGCGAGGGGACGGGCTCCAGACCCACGGCGATTCCGGCGCCTTCGATCGCGACCCCA
GGTCAGGCGGGATA??????????????????????CTAATACCTTACCCCATCCATCTAGAAAA
ATTGGTTCAAATCCTTCGTTACTGGATGCAAGACCCCTCTTCTTTGCATTTATTACGAC
TCTTTCTTCACGAGTATTGGAATTGGAATAGTTTTATTATTTCAACGAAATCCTTTTCC
ATTTTTTTTAATTTAAAAAGGAATCTAAGATTATTGTTGTTCCTATATAATTCTCATGT
ATATGAATACGAATCCATCTTCTTTTTTCTCCGTAACCAATCCTTTTATTTCCAATCAA
CATTTTTGGGGGTGCTTCTTGAACGAATATATTTCTATGGAAAAATACAAGATTTTGT
AGAACTTTTTACTAATG------
ATTTTAAGGCTAACCTACGGTTGTTCAAGGATCCTTTCATGCATTATGTTAGATATCAA
GGAAAAGGCATTCTGGCTTCAAAAAGTACACTTCTTCTGATGAAAAAATGGAAATATT
TCTTTGTCAATTTATGTCAATTTCATTTTTATGTGTGGGTTCATCCAGAACCGATCTAT
ATGAAGTCATTATCCAACCATTCTCTTGACTTTTTGGGTTATCTTTCAAATATACGAAA
AAATCTTTCAGTGGTACGGAGTCAAATGCTAGAAAATTCATTTCTAATAGATAATATT
ATGAAGAAACTCGATTCAAGAGTTCCAATTATTCCTTTGATTGGATCATTGTCAAAAA
CAAAATTTTGTAACGCAGTAGGGCATCCCATTAGTAAACCGAGCTGGGCGGATTCACC
CGATTCTGACATTATCGACCAATTTTTGCGTATATCCAGAAATCTTTCTCATTACTATA
GCGGATCCTCAAAAAAAAAGAATTTGTATCGAGTAAAATATATACTTCGACTTTCTTG
TGTTAAAACTTTGGCTCGTAAACACAAAAGTACCGTCCGGGCTTTTTTGAAAAAACTA
GGTTCAGAATTATTGGAAGAATTTTTTACGGAGGAAAAATCAATTCTTGATTTGGTCT
TCCCCACGGCTTATTCTATTTCGCGAAGGTTATACAGAGGGCGGGTTTGGTATTTGGA
TATTATTTGTATCAATGATCTAGCCAATCATGAATAAGATTAACTGCATTTTATATGTT
TCGGGTTTATTTACTTACTTTTGAAGGACATTTAAACCTTTATTTTCAAAATTACAGTG
GCAAAAAAAACAGTGCATTCTATTCAATATCTCTATGGGGTAAAGAAGGATCAAAAA
TACTAAAAAAAAAAAATTCCTTTATTA---------------
TCTTTATTAACAATCACTAATAATGAAAAGACTTCTTTTTTTTTGAACAAAATATACAA
AATTGATGGTAATGTAAGAAATATGACGCGTCCCTCTATTACTAT------
TAATCATTTTTTCACGAAAAATATTTTTCCCTATCCCCCTGAAGCGGATAATACTATGT
TATTTCCTATGCTTGTATTGGTACTATTTACTTTGTTTATTGGAGCTATAGGAATTCCTT
TCAATCAAGAGGGAACTAATTTGGATATATTATCCAAACTGTTAACTCCGTCTTTAAA
TCTTTTGCATCAAAATGAAAATAATTCTTTTGATTGGTATGAATTTATAACAAATGCTA
TTTTTTCAGTCATTATAACTTTTTTTGGAATATTTATAGCGTCTTCCTTCTATAAACCTC
TTTATTCATCTCTACAAAATTTGAACTTACTTAACTCATTTGCTA------AAAAA---------
AGGGGTCGTTGGGATAAAATAATAGGTGCGATATATGATTGGTCCTATAATCGTGGTT

ATATCGATGTTTTTTATGCAATATT?GCTTCAAGGTTTCTTTTCATGAAAAACAAGGTA
CGCATGATATGCGATTGTCTGGCCCCTCCAGTCAAGGTAATCCAAGACAAGAGATTGC
CTCAGCCGTTAAGTCTTTGTGGGTCTACATTAAGATCTCCTCATGGTTGCCATTCTCAG
TATATGACAAATATGGGGACAATTGCATCGCTTGTGATGTCTGTGACTATCAACGAAG
ATGACGATACGATGGATGGTGATCAGCAGCAGATGACAAGAAAATTGTGGGGCTTAG
TTGTTTGTCATCATACTAGTCCTCGATTTGTTCCATTTCCTTTGAGGTATGCTTGTGAAT
TTCTGATTCAAGTGTTTGGTGTTCAGATCAACAAGGAGGTAGAATTAGCTGCTCAGGT
GAGGGAAAAACATATTTTACAAATTCAGACTATGCTATGCGACATGCTCCTTAGAGAT
GCTCCAGTTGCAATTATCACAGAATCTCCGAATGTGATGGATCTTGTTAAGTGTGATG
GAGCTGCCTTATACTTCAAGAACAAAACTTGGTTGCTGGGAGTAACCCCTACAGAGG
AACAAATCAGAGACATAGCCGAATGGCTGCTCGAATGTCATAACGGAAACACGGGTT
TAAGTACTGACAGCCTTATGGAAGCTGGATATCCAGGTGCTTCAGCTCTTGGTGATGC
AGTTTGTGGGATGGCTGCTATTAGCATTACTTCGAGAGATTTTCTTTTTTGGTTTAGAT
CTCACACTGCCAAAGAGATCAAGTGGGGTGGTGCAAAACATGATCCTGATGACAAAG
ATGACTTAAGAAAGATGCATCCGAGGTCATCCTTCAAGGCCTTTCTAGAGGTGGTAAA
A---

Banisteriopsis_irwinni --

ATTAACTGCATTTTATATGTTTCGGGTTTATTTACTTACTTTTGAAGGACATTTAAATC
TTTATTTTCAAAATTACAGTGGCAAAAAAAAACAGTGCATTCTATTCAATATCTCTATG
GGGTAAAGAAGGATCAAAAATACTAAAAAAAAAAAATTCCTTTATTA---------------
TCTTTATTAACAATCACTAATAATGAAAAGACTTCTTTTTTTTTGAACAAAATATACAA
AATTGATGATAATGTAAGAAATATGACGCGTCCCTCTATTACTAT------
TAATCATTTTTTCACGAAAAAGATTTTTCCCTATCCTCCTGAAGCGGATAATACTATGT
TATTTCCTATGCTTGTGTTGGTACTATTTACTTTGTTTATTGGAGCTATAGGAATTCCTT
TCAATCAAGAGGGAACTAATTTGGATATATTATCCAAACTGTTAACTCCGTCTTTAAA
TCTTTTGCATCAAAATGAAAATAATTCTTTTGAGTGGTATGAATTTATAACAAATGCT
ATTTTTTCAGTCATTATAACTTTTTTTGGAATATTTATAGCGTCTTCCTTCTATAAACCT
CTTTATTCATCGCTACAAAATTTGAACTTACTTAACTCATTTGCTA------AAAAA---------
AGGGGTCGTTGGGATAAAATAATAGGTGCGATATATGATTGGTCCTATAATCGTGGTT
ATATCGATGTTTTTTATGCAATATT--

Banisteriopsis_laevifolia --

CCTGCCCAGCAGAACGACCCGCGAACCGTTTGTCAAAGCGCGTCGAGGAGCGGGCCG
GGCCGAMGGTCCCGTCCCCTTCCCGACGCAGCCCGTCGTGTGGCCTC-
GGCCGCCTGCGGGAACAACAACCACCCCCGGCGCAGAACGCGCCAAGGAAAAC-
AAACTAAGGAGGAATCTTCTC--GTGCTCCCGAACTCGGCGAGCAGGGGA-
ACGTTCCGCCAAAGGTCG-
AACAAAAACGACTCTCGGCAACGGATATCTCGGCTCTCGCATCGATGAAGAACGTAG
CAAAATGCGATACTTGGTGTGAATTGCAGAATCCCGTGAACCATCGAGTCTTTGAACG
CAAGTTGCGCCCGAAGCCTTTCGGCCGAGGGCACGTCTGCCTGGGTGTCACACAACGT
CGTCCCCAAACCCGACCCT-CTCTCCCACG--GGAAGGGAGT-----
CTGGAGGGACGGAAGCTGGTCTCCCGTGGCGTTCCGCCGCGGTTGGCCGAATTCCGA
GTCCGGGCGAGGAAAGCCGCAACAACCGGGGGTTGGAACGATCCTCGATCGATGC--
GTTGCGGCCGTTTC-TTCGCGGAGACGGGCTCCAGACCCACGGCG-
CTCCGGCGCCTTCGATCGCGACCCCAGGTCAGGCGGGATCATTTAAATTATGTGTCTG
ATTCACTAATACCTTACCCCATCCATCTAGAAAAATTGGTTCAAATCCTTCGTTACTGG
ATGCAAGATCCCTCTTCTTTGCATTTATTACGACTCTTTCTTCACGAGTATTGGAATTG
GAATAGTTTTATTATTTCAACGAAATCCTTTTCCATTTTTTTGAATTTAAAAAGGAATC
TAAGATTATTGTTGTTCCTATATAATTCTCATGTATATGAATACGAATCCATCTTCTTT
TTTCTCCGTAACCAATCCTTTTATTTCCAATCAACATTTTCGGGGGTGCTTCTTGAACG
AATATATTTCTATGGAAAAATACAAGATTTTGTAGAACTTTTTACTAATG------
ATTTTAAGGCTAACCCACGGTTGTTCAAGGATCCTTTCATGCATTATGTTAGATATCAA
GGAAAAGGCATTCTGGCTTCAAAAAGTACACTTCTTCTGATGAAAAAATGGAAATATT
TCTTTGTCAATTTATGTCAATTTCATTTTTATGTGTGGGTTCATCCAGAACCGATCCAT
ATGAAGTCATTATCCAACCATTCTCTTGACTTTTTGGGTTATCTTTCAAATATACGAAA
AAATCTTTCAGTGGTACGGAGTCAAATGCTAGAAAATTCATTTCTAATAGATAATATT
ATGAAGAAACTCGATTCAAGAGTTCCAATTATTCCTTTGATTGGATCATTGTCAAAAA
CAAAATTTTGTAACGCAGTAGGGCATCCCATTAGTAAACCGAGCTGGGCGGATTCACC
CGATTCTGACATTATCGACCAATTTTTGCGTATATCCAGAAATCTTTCTCATTACTATA
GCGGATCCTCAAAAAAAAAGAATTTGTATCGAGTAAAATATATACTTCGACTTTCTTG
TGTTAAAACTTTGGCTCGTAAACACAAAAGTACCGTCCGGGCTTTTTTGAAAAAACTA
GGTTCAGAATTATTGGAAGAATTTTTTACGGAGGAAAAATCAATTCTTGATTTGGTCT
TCCCCACGGCTTATTCTATTTCGCGAAGGTTATACAGAGGGCGGGTTTGGTATTTGGA
TATTATTTGTATCAATGATCTAGCCAATCATGAATAAGATTAACTGCATTTTATATGTT
TCGGGTTTATTTACTTACTTTTGAAGGACATTTAAATCTTTATTTTCAAAATTACAGTG
GTAAAAAAAACAGTGCATTCTATTCAATATCTCTATGGGGTAAAGAAGGATCAAAAA
TACTAAAAAAAAAAAATGCCTTTATTA---------------
TCTTTATTAACAATCACTAATAATGAAAAGACTTCTTTTTTTTGAACAAAATATACAA
AATTGATGGTAATGTAAGAAATATGACGCGTCCCTCTATTACTAT------

TAATCATTTTTTCACGAAAAAGATTTTTCCCTATCCCCCTGAAGCGGATAATACTATGT
TATTTCCTATGCTTGTATTGGTACTATTTACTTTGTTTATTGGAGCTATAGGAATTCCTT
TCAATCAAGAAGGAACTAATTTGGATATATTATCCAAACTGTTAACTCCGTCTTTAAA
TCTTTTGCATCAAAATGAAAATAATTCTTTTGATTGGTATGAATTTATAACAAATGCTA
TTTTTTCAGTCATTATAACTTTTTTTGGAATATTTATAGCGTCTTCCTTCTATAAACCTC
TTTATTCATCTCTACAAAATTTGAACTTACTTAACTCATTTGCTA------AAAAA---------
AGGGGTCGTTGGGATAAAATAATAGGTGCGATATATGATTGGTCCTATAATCGTGGTT
ATATCGATGTTTTTTATGCAATATTAGCTTCAAGGTTTCTCTTCATGAAAAACAAGGTA
CGCATGATATGCGATTGCTTGGCCCCTCCAGTCAAGGTAACCCAAGACAAGAGATTGC
CTCAGCCGTTAAGTCTTTGTGGGTCTACATTAAGATCTCCTCATGGTTGCCATTCTCAG
TATATGACAAATATGGGGACAATTGCATCGCTTGTGATGTCTGTGACTATCAACGAAG
ATGATGATACAATGGATGGTGATCAGCAGCAGATGACAAGAAAATTGTGGGGCTTAG
TTGTTTGTCATCATACTAGTCCTCGATTTGTTCCGTTTCCTTTGAGGTATGCTTGTGAAT
TTCTGATTCAAGTGTTTGGTGTTCAGATCAACAAGGAGGTAGAATTAGCTGCTCAGGT
GAGGGAAAAACATATTTTACAAATTCAGACTATGCTATGTGACATGCTCCTTAGAGAT
GCTCCCGTTGCAATTATCACACAATCTCCGAATGTGATGGATCTTGTTAAGTGTGATG
GAGCTGCCTTATACTTCAAGAACAAAACTTGGTTGCTGGGAGTAACCCCTACAGAGG
AACAAATCAGAGACATAGCCGAATGGCTGCTCGAATATCATAGTGGAAACACGGGTT
TAAGTACTGACAGCCTTATGGAAGCTGGATATCCAGGTGCTTCAGCTCTTGGTGATGC
AGTTTGTGGGATGGCTGCCGTTAGCATTACTTCGAGAGATTTTCTTTTTTGGTTTAGAT
CTCACACTGCCAAAGAGATCAAGTGGGGTGGTGCAAAACATGATCCTGATGACAAAG
ATGACTTAAGAAAGATGCATCCGAGGTCATCCTTCAAGGCCTTTCTAGAGGTGGTAAA
AATAAATTGACTTATTATACTCCTGACTATGAAACCAAAGATACTGATATCTTGGCAG
CATTCCGAGTAACTCCTCAACCTGGGGTTCCGCCTGAGGAAGCAGGCGCTGCGGTAGC
TGCTGAATCTTCTACTGGTACATGGACAACTGTGTGGACCGATGGACTTACCAGTCTT
GATCGTTATAAAGGGCGATGCTACGGCATCGAGCCCGTTGCTGGAGAAGAAAATCAA
TATATTGCTTATGTAGCTTACCCCTTAGACCTTTTTGAAGAAGGTTCTGTTACTAACAT
GTTTACTTCCATTGTGGGTAATGTATTTGGGTTCAAAGCCCTACGCGCTCTCCGTCTGG
AGGATTTGCGAATCCCTCCTGCTTATACGAAAACTTTCCAAGGCCCGCCTCATGGTAT
CCAAGTTGAGAGAGATAAATTGAACAAGTATGGTCGCCCCCTATTGGGCTGTACTATT
AAACCTAAATTGGGGTTATCTGCTAAGAATTACGGTAGAGCTGTTTATGAATGTCTAC
GCGGTGGACTTGATTTTACCAAAGACGACGAGAACGTGAACTCCCAACCATTTATGCG
TTGGAGAGACCGTTTCTTATTTTGTGCCGAAGCACTTTATAAAGCACAGGCGGAAACA
GGTGAAATCAAAGGGCATTATTTGAATGCTACTGCAGGTACATGCGAAGAAATGATC
AAAAGGGCTGTATTTGCCAGAGAATTGGGAGCTCCTATCGTAATGCATGACTACTTAA
CAGGGGGATTCACCGCAAATACTAGCTTGGCTCATTATTGCCGAGATAATGGTTTACT
TCTGCACATCCATCGCGCAATGCATGCAGTTATTGATAGACAGAAGAATCATGGTATG
CACTTTCGTGTACTAGCTAAAGCGTTACGCATGTCTGGTGGAGATCATATTCACGCGG
GTACCGTAGTAGGGAAACTTGAGGGGGAAAGAGATATCACTTTAGGCTTTGTTGATTT
ACTGCGTGATGATTTTGTTGAAAAAGATAGAAGCCGCGGGATTTATTTCACTCAAGAC
TGGGTCTCCCTACCAGGTGTTATACCCGTGGCTTCGGGAGGTATTCACGTTTGGCATA
TGCCTGCTCTGACCGAGATCTTTGGAGATGATTCCGTACTACAATTCGGTGGGGGAAC
TTTGGGGCACCCTTGGGGAAATGCACCAGGTGCCGTAGCTAATCGAGTAGCTCTAGA
AGCATGTGTACAAGCTCGTAATGAGGGACGTGATCTTGCTCGTGAGGGTAATGAAATT
ATCCGTGAGGCTAGCAAATGGAGTCCTGAACTAGCTGCTGCTTGTGAAGTGTGGAAA
GAGATTAA-
Banisteriopsis_latifolia
TGTTGGGATGGTAGGCTCCGTGCTTTTTGCATCGAACTGCTAGACCGCAATCCTTTTC
AAGTCTCGAGCATTGCTTTTATGCTCGGGTTATCGAC-------
CGTGTTCCTGTATTTGATACCCATGTTGGAGGAACTAGGCTTCTG-TTCTCCCC----
AGTCCGCTCGATGCCTCTTACGGGCATCGAGCGGAGCACGTAGTT-
TGATCTGTCGAGCCTAGCATGCCTT---CTCGGCTGCAACGGTCGTCGGATCAAAGC-
TCGTGTTCTCGGATGCAGAACACTCGATGGGTCCAAGGATTGAATCCTTG--
CATTAGCCCTGTGTGGGAAATCGTCCCTCGATAAGAACGACGTCCGCCGTGCGTCGCG
ATCCGTCCGATGTCCTCTCGGGACGAGGACGCTTCGTGCGTGCGTCGGTGTC-
CTCGAAGGAATGCTACCTGGTTGATCCTGCCAGTAGTCATATGCTTGTCTCAAAGATT
AAGCCATGCATG--

-?????????????????????CACTAATACCTTACCCCATCCATCTAGAAAAATTGGTTCAAATC
CTTCGTTACTGGATGCAAGATCCCTCTTCTTTGCATTTATTACGACTCTTTCTTCACGA
GTATTGGAATTGGAATAGTTTTATTATTTCAACGAAATCCTTTTCCATTTTTTTTAATTT
AAAAAGGAATCTAAGATTATTGTTGTTCCTATATAATTCTCATGTATATGAATACGAA
TCCATCTTCTTTTTTCTCCGTAACCAATCCTTTTATTTCCAATCAACATTTTCGGGGGTG
TTTCTTGAACGAATATATTTCTATGGAAAAATACAAGATTTTGTAGAACTTTTTACTAA
TG------
ATTTTAAGGCTAACCTACGGTTGTTCAAGGATCCTTTCATGCATTATGTTAGATATCAA
GGAAAAGGCATTCTGGCTTCAAAAAGTACACTTCTTCTGATGAAAAAATGGAAATATT
TCTTTGTCAATTTATGTCAATTTCATTTTTATGTGTGGGTTCATCCAGAACCGATCTAT
ATGAAGTCATTATCCAACCATTCTCTTGACTTTTTGGGTTATCTTTCAAATATACGAAA
AAATCTTTCAGTGGTACGGAATCAAATGCTAGAAAATTCATTTCTAATAGATAATATT
ATGAAGAAACTCGATTCAAGAGTTCCAATTATTCCTTTGATTGGATCATTGTCAAAAA
CAAAATTTTGTAACGCAGTAGGGCATCCCATTAGTAAACCGAGCTGGGCGGATTCACC
CGATTCTGACATTATCGACCAATTTTTGCGTATATCCAGAAATCTTTCTCATTACTATA
GCGGATCCTCAAAAAAAAAGAATTTGTATCGAGTAAAATATATACTTCGACTTTCTTG
TATTAAAACTTTGGCTCGTAAACACAAAAGTACCGTCCGGGCTTTTTTGAAAAAACTA
GGTTCAGAATTATTGGAAGAATTTTTTACGGAGGAAAAATCAATTCTTGATTTGGTCT
TCCCCACGGCTTATTCTATTTCGCGAAGGTTATACAGAGGGCGGGTTTGGTATTTGGA
TATTATTTGTATCAATGATCTAGCCAATCATGAATAAGATTAACTGCATTTTATATGTT
TCGGGTTTATTTACTTACTTTTGAAGGACATTTAAATCTTTATTTTCAAAATTACAGTG
GCAAAAAAAACAGTGCATTCTATTCAATATCTCTATGGGGTAAAGAAGGATCAAAAA
TACTAAAAAAAAAAAATTCCTTTATTA---------------
TCTTTATTAACAATCACTAATAATGAAAAGACTTCTTTTTTTTTGAACAAAATATACAA
AATTGATGGTACTGTAAGAAATATGACGCGTCCCTCTATTACTAT------
TAATCATTTTTTCACGAAAAATATTTTTCCCTATCCTCCTGAAGCGGATAATACTATGT
TATTTCCTATGCTTGTGTTGGTACTATTTACTTTGTTTATTGGAGCTATAGGAATTCCTT
TCAATCAAGAGGGAACTAATTTGGATATATTATCCAAACTGTTAACTCCGTCTTTAAA
TCTTTTGCATCAAAATGAAAATAATTCTTTTGATTGGTATGAATTTATAACAAATGCTA
TTTTTTCAGTCATTATAACTTTTTTTGGAATATTTATAGCGTCTTCGTTCTATAAACCTC
TTTATTCATCGCTACAAAATTTGAACTTACTTAACTCATTTGCTA------AAAAA---------
AGGGGTCGTTGGGATAAAATAATAGGTGCGATATATGATTGGTCCTATAATCGTGGTT
ATATCGATGTTTTTTATGCAATATTGGCTTCAAGGTTTCTCTTCATGAAAAACAAGGTA
CGCATGATATGCGATTGTCTGGCCCCTCCMGTCAAGGTAATCCAAGACGAGAGATTG
CCTCAGCCGTTAAGTCTTTGTGGGTCTACATTAAGATCTCCTCATGGTTGCCATTCTCA
GTATATGACAAATATGGGGACAATTGCATCCCTTGTGATGTCTGTGACTATCAACGAA
GATGATGATACAATGGATGGTGACCAGCAGCAGATGACAAGAAAATTGTGGGGCTTA
GTTGTTTGTCATCATACTAGTCCTCGATTTGTTCCGTTTCCTTTGAGGTATGCTTGTGA
ATTTTTGATTCAAGTGTTTGGTGTTCAGATCAACAAGGAGGTAGAATTAGCTGCTCAG
GTGAGGGAAAAACATATTTTACAAATTCAGACTATGCTATGCGACATGCTCCTYAGAG
ATGCTCCCGTTGCAATTATCACACAATCTCCRAATGTGATGGATCTTGTTAAGTGTGAT
GGAGCTGCCTTATACTTCAAGAACAAAACTTGGTTGCTGGGAGTAACGCCTACAGAG
GAACAAATCAGAGACATAGTCGAATGGCTGCTTGAATATCATAGCGGAAACACGGGT
TTAAGTACTGACAGCCTTATGGAAGCTGGATATCCAGGTGCTTCGGCTCTTGGTGATG
CAGTTTGTGGGATGGCTGCTGTTAGCATTACTTCGAGAGATTTTCTTTTTTGGTTTAGA
TCTCACACTGCTAAAGAGATAAAGTGGGGTGGTGCAAAACATGATCCTGATGACAAA
GATGACTTAAGAAAGATGCATCCGAGGTCATCCTTCAAGGCCTTCCTAGAGG????????-
----

Banisteriopsis_malifolia TGTTGGGATGGTAGGCTCCGTGC-
TTTTGCATCGAACTGCTAGCCCGCAATCCTTTTCAAGTCTCGAGCATTGCTTTTATGCT
CGTGTTTTTG??------
-?GTGTTCCTGTATTTGATACCCATGTTGGCGGAACTAGGCTTCTG-TTCTCCCC-------
CCACTCGGTGCCTCTTACGGGCA-
CGGGTGGACCACACAGCTCTGGCCCATTATTCCTAGCAAGCTCT---
TCGGGCTGCAACGGTTGTTGGATCAAAGC-
TCGTGTTCTCGGATGCAGAATAC?CGATGGGTCCAGGGATTGAATCCTTG--
CATTCGCCCCGTGTGGGAAA?CGTCCCTCGATAGGAACGACATCCGCTGCGCGCCGCG
ATCCGTCC---GTCCTCTCGGGCCCAGGACGCTTCGCGCGTGCACCGGTGTC-
CCCGAAGGAATGCTACCTGGTTGATCCTGCCAGTAGTCATATGCTTGTCTCAAAGATT
AAGCCATGCATGCCTGCCCAGCAGAACGACTCGCGAACCG-
TGGTTACATCGTGCCGAGGGGCGA-
CGGGGCCACTCGCCCCGCCGTTTCCCGGACGCAGCCCGTCGCATGGCCTCGGGCGCCC
GACGGGACTCGCAACCAACCCCGGCGCAGAACGCGCCAAGGAAAACGAAATCGAGG
AGGATCGTCCCT--GCGCTCCCGGAGACGGGGCGCGCTCGG-
ACGTCCCGCCCAATGTCG-AAC-
AAAACGACTCTCGGCAACGGATATCTCGGCTCTCGCATCGATGAAGAACGTAGCAAA
ATGCGATACTTGGTGTGAATTGCAGAATCCCGTGAACCATCGAGTCTTTGAACGCAAG
TTGCGCCCGAAGCCTTCTGGCCGAGGGCACGTCTGCCTGGGTGTCACACAACGTTGTC
CCCAAACCCATCCC----------------GAGAGGGA-----
AGGGAGGGACGGAAGCTGGTCTCCCGTGGCGATCCGTCGCGGTTGGCCGAATCCCAA
GTCCGGGAGAGGGGAGCCGCGACTAACGGTGGTT-GAACGACCCTCGGCTCAGGC----
CGTGGCCTTTTT-CGCGAGGAGACGGGCTCAAGACCCGCGTCG-
CTTGGGCGCCTTCAATCGCGACCCCAGGTCAGGCGGGACTATTTAAATTATGTGTCTG
ATTCACTAATACCTTACCCCATCCATCTAGAAAAATTGGTTCAAATCCTTCGTTACTGG
ATGCAAGATCCCTCTTCTTTGCATTTATTACGACTCTTTCTTCACGAGTATTGGAATTG
GAATAGTTTTCTTATTTCAACGAAATCCTTTTCCATTTTTTTGAATTTAAAAAGGAATC
TAAGATTATTGTTGTTCCTATATAATTCTCATGTATATGAATACGAATCCATCTTCTTT
TTTCTCCGTAACCAATCCTTTTATTTCCAATCAACATTTTCGGGGGTGTTTCTTGAACG
AATATATTTCTATGGAAAAATACAAGATTTTGTAGAACTTTTTACTAATG------
ATTTTAAGGCTAACCTACGGTTGTTCAAGGATCCTTTCATGCATTATGTTAGATATCAA
GGAAAAGGCATTCTGGCTTCAAAAAGTACACTTCTTCTGATGAAAAAATGGAAATATT
TCTTTGTCAATTTATGTCAATTTCATTTTTATGTGTGGGTTCATCCAGAACCGATCTAT
ATGAAGTCATTATCCAACCATTCTCTTGACTTTTTGGGTTATCTTTCAAATATACGAAA
AAATCTTTCAGTGGTACGGAGTCAAATGCTAGAAAATTCATTTCTAATAGATAATATT
ATGAAGAAACTCGATTCAAGAGTTCCAATTATTCCTTTGATTGGATCATTGTCAAAAA
CAAAATTTTGTAACGCAGTAGGGCATCCCATTAGTAAACCGAGCTGGGCGGATTTACC
CGATTCTGACATTATCGACCAATTTTTGCGTATATCCAGAAATCTTTCTCATTACTATA
GCGGATCCTCAAAAAAAAAGAATTTGTATCGAGTAAAATATATACTTCGACTTTCTTG
TGTTAAAACTTTGGCTCGTAAACACAAAAGTACCGTCCGGGCTTTTTTGAAAAAACTA
GGTTCAGAATTATTGGAAGAATTTTTTACGGAGGAAAAATCAATTCTTGATTTGGTCT
TCCCCACGGCTTATTCTATTTCGCGAAGGTTATACAGAGGGCGGGTTTGGTATTTGGA
TATTATTTGTATCAATGATCTAGCCAATCATGAA---
GATTAACTGCATTTTATATGTTTCGGGTTTATTTACTTACTTTTGAAGGACATTTAAAT
CTTTATTTTCAAAATTACAGTGGCAAAAAAAACAGTGCATTCTATTCAATATCTCTAT
GGGGTAAAGAAGGATCAAAAATACTAAAAAAAAAAATTCCTTTATTA---------------
TCTTTATTAACAATCACTAATAATGAAAAGACTTCTTTTTTTTTGAACAAAATATACAA
AATTGATGGTACTGTAAGAAATATGACGCGTCCCTCTATTACTAT------
TAATCATTTTTTCACGAAAAATATTTTTCCCTATCCTCCTGAAGCGGATAATACTATGT
TATTTCCTGTGCTTGTGTTGGTACTATTTACTTTGTTTATTGGAGCTATAGGAATTCCTT
TCAATCAAGAGGGAACTAATTTGGATATATTATCCAAACTGTTAACTCCGTCTTTAAA
TCTTTTGCATCAAAATGAAAATAATTCTTTTGATTGGTATGAATTTATAACAAATGCTA
TTTTTTCAGTCATTATAACTTTTTTTGGAATATTTATAGCGTCTTCCTTCTATAAACCTC
TTTATTCATCGCTACAAAATTTGAACTTACTTAACTCATTTGCTA------AAAAA---------
AGGGGTCGTTGGGATAAAATAATAGGTGCGATATATGATTGGTCCTATAATCGTGGTT
ATATCGATGTTTTTTATGCAATATTAGCTTCAAGGTTTCTCTTCATGAAAAACAAGGTA
CGCATGATATGCGATTGTCTGGCCCCTCCAGTCAAGGTAATCCAAGACGAGAGATTGC

CTCAGCCGTTAAGTCTTTGTGGGTCTACATTAAGATCTCCTCATGGTTGCCATTCTCAG
TATATGACAAATATGGGGACAATTGCATCCCTTGTGATGTCTGTGACTATCAACGAAG
ATGATGATACAATGGATGGTGACCAGCAGCAGATGACAAGAAAATTGTGGGGCTTAG
TTGTTTGTCATCATACTAGTCCTCGATTTGTTCCGTTTCCTTTGAGGTATGCTTGTGAAT
TTTTGATTCAAGTGTTTGGTGTTCAGATCAACAAGGAGGTAGAATTAGCTGCTCAGGT
GAGGGAAAAACATATTTTACAAATTCAGACTATGCTATGTGACATGCTCCTCAGAGAT
GCTCCAGTTGCAATTATCACACAATCTCCGAATGTGATGGATCTTGTTAAGTGTGATG
GAGCTGCCTTATACTTCAAGAACAAAACTTGGTTGCTGGGAGTAACGCCTACAGAGG
AACAAATCAGAGACATAGTCGAATGGCTGCTTGAATATCATAGCGGAAACACGGGTT
TAAGTACTGACAGCCTTATGGAAGCTGGATATCCAGGTGCTTCGGCTCTTGGTGATGC
AGTTTGTGGGATGGCTGCTGTTAGCATTACTTCGAGAGATTTTCTTTTTTGGTTTAGAT
CTCACACTGCTAAAGAGATAAAGTGGGGTGGTGCAAAACATGATCCTGATGACAAAG
ATGACTTAAGAAAGATGCATCCGAGGTCATCCTTCAAGGCCTTCCTAGAGGTGGTAAA
AATAAATTGACTTATTATACTCCTGACTATGAAACCAAAGATACTGATATCTTGGCAG
CATTCCGAGTAACTCCTCAACCTGGAGTTCCGCCTGAGGAAGCAGGCGCTGCGGTAGC
TGCTGAATCTTCTACTGGTACATGGACAACTGTGTGGACCGATGGACTTACCAGTCTT
GATCGTTATAAAGGGCGATGCTACGGCATCGAGCCCGTTGCTGGAGAAGAAAATCAA
TATATTGCTTATGTAGCTTACCCCTTAGACCTTTTTGAAGAAGGTTCTGTTACTAACAT
GTTTACTTCCATTGTGGGTAATGTATTTGGGTTCAAAGCCCTACGCGCTCTCCGTCTGG
AGGATTTGCGAATCCCTCCTGCTTATACGAAAACTTTCCAAGGCCCGCCTCATGGTAT
CCAAGTTGAGAGAGATAAATTGAACAAGTATGGTCGCCCCCTATTGGGCTGTACTATT
AAACCTAAATTGGGGTTATCTGCTAAGAATTACGGTAGAGCTGTTTATGAATGTCTAC
GCGGTGGACTTGATTTTACCAAAGACGACGAGAACGTGAACTCCCAACCATTTATGCG
TTGGAGAGACCGTTTCTTATTTTGTGCCGAAGCAATTTATAAAGCACAGGCGGAAACA
GGTGAAATCAAAGGGCATTATTTGAATGCTACTGCAGGTACATGCGAAGAAATGATC
AAAAGGGCTGTATTTGCCAGAGAATTGGGAGCTCCTATCGTAATGCATGACTACTTAA
CAGGGGGATTCACCGCAAATACTAGCTTGGCTCATTATTGCCGAGATAATGGTTTACT
TCTGCACATCCATCGCGCAATGCATGCAGTTATTGATAGACAGAAGAATCATGGTATG
CACTTTCGTGTACTAGCTAAAGCGTTACGCATGTCTGGTGGAGATCATGTTCACGCGG
GTACCGTAGTAGGGAAACTTGAGGGGGAAAGAGATATCACTTTAGGCTTTGTTGATTT
ACTGCGTGATGATTTTGTTGAAAAAGATAGAAGCCGCGGGGTTTATTTCACTCAAGAC
TGGGTCTCCCTACCAGGTGTTATACCCGTGGCTTCGGGAGGTATTCACGTTTGGCATA
TGCCTGCTCTGACCGAGATCTTTGGAGATGATTCCGTACTACAATTCGGTGGGGGAAC
TTTGGGGCACCCTTGGGGAAATGCACCAGGTGCCGTAGCTAATCGAGTAGCTCTGGA
AGCATGTGTACAAGCTCGTAATGAGGGACGTGATCTTGCTCGTGAGGGTAATGAAATT
ATCCGTGAGGCTAGCAAATGGAGTCCTGAACTAGCTGCTGCTTGTGAAGTGTGGAAA
GAGATTAAA
Banisteriopsis_martiniana --

ATTTAAATTATGTGTCTGATTCACTAATACCTTACCCCATCCATCTAGAAAAATTGGTT
CAAATCCTTCGTTACTGGATGCAAGATCCCTCTTCTTTGCATTTATTACGACTCTTTCTT
CACGAGTATTGGAATTGGAATAGTTTTATTATTTCAACGAAATCCTTTTCCATTTTTTT
TAATTTAAAAAGGAATCTAAGATTATTGTTGTTCCTATATAATTCTCATGTATATGAAT
ACGAATCCATCTTCTTTTTTCTCCGTAACCAATCCTTTTATTTCCAATCAACATTTTCGG
GGGTGCTTCTTGAACGAATATATTTCTATGGAAAAATACAAGATTTTGTAGAACTTTT
TACTAATG------
ATTTTAAGGCTAACCTACGGTTGTTCAAAGATCCTTTCATGCATTATGTTAGATATCAA
GGAAAAGGCATTCTGGCTTCAAAAAGTACACTTCTTCTGATGAAAAAATGGAAATATT
TCTTTGTCAATTTATGTCAATTTCATTTTTATGTGTCGGTTCATCCAGAACCGATCTAT
ATGAAGTCATTATCCAACCATTCTCTTGACTTTTTGGGTTATCTTTCAAGTATACGAAA
AAATCTTTCAGTGGTACGTAGTCAAATGCTAGAAAATTCATTTCTAATAGATAATATT
ATGAAGAAACTCGATTCAAGAGTTCCAATTATTCCTTTGATTGGATCATTGTCAAAAA

CAAAATTTTGTAACGCAGTAGGGTATCCCATTAGTAAACCGAGCTGGGCGGATTCACC
CGATTCTGATATTATCGACCAATTTTTGCGTATATCCAGAAATCTTTCTCATTACTATA
GTGGATCCTCAAAAAAAAAGAGTTTGTATCGAGTAAAATATATACTTCGACTTTCTTG
TGTTAAAACTTTGGCTCGTAAACACAAAAGTACCGTCCGGGCTTTTTTGAAAAAACTA
GGTTCAGAATTATTGGAAGAATTTTTTACGGAGGAAAAATCGATTCTTGATTTGGTCT
TCCCCACGGCTTATTCTATTTTGCGAAGGTTATACAGAGGGCGGGTTTGGTATTTGGA
TATTATTTGTATCAATGATCTAGCCAATCATGAATAAGATTAACTGCATTTTATATGTT
TCGGGTTTATTTACTTACTTTTGAAGGGCATTTAAATCTTTATTTTCAAAATTACAGTG
GCAAAAAAAAGAGTGCATTCTATTCAATATCTCTATGGGGTAAAGAAGGATCAAAAA
TACTAAAAAAAAAAAATTCCTTTATTA---------------
TCTTTATTAACAATCACTAATAATAAAAAGACTTCTTTTTTTTTGAACAAAATATACCA
AATTGATGGTAATGTAAGAAATATGACGCGTCCCTCTATTACTAT------
TAATCATTTTTTCACGAAAAATATTTTTCCCTATCCCCCTGAAGCGGATAACACTATGT
TATTTCCTATGCTTGTGTTGGTACTATTTACTTTGTTTATTGGAGCTATAGGAATTCCTT
TCAATCAAGAGGGAACTAATTTGGATATATTATCCAAACTGTTAACTCCGTCTTTAAA
TCTTTTGCATCAAAATGAAAACAATTCTTTTGATTGGTATGAATTTATAACAAATGCTA
TTTTTTCAGTCATTATAACTTTTTTTGGAATATTTATAGCGTCTTCCTTCTATAAACCTC
TTTATTCATCGCTACAAAATTTGAACTTACTTAACTCATTTGCTA------AAAAA---------
AGGGGTCGTTGGGACAAAATAATAGGTGCGATATATGATTGGTCCTATAATCGTGGTT
ATATCGATGTTTTTTATGCAATATTAGCTTCAAGGTTTCTCTTCATGAAAAATAAGGTA
CGCATGATATGCGATTGTCTGGCCCCTCCAGTCAAGGTAATCCAAGACGAGAGATTGC
CTCAGCCGTTAAGTCTTTGTGGGTCTACATTAAGATCTCCTCATGGTTGCCATTCTCAG
TATATGACAAATATGGGGACAATTGCATCGCTTGTGATGTCTGTGACTATCAACGAAG
ATGATGATACGATGGATGGTGATCAGCAGCAGATGACAAGAAAATTGTGGGGCTTAG
TTGTTTGTCATCATACTAGTCCTCGATTTGTTCCTTTTCCTTTGAGGTATGCTTGTGAAT
TTTTGATTCAAGTGTTTGGTGTTCAGATCAACAAGGAGGTAGAACTAGCTGCTCAGGT
GAGGGAAAAACATATTTTACAAATTCAGACTATGCTATGCGACATGCTCCTTAGAGAT
GCTCCTGTTGCAATTATCACACAATCTCCGAATGTGATGGATCTTGTTAAGTGTGATG
GAGCTGCTTTATACTTCAAGAACAAAACTTGGTTGCTGGGAGTAACCCCTACAGAGGA
ACAAATCAGAGACATAGCCGAATGGCTGCTCCAATATCATGGCGGAAACACGGGTTT
AAGTACTGACAGCCTTATGGAAGCTGGATATCCAGGTGCTTCAGCTCTTGGTGATGCA
GTTTGTGGGATGGCTGCTGTTAGCATTACTTCGAGAGATTTTCTTTTTTGGTTTAGATC
TCACACTGCTAAAGAGATCAAGTGGGGCGGTGCAAAACATGATCCTGACGACAAAGA
TGACTTAAGAAAGATGCATCCTAGGTCATCCTTCAAGGCCTTTCTAGAGGTGGTAAAA
ATAAATTGACTTATTATACTCCTGACTATGAAACCAAAGATACTGATATCTTGGCAGC
ATTCCGAGTAACTCCTCAACCTGGAGTTCCGCCTGAGGAAGCAGGCGCTGCGGTAGCT
GCTGAATCTTCTACTGGTACATGGACAACTGTGTGGACCGATGGACTTACCAGTCTTG
ATCGTTATAAAGGGCGATGCTACGGCATCGAGCCCGTTGCTGGAGAAGAAAATCAAT
ATATTGCTTATGTAGCTTACCCCTTAGACCTTTTTGAAGAAGGTTCTGTTACTAATATG
TTTACTTCCATTGTGGGTAATGTATTTGGGTTCAAAGCCCTACGCGCTCTCCGTCTGGA
GGATTTGCGAATCCCTCCTGCTTATACGAAAACTTTCCAAGGCCCGCCTCATGGTATC
CAAGTTGAGAGAGATAAATTGAACAAGTATGGTCGCCCCCTATTGGGCTGTACTATTA
AACCTAAATTGGGGTTATCTGCTAAGAATTACGGTAGAGCTGTTTATGAATGTCTACG
CGGTGGACTTGATTTTACCAAAGACGACGAGAACGTGAACTCCCAACCATTTATGCGT
TGGAGAGACCGTTTCTTATTTTGTGCCGAAGCAATTTATAAAGCACAGGCGGAAACAG
GTGAAATCAAAGGGCATTATTTGAATGCTACTGCAGGTACATGCGAAGAAATGATCA
AAAGGGCTGTATTTGCCAGAGAATTGGGAGCTCCTATCGTAATGCATGACTACTTAAC
AGGGGGATTCACCGCAAATACTAGCTTGGCTCATTATTGCCGAGATAATGGTTTACTT
CTGCACATCCATCGCGCAATGCATGCAGTTATTGATAGACAGAAGAATCATGGTATGC
ACTTTCGTGTACTAGCTAAAGCGTTACGCATGTCTGGTGGAGATCATATTCACGCGGG
TACCGTAGTAGGGAAACTGGAGGGGGAAAGAGATATCACTTTAGGCTTTGTTGATTTA
CTGCGTGATGATTTTGTTGAAAAAGATAGAAGCCGCGGGATTTATTTCACTCAAGACT
GGGTCTCCCTACCAGGTGTTATACCCGTGGCTTCGGGAGGTATTCACGTTTGGCATAT
GCCTGCTCTAACCGAGATCTTTGGAGATGATTCCGTACTACAATTCGGTGGGGGAACT
TTGGGGCACCCTTGGGGAAATGCACCAGGTGCCGTAGCTAATCGAGTAGCTCTAGAA
GCATGTGTACAAGCTCGTAATGAGGGACGTGATCTTGCTCGTGAGGGTAATGAAATTA
TCCGTGAGGCTAGCAAATGGAGTCCTGAACTAGCTGCTGCTTGTGAAGTGTGGAAAG
AGATTAA-
Banisteriopsis_megaphylla ---

ATTTAAATTATGTGTCTGATTCACTAATACCTTACCCCATCCATCTAGAAAAATTGGTT
CAAATCCTTCGTTACTGGATGCAAGATCCCTCTTCTTTGCATTTATTACGACTCTTTCTT
CACGAGTATTGGAATTGGAATAGTTTTATTATTTCAACGAAATCCTTTTCCATTTTTTT
TAATTTAAAAAGGAATCTAAGATTATTGTTGTTCCTATATAATTCTCATGTATATGAAT
ACGAATCCATCTTCTTTTTTCTCCGTAACCAATCCTTTTATTTCCAATCAACATTTTCGG
GGGTGTTTCTTGAACGAATATATTTCTATGGAAAAATACAAAATTTTGTAGAACTTTTT
ACTAATG------
ATTTTAAGGCTAACCTACGGTTGTTCAAGGATCCTTTCATGCATTATGTTAGATATCAA
GGAAAAGGCATTCTGGCTTCAAAAAGTACACTTCTTCTGATGAAAAAATGGAAATATT
TCTTTGTCAATTTATGTCAATTTCATTTTTATGTGTGGGTTCATCCAGAACCGATCTAT
ATGAAGTCATTATCCAACCATTCTCTTGACTTTTTGGGTTATCTTTCAAATATACGAAA
AAATCTTTCAGTGGTACGGAATCAAATGCTAGAAAATTCATTTCTAATAGATAATATT
ATGAAGAAACTCGATTCAAGAGTTCCAATTATTCCTTTGATTGGATCATTGTCAAAAA
CAAAATTTTGTAACGCAGTAGGACATCCCATTAGTAAACCGAGCTGGGCGGATTCACC
CGATTCTGACATTATCGACCAATTTTTGCGTATATCCAGAAATCTTTCTCATTACTATA
GCGGATCCTCAAAAAAAAAGAATTTGTATCGAGTAAAATATATACTTCGACTTTCTTG
TATTAAAACTTTGGCTCGTAAACACAAAAGTACCGTCCGGGCTTTTTTGAAAAAACTA
GGTTCAGAATTATTGGAAGAATTTTTTACGGAGGAAAAATCAATTCTTGATTTGGTCT
TCCCCACGGCTTATTCTATTTCGCGAAGGTTATACAGAGGGCGGGTTTGGTATTTGGA
TATTATTTGTATCAATGATCTAGCCAATCATGAA---
GATTAACTGCATTTTATATGTTTCGGGTTTATTTACTTACTTTTGAAGGACATTTCAAT
CTTTATTTTCAAAATTACAGTGGCAAAAAAAAACAGTGCATTCTATTCAATATCTCTAT
GGGGTAAAGAAGGATCAAAAATACTAAAAAAAAAAAATTCCTTTATTA--------------
TCTTTATTAACAATCACTAATAATGAAAAGACTTCTTTTTTTTTGAACAAAATATACAA
AATTGATGGTACTGTAAGAAATATGACGCGTCCCTCTATTACTAT------
TAATCATTTTTTCACGAAAAATATTTTTCCCTATCCTCCTGAAGCGGATAATACTATGT
TATTTCCTATGCTTGTGTTGGTACTATTTACTTTGTTTATTGGAGCTATAGGAATTCCTT
TCAATCAAGAGGGAACTAATTTGGATATATTATCCAAACTGTTAACTCCGTCTTTAAA
TCTTTTGCATCAAAATGAAAAGAATTCTTTTGATTGGTATGAATTTATAACAAATGCT
ATTTTTTCAGTCATTATAACTTTTTTTGGAATATTTATAGCGTCTTCGTTCTATAAACCT
CTTTATTCATCGCTACAAAATTTGAACTTACTTAACTCATTTGCTA------AAAAA---------
AGGGGTCGTTGGGATAAAATAATAGGTGCGATATATGATTGGTCCTATAATCGTGGTT
ATATCGATGTTTTTTATGCAATATT--

Banisteriopsis_membranifolia ---

GATTAACTGCATTTTATATGTTTCGGGTTTATTTACTTACTTTTGAAGGACAATTAAAT
CTTTATTTTCAAAATTACAGTGGCAAAAAAAAACAGTGCATTCTATTCAATATCTCTAT
GGGGTAAAGAAGGATCAAAAATACTAAAAAAAAAAAATTCCTTTATTA---------------
TCTTTATTAACAATCACTAATAATGAAAAGACTTCTTTTTTTTTGAACAAAATATACAA
AATTGATGGTAATGTAAGAAATATGACGCGTCCCTCTATTACTAT------
TAATCATTTTTTCACGAAAAATATTTTTCCCTATCCCCCTGAAGCGGATAAAACTATGT
TATTTCCTATGCTTGTATTGGTACTATTTACTTTGTTTATTGGAGCTATAGGAATTCCTT
TCAATCAAGAGGGAACTAATTTGGATATATTATCCAAACTGTTAACTCCGTCTTTAAA
TCTTTTGCATCAAAATGAAAATAATTCTTTTGATTGGTATGAATTTATAACAAATGCTA
TTTTTTCAGTCATTATAACTTTTTTTGGAATATTTATAGCGTCTTCCTTCTATAAACCTC
TTTATTCATCTCTACAAAATTTGAACTTACTTAACTCATTTGCTA------AAAAA---------
AGGGGTCGTTGGGATAAAATAATAGGTGCAATATATGATTGGTCCTATAATCGTGGTT
ATATCGATGTTTTTTATGCAATATT--

Banisteriopsis_muricata
TGTTGGGATGGTAGGCTCCGTGCTTTTTGCATCGAACTACTAGACCGCAATCCTTTTC
AAGTCTCGGGCATTGCTTTTATGCCCGTGYTATCGAC-------
CGTGTTCCTGTATTTAATACCCATGTTGGAGGAACTAGGCCTCTG-
TTCGCCCCCGCAAATCCACTCGATGCCTCCCGAGGGCATCGAGCGGAGCACGTAGTT-
TGATCTGTCGAGCCTAGCGTGCCTTCTCCTCGGCCGCAACGGTCGTCGGATCAAAGCC
TTGTGTTCTCGGATGCAGAATATTCGATGGGTCCAAGGATTGAATCCTTG--
CATTCGCCCTGTGTGGGAAATCGTCCCTCGATAGGAACGACGTCCGTCGTGCGTCGCG
ATCCGTCCGACGTCCTCTCGGGCCGAGGACGCTTCGCGCGTGCGCCGGCGTC-

CCCGAAGGAACGCTACCTGGTTGATCCTGCCAGTAGTCATATGCTTGTCTCAAAGATT
AAGCCATGCAGGCCTGCCCAGCAGAACGACCCGCGAACCG-
TAGTCCGATCGCGTCGGGGAGAGGGCCGGGGCGACCGTCCCTCCCTCTTCCCGGCGCA
GCCCGCCGCGCGGCCTCTGGCCGTCGGCGGGAACAACAACCACCCCCGGCGCAGAAC
GCGCCAAGGAAAAC-AAACTAAGGAGGAACGTCCCC--
GTGCTCCCGGACACGGCGGGCGCGCGG-
ACGTTCCGCCAAAGGTCGAACGAAAAACGACTCTCGGCAACGGATATCTCGGCTCTC
GCATCGATGAAGAACGTAGCAAAATGCGATACTTGGTGTGAATTGCAGAATCCCGTG
AACCATCGAGTCTTTGAACGCAAGTTGCGCCCGAAGCCATCCGGTCGAGGGCACGTCT
GCCTGGGTGTCACACAACGTCGTCCCCAAACCCGACCCCTCCCTAAAACG----
GGGGGAGTCGAGACGAGAGGGACGGAAGCTGGTCTCCCGTGGCGATCCGCCGCGGTT
GGCCGAATCCAGAGTCCCGGCGAGGAAAGCCGCAGCGACCGGCGGTTGGAAC-
CTCCTCGATCGTTGC--GTTGCGGCCCTTTC-
CGCGCGGAGACGGGCTCCAGACCCACGGCG-
CTCCGGCGCCTTCGATCGCGACCCCAGGTCAGGCGGGATCATTTAAATTATGTGTCTG
ATTCACTAATACCTTACCCCATCCATCTAGAAAAATTGGTTCAAATCCTTCGTTACTGG
ATGCAAGATCCCTCTTCTTTGCATTTATTACGACTCTTTCTTCACGAGTATTGGAATTG
GAATAGTTTTATTATTTCAACGAAATCCTTTTCCATTTTTTTTAATTTAAAAAGGAATC
TAAGATTATTGTTGTTCCTATATAATTCTCATGTATATGAATACGAATCCATCTTCTTT
TTTCTCCGTAACCAATCCTTTTATTTCCAATCAACATTTTCGGGGGTGCTTCTTGAACG
AATATATTTCTATGGAAAAATACAAGATTTTGTAGAACTTCTTACTAATG------
ATTTGAAGGCTAACCTACGGTTGTTCAAGGATCCTTTCATGCATTATGTTAGATATCA
AGGAAAAGGCATTCTGGCTTCAAAAAGTACACTTCTTTTGATGAAAAAATGGAAATA
TTTCTTTGTCAATTTATGTCAATTTCATTTTTATGTGTGGGTTCATCCAGAACCGATCTA
TATGAAGTCATTATCCAACCATTCTCTTGACTTTTTGGGTTATCTTTCAAATATACGAA
AAAATCTTTCAGTGGTACGGAGTCAAATGCTAGAAAATTCATTTCTAATAGATAATAT
TATGAAGAAACTCGATTCAAGAGTTCCAATTATTCCTTTGATTGGATCATTGTCAAAA
ACAAAAATTTGTAACGCAGTAGGGCATCCCATTAGTAAACCGAGCTGGGCGGATTCA
CCCGATTCTGACATTATTGACCAATTTTTGCGTATATCCAGAAATCTTTCTCATTACTA
TAGCGGATCCTCAAAAAAAAAGGATTTGTATCGAGTAAAATATATACTTCGACTTTCT
TGTGTTAAAACTTTGGCTCGTAAACACAAAAGTACCGTCCGGGCTTTTTTGAAAAAAC
GAGGTTCAGAATTATTGGAAGAATTTTTTACGGAGGAAAAATCAATTCTTGATTTGGT
CTTCCCCACGGCTTATTCTATTTCGGGAAGGTTATATAGAGGGCGGGTTTGGTATTTG
GATATTATTTGTATCAATGATCTAGCCAATCATGAATAAGATTAACTGCATTTTATATG
TTTCGGGTTTATTTACTTACTTTTGAAGGACATTTAAATCTTTATTTTCAAAATTACAG
TGGCAAAAAAAACAGTGCATTCTATTCAATATCTCTATGGGGTAAAGAAGGATCAAA
AATACTAAAAAAAAAAAATTCCTTTATTA---------------
TCTTTATTAACAATCACTAATAATGAAAAGACTTCTTTTTTTTTGAACAAAATATACAA
AATTGATGATAATGTAAGAAATATGACGCGTCCCTCTATTACTAT------
TAATCATTTTTTCACGAAAAAGATTTTTCCCTATCCCCCTGAAGCGGATAATACTATGT
TATTTCCTATGCTTGTGTTGGTACTATTTACTTTGTTTATTGGAGCTATAGGAATTCCTT
TCAATCAAGAGGGAACTAATTTGGATATATTATCCAAACTGTTAACTCCGTCTTTAAA
TCTTTTGCATCAAAATGAAAATAATTCTTTTGAGTGGTATGAATTTATAACAAATGCT
ATTTTTTCAGTCATTATAACTTTTTTTGGAATATTTATAGCGTCTTCCTTCTATAAACCT
CTTTATTCATCGCTACAAAATTTGAACTTACTTAACTCATTTGCTA------AAAAA---------
AGGGGTCGTTGGGATAAAATAATAGGTGCGATATATGATTGGTCCTATAATCGTGGTT
ATATCGATGTTTTTTATGCAATATTAGCTTCAAGGTTTCTCTTCATGAAAAACAAGGTA
CGCATGATATGCGATTGTCTGGCCCCTCCAGTCAAGGTAATCCAAGACGAGAGATTGC
CTCAGCCGTTAAGTCTTTGTGGGTCTACATTAAGATCTCCTCATGGTTGCCATTCTCAG
TATATGACAAATATGGGGACAATTGCATCGCTTGTGATGTCTGTGACTATCAACGAAG
ATGATGATACAATGGATGGTGATCAGCAGCAGATGACAAGAAAATTGTGGGGCTTAG
TTGTTTGTCATCATACTAGTCCTCGATTTGTTCCGTTTCCTTTGAGGTATGCTTGTGAAT
TTTTGATTCAAGTGTTTGGTGTTCAGATCAACAAGGAGGTAGAATTAGCTGCTCAGGT
GAGGGAAAAACATATTTTACAAATTCAGACTATGCTATGCGACATGCTCCTTAGAGAT
GCTCCTGTTGCAATTATCACACAATCTCCGAATGTGATGGATCTTGTTAAGTGTGATG
GAGCTGCCTTATTCTTCAAGAACAAAACTTGGTTGCTGGGAGTAACGCCTAAAGAGG
AACAAATCAGAGACATAGCCGAATGGCTGCTCGAATATCATAGTGGAAACACGGGTT
TAAGTACTGACAGCCTGATGGAAGCTGGATATCCAGGTGCTTCTGCTCTTGGTGATGC
AGTTTGTGGGATGGCTGCTGTTAGCATTACTTCAAGAGATTTTCTTTTTTGGTTTAGAT
CTCACACTGCTAAAGAGATCAAGTGGGGTGGTGCAAAACATGATCCTGATGACAAAG

ATGACTTAAGAAAGATGCATCCTAGGTCATCTTTCAAGGCCTTTCTAGAGGTGGTAAA
AATAAATTGACTTATTATACTCCTGACTATGAAACCAAAGATACTGATATCTTGGCAG
CATTCCGAGTAACTCCTCAACCTGGAGTTCCGCCTGAGGAAGCAGGCGCTGCGGTAGC
TGCTGAATCTTCTACTGGTACATGGACAACTGTGTGGACCGATGGACTTACCAGTCTT
GATCGTTATAAAGGGCGATGCTACGGCATCGAGCCCGTTGCTGGAGAAGAAAATCAA
TATATTGCTTATGTAGCTTACCCCTTAGACCTTTTTGAAGAAGGTTCTGTTACTAACAT
GTTTACTTCCATTGTGGGTAATGTATTTGGGTTCAAAGCCCTACGCGCTCTCCGTCTGG
AGGATTTGCGAATCCCTCCTGCTTATACGAAAACTTTCCAAGGCCCGCCTCATGGTAT
CCAAGTTGAGAGAGATAAATTGAACAAGTATGGTCGCCCCCTATTGGGCTGTACTATT
AAACCTAAATTGGGGTTATCTGCTAAGAATTACGGTAGAGCTGTTTATGAATGTCTAC
GCGGTGGACTTGATTTTACCAAAGACGACGAGAACGTGAACTCCCAACCATTTATGCG
TTGGAGAGACCGTTTCTTATTTTGTGCCGAAGCACTTTATAAAGCACAGGCGGAAACA
GGTGAAATCAAAGGGCATTATTTGAATGCTACTGCAGGTACATGCGAAGAAATGATC
AAAAGGGCTGTATTTGCCAGAGAATTGGGAGCTCCTATCGTAATGCATGACTACTTAA
CAGGGGGATTCACCGCAAATACTAGCTTGGCTCATTATTGCCGAGATAATGGTTTACT
TCTGCACATCCACCGCGCAATGCATGCAGTTATTGATAGACAGAAGAATCATGGTATG
CACTTTCGTGTACTAGCTAAAGCGTTACGCATGTCTGGTGGAGATCATATTCACGCGG
GTACCGTAGTAGGGAAACTTGAGGGGGAAAGAGATATCACTTTAGGCTTTGTTGATTT
ACTGCGTGATGATTTTGTTGAAAAAGATAGAAGCCGCGGGATTTATTTCACTCAAGAC
TGGGTCTCCCTACCAGGTGTTATACCCGTGGCTTCGGGAGGTATTCACGTTTGGCATA
TGCCTGCTCTGACCGAGATCTTTGGAGATGATTCCGTACTACAATTCGGTGGGGGAAC
TTTGGGGCACCCTTGGGGAAATGCACCAGGTGCCGTAGCTAATCGAGTAGCTCTAGA
AGCATGTGTACAAGCTCGTAATGAGGGACGTGATCTTGCTCGTGAGGGTAATGAAATT
ATCCGTGAGGCTAGCAAATGGAGTCCTGAACTAGCTGCTGCTTGTGAAGTGTGGAAA
GAGATTAA-
Banisteriopsis_nummifera ---

ATTTAAATTATGTGTCTGATTCACTAATACCTTACCCCATCCATCTAGAAAAATTGGTT
CAAATCCTTCGTTACTGGATGCAAGATCCCTCTTTTTTGCATTTATTACGACTCTTTCTT
CACGAGTATTGGAATTGGAATAGTTTTCTTATTTCAACGAAATCCTTTTCCATTTTTTT
TAATTTAAAAAGGAATCTAAGATTATTGTTGTTCCTATATAATTCTCATGTATATGAAT
ACGAATCCATCTTCTTTTTTCTCCGTAACCAATCCTTTTATTTCCAATCAACATTTTCGG
GGGTGCTTCTTGAACGAATATATTTCTATGGAAAAATACAAGATTTTGTAGAACTTTT
TACTAATG------
ATTTTAAGGCTAACCTACGGTTGTTCAAGGATCCTTTCATGCATTATGTTAGATATCAA
GGAAAAGGCATTCTGGCTTCAAAAAGTACACTTCTTCTGATGAAAAAATGGAAATATT
TCTTTGTTAATTTATGTCAATTTCATTTTTATGTGTGGGTTCATCCAGAACCGATCTAT
ATGAAGTCATTATCCAACCATTCTCTTGACTTTTTGGGTTATCTTTCAAGTATACGAAA
AAATCTTTCAGTGGTACGGAGTCAAATGCTAGACAATTCATTTCTAATAGATAATATT
ATGAAGAAACTCGATTCAAGAGTTCCAATTATTCCTTTGATTGGATCATTGTCAAAAA
CAAAATTTTGTAACGCAGTAGGGCATCCCATTAGTAAACCGAGCTGGGCGGATTCACC
CGATTCTGATATTATCGACCAATTTTTGCGTATATCCAGAAATCTTTCTCATTACTATA
GCGGATCCTCAAAAAAAAAGAGTTTGTATCGAGTAAAATATATACTTCGACTTTCTTG
TGTTAAAACTTTGGCTCGTAAACACAAAAGTACCGTCCGGGCTTTTTTGAAAAAACTA
GGTTCAGAATTATTGGAAGAATTTTTTACGGAGGAAAAATCGATTCTTGATTTGGTCT
TCCCCACGGCTTATTCTATTTCGCGAAGGTTATACAGAGGGCGGGTTTGGTATTTGGA
TATTATTTGTATCAATGATCTAGCCAATCATGAATAAGATTAACTGCATTTTATATGTT
TCGGGTTTATTTACTTACTTTTGAAGGACATTTAAATCTTTATTTTCAAAATTACAGTG
GCAAAAAAAACAGTGCATTCTATTCAATATCTCTATGGGGTAAAGAAGGATCAAAAA
TACTAAAAAAAAAAAATTCCTTTATTA---------------
TCTTTATTAACAATCACTAATAATGAAAAGACTTCTTTTTTTTTGAACAAAATAGACCA
AATTGATGGTAATGTAAGAAATATGACGCGTCCCTCTATTACTAT------

TAATCATTTTTTCACGAAAAATATTTTTCCCTATCCCCCTGAAGCGGATAATACTATGT
TATTTCCTATGCTTGTGTTGGTACTATTTACTTTGTTTATTGGAGTTATAGGAATTCCTT
TCAATCAAGAGGGAACTAATTTGGATATATTATCCAAACTGTTAACTCCGTCTTTAAA
TCTTTTGCATCAAAATGAAAATAATTCTTTTGATTGGTATGAATTTATAACAAATGCTA
TTTTTTCAGTCATTATAACTTTTTTTGGAATATTTATAGCGTCTTCCTTCTATAAACCTC
TTTATTCATCGCTACAAAATTTGAACTTACTTAACTCATTTGCTAAAAAT-AAAAA-------
--
AGGGGTCGTTGGGACAAAATAATAGGTGCGATATATGATTGGTCCTATAATCGTGGTT
ATATCGATGTTTTTTATGCAATATT?GCTTCAAGGTTTCTCTTCATGAAAAACAAGGTA
CGCATGATATGCGATTGTCTGGCCCCTCCAGTCAAGGTAATCCAAGACGAGAGATTGC
CTCAGCCGTTAAGTCTTTGTGGGTCTACATTAAGATCTCCTCATGGTTGCCATTCTCAG
TATATGACAAATATGGGGACAATTGCATCGCTTGTGATGTCTGTGACTATCAACGAAG
ATGATGATACGATGGATGGTGATCAGCAGCAGATGACAAGAAAATTGTGGGGCTTAG
TTGTTTGTCATCATACTAGTCCTCGATTTGTTCCGTTTCCTTTGAGGTATGCTTGTGAAT
TTTTGATTCAAGTGTTTGGTGTTCAGATCAACAAGGAGGTAGAATTAGCTGCTCAGGT
GAGGGAAAAACATATTTTACAAATTCAGACTATGCTATGCGACATGCTCCTTAGAGAT
GCTCCCGTTGCAATTATCACACAATCTCCGAATGTGATGGATCTTGTTAAGTGTGATG
GAGCTGCCTTATACTTCAAGAACAAAACTTGGTTGCTGGGAGTAACCCCTACAGAGG
AACAAATCAGAGACATAGCCGAATGGCTGCTCGAATATCATAGTGGAAACACGGGTT
TAAGTACTGACAGCCTTATGGAAGCTGGATATCCAGGTGCTTCAGCTCTTGGTGATTC
AGTTTGTGGGATATATGCTGTTAGCATTACTTCGAGAGATTTTCTTTTTTGGTTTAGAT
CTCACACTGCTAAAGAGATCAAGTGGGGCGGTGCAAAACATGATCCTGATGACAAAG
ATGACTTGAGAAAGATGCATCCGAGGTCATCCTTCAAGGCCTTTCTAGAGG????????AT
AAATTGACTTATTATACTCCTGACTATGAAACCAAAGATACTGATATCTTGGCAGCAT
TCCGAGTAACTCCTCAACCTGGAGTTCCGCCTGAGGAAGCAGGCGCTGCGGTAGCTGC
TGAATCTTCTACTGGTACATGGACAACTGTGTGGACCGATGGACTTACCAGTCTTGAT
CGTTATAAAGGGCGATGCTACGGCATCGAGCCCGTTGCTGGAGAAGAAAATCAATAT
ATTGCTTATGTAGCTTACCCCTTAGACCTTTTTGAAGAAGGTTCTGTTACTAATATGTT
TACTTCCATTGTGGGTAATGTATTTGGGTTCAAAGCCCTACGCGCTCTCCGTCTGGAG
GATTTGCGAATCCCCCCTGCTTATACAAAAACTTTCCAAGGCCCGCCTCATGGTATCC
AAGTTGAGAGAGATAAATTGAACAAGTATGGTCGCCCCCTATTGGGCTGTACTATTAA
ACCTAAATTGGGGTTATCTGCTAAGAATTACGGTAGAGCTGTTTATGAATGTCTACGC
GGTGGACTTGATTTTACCAAAGACGACGAGAACGTGAACTCCCAACCATTTATGCGTT
GGAGAGACCGTTTCTTATTTTGTGCCGAAGCACTTTATAAAGCACAGGCGGAAACAG
GTGAAATCAAAGGGCATTATTTGAATGCTACTGCAGGTACATGCGAAGAAATGATCA
AAAGGGCTGTATTTGCCAGAGAATTGGGAGCTCCTATCGTAATGCATGACTACTTAAC
AGGGGGATTCACCGCAAATACTAGCTTGGCTCATTATTGCCGAGATAATGGTTTACTT
CTGCACATCCATCGCGCAATGCATGCAGTTATTGATAGACAGAAGAATCATGGTATGC
ACTTTCGTGTACTAGCTAAAGCGTTACGCATGTCTGGTGGAGATCATATTCACGCGGG
TACCGTAGTAGGGAAACTTGAGGGGGAAAGAGATATCACTTTAGGCTTTGTTGATTTA
CTGCGTGATGATTTTGTTGAAAAAGATAGAAGCCGCGGGATTTATTTCACTCAAGACT
GGGTCTCCCTACCAGGTGTTATACCCGTGGCTTCGGGAGGTATTCACGTTTGGCATAT
GCCTGCTCTGACCGAGATCTTTGGAGATGATTCCGTACTACAATTCGGTGGGGGAACT
TTGGGGCACCCTTGGGGAAATGCACCAGGTGCCGTAGCTAATCGAGTAGCTCTAGAA
GCATGTGTACAAGCTCGTAATGAGGGACGTGATCTTGCTCGTGAGGGTAATGAAATTA
TCCGTGAGGCTAGCAAATGGAGTCCTGAACTAGCTGCTGCTTGTGAAGTGTGGAAAG
AGATTAA-
Banisteriopsis_oxyclada --

GATTAACTGCATTTTATATGTTTCGGGTTTATTTACTTACTTTTGAAGGACAATTAAAT
CTTTATTTTCAAAATTACAGTGGCAAAAAAAAACAGTGCATTCTATTCAATATCTCTAT
GGGGTAAAGAAGGATCAAAAATACTAAAAAAAAAAAATTCCTTTATTA--------------
TCTTTATTAACAATCACTAATAATGAAAAGACTTCTTTTTTTTTGAACAAAATATACAA
AATTGATGATAATGTAAGAAATATGACGCGTCCCTCTATTACTAT------
TAATCATTTTTTCACGAAAAAAAATTTTTCCCTATCCTCCTGAAGCGGATAATACTATGT
TATTTCCTATGCTTGTGTTGGTACTATTTACTTTGTTTATTGGAGCTATAGGAATTCCTT
TCAATCAAGAGGGAACTAATTTGGATATATTATCCAAACTGTTAACTCCGTCTTTAAA
TCTTTTGCATCAAAATGAAAATAATTCTTTTGAGTGGTATGAATTTATAACAAATGCT
ATTTTTTCAGTCATTATAACTTTTTTTGGAATATTTATAGCGTCTTCCTTCTATAAACCT
CTTTATTCATCGCTACAAAATTTGAACTTACTTAACTCATTTGCTA------AAAAA---------
AGGGGTCGTTGGGATAAAATAATAGGTGCGATATATGATTGGTCCTATAATCGTGGTT
ATATCGATGTTTTTTATGCAATATT---

Banisteriopsis_padifolia --

CAAATCCTTCGTTACTGGATGCAAGATCCCTCTTCTTTGCATTTATTACGACTCTTTCTT
CACGAGTATTGGAATTGGAATAGTTTTATTATTTCAACGAAATCCTTTTCCATTTTTTT
TAATTTAAAAAGGAATCTAAGATTATTGTTGTTCCTATATAATTCTCATGTATATGAAT
ACGAATCCATCTTCTTTTTTCTCCGTAACCAATCCTTTTATTTCCAATCAACATTTTCGG
GGGTGCTTCTTGAACGAATATATTTCTATGGAAAAATACAAGATTTTGTAGAACTTTT
TACTAATG------
ATTTTAAGGCTAACCTACGGTTGTTCAAAGATCCTTTCATGCATTATGTTAGATATCAA
GGAAAAGGCATTCTGGCTTCAAAAAGTACACTTCTTCTGATGAAAAAATGGAAATATT
TCTTTGTCAATTTATGTCAATTTCATTTTTATGTGTCGGTTCATCCAGAACCGATCTAT
ATGAAGTCATTATCCAACCATTCTCTTGACTTTTTGGGTTATCTTTCAAGTATACGAAA
AAATCTTTCAGTGGTACGTAGTCAAATGCTAGAAAATTCATTTCTAATAGATAATATT

ATGAAGAAACTCGATTCAAGAGTTCCAATTATTCCTTTGATTGGATCATTGTCAAAAA
CAAAATTTTGTAACGCAGTAGGGTATCCCATTAGTAAACCGAGCTGGGCGGATTCACC
CGATTCTGATATTATCGACCAATTTTTGCGTATATCCAGAAATCTTTCTCATTACTATA
GTGGATCCTCAAAAAAAAAAGAGTTTGTATCGAGTAAAATATATACTTCGACTTTCTTG
TGTTAAAACTTTGGCTCGTAAACACAAAAGTACCGTCCGGGCTTTTTTGAAAAAACTA
GGTTCAGAATTATTGGAAGAATTTTTTACGGAAGAAAAATCGATTCTTGATTTGGTCT
TCCCCACGGCTTATTCTATTTTGCGAAGGTTATACAGAGGGCGGGTTTGGTATTTGGA
TATTATTTGTATCAATGATCTAGCCAATCATGAA---
GATTAACTGCATTTTATATGTTTCGGGTTTATTTACTTACTTTTGAAGGACATTTAAAT
CTTTATTTTCAAAATTACAGTGGCAAAAAAAAACAGTGCATTCTATTCAATATCTCTAT
GGGGTAAAGAAGGATCAAAAATACTAAAAAAAAAAAATTCCTTTATTA--------------
TCTTTATTAACAATCACTAATAATGAAAAGACTTCTTTTTTTTTGAACAAAATATACCA
AATTGATGGTAATGTAAGAAATATGACGCGTCCCTCTATTACTAT------
TAATCATTTTTTCACGAAAAATATTTTTCCCTATCCCCCTGAAGCGGATAATACTATGT
TATTTCCTATGCTTGTGTTGGTACTATTTACTTTGTTTATTGGAGCTATAGGAATTCCTT
TCAATCAAGAGGGAACTAATTTGGATATATTATCCAAACTGTTAACTCCGTCTTTAAA
TCTTTTGCATCAAAATGAAAACAATTCTTTTGATTGGTATGAATTTATAACAAATGCTA
TTTTTTCAGTCATTATAACTTTTTTTGGAATATTTATAGCGTCTTCCTTCTATAAACCTC
TTTATTCATCGCTACAAAATTTGAACTTACTTAACTCATTTGCTA------AAAAA---------
AGGGGTCGTTGGGACAAAATAATAGGTGCGATATATGATTGGTCCTATAATCGTGGTT
ATATTGATGTTTTTTATGCAATATT---

Banisteriopsis_paraguariensis --

-
ATTTAAATTATGTGTCTGATTCACTAATACCTTACCCCATCCATCTAGAAAAATTGGTT
CAAATCCTTCGTTACTGGATGCAAGATCCCTCTTCTTTGCATTTATTACGACTCTTTCTT
CACGAGTATTGGAATTGGAATAGTTTTATTATTTCAACGAAATCCTTTTCCATTTTTTT
TAATTTAAAAAGGAATCTAAGATTATTGTTGTTCCTATATAATTCTCATGTATATGAAT
ACGAATCCATCTTCTTTTTTCTCCGTAACCAATCCTTTTATTTCCAATCAACATTTTTGG
GGGTGCTTCTTGAACGAATATATTTCTATGGAAAAATACAAGATTTTGTAGAACTTTT
TACTAATG------
ATTTTAAGGCTAACCTACGGTTGTTCAAGGATCCTTTCATGCATTATGTTAGATATCAA
GGAAAAGGCATTCTGGCTTCAAAAAGTACACTTCTTCTGATGAAAAAATGGAAATATT
TCTTTGTCAATTTATGTCAATTTCATTTTTATGTGTGGGTTCATCCAGAACCGATCTAT

ATGAAGTCATTATCCAACCATTCTCTTGACTTTTTGGGTTATCTTTCAAATATACGAAA
AAATCTTTCAGTGGTACGGAGTCAAATGCTAGAAAATTCATTTCTAATAGATAATATT
ATGAAGAAACTCGATTCAAGAGTTCCAATTATTCCTTTGATTGGATCATTGTCAAAAA
CAAAATTTTGTAACGCAGTAGGGCATCCCATTAGTAAACCGAGCTGGGCGGATTCACC
CGATTCTGACATTATCGACCAATTTTTGCGTATATCCAGAAATCTTTCTCATTACTATA
GCGGATCCTCAAAAAAAAAGAATTTGTATCGAGTAAAATATATACTTCGACTTTCTTG
TGTTAAAACTTTGGCTCGTAAACACAAAAGTACCGTCCGGGCTTTTTTGAAAAAACTA
GGTTCAGAATTATTGGAAGAATTTTTTACGGAGGAAAAATCAATTCTTGATTTGGTCT
TCCCCACGGCTTATTCTATTTCGCGAAGGTTATACAGAGGGCGGGTTTGGTATTTGGA
TATTATTTGTATCAATGATCTAGCCAATCATGAATAAGATTAACTGCATTTTATATGTT
TCGGGTTTATTTACTTACTTTTGAAGGACATTTAAACCTTTATTTTCAAAATTACAGTG
GCAAAAAAAACAGTGCATTCTATTCAATATCTCTATGGGGTAAAGAAGGATCAAAAA
TACTAAAAAAAAAAAATTCCTTTATTA---------------
TCTTTATTAACAATCACTAATAATGAAAAGACTTCTTTTTTTTTGAACAAAATATACAA
AATTGATGGTAATGTAAGAAATATGACGCGTCCCTCTATTACTAT------
TAATCATTTTTTCACGAAAAATATTTTTCCCTATCCCCCTGAAGCGGATAATACTATGT
TATTTCCTATGCTTGTATTGGTACTATTTACTTTGTTTATTGGAGCTATAGGAATTCCTT
TCAATCAAGAGGGAACTAATTTGGATATATTATCCAAACTGTTAACTCCGTCTTTAAA
TCTTTTGCATCAAAATGAAAATAATTCTTTTGATTGGTATGAATTTATAACAAATGCTA
TTTTTTCAGTCATTATAACTTTTTTTGGAATATTTATAGCGTCTTCCTTCTATAAACCTC
TTTATTCATCTCGACAAAATTTGAACTTACTTAACTCATTTGCTA------AAAAA---------
AGGGGTCGTTGGGATAAAATAATAGGTGTGATATATGATTGGTCCTATAATCGTGGTT
ATATCGATGTTTTTTATGCAATATT??CTTCAAGGTTTCTTTTCATGAAAAACAAGGTAC
GCATGATATGCGATTGTCTGGCCCCTCCAGTCAAGGTAATCCAAGACAAGAGATTGCC
TCAGCCGTTAAGTCTTTGTGGGTCTACATTAAGATCTCCTCATGGTTGCCATTCTCAGT
ATATGACAAATATGGGGACAATTGCATCGCTTGTGATGTCTGTGACTATCAACGAAGA
TGATGATACGATGGATGGTGATCAGCAGCAGATGACAAGAAAATTGTGGGGCTTAGT
TGTTTGTCATCATACTAGTCCTCGATTTGTTCCATTTCCTTTGAGGTATGCTTGTGAATT
TCTGATTCAAGTGTTTGGTGTTCAGATCAACAAGGAGGTAGAATTAGCTGCTCAGGTG
AGGGAAAAACATATTTTACAAATTCAGACTATGCTATGCGACATGCTCCTTAGAGATG
CTCCAGTTGCAATTATCACACAATCTCCGAATGTGATGGATCTTGTTAAGTGTGATGG
AGCTGCCTTATACTTCAAGAACAAAACTTGGTTGCTGGGAGTAACCCCTACAGAGGA
ACAAATCAGAGACATAGCCGAATGGCTGCTTGAATATCATAGCGGAAACACGGGTTT
AAGTACTGACAGCCTTATGGAAGCTGGATATCCAGGTGCTTCAGCTCTTGGTGATGCA
GTTTGTGGGATGGCTGCTATTAGCATTACTTCGAGAGATTTTCTTTTTTGGTTTAGATC
TCACACTGCCAAAGAGATCAAGTGGGGTGGTGCAAAACATGATCCTGATGACAAAGA
TGACTTAAGAAAGATGCATCCGAGGTCATCCTTCAAGGCCTTTCTAGAGG????????------
----

Banisteriopsis_parviflora ---

TTAACTGCATTTTATATGTTTCGGGTTTATTTACTTACTTTTGAAGGACATTTAAATCTT
TATTTTCAAAATTACAGTGGCAAAAAAAACAGTGCATTCTATTCAATATCTCTATGGG
GTAAAGAAGGATCCAAAATACTAAAAAAAAAAAATTCCTTTATTA---------------
TCTTTATTAACAATCACTAATAATGAAAAAGCTTCTTTTTTTTTGAACAAAATATACCA
AATTGATGGTAATGTAAGAAATATGACGCTTCCCTCTATTACTAT------
TAATCATTTTTTCACGAAAACTATTTTCCCCTATCCCCCTGAAGCGGATAATACTATGT
TATTTCCTATGCTTGTGTTGGTACTATTTACTTTGTTTATTGGAGCTATAGGAATTCCTT
TCAATCAAGAGGGAACTAATTTGGATATATTATCTAAACTGTTAACTCCGTCTTTAAA
TCTTTTGCATCAAAATGAAAAGAATTCTTTTGATTGGTATGAATTTATAACAAATGCT
ATTTTTTCAGTCATTATAACTTTTTTTGGAATATTTATAGCGTCTTCCTTCTATAAACCT
CTTTATTCATCGCTACAAAATTTGAACTTACTTAACTCATTTGCTA------AAAAA---------
AGGGGTCGTTGGGACAAAATAATAGGTGCGATATATGATTGGTCCTATAATCGTGGTT
ATATCGATGTTTTTTATGCAATATT---

ACAACTGTGTGGACCGATGGACTTACCAGTCTTGATCGTTATAAAGGGCGATGCTACG
GCATCGAGCCCGTTGCTGGAGAAGAAAATCAATATATTGCTTATGTAGCTTACCCCTT
AGACCTTTTTGAAGAAGGTTCTGTTACTAATATGTTTACTTCCATTGTGGGTAATGTAT
TTGGGTTCAAAGCCCTACGCGCTCTCCGTCTGGAGGATTTGCGAATCCCTCCTGCTTAT
ACGAAAACTTTCCAAGGCCCGCCTCATGGTATCCAAGTTGAGAGAGATAAATTGAAC
AAGTATGGTCGCCCCCTATTGGGCTGTACTATTAAACCTAAATTGGGGTTATCTGCTA
AGAATTACGGTAGAGCTGTTTATGAATGTCTACGCGGTGGACTTGATTTTACCAAAGA
CGACGAGAACGTGAACTCCCAACCATTTATGCGTTGGAGAGACCGTTTCTTATTTTGT
GCCGAAGCACTTTATAAAGCACAGGCGGAAACAGGTGAAATCAAAGGGCATTATTTG
AATGCTACTGCAGGTACATGCGAAGAAATGATCAAAAGGGCTGTATTTGCCAGAGAA
TTGGGAGCTCCTATCGTAATGCATGACTACTTAACAGGGGGATTCACCGCAAATACTA
GCTTGGCTCATTATTGCCGAGATAATGGTTTACTTCTGCACATCCATCGCGCAATGCAT
GCAGTTATTGATAGACAGAAGAATCATGGTATGCACTTTCGTGTACTAGCTAAAGGGT
TACGCATGTCTGGTGGAGATCATATTCACGCGGGTACCGTAGTAGGGAAACTTGAGG
GGGAAAGAGATATCACTTTAGGCTTTGTTGATTTACTGCGTGATGATTTTATTGAAAA
AGATAGAAGCCGCGGGATTTATTTCACTCAAGACTGGGTCTCCCTACCAGGTGTTATA
CCCGTGGCTTCGGGAGGTATTCACGTTTGGCATATGCCTGCTCTGACCGAGATCTTTG
GAGATGATTCCGTACTACAATTCGGTGGGGGAACTTTGGGGCACCCTTGGGGAAATG
CACCAGGTGCCGTAGCTAATCGAGTAGCTCTAGAAGCATGTGTACAAGCTCGTAATG
AGGGACGTGATCTTGCTCGTGAGGGTAATGAAATTATCCGTGAGGCTAGCAAATGGA
GTCCTGAACTAGCTGCTGCTTGTGAAGTGTGGAAAGAGATTAAA
Banisteriopsis_parviglandula
TGTTGGGATGGTAGGCTCCATGCTTTTTGCATCGAACTGCTAGACCGCAATCCTTTTC
AAGTCTCGAGCATTGCTTTTATGCTCGGGTTATCGAC-------
CGTGTTCCTGTATTTGATACCCATGTCGGAGGAACTAGGCTTCTG-TTCTCCCC----
AGTCCGCTCGATGCCTCTTACGGGCATGGAGCGGAGCACGTAGTT-
TGATCTGTCGAGCCTAGCATGCCTT---CTCGGCTGCAACGGTCGTCGGATCAAAGC-
TTGTGTTCTCGGATGCAGAATACTCGATGGGTCCAAGGAATGAATCCTTG--
CATTGGCCCTGTGTGGGAAATCGTCCCTCGATAAGAACGACGTCCGCCGTGCGTCGCG
ATCCGTCCGGAGTCCTCCCGGGACGAGGACGCTTCGTGCGTGCGTCGGTGTC-

CTCGAAGGAATGCTACCTGGTTGATCCTGCCAGTAGTCATATGCTTGTCTCAAAGATT
AAGCCATGCATG--

Banisteriopsis_prancei ---

-????????????????????????????????TACGCATGATATGCGATTGTCTGGCCCCTCCAGTCAA
GGTAATCCAAGACAAGAGATTGCCTCAGCCGTTAAGTCTTTGTGGGTCTACATTAAGA
TCTCCTCATGGTTGCCATTCTCAGTATATGACAAATATGGGGACAATTGCATCGCTTGT
GATGTCTGTGACTATCAACGAAGATGATGATACGATGGATGGTGATCAGCAGCAGAT
GACAAGAAAATTGTGGGGCTTAGTTGTTTGTCATCATACTAGTCCTCGATTTGTTCCGT
TTCCTTTGAGGTATGCTTGTGAATTTTTGATTCAAGTGTTTGGTGTTCAGATCAACAAG
GAGGTAGAACTAGCTGCTCAGGTGAGGGAAAAACATATTTTACAAATTCAGACTATG
CTATGCGACATGCTCCTTAGAGATGCTCCTGTTGCAATTATCACACAATCTCCGAATG
TGATGGATCTTGTTAAGTGTGATGGAGCTGCCTTATACTTCAAGAACAAAACTTGGTT
GCTGGGAGTAACCCCTACAGAGGAACAAATCAGAGACATAGCCGAATGGCTGCTCCA
ATATCATGGTGGAAACACGGGTTTAAGTACTGACAGCCTTATGGAAGCTGGATATCCA
GGTGCTTCAGCTCTTGGTGATGCAATTTGTGGGATGGCTGCTGTTAGCATTACTTCGA
GAGATTTTCTTTTTTGGTTTAGATCTCACACTGCTAAAGAGATCAAGTGGGGCGGTGC
AAAACATGATCCTGACGACAAAGATGACTTAAGAAAGATGAATCCAAGG????????????
?????????????????????---

Banisteriopsis_pulcherrima ---

ATTTAAATTATGTGTCTGATTCACTAATACCTTACCCCATCCATCTAGAAAAATTGGTT
CAAATCCTTCGTTACTGGATGCAAGATCCCTCTTCTTTGCATTTATTACGACTCTTTCTT
CACGAGTATTGGAATTGGAATAGTTTTATTATTTCAACGAAATCCTTTTCCATTCTTTT
AAATTTAAAAAGGAATCTAAGATTATTGTTGTTCCTATATAATTCTCATGTATATGAAT
ACGAATCCATCTTCTTTTTTCTCCGTAACCAATCCTTTTATTTCCAATCAACATTTTCGG
GGGTGCTTCTTGAACGAATATATTTCTATGGAAAAATACAAGATTTTGTAGAACTTTT
TACTAATG------
ATTTTAAGGCTAACCTACGGTTGTTCAAAGATCCTTTCATGCATTATGTTAGATATCAA
GGAAAAGGCATTCTGGCTTCAAAAAGTACACTTCTTCTGATGAAAAAATGGAAATATT
TCTTTGTCAATTTATGTCAATTTCATTTTTATGTGTCGGTTCATCCAGAACCGATCTAT
ATGAAGTCATTATCCAACCATTCTCTTGACTTTTTGGGTTATCTTTCAAGTATACGAAA
AAATCTTTCAGTGGTACGGAGTCAAATGCTAGAAAATTCATTTCTAATAGATAATATT
ATGAAGAAACTCGATTCAAGAGTTCCAATTATTCCTTTGATTGGATCATTGTCAAAAA
CAAAATTTTGTAACGCAGTAGGGTATCCCATTAGTAAACCGAGCTGGGCGGATTCACC
CGATTCTGATATTATCGACCAATTTTTGCGTATATCCAGAAATCTTTCTCATTACTATA
GTGGATCCTCAAAAAAAAAGAGTTTGTATCGAGTAAAATATATACTTCGACTTTCTTG
TGTTAAAACTTTGGCTCGTAAACACAAAAGTACCGTCCGGGCTTTTTTGAAAAAACTA

GGTTCAGAATTATTGGAAGAATTTTTTACGGAGGAAAAATCGATTCTTGATTTGGTCT
TCCCCACGGCTTATTCTATTTTGCGAAGGTTATACAGAGGGCGGGTTTGGTATTTGGA
TATTATTTGTATCAATGATCTAGCCAATCATGAA---
GATTAACTGCATTTTATATGTTTCGGGTTTATTTACTTACTTTTGAAGGACATTTAAAT
CTTTATTTTCAAAATTACAGTGGCAAAAAAAACAGTGCATTCTATTCAATATCTCTAT
GGGGTAAAGAAGGATCAAAAATACTAAAAAAAAAAAATTCCTTTATTA--------------
TCTTTATTAACAATCACTAATAATGAAAAGACTTCTTTTTTTTTGAACAAAATATACCA
AATTGATGGTAATGTAAGAAATATGACGCGTCCCTCTATTACTAT------
TAATCATTTTTTCACGAAAAATATTTTTCCCTATCCCCCTGAAGCGGATAATACTATGT
TATTTCCTATGCTTGTGTTGGTACTATTTACCTTGTTTATTGGAGCTATAGGAATTCCTT
TCAATCAAGAGGGAACTAATTTGGATATATTATCCAAACTGTTAACTCCGTCTTTAAA
TCTTTTGCATCAAAATGAAAACAATTCTTTTGATTGGTATGAATTTATAACAAATGCTA
TTTTTTCAGTCATTATAACTTTTTTTGGAATATTTATAGCGTCTTCCTTCTATAAACCTC
TTTATTCATCGCTACAAAATTTGAACTTACTTAACTCATTTGCTA------AAAAA---------
AGGGGTCGTTGGGACAAAATAATAGGTGCGATATATGATTGGTCCTATAATCGTGGTT
ATATCGATGTTTTTTATGCAATATT--

Banisteriopsis_pulchra --

ATTTAAATTATGTGTCTGATTCACTAATACCTTACCCCATCCATCTAGAAAAATTGGTT
CAAATCCTTCGTTACTGGATGCAAGATCCCTCTTCTTTGCATTTATTACGACTCTTTCTT
CACGAGTATTGGAATTGGAATAGTTTTATTATTTCAACGAAATCCTTTTCCATTTTTTT
TAATTTAAAAAGGAATCTAAGATTATTGTTGTTCCTATATAATTCTCATGTATATGAAT
ACGAATCCATCTTCTTTTTTCTCCGTAACCAATCCTTTTATTTCCAATCAACATTTTCGG
GGGTGTTTCTTGAACGAATATATTTCTATGGAAAAATACAAGATTTTGTAGAACTTTTT
ACTAATG------
ATTTTAAGGCTAACCTACGGTTGTTCAAGGATCCTTTCATGCATTATGTTAGATATCAA
GGAAAAGGCATTCTGGCTTCAAAAAGTACACTTCTTCTGATGAAAAAATGGAAATATT
TCTTTGTCAATTTATGTCAATTTCATTTTTATGTGTGGGTTCATCCAGAACCGATCTAT
ATGAAGTCATTATCCAACCATTCTCTTGACTTTTTGGGTTATCTTTCAAATATACGAAA
AAATCTTTCAGTGGTACGGAATCAAATGCTAGAAAATTCATTTCTAATAGATAATATT
ATGAAGAAACTCGATTCAAGAGTTCCAATTATTCCTTTGATTGGATCATTGTCAAAAA
CAAAATTTTGTAACGCAGTAGGGCATCCCATTAGTAAACCGAGCTGGGCGGATTCACC
CGATTCTGACATTATCGACCAATTTTTGCGTATATCCAGAAATCTTTCTCATTACTATA
GCGGATCCTCAAAAAAAAAGAATTTGTATCGAGTAAAATATATACTTCGACTTTCTTG

TGTTAAAACTTTGGCTCGTAAACACAAAAGTACCGTCCGGGCTTTTTGAAAAAACTA
GGTTCAGAATTATTGGAAGAATTTTTTACGGAGGAAAAATCAATTCTTGATTTGGTCT
TCCCCACGGCTTATTCTATTTCGCGAAGGTTATACAGAGGGCGGGTTTGGTATTTGGA
TATTATTTGTATCAATGATCTAGCCAATCATGAATAAGATTAACTGCATTTTATATGTT
TCGGGTTTATTTACTTACTTTTGAAGGACATTTAAATCTTTATTTTCAAAATTACAGTG
GCAAAAAAAAACAGTGCATTCTATTCAATATCTCTATGGGGTAAAGAAGGATCAAAAA
TACTAAAAAAAAAAAATTCCTTTATTA---------------
TCTTTATTAACAATCACTAATAATGAAAAGACTTCTTTTTTTTTGAACAAAATATACAA
AATTGATGGTACTGTAAGAAATATGACGCGTCCCTCTATTACTAT------
TAATCATTTTTTCACGAAAAATATTTTTCCCTATCCTCCTGAAGCGGATAATACTATGT
TATTTCCTATGCTTGTGTTGGTACTATTTACTTTGTTTATTGGAGCTATAGGAATTCCTT
TCAATCAAGAGGGAACTAATTTGGATATATTATCCAAACTGTTAACTCCGTCTTTAAA
TCTTTTGCATCAAAATGAAAATAATTCTTTTGATTGGTATGAATTTATAACAAATGCTA
TTTTTTCAGTCATTATAACTTTTTTTGGAATATTTATAGCGTCTTCGTTCTATAAACCTC
TTTATTCATCGCTACAAAATTTGAACTTACTTAACTCATTTGCTA------AAAAA---------
AGGGGTCGTTGGGATAAAATAATAGGTGCGATATATGATTGGTCCTATAATCGTGGTT
ATATCGATGTTTTTTATGCAATATT?????CAAGGTTTCTCTTCATGAAAAACAAGGTAC
GCATGATATGCGATTGTCTGGCCCCTCCAGTCAAGGTAATCCAAGACGAGAGATTGCC
TCAGCCGTTAAGTCTTTGTGGGTCTACATTAAGATCTCCTCATGGTTGCCATTCTCAGT
ATATGACAAATATGGGGACAATTGCATCCCTTGTGATGTCTGTGACTATCAACGAAGA
TGATGATACAATGGATGGTGACCAGCAGCAGATGACAAGAAAATTGTGGGGCTTAGT
TGTTTGTCATCATACTAGTCCTCGATTTGTTCCGTTTCCTTTGAGGTATGCTTGTGAATT
TTTGATTCAAGTGTTTGGTGTTCAGATCAACAAGGAGGTAGAATTAGCTGCTCAGGTG
AGGGAAAAACATATTTTACAAATTCAGACTATGCTATGCGACATGCTCCTTAGAGATG
CTCCCGTTGCAATTATCACACAATCTCCGAATGTGATGGATCTTGTTAAGTGTGATGG
AGCTGCCTTATACTTCAAGAACAAAACTTGGTTGCTGGGAGTAACGCCTACAGAGGA
ACAAATCAGAGACATGGTCGAATGGCTGCTCGAATATCATAGCGGAAACACGGGTTT
AAGTACTGACAGCCTTATGGAAGCTGGATATCCAGGTGCTTCGGCTCTTGGTGATGCA
GTTTGTGGGATGGCTGCTGTTAGCATTACTTCGAGAGATTTTCTTTTTTGGTTTAGATC
TCACACTGCTAAAGAGATAAAATGGGGTGGTGCAAAACATGATCCTGATGACAAAGA
TGACTTAAGAAAGATGCATCCGAGGTCATCCTTCAAGGCCTTCCTAGAGGTGGTAAAA

Banisteriopsis_schizoptera --

CTTACTTTTGAAGGACATTTAAATCTTTATTTTCAAAATTACAGTGGCAAAAAAAACA
GTGCATTCTATTCAATATCTCTATGGGGTAAAGAAGGATCAAAAATACTAAAAAAAA
AAATTCCTTTATTA---------------
TCTTTATTAACAATCCCTAATAATGAAAAGACTTCTTTTTTTTTGAACAAAATATACAA
AATTGATGGTAATGTAAGAAATATGACGCGTCCCTCTATTACTAT------
TAATCATTTTTTCACGAAAAATATTTTTCCCTATCCCCCTGAAGCGGATAATACTATGT
TATTTCCTATGCTTGTATTGGTACTATTTACTTTGTTTATTGGAGCTATAGGAATTCCTT
TCAATCAAGAGGGAACTAATTTGGATATATTATCCAAACTGTTAACTCCGTCTTTAAA
TCTTTTGCATCAAAATGAAAATAATTCTTTTGATTGGTATGAATTTATAACAAATGCTA
TTTTTTCAGTCATTATAACTTTTTTTGGAATATTTATAGCGTCTTCCTTCTATAAACCTC
TTTATTCATCTCTACAAAATTTGAACTTACTTAACTCATTTGCTA------AAAAA---------
AGGGGTCGTTGGGATAAAATAATAGGTGCGATATATGATTGGTCCTATAATCGTGGTT
ATATCGATGTTTTTTATGCAATATT--

Banisteriopsis_schwannioides ---

-
ATTTAAATTATGTGTCTGATTCACTAATACCTTACCCCATCCATCTAGAAAAATTGGTT
CAAATCCTTCGTTACTGGATGCAAGATCCCTCTTCTTTGCATTTATTACGACTCTTTCTT
CACGAGTATTGGAATTGGAATAGTTTTCTTATTTCAACGAAATCCTTTTCCATTTTTTT
GAATTTAAAAAGGAATCTAAGATTATTGTTGTTCCTATATAATTCTCATGTATATGAAT
ACGAATCCATCTTCTTTTTTCTCCGTAACCAATCCTTTTATTTCCAATCAACATTTTCGG
GGGTGTTTCTTGAACGAATATATTTCTATGGAAAAATACAAGATTTTGTAGAACTTTTT
ACTAATG------
ATTTTAAGGCTAACCTACGGTTGTTCAAGGATCCTTTCATGCATTATGTTAGATATCAA
GGAAAAGGCATTCTGGCTTCAAAAAGTACACTTCTTCTGATGAAAAAATGGAAATATT
TCTTTGTCAATTTATGTCAATTTCATTTTTATGTGTGGGTTCATCCAGAACCGATCTAT
ATGAAGTCATTATCCAACCATTCTCTTGACTTTTTGGGTTATCTTTCAAATATACGAAA
AAATCTTTCAGTGGTACGGAGTCAAATGCTAGAAAATTCATTTCTAATAGATAATATT
ATGAAGAAACTCGATTCAAGAGTTCCAATTATTCCTTTGATTGGATCATTGTCAAAAA
CAAAATTTTGTAACGCAGTAGGGCATCCCATTAGTAAACCGAGCTGGGCGGATTTACC
CGATTCTGACATTATCGACCAATTTTTGCGTATATCCAGAAATCTTTCTCATTACTATA

GCGGATCCTCAAAAAAAAAGAATTTGTATCGAGTAAAATATATACTTCGACTTTCTTG
TGTTAAAACTTTGGCTCGTAAACACAAAAGTACCGTCCGGGCTTTTTTGAAAAAACTA
GGTTCAGAATTATTGGAAGAATTTTTTACGGAGGAAAAATCAATTCTTGATTTGGTCT
TCCCCACGGCTTATTCTATTTCGCGAAGGTTATACAGAGGGCGGGTTTGGTATTTGGA
TATTATTTGTATCAATGATCTAGCCAATCATGAATAAGATTAACTGCATTTTATATGTT
TCGGGTTTATTTACTTACTTTTGAAGGACATTTAAATCTTTATTTTCAAAATTACAGTG
GCAAAAAAAAACAGTGCATTCTATTCAATATCTCTATGGGGTAAAGAAGGATCAAAAA
TACTAAAAAAAAAAAATTCCTTTATTA---------------
TCTTTATTAACAATCACTAATAATGAAAAGACTTCTTTTTTTTTGAACAAAATATACAA
AATTGATGGTACTGTAAGAAATATGACGCGTCCCTCTATTACTAT------
TAATCATTTTTTCACGAAAAATATTTTTCCCTATCCTCCTGAAGCGGATAATACTATGT
TATTTCCTGTGCTTGTGTTGGTACTATTTACTTTGTTTATTGGAGCTATAGGAATTCCTT
TCAATCAAGAGGGAACTAATTTGGATATATTATCCAAACTGTTAACTCCGTCTTTAAA
TCTTTTGCATCAAAATGAAAATAATTCTTTTGATTGGTATGAATTTATAACAAATGCTA
TTTTTTCAGTCATTATAACTTTTTTTGGAATATTTATAGCGTCTTCCTTCTATAAACCTC
TTTATTCATCGCTACAAAATTTGAACTTACTTAACTCATTTGCTA------AAAAA---------
AGGGGTCGTTGGGATAAAATAATAGGTGCGATATATGATTGGTCCTATAATCGTGGTT
ATATCGATGTTTTTATGCAATATT?????????????????????????????????????GCATGATATGC
GATTGTCTGGCCCCTCCAGTCAAGGTAATCCAAGACGAGAGATTGCCTCAGCCGTTAA
GTCTTTGTGGGTCTACATTAAGATCTCCTCATGGTTGCCATTCTCAGTATATGACAAAT
ATGGGGACAATTGCATCCCTTGTGATGTCTGTGACTATCAACGAAGATGATGATACAA
TGGATGGTGACCAGCAGCAGATGACAAGAAAATTGTGGGGCTTAGTTGTTTGTCATCA
TACTAGTCCTCGATTTGTTCCGTTTCCTTTGAGGTATGCTTGTGAATTTTTGATTCAAGT
GTTTGGTGTTCAGATCAACAAGGAGGTAGAATTAGCTGCTCAGGTGAGGGAAAAACA
TATTTTACAAATTCAGACTATGCTATGTGACATGCTCCTCAGAGATGCTCCAGTTGCA
ATTATCACACAATCTCCGAATGTGATGGATCTTGTTAAGTGTGATGGAGCTGCCTTAT
ACTTCAAGAACAAAACTTGGTTGCTAGGAGTAACGCCTACAGAGGAACAAATCAGAG
ACATAGTCGAATGGCTGCTTGAATATCATAGCGGAAACACGGGTTTAAGTACTGACA
GCCTTATGGAAGCTGGATATCCAGGTGCTTCGGCTCTTGGTGATGCAGTTTGTGGGAT
GGCTGCTGTTAGCATTACTTCGAGAGATTTTCTTTTTTGGTTTAGATCTCACACTGCTA
AAGAGATAAAGTGGGGTGGTGCAAAACATGATCCTGATGACAAAGATGACTTAAGAA
AGATGCATCCGAGGTCATCCTTCAAGGCCTTCCTAGAGGTGGTAAAAATAAATTGACT
TATTATACTCCTGACTATGAAACCAAAGATACTGATATCTTGGCAGCATTCCGAGTAA
CTCCTCAACCTGGAGTTCCGCCTGAGGAAGCAGGCGCTGCGGTAGCTGCTGAATCTTC
TACTGGTACATGGACAACTGTGTGGACCGATGGACTTACCAGTCTTGATCGTTATAAA
GGGCGATGCTACGGCATCGAGCCCGTTGCTGGAGAAGAAAATCAATATATTGCTTAT
GTAGCTTACCCCTTAGACCTTTTTGAAGAAGGTTCTGTTACTAACATGTTTACTTCCAT
TGTGGGTAATGTATTTGGGTTCAAAGCCCTACGCGCTCTCCGTCTGGAGGATTTGCGA
ATCCCTCCTGCTTATACGAAAACTTTCCAAGGCCCGCCTCATGGTATCCAAGTTGAGA
GAGATAAATTGAACAAGTATGGTCGCCCCCTATTGGGCTGTACTATTAAACCTAAATT
GGGGTTATCTGCTAAGAATTACGGTAGAGCTGTTTATGAATGTCTACGCGGTGGACTT
GATTTTACCAAAGACGACGAGAACGTGAACTCCCAACCATTTATGCGTTGGAGAGAC
CGTTTCTTATTTTGTGCCGAAGCAATTTATAAAGCACAGGCGGAAACAGGTGAAATCA
AAGGGCATTATTTGAATGCTACTGCAGGTACATGCGAAGAAATGATCAAAAGGGCTG
TATTTGCCAGAGAATTGGGAGCTCCTATCGTAATGCATGACTACTTAACAGGGGGATT
CACCGCAAATACTAGCTTGGCTCATTATTGCCGAGATAATGGTTTACTTCTGCACATC
CATCGCGCAATGCATGCAGTTATTGATAGACAGAAGAATCATGGTATGCACTTTCGTG
TACTAGCTAAAGCGTTACGCATGTCTGGTGGAGATCATGTTCACGCGGGTACCGTAGT
AGGGAAACTTGAGGGGGAAAGAGATATCACTTTAGGCTTTGTTGATTTACTGCGTGAT
GATTTTGTTGAAAAAGATAGAAGCCGCGGGGTTTATTTCACTCAAGACTGGGTCTCCC
TACCAGGTGTTATACCCGTGGCTTCGGGAGGTATTCACGTTTGGCATATGCCTGCTCT
GACCGAGATCTTTGGAGATGATTCCGTACTACAATTCGGTGGGGGAACTTTGGGGCAC
CCTTGGGGAAATGCACCAGGTGCCGTAGCTAATCGAGTAGCTCTGGAAGCATGTGTA
CAAGCTCGTAATGAGGGACGTGATCTTGCTCGTGAGGGTAATGAAATTATCCGTGAG
GCTAGCAAATGGAGTCCTGAACTAGCTGCTGCTTGTGAAGTGTGGAAAGAGATTAA-
Banisteriopsis_scutellata --

GATTAACTGCATTTTATATGTTTCGGGTTTATTTACTTACTTTTGAAGGACATTTAAAT
CTTTATTTTCAAAATTACAGTGGCAAAAAAAACAGTGCATTCTATTCAATATCTCTAT
GGGGTAAAGAAGGATCAAAAATACTAAAAAAAAAAAATTCCTTTATTA--------------
TCTTTATTAACAATCACTAATAATGAAAAAGCTTCTTTTTTTTTGAACAAAATATACCA
AATTGATGGTAATGTAAGAAATATGACGCTTCCCTCTATTACTAT------
TAATCATTTTTTCACGAAAACTCTTTTTCCCTATCCCCCTGAAGCGGATAATACTATGT
TATTTCCTATGCTTGTGTTGGTACTATTTACTTTGTTTATTGGAGCTATAGGAATTCCTT
TCAATCAAGAGGGAACTAATTTGGATATATTATCTAAACTGTTAACTCCGTCTTTAAA
TCTTTTGCATCAAAATGAAAATAATTCTTTTGATTGGTATGAATTTATAACAAATGCTA
TTTTTTCAGTCATTATAACTTTTTTTGGAATATTTATAGCGTCTTCCTTCTATAAACCTC
TTTATTCATCGCTACAAAATTTGAACTTACTTAACTCATTTGCTA------AAAAA---------
AGGGGTCGTTGGGACAAAATAATAGGTGCGATATATGATTGGTCCTATAATCGTGGTT
ATATCGATGTTTTTTATGCAATATT---

ATAAATTGACTTATTATACTCCTGACTATGAAACCAAAGATACTGATATCTTGGCAGC
ATTCCGAGTAACTCCTCAACCTGGAGTTCCGCCTGAGGAAGCAGGCGCTGCGGTAGCT
GCTGAATCTTCTACTGGTACATGGACAACTGTGTGGACCGATGGACTTACCAGTCTTG
ATCGTTATAAAGGGCGATGCTACGGCATCGAGCCCGTTGCTGGAGAAGAAAATCAAT
ATATTGCTTATGTAGCTTACCCCTTAGACCTTTTTGAAGAAGGTTCTGTTACTAATATG
TTTACTTCCATTGTGGGTAATGTATTTGGGTTCAAAGCCCTACGCGCTCTCCGTCTGGA
GGATTTGCGAATCCCTCCTGCTTATACGAAAACTTTCCAAGGCCCGCCTCATGGTATC
CAAGTTGAGAGAGATAAATTGAACAAGTATGGTCGCCCCCTATTGGGCTGTACTATTA
AACCTAAATTGGGGTTATCTGCTAAGAATTACGGTAGAGCTGTTTATGAATGTCTACG
CGGTGGACTTGATTTTACCAAAGACGACGAGAACGTGAACTCCCAACCATTTATGCGT
TGGAGAGACCGTTTCTTATTTTGTGCCGAAGCACTTTATAAAGCACAGGCGGAAACAG
GTGAAATCAAAGGGCATTATTTGAATGCTACTGCAGGTACATGCGAAGAAATGATCA
AAAGAGCTGTATTTGCCAGAGAATTGGGAGCTCCTATCGTAATGCATGACTACTTAAC
AGGGGGATTCACCGCAAATACTAGCTTGGCTCATTATTGCCGAGATAATGGTTTACTT
CTGCACATCCATCGCGCAATGCATGCAGTTATTGATAGACAGAAGAATCATGGTATGC
ACTTTCGTGTACTAGCTAAAGGGTTACGCATGTCTGGTGGAGATCATATTCACGCGGG
TACCGTAGTAGGGAAACTTGAGGGGGAAAGAGATATCACTTTAGGCTTTGTTGATTTA
CTGCGTGATGATTTTGTTGAAAAAGATAGAAGCCGCGGGATTTATTTCACTCAAGACT
GGGTCTCCCTACCAGGTGTTATACCCGTGGCTTCGGGAGGTATTCACGTTTGGCATAT
GCCTGCTCTGACCGAGATCTTTGGAGATGATTCCGTACTACAATTCGGTGGGGGAACT
TTGGGGCACCCTTGGGGAAATGCACCAGGTGCCGTAGCTAATCGAGTAGCTCTAGAA
GCATGTGTACAAGCTCGTAATGAGGGACGTGATCTTGCTCGTGAGGGTAATGAAATTA
TCCGTGAGGCTAGCAAATGGAGTCCTGAACTAGCTGCTGCTTGTGAAGTGTGGAAAG
AGATTAAA

Banisteriopsis_sellowiana --

GATTAACTGCATTTTATATGTTTCGGGTTTATTTACTTACTTTTGAAGGACATTTAAAT
CTTTATTTTCAAAATTACAGTGGCAAAAAAAACAGTGCATTCTATTCAATATCTCTAT
GGGGTAAAGAAGGATCAAAAATACTAAAAAAAAAAAATTCCTTTATTA---------------
TCTTTATTAACAATCACTAATAATGAAAAGACTTCTTTTTTTTTGAACAAAATAGACCA
AATTGATGGTAATGTAAGAAATATGACGCGTCCCTCTATTACTAT------
TAATCATTTTTTCACGAAAAATATTTTTCCCTATCCCCCTGAAGCGGATAATACTATGT
TATTTCCTATGCTTGTGTTGGTACTATTTACTTTGTTTATTGGAGTTATAGGAATTCCTT
TCAATCAAGAGGGAACTAATTTGGATATATTATCCAAACTGTTAACTCCGTCTTTAAA
TCTTTTGCATCAAAATGAAAATAATTCTTTTGATTGGTATGAATTTATAACAAATGCTA
TTTTTTCAGTCATTATAACTTTTTTTGGAATATTTATAGCGTCTTCCTTCTATAAACCTC
TTTATTCATCGCTACAAAATTTGAACTTACTTAACTCATTTGCTA------AAAAA---------
AGGGGTCGTTGGGACAAAATAATAGGTGCGATATATGATTGGTCCTATAATCGTGGTT
ATATCGATGTTTTTTATGCAATATT????????????????????????????AGGTACGCATGATAT
GCGATTGTCTGGCCCCTCCAGTCAAGGTAATCCAAGACGAGAGATTGCCTCAGCCGTT
AAGTCTTTGTGGGTCTACATTAAGATCTCCTCATGGTTGCCATTCTCAGTATATGACAA
ATATGGGGACAATTGCATCGCTTGTGATGTCTGTGACTATCAACGAAGATGATGATAC
GATGGATGGTGATCAGCAGCAGATGACAAGAAAATTGTGGGGCTTAGTTGTTTGTCAT
CATACTAGTCCTCGATTTGTTCCGTTTCCTTTGAGGTATGCTTGTGAATTTTTGATTCA
AGTGTTTGGTGTTCAGATCAACAAGGAGGTAGAATTAGCTGCTCAGGTGAGGGAAAA
ACATATTTTACAAATTCAGACTATGCTATGCGACATGCTCCTTAGAGATGCTCCCGTT
GCAATTATCACACAATCTCCGAATGTGATGGATCTTGTTAAGTGTGATGGAGCTGCCT
TATACTTCAAGAACAAAACTTGGTTGCTGGGAGTAACCCCTACAGAGGAACAAATCA
GAGACATAGCCGAATGGCTGCTCGAATATCATAGTGGAAACACGGGTTTAAGTACTG
ACAGCCTTATGGAAGCTGGATATCCAGGTGCTTCAGCTCTTGGTGATTCAGTTTGTGG
GATATATGCTGTTAGCATTACTTCGAGAGATTTTCTTTTTTGGTTTAGATCTCACACTG
CTAAAGAGATCAAGTGGGGCGGTGCAAAACATGATCCTGATGACAAAGATGACTTGA
GAAAGATGCATCCGAGGTCATCCTTCAAGGCC?????????????????----------------------------
----

--

Banisteriopsis_stellaris
TGTTGGGATGGTAGGCTCCGTGCTTTTTGCATCGAACTGCTAGACCGCAATCCTTTTC
AAGCCTCAAGCATTGCTTT-ATGCTTGTGTTATCGAC-------
CGTGTTCCTGTATTTGATACCCATGTCGGAGGAACTAGGCTTCTG-TTCTCCCC----
ATTCCACTCGATGCCTCTTGGGGGCATAGAGCGGAGCACGTGGTT-
TGATCTGTCGAGCCTAGCATGCCTG---TCCGGTTGCAACGGTTGACGGATCAAAGC-
TTGTGTTCTCGGATGCAGAATGTTCGATGGGTGCAAGGATCGAATCCTTG--
CATTTGCCCTGTGTGGGAAATCGTCCCTCGATAAGAACGACGTCCGTCGTGCGCTGCG
ATCCATCCGATGTCCTCTCGGACCGAGGATGCTTCGTGCGTGCGTCGGTGTC-
CTCGAAGGAATGCTACCTGGTTGATCCTGCCAGTAGTCATATGCTTGTCTCAAAGATT
AAGCCATGCATGCCTGCCCAGCAGAACGACCCGCGAACCG-
TTGTCAAAGCGCGTCGAGGAGCGGGCCGGGCCGACGGTCCAGCCCCCTTCCCGACGC
AGCCCGCCGCGCGGCCTC-GGCCGCCTGCGGGAACAACACCCA-
CCCCGGCGCAGAACGCGCCAAGGAAAAC-AAACCAAGGAGGGATCTTCTC--
GTGCTCCCGAACACGGGTAGCGCGGGA-AGGTTCCGCCAAACGTCG-
AACAAAAACGACTCTCGGCAACGGATATCTCGGCTCTCGCATCGATGAAGAACGTAG
CAAAATGCGATACTTGGTGTGAATTGCAGAATCCCGTGAACCATCGAGTCTTTGAACG
CAAGTTGCGCCCGAAGCCATTCGGTCGAGGGCACGTCTGCCTGGGTGTCACACAACGT
CGTCCACAAACCCGACCCT-CTCTCCCACG---GGGGGGAGT-----
CGGGAGGGACGGAAGCTGGTCTCCCGTGGCGATCAGCCGCGGTTGGCCGAATCACGA
GTCCGGGCGAGGAAAGCCGCAACAACCGGTGGTTGGAAC-
ATCCTCGATCGATGCTTGTTGCGGCCTTTTC-
CGCGCGGAGACGGGCTCCAGACCCGCGGCG-
CTCCGGCGCCTTCGATCGCGACCCCAGGTCAGGCGGGATTATTTAAATTATGTGTCTG
ATTCACTAATACCTTACCCCATCCATCTAGAAAAATTGGTTCAAATCCTTCGTTACTGG
ATGCAAGATCCCTCTTCTTTGCATTTATTACGACTCTTTCTTCACGAGTATTGGAATTG
GAATAGTTTTATTATTTCAACGAAATCCTTTTCCATTTTTTTTAATTTAAAAAGGAATC
TAAGATTATTGTTGTTCCTATATAATTCTCATGTATATGAATACGAATCCATCTTCTTT
TTTCTCCGTAACCAATCCTTTTATTTCCAATCAACATTTTCGGGGGTGCTTCTTGAACG
AATATATTTCTATGGAAAAATACAAGATTTTGTAGAACTTTTTACTAATG------
ATTTTAAGGCTAACCTACGGTTGTTCAAGGATCCTTTCATGCATTATGTTAGATATCAA
GGAAAAGGCATTCTGGCTTCAAAAAGTACACTTCTTCTGATGAAAAAATGGAAATATT
TCTTTGTCAATTTATGTCAATTTCATTTTTATGTGTGGGTTCATCCAGAACCGATCTAT
ATGAAGTCATTATCCAACCATTCTCTTGACTTTTTGGGTTATCTTTCAAATATACGAAA
AAATCTTTCAGTGGTACGGAGTCAAATGCTAGAAAATTCATTTCTAATAGATAATATT
ATGAAGAAACTCGATTCAAGAGTTCCAATTATTCCTTTGATTGGATCATTGTCAAAAA
CAAAATTTTGTAACGCAGTAGGGCATCCCATTAGTAAACCGAGCTGGGCGGATTCACC
CGATTCTGACATTATCGACCAATTTTTGCGTATATCCAGAAATCTTTCTCATTACTATA
GCGGATCCTCAAAAAAAAAGAATTTGTATCGAGTAAAATATATACTTCGACTTTCTTG
TGTTAAAACTTTGGCTCGTAAACACAAAAGTACCGTCCGGGCTTTTTTGAAAAAACTA
GGTTCAGAATTATTGGAAGAATTTTTTACGGAGGAAAAATCAATTCTTGATTTGGTCT
TCCCCACGGCTTATTCTATTTCGCGAAGGTTATACAGAGGGCGGGTTTGGTATTTGGA
TATTATTTGTATCAATGATCTAGCCAATCATGAA---
GATTAACTGCATTTTATATGTTTCGGGTTTATTTACTTACTTTTGAAGGACATTTAAAT
CTTTATTTTCAAAATTACAGTGGCAAAAAAAAACAGTGCATTCTATTCAATATCTCTAT
GGGGTAAAGAAGGATCAAAAATACTAAAAAAAAAAAATTCCTTTATTA--------------
TCTTTATTAACAATCACTAATAATGAAAAGACTTCTTTTTTTTTGAACAAAATATACAA
AATTGATGGTAATGTAAGAAATATGACGCGTCCCTCTATTACTAT------
TAATCATTTTTTCACGAAAAATATTTTTCCCTATCCCCCTGAAGCGGATAATACTATGT
TATTTCCTATGCTTGTATTGGTACTATTTACTTTGTTTATTGGAGCTATAGGAATTCCTT
TCAATCAAGAGGGAACTAATTTGGATATATTATCCAAACTGTTAACTCCGTCTTTAAA
TCTTTTGCATCAAAATGAAAATAATTCTTTTGATTGGTATGAATTTATAACAAATGCTA
TTTTTTCAGTCATTATAACTTTTTTTGGAATATTTATAGCGTCTTCCTTCTATAAACCTC
TTTATTCATCTCTACAAAATTTGAACTTACTTAACTCATTTGCTA------AAAAA---------
AGGGGTCGTTGGGATAAAATAATAGGTGCGATATATGATTGGTCCTATAATCGTGGTT
ATATCGATGTTTTTTATGCAATATT--

ATAAATTGACTTATTATACTCCTGACTATGAAACCAAAGATACTGATATCTTGGCAGC
ATTCCGAGTAACTCCTCAACCTGGAGTTCCGCCTGAGGAAGCAGGCGCTGCGGTAGCT
GCTGAATCTTCTACTGGTACATGGACAACTGTGTGGACCGATGGACTTACCAGTCTTG
ATCGTTATAAAGGGCGATGCTACGGCATCGAGCCCGTTGCTGGAGAAGAAAATCAAT
ATATTGCTTATGTAGCTTACCCCTTAGACCTTTTTGAAGAAGGTTCTGTTACTAACATG
TTTACTTCCATTGTGGGTAATGTATTTGGGTTCAAAGCCCTACGCGCTCTCCGTCTGGA
GGATTTGCGAATCCCTCCTGCTTATACGAAAACTTTCCAAGGCCCGCCTCATGGTATC
CAAGTTGAGAGAGATAAATTGAACAAGTATGGTCGCCCCCTATTGGGCTGTACTATTA
AACCTAAATTGGGGTTATCTGCTAAGAATTACGGTAGAGCTGTTTATGAATGTCTACG
CGGTGGACTTGATTTTACCAAAGACGACGAGAACGTGAACTCCCAACCATTTATGCGT
TGGAGAGACCGTTTCTTATTTTGTGCCGAAGCACTTTATAAAGCACAGGCGGAAACAG
GTGAAATCAAAGGGCATTATTTGAATGCTACTGCAGGTACATGCGAAGAAATGATCA
AAAGGGCTGTATTTGCCAGAGAATTGGGAGCTCCTATCGTAATGCATGACTACTTAAC
AGGGGGATTCACCGCAAATACTAGCTTGGCTCATTATTGCCGAGATAATGGTTTACTT
CTGCACATCCATCGCGCAATGCATGCAGTTATTGATAGACAGAAGAATCATGGTATGC
ACTTTCGTGTACTAGCTAAAGCGTTACGCATGTCTGGTGGAGATCATATTCACGCGGG
TACCGTAGTAGGGAAACTTGAGGGGGAAAGAGATATCACTTTAGGCTTTGTTGATTTA
CTGCGTGATGATTTTGTTGAAAAAGATAGAAGCCGCGGGATTTATTTCACTCAAGACT
GGGTCTCCCTACCAGGTGTTATACCCGTGGCTTCGGGAGGTATTCACGTTTGGCATAT
GCCTGCTCTGACCGAGATCTTTGGAGATGATTCCGTACTACAATTCGGTGGGGGAACT
TTGGGGCACCCTTGGGGAAATGCACCAGGTGCCGTAGCTAATCGAGTAGCTCTAGAA
GCATGTGTACAAGCTCGTAATGAGGGACGTGATCTTGCTCGTGAGGGTAATGAAATTA
TCCGTGAGGCTAGCAAATGGAGTCCTGAACTAGCTGCTGCTTGTGAAGTGTGGAAAG
AGATTAAA
Banisteriopsis_variabilis
TGTTGGGATGGTAGGCTCCGTGCTTTTTGCATCGAACTGCTAGACCGCAATCCTTTTC
AAGTCTCGAGCATTGCTTTTATGCTCGGGTTATCGAC-------
CGTGTTCCTGTATTTGATACCCATGTCGGAGGAACTAGGCTTCTG-TTCTCCCC----
AGTCCGCTCGATGCCTCTTACGGGCATCGAGCGGAGCACGTAGTT-
CGATCTGTCGAGCCTAGCACGCCTT---CTCGGCTGCAACGGTCGTCGGATCAAAGC-
TCGTGTTCTCGGATGCAGAATACTCGATGGGTCCAAGGATTGAATCCTTG--
CATTGGCCCTGTGTGGGAAATCGTCCCTCGATAAGAACGACGTCCGCCGTGCGTCGCG
ATCCGTCCGATGTCCTCTCGGGACGAGGACGCTTCGTGCGTGCGTCGGTGTC-
CTCGAAGGAATGCTACCTGGTTGATCCTGCCAGTAGTCATATGCTTGTCTCAAAGATT
AAGCCATGCTGGCCGGCCCAGCAGAACGACCCGCGAACCG-
TCGTCACAGCGCGTCGGGGGGAGGGC------------
CCCGCCCACATCCAGGCGCAGCCCGCCGCGCGGCCTC-
GGCCGCCGGTGGGGACAAAACCCACCCCCGGCGCAAAACGCGCCAAGGAAAAC-
AAACTGAGGAGGAACGTCCCCGTGTGCCGCCGGTCCTGGGGAGCACGCGG-
ACGTCCCGCCGAACGTAAAAGAAAAAACGACTCTCGGCAACGGATATCTCGGCTCTC
GCATCGATGAAAAACTTAGCAAAATGCGATACTTGGGGTGAATTGCAAAATCCCGTG
AACCATCGAGTCTTTGAACGCAAGTTGCGCCCGAAGCCATTCGGTCGAGGGCACTTCT
GCCTGGGTGTCACCCAACGTCGTCCCCAAACCCGTTCCT-CTCTCCAACG----
GGGGGGAT-----CGGGAGGGACGGAAGCTGGTCTCCCGGGGCCATCCCCC------------------
--CCCGC-------------

Banisteriopsis_vernoniifolia
TGTTGGGATGGTAGGCTCCGTGCTTTTTGCATCGAACTGCTAGACCGCAATCCTTTTC
AAGYCTCRAGCATTGCTTTTATGCTCGGGTTATCGAC-------
CGTGTTCCTGTATTTGATACCCATGTCGGAGGAACTAGGCTTCTG-TTCTCCCC----
AGTCCACTCGATGCCTCTTACGGGCATCGAGCGGAGCACGTAGTT-
CGATCTGTCGAGCCTAGCACGCCTK---TTCGGYTGCAACGGTCGTYGGATCAAAGC-
TTGTGTTCTCGGATGCAGAATATTCGATGGGTCCAAGGATTGAATCCTTG--
CATTGGCCCTGTGTGGGAAATCGTCCCTCGATGAAAACGACGTCCGCCGTGCGTCGCG
ATCCGTCCGGTGTCCTCTCGGAC?GAGGACGCTTCGCGCGTGCGTCGGCGTC-
CTCGAAGGAATGCTACCTGGTTGCTCCTGCC------CATATGCTTGTCT-------------------
CCTGCCCAGCAGAACGACCCGCGAACCG-
TCGTCGAAGCGCGCCGAGGAGCGAGCCGGGCCGATAGTCCCGCCCGCTCCACGACGC
AGCCCGCCGCGTGGCCCC-
GGCCGCCCGCGGGAACAACACCCACCCCCGGCGCAGAACGCGCCAAGGAAAAC-
AAACTAAGGAGGAATCTTCCC--GTGCTCCCGAACGCGGCGAGCGCGGGG-
AAGTTCCGCCCAAGGTCG-
AACAAAAACGACTCTCGGCAACGGATATCCCGGCTCTCGCATCGATGAAAAACGTAG
CAAAATGCGATACTTGGTGTGAATTGGAAAATCCCGTGAACCCTCGAGTCTTTGAACG
CAAGTTGCGCCCCAAGCCTTTCCGTCGAAGGCACGTTTGCCTGGGGGGCACACAACGT
TCTCCCCAAACCCGACCCC-CTCTCCCACG----GGGGGA--

ATTTAAATTATGTGTCTGATTCACTAATACCTTACCCCATCCATCTAGAAAAATTGGTT
CAAATCCTTCGTTACTGGATGCAAGATCCCTCTTCTTTGCATTTATTACGACTCTTTCTT
CACGAGTATTGGAATTGGAATAGTTTTATTATTTCAACGAAATCCTTTTCCATTTTTTT
TAATTTAAAAAGGAATCTAAGATTATTGTTGTTCCTATATAATTCTCATGTATATGAAT
ACGAATCCATCTTCTTTTTTCTCCGTAACCAATCCTTTTATTTCCAATCAACATTTTTGG
GGGTGCTTCTTGAACGAATATATTTCTATAGAAAAATACAAGATTTTGTAGAACTTTT
TACTAATG------
ATTTTAAGGCTAACCTACGGTTGTTCAAGGATCCTTTCATGCATTATGTTAGATATCAA
GGAAAAGGCATTCTGGCTTCAAAAAGTACACTTCTTCTGATGAAAAAATGGAAATATT
TCTTTGTCAATTTATGTCAATTTCATTTTTATGTGTGGGTTCATCCAGAACCGATCTAT
ATGAAGTCATTATCCAACCATTCTCTTGACTTTTTGGGTTATCTTTCAAATATACGAAA
AAATCTTTCAGTGGTACGGAGTCAAATGCTAGAAAATTCATTTCTAATAGATAATATT
ATGAAGAAACTCGATTCAAGAGTTCCAATTATTCCTTTGATTGGATCATTGTCAAAAA
CAAAATTTTGTAACGCAGTAGGGCATCCCATTAGTAAACCGAGCTGGGCGGATTCACC
CGATTCTGACATTATCGACCAATTTTTGCGTATATCCAGAAATCTTTCTCATTACTATA

GCGGATCCTCAAAAAAAAAGAATTTGTTTCGAGTAAAATATATACTTCGACTTTCTTG
TGTTAAAACTTTGGCTCGTAAACACAAAAGTACCGTCCGGGCTTTTTTGAAAAAACTA
GGTTCAGAATTATTGGAAGAATTTTTTACGGAGGAAAAATCAATTCTTGATTTGGTCT
TCCCCACGGCTTATTCTATTTCGCGAAGGTTATACAGAGGGCGGGTTTGGGATTTGGA
TATTATTTGGATCAATGATCTAGCCAATCATGAA---
GATTAACTGCATTTTATATGTTTCGGGTTTATTTACTTACTTTTGAAGGACAATTAAAC
CTTTATTTTCAAAATTACAGTGGCAAAAAAAACAGTGCATTCTATTCAATATCTCTAT
GGGGTAAAGAAGGATCAAAAATACTAAAAAAAAAAAATTCCTTTATTA--------------
TCTTTATTAACAATCACTAATAATGAAAAGACTTCTTTTTTTTTGAACAAAATATACAA
AATTGATGGTAATGTAAGAAATATGACGCGTCCCTCTATTACTATTAATCATAATCAT
TTTTTCACGAAAAATATTTTTCCCTATCCCCCTGAAGCGGAAAATACTATGTTATTTCC
TATGCTTGTATTGGTACTATTTACTTTGTTTATTGGAGCTATAGGAATTCCTTTCAATC
AAGAGGGAACTAATTTGGATATATTATCCAAACTGTTAACTCCGTCTTTAAATCTTTTG
CATCAAAATGAAAATAATTCTTTTGATTGGTATGAATTTATAACAAATGCTATTTTTTC
AGTCATTATAACTTTTTTTGGAATATTTATAGCGTCTTCCTTCTATAAACCTCTTTATTC
ATCTCTACAAAATTTGAACTTACTTAACTCATTTGCTA------AAAAA---------
AGGGGTCGTTGGGATAAAATAATAGGTGCGATATATGATTGGTCCTATAATCGTGGTT
ATATCGATGTTTTTTATGCAATATT---

Bronwenia_cinerascens ---

-????????????TGTCTGACCCACTAATACCTTACCCCATCCATCTAGAAAAATTAGTTCAA
ATCCTTCGTTACTGGATGCAAGATCCCTCTTCTTTGCATTTATTACGACTCTTTCTTCAC
GAGTATTGGAATTGGAATAGTTTTATTATTTCAACGAAATCCTTTTCCATTTTTTTTAA
TTTAAAAAGGAATCTAAGATTATTGTTGTTCCTATATAATTCTCATGTATATGAATACG
AATCCATCTTCTTTTTTCTCCGTAACCAATCCTTTTATTTCCGATCAACATTTTCGGGGG
TGCTTCTTGAACGAATATATTTCTATGGAAAAATACAAGATTTTGTAGAACTTTTTACT
AATG------
ATTTTAAGGCTAACCTACGGTTGTTCAAGGATCCTTTCATGCATTATGTTAGATATCAA
GGAAAAGGCATTCTGGCTTCAAAAAGTACACTTCTTCTGATGAAAAAATGGAAATATT
TTTTTGTCAATTTATGTCAATTTCATTTTTATGTGTGGGTTCATCCAGAACCGATCTAT
ATGAAGTCATTATCCAACCATTCTCTTGACTTTTTGGGTTATCTTTCAAGTATACGAAA
AAATCTTTCAGTGGTACGGAGTCAAATGCTAGAAAATTCATTTCTAATAGATAATATT
AGGAAGAAACTCGATTCAAGAGTTCCAATTATTCCTTTGATTGGATCATTGTCAAAAA
CAAAATTTTGTAACGCAGTAGGGCATCCCATTAGTAAACCGAGCTGGGCGGATTCACC

CGATTCTGATATTATCGACCAATTTTTGCGTATATCCAGAAATCTTTCTCATTACTATA
GTGGATCCTCAAAAAAAAAGAGTTTGTATCGAGTAAAATATATACTTCGACTTTCTTG
TGTTAAAACTTTGGCTCGTAAACACAAAAGTACCGTCCGGGCTTTTTTGAAAAAACTA
GGTTCAGAATTATTGGAAGAATTTTTTACGGAGGAAAAATCGATTCTTGATTTGGTCT
TCCCCACGGCTTATTCTATTTCGCGAAGGTTATACAGAGGGCGGGTTTGGTATTTGGA
TATTATTTGTATCAATGATCTAGCCAATCATGAATAAGATTAACTGCATTTTATATGTT
TCGGGTTTATTTACTTACTTTTGAAGGACATTTAAATCTTTATTTTCAAAATTACAGTG
GCAAAAAAAAACAGTTCATTCTATTCAATATCTCTATGGGGTAAAGAAGGATCCAAAA
TACTAAAAAAAAAAAATTCCTTTATTA---------------
CCTTTATTAACAATCACTAATAATGAAAAGACTTCTTTTTTTTTGAACAAAATATATCA
AATTGACGGTAATGTAAGAAATAGGACGCGTCCCTCTATTACTAT------
TAATCATTTTTTCACGAAAAATATTTTTCCCTATCCCCCTGAAGCGGATAATACTATGT
TATTTCCTATACTTGTGTTGGTACTATTTACTTTGTTTATTGGAGCTATAGGAGTTCCTT
TCAATCAAGAGGGAACTAATTTGGATATATTATCCAAACTGTTAACTCCGTCTTTAAA
TCTTTTGCATCAAAATGAAAATAATTCTTTTGATTGGTATGAATTTATAACAAATGCTA
TTTTTTCAGTCATTATAACTTTTTTTGGAATATTTATAGCGTCTTCCTTCTATAAACCTC
TTTATTCATCGCTACAAAATTTGAACTTACTTAACTCATTTACTA------
AAAAAGGTCCTAAGAGGGGTCGTTGGGACAAAATAATAGGTGCGATATATGATTGGT
CCTATAATCGTGGTTATATCGATGTTTTTATGCAATATTGGCTTCAAGGTTTCT?TTCA
TGAAAAACAAGGTACGGATGATATGCGATTGTCTGGCTCCTCCAGTCAAAGTAATCCA
AGACGAGAGATTGCCTCAGCCGTTAAGTCTTTGTGGGTCTACATTAAGATCTCCTCAT
GGCTGCCATGCTCAGTATATGACAAATATGGGGACAATTGCATCTCTTGTGATGTCTG
TGACTATCAATGAAGATGATGATACGATGGATGGTGATCAGCAGCAGATGACAAGAA
AATTGTGGGGCTTAGTTGTTTGTCATCATACTAGTCCTCAATTTGTTCCGTTTCCTTTG
AGGTATGCTTGTGAATTTTTGATTCAAGTGTTTGGTGTTCAGATCAACAAGGAGGTAG
AGTTAGCTGCTCAGATGAGGGAAAAACATATTTTACAAATTCAGACTATGCTATGTGA
CATGCTCCTTAGAGATGCTCCCGTTGCAATCATCACACAATCTCCAAATGTGATGGAT
CTTGTTAAGTGTGATGGAGCTGCCCTATACTTCAAGAACAAAACTTGGTTGCTGGGAG
TAACCCCTACAGAGGAACAAATCAGAGACATAGCTGAATGGCTGCTCGAATATCATA
GTGGAAACACGGGTTTAAGTACTGACAGCCTTATGGAAGCTGGATATCCTGGTGCTTC
AGCTCTTGGTGATGCAGTTTGTGGGATGGCTGCTGTTAGCATTACTTCGAGAGATTTTC
TTTTTTGGTTTAGATCTCACACTGCTAAAGAGATCAAGTGGGGCGGTGCAAAACATGA
TCCTGATGACAAAGATGACTTAAGAAAGATGCATCCGAGGTCATCCTTCAAGGCCTTT
CTAGAGGTGGTAAAAATAAATTGACTTATTATACTCCTGACTATGAAACCAAAGATAC
TGATATCTTGGCAGCATTCCGAGTAACTCCTCAACCTGGAGTTCCGCCTGAGGAAGCA
GGCGCTGCGGTAGCTGCTGAATCTTCTACTGGTACATGGACAACTGTGTGGACCGATG
GACTTACCAGTCTTGATCGTTATAAAGGGCGATGCTACCACATCGAGCCCGTTGCTGG
AGAAGAAAATCAATATATTGCTTATGTAGCTTACCCCTTAGACCTTTTTGAAGAAGGT
TCTGTTACTAACATGTTTACTTCCATTGTGGGTAATGTATTTGGGTTCAAAGCCCTACG
CGCTCTCCGTCTGGAGGATTTGCGAATCCCTCCTGCTTATTCGAAAACTTTCCAAGGCC
CGCCTCATGGTATCCAAGTTGAGAGAGATAAATTGAACAAGTATGGTCGCCCCCTATT
GGGCTGTACTATTAAACCTAAATTGGGGTTATCTGCTAAGAATTACGGTAGAGCTGTT
TATGAATGTCTACGCGGTGGACTTGATTTTACCAAAGACGACGAGAACGTGAACTCCC
AACCATTTATGCGTTGGAGAGACCGTTTCTTATTTTGTGCCGAAGCACTTTATAAAGC
ACAGGCGGAAACAGGGGAAATCAAAGGGCATTATTTGAATGCTACTGCAGGTACATG
CGAAGAAATGATCAAAAGGGCTGTATGTGCCAGAGAATTGGGAGTTCCTATCGTAAT
GCATGACTACTTAACAGGGGGATTCACCGCAAATACTAGCTTGGCTCATTATTGCCGA
GATAATGGTTTACTTCTGCACATCCATCGCGCAATGCATGCAGTTATTGATAGACAGA
AGAATCATGGTATGCACTTTCGTGTACTAGCTAAAGCGTTACGCCTGTCTGGTGGAGA
TCATATTCACGCGGGTACTGTAGTAGGGAAACTTGAGGGGGAAAGAGAGATCACTTT
AGGCTTTGTTGATTTACTGCGTGATGATTTTGTTGAAAAAGATAGAAGCCGCGGGATT
TATTTCACTCAAGACTGGGTCTCCCTACCAGGTGTTATACCCGTGGCTTCGGGAGGTA
TTCACGTTTGGCATATGCCTGCTCTGACCGAGATCTTTGGAGATGATTCCGTACTACA
ATTCGGTGGAGGAACTTTGGGGCACCCTTGGGGAAATGCACCAGGTGCCGTAGCTAA
TCGAGTAGCTCTAGAAGCATGTGTACAAGCTCGTAATGAGGGACGTGATCTTGCTCGT
GAGGGTAATGAAATTATCCGTGAGGCTAGCAAATGGAGTCCTGAACTAGCTGCTGCTT
GTGAAGTGTGGAAAGAGATTAA-
Bronwenia_ferruginea --

ATTTAAATTATGTGTCTGATCCACTAATACCTTACCCCATCCATCTAGAAAAATTGGTT
CAAATCCTTCGTTACTGGATGCAAGATCCCTCTTCTTTGCATTTATTACGACTCTTTCTT
CACGAGTATTGGAATTGGAATAGTTTTATTATTTCAACGAAATCCTTTTCCATTTTTTT
TAATTTAAAAAGGAATCTAAGATTATTGTTGTTCCTATATAATTCTCATGTATATGAAT
ACGAATCCATCTTCTTTTTTCTCCGTAACCAATCCTTTTCTTTCCGATCAACATTTTCGG
GGGTGCTTCTTGAACGAATATATTTCTATGGAAAAATACAAGATTTTGTAGAACTTTT
TACTAATG------
ATTTTAAGGCTAACCTACGGTTGTTCAAGGATCCTTTCATGCATTATGTTAGATATCAA
GGAAAAGGCATTCTGGCTTCAAAAAGTACACTTCTTCTGATGAAAAAATGGAAATATT
TTTTTGTCAATTTATGTCAATTTCATTTTTATGTGTGGGTTCATCCAGAACCTATCTATA
TGAAGTCATTATCCAACCATTCTCTTGACTTTTTGGGTTATCTTTCAAGTATACGAAAA
AATCTTTCAGTGGTACGGAGTCAAATGCTAGAAAATTCATTTCTAATAGATAATATTA
GGAAGAAACTCGATTCAAGAGTTCCAATTATTCCTTTGATTGGATCATTGTCAAAAAC
AAAATTTTGTAACGCAGTAGGGCATCCCATTAGTAAACCGAGCTGGGCGGATTCACCC
GATTCTGATATTATCGACCAATTTTTGCGTATATCCAGAAATCTTTCTCATTACTATAG
TGGATCCTCAAAAAAAAAGAGTTTGTATCGAGTAAAATATATACTTCGACTTTCTTGT
GTTAAAACTTTGGCTCGTAAACACAAAAGTACCGTCCGGGCTTTTTTGAAAAAACTAG
GTTCAGAATTATTGGAAGAATTTTTTACGGAGGAAAAATTGATTCTTGATTTGGTCTTC
CCCACGGCTTATTCTATTTCGCGAAGGTTATACAGAGGGCGGGTTTGGTATTTGGATA
TTATTTGTATCAATGATCTAGCCAATCATGAATAAGATTAACTGCATTTTATATGTTTC
GGGTTTATTTACTTACTTTTGAAGGACATTTAAATCTTTATTTTCAAAATTACAGTGGC
AAAAAAAACAGTTCATTCTATTCAATATCTCTATGGGGTAAAGAAGGATCCAAAATA
CTAAAAAAAAAAATTCCTTTATTA---------------
CCTTTATTAACAATCACTAATAATGAAAAGACTTCTTTTTTTTTGAACAAAATATATCA
AATTGACGGTAATGTAAGAAATATGACGCGTCCCTCTATTACTAT------
TAATCATTTTTTCACGAAAAATCTTTTTCCCTATCCCCCTGAAGCGGATAATACTATGT
TATTTCCTATACTTGTGTTGGTACTATTTACTTTGTTTATTGGAGCTATAGGAGTTCCTT
TCAATCAAGAGGGAACTAATTTGGATATATTATCCAAACTGTTAACTCCGTCTTTAAA
TCTTTTGCATCAAAATGAAAATAATTCTTTTGATTGGTATGAATTTATAACAAATGCTA
TTTTTTCAGTCATTATAACTTTTTTTGGAATATTTATAGCGTCTTCCTTCTATAAACCTC
TTTATTCATCGCTACAAAATTTGAACTTACTTAACTCATTTACTA------
AAAAAGGTCCTAAGAGGGGTCGTTGGGACAAAATAATAGGTGCGATATATGATTGGT
CCTATAATCGTGGTTATATCGATGTTTTTTATGCAATATT?GCTTCAAGGTTTCTCTTCA
TGAAAAACAAGGTACGGATGATATGCGATTGTCTGGCTCCTCCGGTCAAAGTAATCCA
AGATGAGAGATTGCCTCAGCCGTTAAGTCTTTGTGGGTCTACATTAAGATCTCCTCAT
GGCTGCCATGCTCAGTATATGACAAATATGGGGACAATTGCATCTCTTGTGATGTCTG
TGACTATCAATGAAGATGATGATACGATGGATGGTGATCAGCAGCAGATGACAAGAA
AATTGTGGGGCCTAGTTGTTTGTCATCATACTAGTCCTCGATTTGTTCCGTTTCCTTTG
AGGTATGCTTGTGAATTTTTGATTCAAGTGTTTGGTGTTCAGATCAACAAGGAGGTAG
AGTTAGCTGCTCAGATGAGGGAAAAACATATTTTACAAATTCAGACTATGCTATGTGA
CATGCTCCTTAGAGATGCTCCCGTTGCAATCATCACACAATCTCCAAATGTGATGGAT
CTTGTTAAGTGTGAGGGAGCTGCCCTATACTTCAAGAACAAAACTTGGTTGCTGGGAG
TAACCCCTACAGAGGAACAAATCAGAGACATAGCTGAATGGCTGCTCGAATATCATA
CTGGAAACACGGGTTTAAGTACTGACAGCCTCATGGAAGCTGGATATCCTGGTGCTTC
AGCTCTTGGTGATGCAGTTTGTGGGATGGCTGCTGTTAGCATTACTTCGAGAGATTTTC
TTTTTTGGTTTAGATCTCACACTGCTAAAGAGATCAAGTGGGGCGGTGCAAAACATGA
TCCTGATGACAAAGATGACTTAAGAAAGATGCATCCGAGGTCATCCTTCAAGGCCTTT
CTAGAGGTGGTAAAAATAAATTGACTTATTATACTCCTGACTATGAAACCAAAGATAC
TGATATCTTGGCAGCATTCCGAGTAACTCCTCAACCTGGAGTTCCGCCTGAGGAAGCA
GGCGCTGCGGTAGCTGCTGAATCTTCTACTGGTACATGGACAACTGTGTGGACCGATG
GACTTACCAGTCTTGATCGTTATAAAGGGCGATGCTACCACATCGAGCCCGTTGCTGG
AGAAGAAAATCAATATATTGCTTATGTAGCTTACCCCTTAGACCTTTTTGAAGAAGGT
TCTGTTACTAACATGTTTACTTCCATTGTGGGTAATGTATTTGGGTTCAAAGCCCTACG

CGCTCTCCGTCTGGAGGATTTGCGAATCCCTCCTGCTTATTCGAAAACTTTCCAAGGCC
CGCCTCATGGTATCCAAGTTGAGAGAGATAAATTGAACAAGTATGGTCGCCCCCTATT
GGGCTGTACTATTAAACCTAAATTGGGGTTATCTGCTAAGAATTACGGTAGAGCTGTT
TATGAATGTCTACGCGGTGGACTTGATTTTACCAAAGACGACGAGAACGTGAACTCCC
AACCATTTATGCGTTGGAGAGACCGTTTCTTATTTTGTGCCGAAGCACTTTATAAAGC
ACAGGCGGAAACAGGTGAAATCAAAGGGCATTATTTGAATGCTACTGCAGGTACATG
CGAAGAAATGATCAAAAGGGCTGTATGTGCCAGAGAATTGGGAGTTCCTATCGTAAT
GCATGACTACTTAACAGGGGGATTCACCGCAAATACTAGCTTGGCTCATTATTGCCGA
GATAATGGTTTACTTCTGCACATCCATCGCGCAATGCATGCAGTTATTGATAGACAGA
AGAATCATGGTATGCACTTTCGTGTACTAGCTAAAGCGTTACGCCTGTCTGGTGGAGA
TCATATTCACGCGGGTACTGTAGTAGGGAAACTTGAGGGGGAAAGAGAGATCACTTT
AGGCTTTGTTGATTTACTGCGTGATGATTTTGTTGAAAAAGATAGAAGCCGCGGGATT
TATTTCACTCAAGACTGGGTCTCCCTACCAGGTGTTATACCCGTGGCTTCGGGAGGTA
TTCACGTTTGGCATATGCCTGCTCTGACCGAGATCTTTGGAGATGATTCCGTACTACA
ATTCGGTGGAGGAACTTTGGGGCACCCTTGGGGAAATGCACCAGGTGCCGTAGCTAA
TCGAGTAGCTCTAGAAGCATGTGTACAAGCTCGTAATGAGGGACGTGATCTTGCTCGT
GAGGGTAATGAAATTATCCGTGAGGCTAGCAAATGGAGTCCTGAACTAGCTGCTGCTT
GTGAAGTGTGGAAAGAGATTAA-
Diplopterys_cabrerana --

ATTTAAATTATGTGTCTGATCCACTAATACCTTACCCCATCCATCTAGAAAAATTGGTT
CAAATCCTTCGTTACTGGATGCAAGATCCCTCTTCTTTGCATTTATTACGACTCTTTCTT
CACGAGTATTGGAATTGGAATAGTTTTATTATTTCAACGAAATCCTTTTCCACTATTTT
TAATTTAAAAAGGAATCTAAGATTATTGTTGTTCCTATATAATTCTCATGTATATGAAT
ACGAATCCATCTTCTTTTTTCTCCGTAACCAATCCTTTTATTTCCGATCAACATTTTCGG
GGGTGCTTCTTGAACGTATATATTTCTATGGAAAAATACAAGATTTTGTAGAACTTTTT
ACTAATG------
ATTTTAAGGCTAACCTACGGTTGTTCAAGGATCCTTTCATGCATTATGTTAGATATCAA
GGAAAAGGTATTCTGGCTTCAAAAAGTACACTTCTTCTGATGAAAAAATGGAAATATT
TCTTTGTCAATTTATATCAATTTCATTTTTATGTGTGGGTTCATTCAGAACCGATCTAT
ATGAAGTCATTATCCAACCATTCTCTTGACTTTTTGGGTTATCTTTCAAGTATACGAAA
AAATCTTTCAGTGGTACGGAGTCAAATGCTAGAAAATTCATTTCTAATAGATAATATT
ATGAAGAAACTCGATTCAAGAGTTCCAATTATTCCTTTGATTGGATCATTGTCAAAAA
CAAAATTTTGTAACACAGTAGGGCATCCCATTAGTAAACCGAGCTGGGCGGATTCACC
CGATTCTGATATTATCGACCAATTTTTGCGTATATCCAGAAATCTTTCTCATTACTATA
GCGGATCCTCAAAAAAAAAGAGTTTGTATCGAGTAAAATACATACTTCGACTTTCTTG
TGTTAAAACTTTGGCTCGTAAACACAAAAGTACCGTCCGGGCTTTTTTGAAAAAACTA
GGTTCAGAATTATTGGAAGAATTTTTTATGGAGGAAAAATCGATTCTTGATTTGGTCT
TCCCCACGGCTTATTCTATTTCGCGAAGGTTATACAGAGGGCGGGTTTGGTATTTGGA
TATTATTTGTATCAATGATCTAGCCAATCATGAATAA??????????????????????CGGGTT
TATTTACTTACTTTTGAAGGACATTTAAATCTTTATTTTCAAAATTACAGTGGCAAAAA
AAACAATTCATTCTATTCAATATCTCTATGGGGTAAAGAAGGATCAAAAATACTAAAA
AAAAAAATTCCTTTATTA---------------
CCTTTATTAACAATCACTAATAATGAAAAGACTTCTTTTTTTTTGAACAAAACATATCA
AATTGACGGTAATGTAAGAAATATCACGCGTCCCTTTATTACTAT------
TAATCATTTTTTCACGAAAAATCTTTTTCCCTATCCCCCTGAAGCGGATAATACTATGT
TATTTCCTATGCTTGTGTTGGTACTATTTACTTTGTTTATTGGAGCTATAGGAGTTCCTT
TCAATCAAGAGGGAACTAATTTGGATATATTATCCAAACTTTTCACTCCGTCTTTAAAT
CTTTTGCATCAAAATGAAAATAATTCTTTTGATTGGTATGAATTTATAACAAATGCTAT
TTTTTCAGTCATTATAACTTTTTTTGGAATATTTATAGCGTCTTCCTTCTACAAACCTAT
TTATTCATCGTTACAAAATTTGAACTTACTTAACTCATTTGTTA------
AAAAAGGTCCTAAGAGGGGTCGTTGGGACAAAATAATAGGTGCGATATATAATTGGT

CCTATAATCGTGGTTATATCGATGCT??????????????AGCTTCAAGGTTTCTCTTCATGAA
AAACAAGGTACGCATGATATGCGATTGTCTGGCTCCTCCAGTCAAAGTAATCCAAGAT
GACAGACTGCCTCAGCCATTAAGTCTTTGTGGGTCTACGTTAAGATCTCCTCATGGTT
GCCATGCTCAGTACATGACAAATATGGGGACAATTGCATCTCTTGTGATGTCTGTGAC
AATCAACGAAGATGATGATACGATGGATGGTGATCAGCAGCAGATGACAAGAAAATT
GTGGGGCCTAGTAGTTTGTCATCATACTAGTCCTCGATTTGTTCCATTCCCTTTGAGGT
ATGCTTGTGAATTTTTGATTCAAGTGTTTGGTGTTCAGATCAACAAGGAGGTAGACTT
AGCGGTTCAGATGAGGGAAAAACATATTTTACAAATTCAGACTATGTTATGTGACATG
CTCCTTAGAGATGCTCCTGTTGCAATTATCACACAATCTCCGAATGTGATGGATCTTGT
TAAGTGTGATGGAGCTGCCCTATACTTTAGGAACAAAACTTGGTTGCTGGGAGTAACC
CCTACAGAGGAACAAATCAGAGACATAGCTGAATGGCTGCTCAAGTATCATAGTGGA
AACACGGGTTTAAGTACTGACAGCCTTATGGAAGCTGGATATCCAGGTGCTTCAGCTC
TTGGTGATGCAGTTTGTGGGATGGCTGCTGTTAGCATTACTTCAGGCGATTTTCTTTTT
TGGTTTAGATCTCACACTGCTAAAGAGATCAAGTGGGGCGGTGCAAAGCATGACCCT
GATGACAAAGATGACTTAAGAAAGATGCATCCGAGGTCACCATTCAAGGCCTTTCTA
GAGGTGGTAAAAATAAATTGACTTATTATACTCCTGACTATGAAACCAAAGATACTGA
TATCTTGGCAGCATTCCGAGTAAGTCCTCAACCTGGAGTTCCGCCTGAGGAAGCAGGT
GCTGCGGTAGCTGCTGAATCTTCTACTGGTACATGGACAACTGTGTGGACCGATGGAC
TTACCAGTCTTGATCGTTATAAAGGGCGATGCTACCACATCGAGCCCGTTGCTGGAGA
AGAAAATCAATATATTGCTTATGTAGCTTACCCCTTAGACCTTTTTGAAGAAGGTTCT
GTTACTAACATGTTTACTTCCATTGTGGGTAATGTATTTGGGTTCAAAGCCCTACGCGC
TCTCCGTCTGGAGGATTTGCGAATCCCTCCTGCTTATTCGAAAACTTTCCAAGGCCCGC
CTCATGGTATCCAAGTTGAGAGAGATAAATTGAACAAGTATGGTCGCCCCCTATTGGG
CTGTACTATTAAACCTAAATTGGGATTATCTGCTAAGAATTACGGTAGAGCTGTTTAT
GAATGTCTACGCGGTGGACTTGATTTTACCAAAGACGACGAGAACGTGAACTCCCAA
CCATTTATGCGTTGGAGAGACCGTTTCTTATTTTGTGCCGAAGCACTTTATAAAGCAC
AGGCGGAAACAGGTGAAATCAAAGGGCATTATTTGAATGCTACTGCAGGTACATGTG
AAGAAATGATCAAAAGGGCTGTATTTGCCAGAGAATTGGGAGTTCCTATCGTAATGC
ATGACTACTTAACAGGGGGATTCACCGCAAATACTAGCTTGGCTCATTATTGCCGAGA
TAATGGTTTACTTCTGCACATCCATCGCGCAATGCATGCAGTTATTGATAGACAGAAG
AATCATGGTATGCACTTTCGTGTACTAGCTAAAGCGTTACGCCTGTCTGGTGGAGATC
ATATTCACGCGGGTACTGTAGTAGGGAAACTTGAGGGGGAAAGAGAGATCACTTTAG
GCTTTGTTGATTTACTGCGTGATGATTTTGTTGAAAAAGATAGAAGCCGCGGGATTTA
TTTCACTCAAGACTGGGTCTCCCTACCAGGTGTTATACCTGTGGCTTCGGGAGGTATTC
ACGTTTGGCATATGCCTGCTCTGACCGAGATCTTTGGAGATGATTCCGTACTACAATT
CGGTGGAGGAACTTTGGGGCACCCTTGGGGAAATGCACCAGGTGCCGTAGCTAATCG
AGTAGCTCTAGAAGCATGTGTACAAGCTCGTAATGAGGGACGTGATCTTGCTCGTGAG
GGTAATGAAATTATCCGTGAGGCTAGCAAATGGAGTCCTGAACTAGCTGCTGCTTGTG
AAGTGTGGAAAGAGATTAA-
Diplopterys_hypericifolia --

ATTTAAATTATGTGTCTGATCCACTAATACCTTACCCCATCCATCTAGAAAAATTGGTT
CAAATCCTTCGTTACTGGATGCAAGATCCCTCTTCTTTGCATTTATTACGACTCTTTCTT
CACGAGTATTGGAATTGGAATAGTTTTATTATTTCAACGAAATCCTTTTCCACTATTTT
TAATTTAAAAAGGAATCTAAGATTATTGTTGTTCCTATATAATTCTCATGTATATGAAT
ACGAATCCATCTTCTTTTTTCTCCGTAACCAATCCTTTTATTTCCGATCAACATTTTCGG
GGGTGCTTCTTGTACGAATATATTTCTATGGAAAAATACAAGATTTTGTAGAACTTTTT
ACTAATG------
ATTTTAAGGCTAACCTACGGTTGTTCAAGGATCCTTTCATGCATTATGTTAGATATCAA
GGAAAAGGCATTCTGGCTTCAAAAAGTACACTTCTTCTGATGAAAAAATGGAAATATT
TCTTTGTCAATTTATATCAATTTCATTTTTATGTGTGGTTTCATTCAGAACCGATCTATA
TGAAGTCATTATCCAACCATTCTCTTGACTTTTTGGGTTATCTTTCAAGTATACGAAAA

AATCTTTCAGTGGTACGGAGTCAAATGCTAGAAAATTCATTTCTAATAGATAATATTA
TGAAGAAACTCGATTCAAGAGTTCCAATTATTCCTTTGATTGGATCATTGTCAAAAAC
AAAATTTTGTAACACAGTAGGGCATCCCATTAGTAAACCGAGCTGGGCGGATTCACCC
GATTCTGATATTATCGACCAATTTTTGTGTATATCCAGAAATCTTTCTCATTACTATAG
CGGATCCTCAAAAAAAAAGAGTTTGTATCGAGTAAAATACATACTTCGACTTTCTTGT
GTTAAAACTTTGGCTCGTAAACACAAAAGTACCGTCCGGGCTTTTTTGAAAAAACTAG
GTTCAGAATTATTGGAAGAATTTTTTATGGAGGAAAAATCGATTCTTGATTTGGTCTTC
CCCACGGCTTATTCTATTTCGCGAAGGTTATACAGAGGGCGGGTTTGGTATTTGGATA
TTATTTGTATCAATGATCTAGCCAATCATGAATAAGATTAACTGCATTTTATATGTTTC
GGGTTTATTTACTTACTTTTGAAGGACATTTAAATCTTTATTTTCAAAATTACAGTGGC
AAAAAAAACAATTCATTCTATTCAATATCTCTATGGGGTAAAGAAGGATCAAAAATA
CTAAAAAAAAAAAATTCCTTTATTA---------------
CCTTTATTAACAATCACTAATAATGAAAAGACTTCTTTTTTTTTGAACAAAATATATCA
AATTGACGGTAATGTAAGAAATATCACGCGTCCCTTTATTACTAT------
TAATCATTTTTTCACGAAAAATCTTTTTCCCTATCCCCCTGAAGCGGATAATACTATGT
TATTTCCTATGCTTGTGTTGGTACTATTTACTTTGTTTATTGGAGCTATAGGAGTTCCTT
TCAATCAAGAGGGAACTAATTTGGATATATTATCCAAACTTTTCACTCCGTCTTTAAAT
CTTTTGCATCAAAATGAAAATAATTCTTTTGATTGGTATGAATTTATAACAAATGCTAT
TTTTTCAGTCATTATAACTGTTTTTGGAATATTTATAGCGTCTTCCTTCTACAAACCTAT
TTATTCATCGTTACAAAATTTGAACTTACTTAACTCATTTGTTA------
AAAAAGGTCCTAAGAGGGGTCGTTGGGACAAAATAATAGGTGCGATATATGATTGGT
CCTATAATCGTGGTTATATCGATGTTTTTTATGCAATATTAGCTTCAAGGTTTCTCTTC
ATGAAAAACAAGGTACGCATGATATGCGATTGTCTGGCTCCTCCAGTCAAAGTAATCC
AAGACGACAGACTGCCTCAGCCATTAAGTCTTTGTGGGTCTACGTTAAGATCTCCTCA
TGGTTGCCACGCTCAGTACATGACAAATATGGGGACAATTGCATCTCTTGTGATGTCT
GTGACAATCAACGAAGATGATGATACGATGGATGGTGATCAGCAGCAGATGACAAGA
AAATTGTGGGGCCTAGTTGTTTGTCATCATACTAGTCCTCGATTTGTTCCATTCCCTTT
GAGGTATGCTTGTGAATTTTTGATTCAAGTGTTTGGTGTTCAGATCAACAAGGAGGTA
GAGTTAGCGGCTCAGATGAGGGAAAAACATATTTTACAAATTCAGACTATGCTATGTG
ACATGCTCCTTAGAGATGCTCCTGTTGCAATTATCACACAATCTCCGAATGTGATGGA
TCTTGTTAAGTGTGATGGAGCTGCCCTATACTTCAAGAACAAAACTTGGTTGCTGGGA
GTAACCCCTACAGAGGAACAAATCAGAGACATAGCTGAATGGCTGCTCAAATATCAT
AGTGGAAACACGGGTTTAAGTACTGACAGCCTTATGGAAGCTGGATATCCAGGTGCTT
CAGCTCTTGGTGATGCAGTTTGTGGGATGGCTGCTGTTAGCATTACTTCAAGAGATTTT
CTTTTTTGGTTTAGATCTCACACTGCTAAAGAGATCAAGTGGGGCGGTGCAAAGCATG
ACCCTGATGACAAAGATGACTTAAGAAAGATGCATCCAAGGTCATCATTCAAAGCCT
TTCTAGAGGTGGTAAAAATAAATTGACTTATTATACTCCTGACTATCAAACCAAAGAT
ACTGATATCTTGGCAGCATTCCGAGTAACTCCTCAACCTGGAGTTCCGCCTGAGGAAG
CAGGTGCTGCGGTAGCTGCTGAATCTTCTACTGGTACATGGACAACTGTGTGGACCGA
TGGACTTACCAGTCTTGATCGTTATAAAGGGCGATGCTACCACATCGAGCGCGTTGCT
GGAGAAGAAAGTCAATATATTGCTTATGTAGCTTACCCCTTAGACCTTTTTGAAGAAG
GTTCTGTTACTAACATGTTTACTTCCATTGTGGGTAATGTATTTGGGTTCAAAGCCCTA
CGCGCTCTCCGTCTGGAGGATTTGCGAATCCCTCCTGCTTATACGAAAACTTTCCAAG
GCCCGCCTCATGGTATCCAAGTTGAGAGAGATAAATTGAACAAGTATGGTCGCCCCCT
ATTGGGCTGTACTATTAAACCTAAATTGGGGTTATCCGCTAAGAATTACGGTAGAGCT
GTTTATGAATGTCTACGCGGTGGACTTGATTTTACCAAAGACGACGAGAACGTGAACT
CCCAACCATTTATGCGTTGGAGAGACCGTTTCTTATTTTGTGCCGAAGCACTTTATAAA
GCACAGGCGGAAACAGGTGAAATCAAAGGGCATTATTTGAATGCTACTGCAGGTACA
TGCGAAGAAATGATCAAAAGGGCTGTATTTGCCAGAGAATTGGGAGTTCCTATCGTA
ATGCATGACTACTTAACAGGGGGATTCACCGCAAATACTAGCTTGGCTCATTATTGCC
GAGATAATGGTTTACTTCTGCACATCCATCGCGCAATGCATGCAGTTATTGATAGACA
GAAGAATCATGGTATGCACTTTCGTGTACTAGCTAAAGCGTTACGCCTGTCTGGTGGA
GATCATATTCACGCGGGTACTGTAGTAGGGAAACTTGAGGGGGAAAGAGAGATCACT
TTAGGCCTTGTTGATTTACTGCGTGATGATTTTGTTGAAAAAGATAGAAGCCGCGGGA
TTTATTTCACTCAAGACTGGGTCTCCCTACCAGGTGTTATACCTGTGGCTTCGGGAGGT
ATTCACGTTTGGCATATGCCTGCTCTGACCGAGATCTTTGGAGATGATTCCGTACTAC
AGTTCGGTGGAGGAACTTTGGGGCACCCTTGGGGAAATGCACCAGGTGCCGTAGCTA
ATCGAGTAGCTCTAGAAGCATGTGTACAAGCTCGTAATGAGGGACGTGATCTTGCTCG
TGAGGGTAATGAAATTATCCGTGAGGCTAGCAAATGGAGTCCTGAACTAGCTGCTGCT
TGTGAAGTGTGGAAAGAGATTAA-

Ectopopterys_soejartoi AGTTGGGATGGTAGGCTCCGTGC-
TGTTGCATCGAACTACTAGACCGCA-TCCTCTTCATGGCTCAAGCATTGGTTT---
ACTTTTGCTTGTGACTCGGTGGCGTGTTCCTGTATTTGATACCCATGTCGGTGGAACTT
CGTTTCTG??TTGCCTC----
ATTCCTCTTGGTGCCTCTATGGGGCATCGGGTGGAGCACGTAGCT-
CGATCCGCCAACCCTGGCACGCCGA---TTAGGCTGCGATGGTTGTTGAATCTAGGC--
TGTGCTCTCGGATGCAGAAAATTTAATGGGTCCAAGGATTGAATCCTTGTTTATTTGC
CCTATGCGGGATATCGTCCCTCGGTGCGAACGACATCCTTTGCTCGCCACGATTCATC
CGACGCTCGCTCGGGCTGAGGATGTTTTGTGCGTGCATCGGTGTCTTTTTAAGGAATG
CTACCTGGTTGATCCTGCCAGTAGTCATATGCTTGTCTCAAAGATTAAGCCATGCATG-

ATTTAAATTATGTGTCTGATCCACTAATACCTTACCCTATCCATCTCGAAAAATTGGTT
CAAATCCTTCGTTACTGGATGCAAGATCCCTCTTCTTTGCATTTATTACGACTTTTTCTT
CACGAGTATTGGAATTGGAATAGTTTTATTATTTCAACGAAATCCATTTCCATTTTTTT
GAATTTAAAAAGGAATCTAAGATTATTGTTGTTCCTATATAATTCTCATGTCTATGAAT
ACGAATCCATCTTCTTTTTTCTCCGTAACCAATCCTTTTATTTCCGATCAACATTTTCGC
GGGTGCTTCTTGAACGAATATATTTCTATGGAAAAATACAAGATTTTGTAGAACTTTT
TACTAATG------
ATTTTAAGGCTAACCCACGGTTGTTCAAGGATCCTTTCATGCATTATGTTAGATATCAA
GGAAAAGGCATTCTGGCTTCAAAAAGTACACTTCTTCTGATGAAAAAATGGAAATCTT
TCTTTGTCAATTTATGTCAATCTCATTTTTATGTGTGGTTTCATCCAGAACCGATCTAT
ATGAAGTCATTATCCAACCATTCTCTTGACTTTTTGGGTTATCTTTCAAGTATACGAAA
AAATCTTTCAGTGGTACGGAGTCAAATGCTAGAAAATTCATTTCTAATAGATAATATT
ATGAAGAAACTTGATTCAAGAGTTCCAATTATTCCTTTGATTGGATCATTGTCAAAAA
CAAAATTTTGTAACGCAGCAGGGCATCCCATTAGTAAACCGAGCTGGGCGGATTCAC
CCGATTCTGACATTATCGACCAATTTTTGCGTATATCCAGAGATCTTTCTCATTACTAT
AGCGGATCCTCAAAAAAAAAGGGTTTGTATCGACTAAAATATATACTTCGACTCTCTT
GTGTTAAAACTTTGGCCCGTAAACACAAAAGTACCGTACGGGCTTTTTTGAAAAAACT
AGGTTCAGAATTATTGGAAGAATTTTTTAAGGAGGAAAAATCGATTCTTGATTTGGTC
TTCCCCACAGCTTATTCTATTTCGCGAAGGTTATATAGAGGGCGGGTTTGGTATTTGG
ATATTATTTGTATCAATGATCTAGCCAATCCTGAATAAGATTAACTGCATTTTATATGT
TTCGGGTTTATTTACTCACTTTTGAAGGGCATTTAAATCTTTATTTTCAAAATTACAGT
GGCGAAAAAAACAGCTCATTCTATTCAATATCTCTATGGGGTAAAGAAGGATCAAAA
ATACTAAAAAAAAAAAATTCCTTTATTA---------------
CCTTTATTAACAATGACTAATAATGAAAAGACTTCTTTTTTTTTGAAGAAAATATATCA
AATTGACGGTAATGTAAGAAATATGACGCGTCCCTTTATTACTAT------
TAATCATTTTTTCACGAAAAACATTTTTCCCTATCCCTATGAAGTAGATAATACTATGT
TATTTCCTATGCTTGTGTTGGTACTATTTACTTTGTTTATTGGAGCTATAGGAGTTCCTT
TCAATCAAGAGGGAACTAATTTGGATATATTATCAAAACTGTTAACTCCGTCTTTAAA
TCTTTTGCATCAAAATGAAAATAATTCTTTTGATTGGTATGAATTTATAACAAATGCTC
TTTTTTCAGTCATTATAACTTTTTTTGGAATATTTATAGCATCTTCCTTCTATAAACCTC
TTTATTCATCGCTACAAAATTTGAACTTACTTAACTCATTTGCTA------
AAAAGGGTCCTAAGAGGGGTCGTTGGGACAAAATAATAGGTGCGATATATGATTGGT
CCTATAATCGTGGTTATATCGATGTTTTTTATGCAATATTGGCTTCAAGGTTTCTCTTC
ATGAAAAACAAGGTACGCATGATATGCGATTGTATGGCCCCTCCAGTCGAAGTAATC
CAAGACGAGAGATTGCCTCAGCCATTAAGTCTTTGTGGGTCGACGTTAAGATCTCCTC
ATGGTTGCCATGCGCAGTATATGACAAATATGGGGACAACTGCATCTCTTGTGATGTC
TGTGACTATCAACGAAGATGACCATATGATGGACGGTGATCAGCAGCAGATGACAAA
AAAATTGTGGGGCTTAGTTGTTTGTCACCATACCAATCCTCGATTTGTTCCGTTTCCTT
TGAGGGATGCTTGTGAATTTTTGATTCAAGTGTTTGGTGTTCAGATCAACAAGGAGGT
AGAGTTAGCTGCTCAGGTGAGGGAAAAACATATTTTACAAATTCAGACTATGCTATGC
GACATGCTCCTTAGAGATGCTCCCGTTGCAATTATCACTCAATCTCCGAATGTGATGG
ATCTTGTTAAGTGTGATGGAGCTGCCCTATACTTCAAGAACAAAACTTGGTTGCTGGG
AGTAACCCCTACGGAGGAACAAATCAGAGACATAGCTGAATGGCTTCTCGAATATCA
CAGTGGAAACACGGGTTTAAGTACTGATAGCCTTATGGAAGCTGGATATCCGGGAGC

TTCAGCTCTTGGTGATGCAGTTTGTGGGATGGCTGCTGTTAGCATTACTTCGAGAGATT
TTCTTTTTTGGTTTAGATCTCACACTGCTAAAGAGATCAAGTGGGGCGGTGCAAAACA
TGATCCTGATGACAAAGATGATGGAAGGAAGATGCATCCGAGGTCGTCCTTCAAGGC
CTTTCTAGAGGTTGTAAAAATAAATTGACTTATTATACTCCTGACTATGAAACCAAAG
ATACTGATATCTTGGCAGCATTCCGAGTAACTCCTCAACCTGGAGTTCCGCCTGAGGA
AGCAGGTGCTGCGGTAGCTGCTGAATCTTCTACTGGTACATGGACAACTGTGTGGACC
GATGGGCTTACCAGTCTTGATCGTTATAAAGGGCGATGCTACCACATCGAGCCCGTTG
CTGGAGAAGAAAATCAATATATTGCTTATGTAGCTTACCCCTTAGACCTTTTTGAAGA
AGGTTCTGTTACTAACATGTTTACTTCCATTGTGGGTAATGTATTTGGGTTCAAAGCCC
TACGCGCTCTCCGTCTGGAGGATTTGCGAATCCCTCCTGCTTATTCGAAAACTTTCCAA
GGCCCGCCCCATGGTATCCAAGTTGAGAGAGATAAATTGAACAAGTATGGTCGCCCC
CTATTGGGCTGTACTATTAAACCTAAGTTGGGGTTATCTGCTAAGAATTACGGTAGAG
CTGTTTATGAATGTCTACGCGGTGGACTTGATTTTACCAAAGACGACGAGAACGTGAA
CTCCCAACCATTTATGCGTTGGAGAGACCGTTTCTTATTTTGTGCCGAAGCCCTTTATA
AAGCACAGGCGGAAACAGGTGAAATCAAAGGGCATTATTTGAATGCTACTGCAGGTA
CATGCGAAGAAATGATCAAAAGGGCTGTATTTGCCAGAGAATTGGGAGTTCCTATCG
TAATGCATGACTACTTAACAGGGGGATTCACCGCAAATACTAGCTTGGCTCATTATTG
CCGAGATAATGGTTTACTTCTGCACATCCATCGCGCAATGCATGCAGTTATTGATAGA
CAGAAGAATCATGGTATGCACTTTCGTGTACTAGCTAAAGCGTTACGCCTGTCTGGTG
GAGATCATATTCACGCGGGTACTGTAGTAGGGAAACTTGAGGGGGAAAGAGAGATCA
CTTTAGGCTTTGTTGATTTACTGCGTGATGATTTTGTTGAAAAAGATAGAAGCCGCGG
GATTTATTTCACTCAAGACTGGGTCTCCCTACCAGGTGTTATACCCGTGGCTTCGGGG
GGTATTCACGTTTGGCATATGCCTGCTCTGACCGAGATCTTTGGAGATGATTCCGTACT
ACAATTCGGTGGAGGAACTTTGGGGCACCCTTGGGGAAATGCACCAGGTGCCGTAGC
TAATCGAGTAGCTCTAGAAGCATGTGTACAAGCTCGTAATGAGGGACGTGATCTTGCT
CGTGAGGGTAATGAAATTATCCGTGAGGCTAGCAAATGGAGTCCTGAACTAGCTGCT
GCTTGTGAAGTGTGGAAAGAGATTAAA
Janusia_anisandra ---

-???
??
??
???ACGAAGCTATTTCTAGGGAAAAATAAAAG
ATTTTGTAGCACTTTTTACTAATG------
ATTTTAAGGCTAACCTACGGTTGTTCAACGATCCTTTGATGCATTATGTTAGATATCAA
GGAAAGGGCATTCTGGCTTCAAAAAGCACACTTCTTCTGATGAAAAAATGGAAATAT
TTCTTTGTCAATTTATGTCAATTTCATTTTTATGTGTGGGTTCATCCAAAACCGATCCA
TATGAAGTCATTATCCAACCATTCTCTTGACTTTTTGGGTTATCTTTCAAGTATACGAA
AGAATCTTTCAGTGGTACGGAGTCAAATGCTAGAAAATTCATTTCTAATAGATAAGAC
TATGAAGAAACTTGATTCAAGAATTCCAATTATTCCTTTGATTGGATCATTGTCAAAA
ACCAAATTTTGTAACGCCGTAGGGCATCCCATTAGTAAACCGAGCTGGGCGGATTCGC
CCGATTCTGATATTATCGACCAATTTTTGCGTATATCCAGAAATCTTTCTCATTACTAT
AGCGGATCCTCAAAAAAAAAGAGTTTGTATCGAGTAAAATATATACTTCGACTTTCTT
GTGTTAAAACTTTGGCTCGTAAACACAAAACGACCGTCCGGTCTTTTTTGAAAAAACT
AGGTTCAGAATTTTTGGAAGAATTTTTTACGGAGGAAAAATCGATTCTTGATTTGGTC
TTCCCCCCGGCTTATTCTATTTCGCGACGGTTATACAGAGGGCGGGTTTGGTATTTGGA
TATTATTTGTATCAATGATCTAGCCAATCATGAATAAGATTAACTGCATTTTATATGTT
TCGGGTTTATTTACTTACTTTTGAAGGACATTTAAATCTTTATTTTCAAAATTATAGTG
GCAAAAAAAACAGTGCATTCTATTCAATATCTCTATGGGGTAAAGAAGGATCAAAAA
TACTAAAAAAAAAAAATTCCTTTATTA---------------
CCTTTATTAACAATTACTAATAATGAAAAGACTTCTTTTTTTTTGAACAAAATATATCG
AATTGATGTTAATGTAAGAAATATGATACGTCCCTCTATTACTAT------

TAATCATTTTTTCACGAAAAATATTTTTCCCTATCCCCCTGAAGCGGATAATACTATGT
TATTTCCTATGCTTGTGTTGGTACTATTTACTTTGTTTATTGGAGCTATAGGAATTCCTT
TCAATCAAGAGGGAACTAATTTGGATATATTATCCAAACTGTTAACTCCGTCTTTAAA
TCTTTTGCATCAAAATGAAAATAATTCTTTTGATTGGTATGAATTTATAACAAATGCTA
TTTTTTCAGTCATTATAACTTTTTTTGGAATATTTATAGCGTCTTCCTTTTATAAACCTA
TTTATTCATCGCTACAAAATTTGAACTTACTTAACTCATTTGCTAAAAATAAAAAA-----
----
AGGGGTCGTTGGGACAAAATAATGGGTGCGATATATGATTGGTCCTATAATCGTGGTT
ATATCGATGTTTTTTATACAATATTGGCTTCAAGGTTTCTCTTCATGAAAAACAAGGTA
CGGATGATATGCGATTGTCTGGCCCCTCCAGTCAAAGTAATCCAAGACGAGAGATTGC
CTCAGCCGTTAAGTCTTTGTGGGTCTACATTAAGATCTCCTCATGGTTGCCATGCTCAG
TATATGACAAATATGGGGACAATTGCATCTCTTGTGATGTCTGTGACTGTCAACGAAG
ATGATGATACAATGGATGGTGATCAGCAGCAGATGGCAAGAAAATTGTGGGGCTTAG
TTGTTTGTCATCATAGTAGTCCTCAATTTGTTCCGTTTCCTTTGAGGTATGCTTGTGAAT
TTTTGATTCAAGTGTTTGGTGTTCAGATCAACAAGGAGGTAGAGTTAGCTGCTCAGGT
GAGGGAAAAGCATATTTTACAAATTCAGACTATGCTATGCGACATGCTCCTTAGAGAT
GCTCCTGTTGCAATTATCACACAATCTCCGAATGTGATGGATCTTGTTAAGTGTGATG
GAGCTGCCCTATACTTCAAGAACAAAACTTGGTTTCTGGGAGTAACCCCAACAGAGG
AACAAATCAGAGGCATAGCTGAATGGCTGCTCAAATATCATAGTGGAAACACTGGTT
TAAGTACTGACAGCCTTATGGAAGCTGGATATCCAGGTGCTTCAGGTCTTGGTGATGC
AGTTTGTGGGATGGCTGCTGTTAGCATTACCTCGAGAGATTTTCTTTTTTGGTTCAGAT
CTCACACTGCTAAAGAGATCAAGTGGGGCGGTGCAAAACATGATCCTGATGACAAGG
ATGACTTAAGAAAGATGCATCCGAGGTCATCCTTCAAGGCCTTTCTTGAGGTGGTAAA
AATAAATTGACTTATTATACTCCTGACTATGAAACCAAAGATACTGATATCTTGGCAG
CATTCCGAGTAACTCCTCAACCTGGAGTTCCACCTGAGGAAGCAGGCGCTGCGGTAGC
TGCTGAATCTTCTACTGGTACATGGACAACTGTGTGGACCGATGGACTTACCAGTCTT
GATCGTTATAAAGGGCGATGCTACCATATCGAGCCCGTTGCTGGAGAAGAAAATCAA
TATATTGCTTATGTAGCTTACCCCTTAGACCTTTTTGAAGAAGGTTCTGTTACTAACAT
GTTTACTTCCATTGTGGGTAATGTATTTGGGTTCAAAGCCCTACGCGCTCTCCGTCTGG
AGGATTTGCGAATCCCTCCTGCTTATACGAAAACTTTCCAAGGCCCGCCTCATGGTAT
CCAAGTTGAGAGAGATAAATTGAACAAGTATGGTCGCCCCCTATTGGGCTGTACTATT
AAACCTAAATTGGGGTTATCTGCTAAGAATTACGGTAGAGCTGTTTATGAATGTCTAC
GCGGTGGACTTGATTTTACCAAAGACGACGAGAACGTGAACTCCCAACCATTTATGCG
TTGGAGAGACCGTTTCTTATTTTGTGCCGAAGCACTTTATAAAGCACAGGCGGAAACA
GGTGAAATCAAAGGGCATTATTTGAATGCTACTGCAGGTACATGCGAAGAAATGATC
AAAAGGGCTGTATGTGCCAGAGAATTGGGAGTTCCTATCGTAATGCATGACTACTTAA
CAGGTGGATTCACCGCAAATACTAGCTTGGCTCATTATTGCCGAGATAATGGTTTACT
TCTGCACATCCACCGCGCAATGCATGCAGTTATTGATAGACAGAAGAATCATGGTATG
CACTTTCGTGTACTAGCTAAAGCGTTACGCATGTCTGGTGGAGATCATATTCACGCGG
GTACCGTAGTAGGGAAACTTGAGGGGGAAAGAGAGATCACTTTAGGCTTTGTTGATTT
ACTGCGTGATGATTTTGTTGAAAAAGATAGAAGCCGCGGGATTTATTTCACTCAAGAC
TGGGTCTCCCTACCAGGTGTTATACCTGTGGCTTCGGGAGGTATTCACGTTTGGCATAT
GCCTGCTCTGACCGAGATCTTTGGAGATGATTCCGTACTACAATTCGGTGGCGGAACT
TTGGGGCACCCTTGGGGAAATGCACCAGGTGCCGTCGCTAATCGAGTAGCTCTAGAA
GCATGTGTACAAGCTCGTAATGAGGGACGTGATCTTGCTCGTGAGGGTAATGAAATTA
TCCGTGAGGCTAGCAAATGGAGTCCTGAACTAGCTGCTGCTTGTGAAGTGTGGAAAG
AGATTAA-
Janusia_hexandra --

ATTTAAATTATGTGTCTGATCCACTAATACCTTACCCCATCCATCTAGAAAAATTGGTT
CAAATCCTTCGTTACTGGATGCAAGATCCCTCTTCTTTGCATTTATTACGACTCTTTCTT
CACGAGTATTGGAATTGGAATAGTTTTCTTATTTCAACGAAATCCTTTTCCATTTGTTT

GAATTTAAAAAGGAATCTAAGATTATTGTTGTTCCTATATAATTCTCATGTATATGAAT
ACGAATCCATCTTCTTTTTCTCCGTAACCAATCCTTTTCTTTCCGATCAACATTTTTGG
GGGTGCTTCTTGAACGAATATATTTTTATGGAAAAATACAAGATTTTGTAGCACTTTTT
ACTAATG------
ATTTTAAGGCTAACCTACGGTTGTTCAACGATCCTTTCATGCATTATGTTAGATATCAA
GGAAAGGGCATTCTGGCTTCAAAAAGTACACTTCTTCTGATGAAAAAATGGAAATATT
TCTTTGTCAATTTATGTCAATTTCATTTTTATGTGTGGGTTCATCCAAAACCGATTCAT
ATGAAGTCATTATCCAACCATTCTCTTGACTTTTTGGGTTATCTTTCAAGTATACGAAA
GAATCTTTCAGTGGTACGGAGTCAAATGCTAGAAAATGCATTTCTAATAGATAAGATT
ATGAAGAAACTTGATTCAAGAATTCCAATTATTCCTTTGATTGGATCATTGTCAAAAA
CCAAATTTTGTAACGCCGTAGGACATCCCATTAGTAAACCGAGCTGGGCGGATTCGCC
CGATTCTGATATTATCGACCAATTTTTGCGTATATCCAGAAATTTTTCTCATTACTATA
GCGGATCCTCAAAAAAAAAGAGTTTGTATCGAGTAAAATATATACTTCGACTTTCTTG
TGTTAAAACTTTGGCTCGTAAACACAAAACGACCGTCCGGTCTTTTTTGAAAAAACTA
GGTTCAGAATTATTGGAAGAATTTTTTACGGAGGAAAAATCGATTCTTGATTTGGTCT
TCCCCCCGATTTATTCTATTTCGCGAAGGTTATACAGAGGGCGGGTTTGGTATTTGGAT
ATTATTTGTATCAATGATCTAGCCAATCATGAATAAGATTAACTGCATTTTATATGTTT
CGGGTTTATTTACTTACTTTTGAAGGACATTTTAATCTTTATTTTCAAAATTATAGTGG
CAAAAAAAACAGTGCATTCTATTCAATATCTCTATGGGGTAAAGAAGGATCAAAAAT
ACTAAAAAAAAAAAATTCCTTTATTACCTTTATTAACAATTCCTTTATTAACAATTACTA
ATAATGAAAAGACTTCTTTTTTTTTGAACAAAATATATCGAATTGATGGTAATGTAAG
AAATATGATACGTCCCTCTATTACTAT------
TAATCATTTTTTCACGAAAAATATTTTTCCCTATCCCCCTGAAGCGGATAATACTATGT
TATTTCCTATGCTTGTGTTGGTACTATTTACTTTGTTTATTGGAGCTATAGGAATTCCTT
TCAATCAAGAGGGAACTCATTTTGATATATTATCCAAACTGTTAACTCCGTCTTTAAAT
CTTTTGCATCAAAATGAAAAGAATTCTTTTGATTGGTATGAATTTATAACAAATGCTG
TTTTTTCAGTCATTATAACTTTTTTTGGAATATTTATAGCGTCTTCCTTTTATAAACCTA
TTTATTCATCGCTACAAAATTTGAACTTACTTAACTCATTTGCTAAAAATAAAAAA-----
----
AGGGGTCGTTGGGACAAAATAATGGGTGCGATATATGATTGGTCCTATAATCGTGGTT
ATATTGATGTTTTTTATACAATATT??????????????????????????????GGTACGGATGATAT
GCGATTGTCTGGCCCCTCCAGTCAAAGTAATCCAAGACGAGAGATTGCCTCAGCCATT
AAGTCTTTGTGGGTCTACATTAAGATCTCCTCATGGTTGCCATGCTCAGTATATGACA
AATATGGGGACAATTGCATCTCTTGTGATGTCTGTGACTGTCAACGAAGATGATGATA
CGATGGATGGTGATCAGCAGCAGATGGCAAGAAAATTGTGGGGCTTAGTTGTTTGTC
ATCATACTAGTCCTCAGTTTGTTCCATTTCCTTTGAGGTATGCTTGTGAATTTTTGATTC
AAGTGTTTGGTGTCCAGATCAACAAGGAAGTAGAGTTAGCTGCTCAGGTGAGGGAAA
AGCATATTTTACAAATTCAGACTATGCTATGCGACATGCTCCTTAGAGATGCTCCTGTT
GCAATTATCACACAATCTCCGAATGTGATGGATCTTGTTAAGTGTGATGGAGCTGCCC
TATACTTCAAGAACAAAACTTGGTTTCTGGGAGTAACCCCAACAGAGGAACAAATCA
GAGGCATAGCTGAATGGCTGCTCAAATATCATAGTGGAAACACTGGTTTAAGTACTG
ACAGCCTTATGGAAGCTGGATATCCAGGTGCTTCAGCTCTTGGTGATGCAGTTTGTGG
GATGGCTGCTGTTAGCATTACATCAAGAGATTTTCTTTTTTGGTTCAGATCTCACACTG
CTAAAGAGATCAAGTGGGGCGGTGCAAAACATGATCCTGATGACAAGGATGACTTAA
GAAAGATGCATCCGAGGTCATCTTTCAAGGCCTTCCT?????????????ATAAATTGACTTA
TTATACTCCTGACTATGAAACCAAAGATACTGATATCTTGGCAGCATTCCGAGTAACT
CCTCAACCTGGAGTTCCACCTGAGGAAGCAGGCGCTGCGGTAGCTGCTGAATCTTCTA
CTGGTACATGGACAACTGTGTGGACCGATGGACTTACCAGTCTTGATCGTTATAAAGG
GCGATGCTACCATATCGAGCCCGTTGCTGGAGAAGAAAATCAATATATTGCTTATGTA
GCTTACCCCTTAGACCTTTTTGAAGAAGGTTCTGTTACTAACATGTTTACTTCCATTGT
GGGTAATGTATTTGGGTTCAAAGCCCTACGCGCTCTCCGTCTGGAGGATTTGCGAATC
CCTCCTGCTTATACGAAAACTTTCCAAGGCCCGCCTCATGGTATCCAAGTTGAGAGAG
ATAAATTGAACAAGTATGGTCGCCCCCTATTGGGCTGTACTATTAAACCTAAATTGGG
GTTATCTGCTAAGAATTACGGTAGAGCTGTTTATGAATGTCTACGCGGTGGACTTGAT
TTTACCAAAGACGACGAGAACGTGAACTCCCAACCATTTATGCGTTGGAGAGACCGTT
TCTTATTTTGTGCCGAAGCACTTTATAAAGCACAGGCGGAAACAGGTGAAATCAAAG
GGCATTATTTGAATGCTACTGCAGGTACATGCGAAGAAATGATCAAAAGGGCTGTAT
GCGCCAGAGAATTGGGAGTTCCTATCGTAATGCATGACTACTTAACAGGCGGATTCAC
CGCAAATACTAGCTTGGCTCATTATTGCCGAGATAATGGTTTACTTCTGCACATCCAC
CGCGCAATGCATGCAGTTATTGATAGACAGAAGAATCATGGTATGCACTTTCGTGTAC

TAGCTAAAGCGTTACGCATGTCTGGTGGAGATCATATTCACGCGGGTACCGTAGTAGG
GAAACTTGAGGGGGAAAGAGAGATCACTTTAGGCTTTGTTGATTTACTGCGTGATGAT
TTTGTTGAAAAAGATAGAAGCCGCGGGATTTATTTTACTCAAGACTGGGTCTCCCTAC
CAGGTGTTATACCTGTGGCTTCGGGAGGTATTCACGTTTGGCATATGCCTGCTCTGAC
CGAGATCTTTGGAGATGATTCCGTACTACAATTCGGTGGCGGAACTTTGGGGCACCCT
TGGGGAAATGCACCAGGTGCCGTCGCTAATCGAGTAGCTCTAGAAGCATGTGTACAA
GCTCGTAATGAGGGACGTGATCTTGCTCGTGAGGGTAATGAAATTATCCGTGAGGCTA
GCAAATGGAGTCCTGAACTAGCTGCTGCTTGTGAAGTGTGGAAAGAGATTAA-
Peixotoa_cordistipula --

ATTTAAATTATGTGTCTGATCCACTAATACCTTACCCCATCCATCTAGAAAAATTGGTT
CAAATCCTTCGTTACTGGATGCAAGATCCCTTTTCTTTGCATTTGTTACGACTCTTTCTT
CACGAGTATTGGAATTGGAATAGTTTTATTATTTCAACGAAATCCTTTTCCATTTTTTT
TAATTTAAAAAGGAATCTAAGATTATTGTTGTTCCTATATAATTCTCATGTATATGAAT
ACGAATCCATCTTCTTTTTTCTCCGTAACCAATCCTTTTATTTCCGATCAACATTTTCGG
GGGTGCTTCTTGAACGAATATATTTCTATGGAAAAATACAAGATTTTGTAGAACTTTT
TACTAATG------
ATTTTAAGGCTAACCTACGGTTGTTTAAGGATCCTTTCATGCATTATGTTAGATATCAA
GGAAAAGGCATTCTGGCTTCAAAAAGTATACTTCTTCTGATGAAAAAATGGAAATATT
TCTTTGTCAATTTATGTCAATTTCATTTTTATGTGTGGGTTCATCCAGAACCGATCTAT
ATGAAGTCACTATCCAACCATTCTCTTGACTTTTTGGGTTATCTTTCAAGTATACGAAA
AAATCTTTCGGTGGTACGGAGTCAAATGCTAGAAAATTCATTTCTAATAGATAATATT
ATGAAGAAACTCGATTCAAGAGTTCCAATTATTCCTTTGATTGGATCATTGTCAAAAA
CAAAATTTTGTAACGCAGTAGGATATCCCATTAGTAAACCGAGCTGGGCGGATTCACC
CGATTCTGAGATTATCGACCAATTTTTGCGTATATCCAGAAATCTTTCTCATTACTATA
GCGGATCCTCAAAAAAAAAGAGTTTGTATCGAGTAAAATATATACTTCGACTTTCTTG
TGTTAAAACTTTGGCTCGTAAACACAAAAGTACCGTCCGGGCTTTTTTGAAAAAACTA
GGTTCAGAATTATTGGAAGAATTTTTTACAGAGGAAAAATCGATTCTTGATTTGGTCT
TCCCCACGGCTTATTCTATTTCGCGAAGGTTATACAGAGGGCGGGTTTGGTATTTGGA
TATTATTTGTATCAATGATCTAGCCAATCATGAATAAGATTAACTGCATTTTATATGTT
TCGGGTTTATTTACTTACTTTTGAAGGACATTTAAATCTTTATTTTCAAAATTACAGTG
GCAAAAAAAACAGTGCATTCTATTCATTATCTCTATGGGGTAAAGAAGGATCAAAAA
TACTAAAAAAAAAAAATTCCTTTATT?---------------
CCTTTATTAACAATCACTAATAATGAAAAG?CTTCTTTTTTTTTGAACAAAATATACCA
AATTGATGGTAATGTAAGAAATATGACGCGTCCCTCTATTACTAT------
TAATCATTTTTTCACGAAAAATATTTTTCCTTATCCCCTTGAAGCGGATAATACTATGT
TATTTCCTATGCTTGTGTTGGTACTATTTACTTTGTTTATTGGAGCTATAGGAATTCCTT
TCAATCAAGAGGGAACTAATTTGGATATATTATCCAAACTGTTAACTCCGTCTTTAAA
TCTTTTGCATCAAAATGAAAATAATTCTTTTGATTGGTATGAATTTATAACAAATGCTA
TTTTTTCAGTCATTATAACTTTTTTTTGGAATATTTATAGCGTCTTCCTTCTATAAACCTA
TTTATTCATCGCTACAAAATTTGAACTTACTTAACTCATTTGCTA------AAAAA---------
AGGGGTCGTTGGGACAAAATAATAGGTGCGATATATGATTGGTCCTATAATCGTGGTT
ATATCGATGTTTTTTATACAATATT????????????????????????????????????ACGCATGATATG
CGATTGTCTGGCCCCTCCAGTCCAAGTAATCCAAGATGAGAGATTGCCTCAGCCGTTA
AGTCTTTGTGGGTCTACATTAAGATCTCCTCATGGTTGCCATGCTCAGTATATGACAA
ATATGGGGACAATTGCATCTCTTGTGATGTCTGTGACTATCAATGAAGATGATGATAC
CATGGATGGTGATCAGCAGCAGATGACAAGAAAATTGTGGGGCTTAGTTGTTTGTCAT
CATACTAGTCCCCGATTTGTTCCGTTTCCTTTGAGGTATGCTTGTGAATTTTTGATTCA
AGTGTTTGGTGTTCAGATCAACAAGGAGGTAGAGTTAGCTGCTCAGGTGAGGGAAAA
ACATATTTTACAAATTCAGACTATGCTATGCGACATGCTGCTTAGAGATGCTCCCGTT
GCAATTATCACACAATCTCCGAATGTGATGGATCTTGTTAAGTGTGATGGAGCTGCCC
TATACTTCAAGAACAAAACTTGGTTGCTGGGAGTAACCCCTACAGAGGAACAAATCA

GAGACATAGCTGAATGGCTGCTCAAATATCATAGTGGAAACACAGGTTTAAGTACTG
ACAGCCTTATGGAAGCTGGATATCCAGGTGCTTCAACTCTCGGTGATGCAGTTTGTGG
GATGGCAGTTGTTAGTATTACTTCGAGAGATTTTCTTTTTTGGTTTAGATCTCACACTG
CTAAAGAGATCAAGTGGGGTGGTGCAAAACATGATCCTGATGACAAAGATGACTTAA
GAAAGATGCATCCAAGGTCATCCTTCAAGGCCTTTCTAGAG?????????ATAAATTGACT
TATTATACTCCTGACTATGAAACCAAAGATACTGATATCTTGGCAGCATTCCGAGTAA
CTCCCCAACCTGGAGTTCCGCCTGAGGAAGCAGGCGCTGCGGTAGCTGCTGAATCTTC
TACTGGTACATGGACAACTGTGTGGACCGATGGACTTACCAGTCTTGATCGTTATAAA
GGGCGATGCTACCACATCGAGCCCGTTGCTGGAGAAGAAAATCAATATATTGCTTATG
TAGCTTACCCCTTAGACCTTTTTGAAGAAGGTTCTGTTACTAACATGTTTACTTCCATT
GTGGGTAATGTATTTGGGTTCAAAGCCCTACGCGCTCTCCGTCTGGAGGATTTGCGAA
TCCCTCCTGCTTATACGAAAACTTTCCAAGGCCCGCCTCATGGTATCCAAGTTGAGAG
AGATAAATTGAACAAGTATGGTCGCCCCCTATTGGGCTGTACTATTAAACCTAAATTG
GGGTTATCTGCTAAGAATTACGGTAGAGCTGTTTATGAATGTCTACGCGGTGGACTTG
ATTTTACCAAAGACGACGAGAACGTGAACTCCCAACCATTTATGCGTTGGAGAGACC
GTTTCTTATTTTGTGCCGAAGCACTTTATAAAGCACAGGCGGAAACAGGTGAAATCAA
AGGGCATTATTTGAACGCTACTGCAGGTACATGCGAAGAAATGATCAAAAGGGCTGT
ATTTGCCAGAGAATTGGGAGTTCCTATCGTAATGCATGACTACTTAACAGGGGGATTC
ACCGCAAATACTAGCTTGGCTCATTATTGCCGAGATAATGGTTTACTTCTGCACATCC
ATCGCGCAATGCATGCAGTTATTGATAGACAGAAGAATCATGGTATGCACTTTCGTGT
ACTAGCTAAAGCGTTACGCATGTCTGGGGGAGATCATATTCACGCGGGTACCGTAGTA
GGGAAACTTGAGGGGGAAAGAGAGATCACTTTAGGCTTTGTTGATTTACTGCGTGATG
ATTTTGTTGAAAAAGATAGAAGCCGCGGGATTTATTTCACTCAAGACTGGGTCTCCCT
ACCAGGTGTTATACCCGTGGCTTCGGGAGGTATTCACGTTTGGCATATGCCTGCTCTG
ACCGAGATCTTTGGAGATGATTCCGTACTACAATTCGGTGGAGGAACTTTGGGGCACC
CTTGGGGAAATGCACCAGGTGCCGTAGCTAATCGAGTAGCTCTAGAAGCATGTGTAC
AAGCTCGTAATGAGGGACGTGATCTTGCTCGTGAGGGTAATGAAATTATCCGTGAGG
CTAGCAAATGGAGTCCTGAACTAGCTGCTGCTTGTGAAGTGTGGAAAGAGATTAA-
Peixotoa_glabra ---

ATTTAAATTATGTGTCTGATCCACTAATACCTTACCCCATCCATCTAGAAAAATTGGTT
CAAATCCTTCGTTACTGGATGCAAGATCCCTTTTCTTTGCATTTGTTACGACTCTTTCTT
CACGAGTATTGGAATTGGAATAGTTTTATTATTTCAACGAAATCCTTTTCCATTTTTTT
TAATTTAAAAAGGAATCTAAGATTATTGTTGTTCCTATATAATTCTCATGTATATGAAT
ACGAATCCATCTTCTTTTTTCTCCGTAACCAATCCTTTTATTTACGATCAACATTTTCGG
GGGTGCTTCTTGAACGAATATATTTCTATGGAAAAATA????????????????????????????----
-
-??
??????????ATGAAAAAATGGAAATATTTCTTTGTCAATTTATGTCAATTTCATTTTTATG
TGTGGGTTCATCCAGAACCGATCTATATGAAGTCACTATCCAACCATTCTCTTGACTTT
TTGGGTTATCTTTCAAGTATACGAAAAAATCTTTCGGTGGTACGGAGTCAAATGCTAG
AAAATTCATTTCTAATAGATAATATTATGAAGAAACTCGATTCAAGAGTTCCAATTAT
TCCTTTGATTGGCTCATTGTCAAAAACAAAATTTTGTAACGCAGTAGGATATCCCATT
AGTAAACCGAGCTGGGCGGATTCACCCGATTCTGAGATTATCGACCAATTTTTGCGTA
TATCCAGAAATCTTTCTCATTACTATAGCGGATCCTCAAAAAAAAAAGAGTTTGTATCG
AGTAAAATATATACTTCGACTTTCTTGTGTTAAAACTTTGGCTCGTAAACACAAAAGT
ACCGTCCGGGCTTTTTTGAAAAAACTAGGTTCAGAATTATTGGAAGAATTTTTTACAG
AGGAAAAATCGATTCTTGATTTGGTCTTCCCCACGGCTT??????????????????????????????
???GAATAACTGCATTTTATATGTTTC
GGGTTTATTTACTTACTTTTGAAGGACATTTAAATCTTTATTTTCAAAATTACAGGGGA
AAAAAAAACAGTGCATTCTATTCACTATCTCTATGGGGTAAAGAAGGATCAAAAATA
CTAAAAAAAAAAAATTCCTTTATTA---------------

CCTTTATTAACAATCACTAATAATGAAAAGACTTCTTTTTTTTGAACAAAATATACCA
AATTGATGGTAATGTAAGAAATATGACGCGTCCCTCTATTACTAT------
TAATCATTTTTTCACGAAAAATATTTTTCCTTATCCCCTTGAAGCGGATAATACTATGT
TATTTCCTATGCTTGTGTTGGTACTATTTACTTTGTTTATTGGAGCTATAGGAATTCCTT
TCAATCAAGAGGGAACTAATTTGGATATATTATCCAAACTGTTAACTCCGTCTTTAAA
TCTTTTGCATCAAAATGAAAATAATTCTTTTGATTGGTATGAATTTATAACAAATGCTA
TTTTTTCAGTCATTATAACTTTTTTTGGAATATTTATAGCGTCTTCCTTCTATAAACCTA
TTTATTCATCGCTACAAAATTTGAACTTACTTAACTCATTTGCTA------AAAAA---------
AGGGGTCGTTGGGACAAAATAATAGGTGCGATATATGATTGGTCCTATAATCGTGGTT
ATATCGATGCTTTTTATGCAATATTAGCTTCAAGGTTTCTCTTCATGAAAAACAAGGTA
CGCATGATATGCGATTGTCTGGCCCCTCCAGTCCAAGTAATCCAAGACGAGAGATTGC
CTCAGCCGTTAAGTCTTTGTGGGTCTACATTAAGATCTCCTCATGGTTGCCATGCTCAG
TATATGACAAATATGGGGACAATTGCATCTCTTGTGATGTCTGTGACTGTCAACGAAG
ATGACGATACCATGGATGGTGATCAGCAGCAGATGACAAGAAAATTGTGGGGCTTAG
TTGTTTGTCATCATACTAGTCCCCGATTTGTTCCGTTTCCTTTGAGGTATGCTTGTGAAT
TTTTGATTCAAGTGTTTGGTGTTCAGATCACCAAGGAGGTAGAGTTAGCTGCTCAGGT
GAGGAAAAACCATACTTTACAAATTCAGACTATGCTATGCGACATGCTGCTTAGAGAT
GCTCCCGTTGCAATTATCACACAATCTCCGAATGTGATGGATCTTGTTAAGTGTGATG
GAGCTGCCCTATACTTCAAGAACAAAACTTGGTTGCTGGGAGTAACCCCTACAGAGG
AACAAATCAGAGACATAGCTGAATGGCTGCTCAAATATCATAGTGGAAACACAGGTT
TAAGTACTGACAGCCTTATGGAAGCTGGATATCCAGGTGCTTCAACTCTCGGTGATGC
AGTTTGTGGGATGGCAGCTGTTAGTATTACTTCGAGAGATTTTCTTTTTTGGTTTAGAT
CTCACACTGCTAAAGAGATCAAGTGGGGTGGTGCAAAACATGATCCTGATGACAAAG
ATGACTTAAGAAAGATGCATCCAAGGTCATCCTTCAAGGCCTTTCTAGAGGTGGTAAA
AATAAATTGACTTATTATACTCCTGACTATGAAACCAAAGATACTGATATCTTGGCAG
CATTCCGAGTAACTCCTCAACCTGGAGTTCCGCCTGAGGAAGCAGGTGCTGCGGTAGC
TGCTGAATCTTCTACTGGTACATGGACAACTGTGTGGACCGATGGGCTTACCAGTCTT
GATCGTTATAAAGGGCGATGCTACCACATCGAGGCCGTTGTTGGAGAAGAAAATCAA
TATATTGCTTATGTAGCTTACCCCTTAGACCTTTTTGAAGAAGGTTCTGTTACTAACAT
GTTTACTTCCATTGTGGGTAATGTATTTGGGTTCAAAGCCCTACGCGCTCTCCGTCTGG
AGGATTTGCGAATCCCTCCTGCTTATACGAAAACTTTCCAAGGCCCGCCTCATGGTAT
CCAAGTTGAGAGAGATAAATTGAACAAGTATGGTCGCCCCCTATTGGGCTGTACTATT
AAACCTAAATTGGGGTTATCCGCTAAGAATTACGGTAGAGCTGTTTATGAATGTCTAC
GCGGTGGACTTGATTTTACCAAAGACGACGAGAACGTGAACTCCCAACCATTTATGCG
TTGGAGAGACCGTTTCTTATTTTGTGCCGAAGCAATTTATAAAGCACAGGCGGAAACA
GGTGAAATCAAAGGGCATTATTTGAATGCTACTGCAGGTACATGCGAAGAAATGATC
AAAAGGGCTGTATTTGCCAGAGAATTGGGAGTTCCTATCGTAATGCATGACTACTTAA
CAGGGGGATTCACCGCAAATACTAGCTTGGCTCATTATTGCCGAGATAATGGTTTACT
TCTGCACATCCACCGCGCAATGCATGCAGTTATTGATAGACAGAAGAATCATGGTATG
CACTTTCGTGTACTAGCTAAAGCGTTACGCCTGTCTGGTGGAGATCATATTCACGCGG
GTACTGTAGTAGGGAAACTTGAGGGGGAAAGAGAGATCACTTTAGGCCTTGTTGATTT
ACTGCGTGATGATTTTGTTGAAAAAGATAGAAGCCGCGGGATTTATTTCACTCAAGAC
TGGGTCTCCCTACCAGGTGTTATACCTGTGGCTTCGGGAGGTATTCACGTTTGGCATAT
GCCTGCTCTGACCGAGATCTTTGGAGATGATTCCGTACTACAATTCGGTGGAGGAACT
TTGGGGCACCCTTGGGGAAATGCACCAGGTGCCGTAGCTAATCGAGTAGCTCTAGAA
GCATGTGTACAAGCTCGTAATGAGGGACGTGATCTTGCTCGTGAGGGTAATGAAATTA
TCCGTGAAGCTAGCAAATGGAGTCCTGAACTAGCTGCTGCTTGTGAAGTGTGGAAAG
AGATTAA-
Philgamia_glabrifolia --

ATTTAAATTATGTGTCTGATTCACTAATACCTTACCCCATCCATCTAGAAAAATTGGTT
CAAATCCTTCGTTACTGGATGCAAGATCCCTCTTCTTTGCATTTATTACGACTCTTTCTT

CACGAGTATTGGAATTGGAATAGTTTTATTATTTCAACGAAATCCTTTTCCATTTTTTT
TAATTTAAAAAGGAATCTAAGATTATTGTTGTTCCTATATAATTCTCATGTATATGAAT
ACGAATCCATCTTCTTTTTTCTCCGTAACCAATCCTTTTATTTCCAATCAACATTTTCGG
GGGTGCTTCTTGAACGAATATATTTCTATGGAAAAATACAAGATTTTGTAGAATTTTTT
ACTAATG------
ATTTTAAGGCTAACCTACGGTTGTTCAAGGATCCTTTCATGCATTATGTTAGATATCAA
GGAAAAGGCATTCTGGCTTCAAAAAGTGCACTTCTTCTGATGAAAAAATGGAAATATT
TCTTTGTCAATTTATGTCAATTTCATTTTTATGTGTGGGTTCATCCAGAACCGATCTAT
ATGAAGTCATTATCCAACCATTCTCTTGACTTTTTGGGTTATCTTTCAAGTATACGAAA
AAATCTTTCAGTGGTACGGAGTCAAATGCTAGAAAATTCATTTCTAATAGATAATATT
ATGAAGAAACTCGATTCAAGAGTTCCAATTATTCCTTTGATTGGATCATTGTCAAAAA
CAAAATTTTGTAACGCAGTAGGGCATCCCATTAGTAAACCGAGCTGGGCCGATTCACC
CGATTCTGATATTATCGACCAATTTTTGCGTATATCCAGAAATCTTTCTCATTACTATA
GCGGGTCCTCAAAAAAAAAGAGTTTGTATCGAGTAAAATATATACTTCGACTTTCTTG
TGTTAAAACTTTGGCTCGTAAACACAAAAGTACCGTCCGGGCTTTTTTGAAAAAACTA
GGTTCAGAATTATTGGAAGAATTTTTTACGGAGGAAAAATCGATTCTTGATTTGGTCT
TCCCCACGGCTTATTCTATTTCGCGAAGGTTATACAGAGGGCGGGTTTGGTATTTGGA
TATTATTTGTATCAATGATCTAGCCAATCATGAATAAGATTAACTGCATTTTATATGTT
TCGGGTTTATTTACTTACTTTTGAAGGACATTTAAATCTTTATTTTCAAAATTACAGTG
GCAAAAAAAACAGTGCATTCTATTCAATATCTCTATGGGGTAAAGAAGGATCAAAAA
TACTAAAAAAAAAAAATTCCTTTATTA---------------
CCTTTATTAACAATCACTAATAATGAAAAGACTTCTTTTTTTTTGAACAAAATATACCA
AATTGATGGTAATGTAAGAAATATGACGCGTCCCTCTATTACTAT------
TAATCATTTTTTCACGAAAAATATTTTTCCCTATCCCCCTGAAGCGGATAATACTATGT
TATTTCCTATGCTTGTGTTGGTACTATTTACTTTGTTTATTGGAGCTATAGGAATTCCTT
TCAATCAAGAGGGAACTAATTTGGATATATTATCCAAACTGTTAACTCCGTCTTTAAA
TCTTTTGCATCAAAATGAAAATAATTCTTTTGATTGGTATGAATTTATAACAAATGCTA
TTTTTTCAGTCATTATAACTTTTTTTGGAATATTTATAGCGTCTTCCTTCTATAAACCTC
TTTATTCATCGCTACAAAATTTGAACTTACTTAACTCATTTGCTA------AAAAA---------
AGGGGTCGTTGGGACAAAATCATAGGTGCGATATATGATTGGTCCTATAATCGTGGTT
ATATCGATGTTTTTTATGCAATATTAGCTTCAAGGTTTCTCTTCATGAAAAACAAGGTA
CGCATGATATGCGATTGTCTGGCCCCTCCAGTCAAGGTAATCCAAGACGAGAGATTGC
CTCAGCCATTAAGTCTTTGTGGGTCTACATTAAGATCTCCTCATGGCTGCCATTCTCAG
TATATGACAAATATGGGGACAATTGCATCGCTTGTGATGTCTGTGACTATCAACGAAG
ATGATGATACGATGGATGGTGATCAGCAGCAGATGACAAGAAAATTGTGGGGCTTAG
TTGTTTGTCATCATACTAGTCCTCGGTTTGTTCCGTTTCCTTTGAGGTATGCCTGTGAAT
TTTTGATTCAAGTGTTTGGTGTTCAGATCAACAAGGAGGTAGAATTAGCTGCTCAGGT
GAGGGAAAAACATATTTTACAAATTCAGACTATGCTATGCGACATGCTCCTTAGAGAT
GCTCCAGTTGCAATTATCACACAATCTCCGAATGTGATGGATCTTGTTGAGTCTGATG
GAACTGCCTTATACTTCAAGAACAAAACTTGGTTGCTCGGGGTAACCCCTACAGAGGA
ACAAATCAGAGACATAGCAGAATGGCTGCTCCAATATCATAGCGGAAACACCGGTTT
AAGTACTGACAGCCTTATGGAAGCTGGATATCCAGGTGCTTCAGCTCTTGGTGATTCA
GTTTGTGGGATGGCTGCTGTTAGCATTACTTCGAGGGATTTTCTTTTTTGGTTTAGATC
TCACACTGCTAAAGAGATCAAGTGGGGCGGTGCAAAACATGATCCTGATGACAAAGA
TGACTTAAGAAAGATGCATCCGAGGTCATCCTTCAAGGCCTTTCTAGAGGTGGTAAAA
ATAAATTGACTTATTATACTCCTGACTATGAAACCAAAGATACTGATATCTTGGCAGC
ATTCCGAGTAACTCCTCAACCTGGAGTTCCGCCTGAGGAAGCAGGCGCTGCGGTAGCT
GCTGAATCTTCTACTGGTACATGGACAACTGTGTGGACCGATGGACTTACCAGTCTTG
ATCGTTATAAAGGGCGATGCTACCACATCGAGCCCGTTGCTGGAGAAGAAAATCAAT
ATATTGCTTATGTAGCTTACCCCTTAGACCTTTTTGAAGAAGGTTCTGTTACTAACATG
TTTACTTCCATTGTGGGTAATGTATTTGGGTTCAAAGCCCTACGCGCTCTCCGTCTGGA
GGATTTGCGAATCCCTCCTGCTTATACGAAAACTTTCCAAGGCCCGCCTCATGGTATC
CAAGTTGAGAGAGATAAATTGAACAAATATGGTCGCCCCCTATTGGGCTGTACTATTA
AACCTAAATTGGGGTTATCTGCTAAGAATTACGGTAGAGCTGTTTATGAATGTCTACG
CGGTGGACTTGATTTTACCAAAGACGACGAGAACGTGAACTCCCAACCATTTATGCGT
TGGAGAGACCGTTTCTTATTTTGTGCCGAAGCAATTTATAAAGCACAGGCGGAAACAG
GTGAAATCAAAGGGCATTATTTGAATGCTACTGCAGGTACATGCGAAGAAATGATCA
AAAGGGCTGTATTTGCCAGAGAATTGGGAGCTCCTATCGTAATGCATGACTACTTAAC
AGGGGGATTCACCGCAAATACTAGCTTGGCTCATTATTGCCGAGATAATGGTTTACTT
CTGCACATCCATCGCGCAATGCATGCAGTTATTGATAGACAGAAGAATCATGGTATGC

ACTTTCGTGTACTAGCTAAAGCGTTACGCATGTCTGGTGGAGATCATATTCACGCGGG
TACCGTAGTAGGGAAACTTGAGGGGGAAAGAGACATCACTTTAGGCTTTGTTGATTTA
CTGCGTGATGATGTTATTGAAAAAGATAGAAGCCGCGGGATTTATTTCAGTCAAGACT
GGGTCTCCCTACCAGGTGTTATACCCGTGGCTTCGGGAGGTATTCACGTTTGGCATAT
GCCTGCTCTGACCGAGATCTTTGGAGATGATTCCGTACTACAATTCGGTGGGGGAACT
TTGGGGCACCCTTGGGGAAATGCACCAGGTGCCGTAGCTAATCGAGTAGCTCTAGAA
GCATGTGTACAAGCTCGTAATGAGGGACGTGATCTTGCTCGTGAGGGTAATGAAATTA
TCCGTGAGGCTAGCAAATGGAGTCCTGAACTAGCTGCTGCTTGTGAAGTGTGGAAAG
AGATTAA-
Philgamia_hibbertioides --

ATTTAAATTATGTGTCTGATTCACTAATACCTTACCCCATCCATCTAGAAAAATTGGTT
CAAATCCTTCGTTACTGGATGCAAGATCCCTCTTCTTTGCATTTATTACGACTCTTTCTT
CACGAGTATTGGAATTGGAATAGTTTTATTATTTCAACGAAATCCTTTTCCATTTTTTT
TAATTTAAAAAGGAATCTAAGATTATTGTTGTTCCTATATAATTCTCATGTATATGAAT
ACGAATCCATCTTCTTTTTTCTCCGTAACCAATCCTTTTATTTCCAATCAACATTTTCGG
GGGTGCTTCTTGAACGAATATATTTCTATGGAAAAATACAAGATTTTGTAGAATTTTTT
ACTAATG------
ATTTTAAGGCTAACCTACGGTTGTTCAAGGATCCTTTCATGCATTATGTTAGATATCAA
GGAAAAGGCATTCTGGCTTCAAAAAGTACACTTCTTCTGATGAAAAAATGGAAATATT
TCTTTGTCAATTTATGTCAATTTCATTTTTATGTGTGGGTTCATCCAGAACCGATCTAT
ATGAAGTCATTATCCAACCATTCTCTTGACTTTTTGGGTTATCTTTCAAGTATACGAAA
AAATCTTTCAGTGGTACGGAGTCAAATGCTAGAAAATTCATTTCTAATAGATAATATT
ATGAAGAAACTCGATTCAAGAGTTCCAATTATTCCTTTGATTGGATCATTGTCAAAAA
CAAAATTTTGTAACGCAGTAGGGCATCCCATTAGTAAACCGAGCTGGGCGGATTCACC
CGATTCTGATATTATCGACCAATTTTTGCGTATATCCAGAAATCTTTCTCATTACTATA
GCGGGTCCTCAAAAAAAAAAGAGTTTGTATCGAGTAAAATATATACTTCGACTTTCTTG
TGTTAAAACTTTGGCTCGTAAACACAAAAGTACCGTCCGGGCCTTTTTGAAAAAACTA
GGTTCAGAATTATTGGAAGAATTTTTTACGGACGAAAAATCGATTCTTGATTTGGTCT
TCCCCACGGCTTATTCTATTTCGCGAAGGTTATACAGAGGGCGGGTTTGGTATTTGGA
TATTATTTGTATCAATGATCTAGCCAATCATGAATAAGATTAACTGCATTTTATATGTT
TCGGGTTTATTTACTTACTTTTGAAGGACATTTAAATCTTTATTTTCAAAATTACAGTG
GCAAAAAAAACAGTGCATTCTATTCAATATCTCTATGGGGTAAAGAAGGATCAAAAA
TACTAAAAAAAAAAAATTCCTTTATTA---------------
CCTTTATTAACAATCACTAATAATGAAAAGACTTCTTTTTTTTTGAACAAAATATACCA
AATTGATGGTAATGTAAGAAATATGACGCGTCCCTCTATTACTAT------
TAATCATTTTTTCACGAAAAATATTTTTCCCTACCCCCCTGAAGCGGATAATACTATGT
TATTTCCTATGCTTGTGTTGGTACTATTTACTTTGTTTATTGGAGCTATAGGAATTCCTT
TCAATCAAGAGGGAACTAGTTTGGATATATTATCCAAACTGTTAACTCCGTCTTTAAA
TCTTTTGCATCAAAATGAAAATAATTCTTTTGATTGGTATGAATTTATAACAAATGCTA
TTTTTTCAGTCATTATAACTTTTTTTGGAATATTTATAGCGTCTTCCTTCTATAAACCTC
TTTATTCATCGCTACAAAATTTGAACTTACTTAACTCATTTGCTA------AAAAA---------
AGGGGTCGTTGGGACAAAATAATAGGTGCGATATATGATTGGTCCTATAATCGTGGTT
ATATCGATGTTTTTTATGCAATATTGGCTTCAAGGTTTCTCTTCATGAAAAACAAGGTA
CGCATGATATGCGATTGTCTGGCCCCTCCAGTCAAGGTAATCCAAGACGAGAGATTGC
CTCAGCCATTAAGTCTTTGTGGGTCTACATTAAGATCTCCTCATGGCTGCCATTCTCAG
TATATGACAAATATGGGGACAATTGCATCGCTTGTGATGTCTGTGACTATCAACGAAG
ATGATGATACGATGGATGGTGATCAGCAGCAGATGACAAGAAAATTGTGGGGCTTAG
TTGTTTGTCATCATACTAGTCCTCGGTTTGTTCCGTTTCCTTTGAGGTATGCTTGTGAAT
TTTTGATTCAAGTGTTTGGTGTTCAGATCAACAAGGAGGTAGAATTAGCTGCTCAGGT
GAGGGAAAAACATATTTTACAAATTCAGACTATGCTATGCGACATGCTCCTTAGAGAT
GCTCCAGTTGCAATTATCACACAATCTCCGAATGTGATGGATCTTGTTAAGTCTGATG

GAACTGCCTTATACTTCAAGAACAAAACTTGGTTGCTCGGGGTAACCCCTACAGAGGA
ACAAATCAGAGACATAGCAGAATGGCTGCTCCAATATCATAGCGGAAACACCGGTTT
AAGTACTGACAGCCTTATGGAAGCTGGATATCCAGGTGCTTCAGCTCTTGGTGATTCA
GTTTGTGGGATGGCTGCTGTTAGCATTACTTCGAGGGATTTTCTTTTTTGGTTTAGATC
TCACACTGCTAAAGAGATCAAGTGGGGCGGTGCAAAACATGATCCTGATGACAAAGA
TGACTTAAGAAAGATGCATCCGAGGTCATCCTTCAAAGCCTTTCTAGAGGTGGTAAAA
ATAAATTGACTTATTATACTCCTGACTATGAAACCAAAGATACTGATATCTTGGCAGC
ATTCCGAGTAACTCCTCAACCTGGAGTTCCGCCTGAGGAAGCAGGCGCTGCGGTAGCT
GCTGAATCTTCTACTGGTACATGGACAACTGTGTGGACCGATGGACTTACCAGTCTTG
ATCGTTATAAAGGGCGATGCTACCACATCGAGCCCGTTGCTGGAGAAGAAAATCAAT
ATATTGCTTATGTAGCTTACCCCTTAGACCTTTTTGAAGAAGGTTCTGTTACTAACATG
TTTACTTCCATTGTGGGTAATGTATTTGGGTTCAAAGCCCTACGCGCTCTCCGTCTGGA
GGATTTGCGAATCCCTCCTGCTTATACGAAAACTTTCCAAGGCCCGCCTCATGGTATC
CAAGTTGAGAGAGATAAATTGAACAAATATGGTCGCCCCCTATTGGGCTGTACTATTA
AACCTAAATTGGGGTTATCTGCTAAGAATTACGGTAGAGCTGTTTATGAATGTCTACG
CGGTGGACTTGATTTTACCAAAGACGACGAGAACGTGAACTCCCAACCATTTATGCGT
TGGAGAGACCGTTTCTTATTTTGTGCCGAAGCAATTTATAAAGCACAGGCGGAAACAG
GTGAAATCAAAGGGCATTATTTGAATGCTACTGCAGGTACATGCGAAGAAATGATCA
AAAGGGCTGTATTTGCCAGAGAATTGGGAGCTCCTATCGTAATGCATGACTACTTAAC
AGGGGGATTCACCGCAAATACTAGCTTGGCTCATTATTGCCGAGATAATGGTTTACTT
CTGCACATCCATCGCGCAATGCATGCAGTTATTGATAGACAGAAGAATCATGGTATGC
ACTTTCGTGTACTAGCTAAAGCGTTACGCATGTCTGGTGGAGATCATATTCACGCGGG
TACCGTAGTAGGGAAACTTGAGGGGGAAAGAGACATCACTTTAGGCTTTGTTGATTTA
CTGCGTGATGATGTTATTGAAAAAGATAGAAGCCGCGGGATTTATTTCAGTCAAGACT
GGGTCTCCCTACCAGGTGTTATACCCGTGGCTTCGGGAGGTATTCACGTTTGGCATAT
GCCTGCTCTGACCGAGATCTTTGGAGATGATTCCGTACTACAATTCGGTGGGGGAACT
TTGGGGCACCCTTGGGGAAATGCACCAGGTGCCGTAGCTAATCGAGTAGCTCTAGAA
GCATGTGTACAAGCTCGTAATGAGGGACGTGATCTTGCTCGTGAGGGTAATGAAATTA
TCCGTGAGGCTAGCAAATGGAGTCCTGAACTAGCTGCTGCTTGTGAAGTGTGGAAAG
AGATTAA-
Sphedamnocarpus_angolensis --

-
ATTTAAATTATGTGTCTGATTCACTAATACCTTACCCCATCCATCTAGAAAAATTGGTT
CAAATCCTTCGTTACTGGATGCAAGATCCCTCTTCTTTGCATTTATTACGACTCTTTCTT
CACGAGTATTGGAATTGGAATAGTTTTCTTATTTCAACGAAATCCTTTTCCATTTTTCT
TAATTTAAAAAGGAATCTAAGATTATTGTTGTTCCTATATAATTCTCATGTATATGAAT
ACGAATCCATCTTCTTTTTTCTCCGTAACCAATCCTTTTATTTCCAATCAACATTTTCGG
GGGTGCTTCTTGAACGAATATATTTCTATGGAAAAATACAAGATTTTGTAGAACTTTT
TACTAATG------
ATTTTAAGGCTAACCTACGGTTGTTCAAGGATCCTTTCATGCATTATGTTAGATATCAA
GGAAAAGGCATTCTGGCTTCAAAAAGTACACTTCTTCTGATGAAAAAATGGAAATATT
TCTTTGTCAATTTATGTCAATTTCATTTTTATGTGTGGGTTCATCCAGAACCGATCTAT
ATGAAGTCATTATCCAACCATTCTCTTGACTTTTTGGGTTATCTTTCAAGTATACGAAA
AAATCTTTCAGTGGTACGGAGTCAAATGCTAGAAAATTCATTTCTAATAGATAATATT
ATGAAGAAACTCGATTCAAGAGTTCCGATTATTCCTTTGATTGGATCATTGTCAAAAA
CAAAATTTTGTAACGCAGTAGGGCATCCCATTAGTAAACCGAACTGGGCGGATTCACC
CGATTCTGATATTATCGACCAATTTTTGCGTATATCCAGAAATCTTTCTCATTACTATA
GCGGATCCTCAAAAAAAAAGAGTTTGTATCGAGTAAAATATATACTTCGACTTTCTTG
TGTTAAAACTTTGGCTCGTAAACACAAAAGTACCGTCCGGGCTTTTTTGAAAAAACTA
GGTTCAGAATTATTGGAAGCATTTTTTACGGAGGAAAAATCGATTCTTGATTTGGTCT
TCCCCACGGCTTATTCTATTTCGCGAAGGTTATACAGAGGGCGGGTTTGGTATTTGGA

TATTATTTGTATCAATGATCTAGCCAATCATGAATAAGATTAACTGCATTTTATATGTT
TCGGGTTTATTTACTTACTTTTGAAGGACATTTAAATCTTTATTTTCAAAATTACAGTG
GCAAAAAAAAACAGTGCATTCTATTCAATATCTCTATGGGGTAAAGAGGGATCAAAAA
TACTAAAAAAAAAAAATTCCTTTATTA---------------
CCTTTATTAACAATCACTAATAATGAAAAGACTTCTTTTTTTTTGAACAAAATATACCA
AATTGATGGTAATGTAAGAAATATGACGCGTCCCTCTATTACTAT------
TAATCATTTTTTCACGAAAAATCTTTTTCCCTATCCCCCTGAAGCGGATAATACTATGT
TATTTCCTATGCTTGTGTTGGTACTATTTACTTTGTTTATTGGAGCTATAGGAATTCCTT
TCAATCAAGAGGGAACTAATTTGGATATATTATCCAAACTGTTAACTCCGTCTTTAAA
TCTTTTGCATCAAAATGAAAATAATTCTTTTGATTGGTATGAATTTATAACAAATGCTA
TTTTTTCAGTCATTATAACTTTTTTTGGAATATTTATAGCGTCTTCCTTCTATAAACCTC
TTTATTCATCGCTACAAAATTTGAACTTACTTAACTCATTTGCTA------AAAAA---------
AGGGGTCGTTGGGACAAAATAATAGGTGCGATATATGATTGGTCCTATAATCGTGGTT
ATATCGATGTTTTTTATGCAATATTAGCTTCAAGGTTTCTCTTCATGAAAAACAAGGTA
CGCATGATATGCGATTGTCTGGCCCCTCCAGTCAAGGTAATCCAAGACGAGAGATTGC
CTCTGCCATTAAGTCTTTGTGGGTCTACATTAAGATCTCCTCATGGCTGCCATTCTCAG
TATATGACAAATATGGGGACAATTGCATCACTTGTGATGTCTGTGACCATCAACGAAG
ATGATGATACGATGGATGGTGATCAGCAGCAGATGGCAAGAAAATTGTGGGGCTTAG
TTGTTTGTCATCATACTAGTCCTCGGTTTGTTCCGTTTCCTTTGAGGTATGCTTGTGAAT
TTTTGATTCAAGTGTTTGGTGTTCAGATCAACAAGGAGGTAGAATTAGCTGCTCAGGT
GAGGGAAAAACATATTTTACAAATTCAGACTATGCTATGCGACATGCTCCTTAGAGAT
GCTCCCGTTGCAATTATCACACAATCTCCGAATGTGATGGATCTTGTTAAGTGTGATG
GAGCTGCCTTATACTTCAAGAACAAAACTTGGTTGCTGGGAGTAACCCCTACAGAGG
AACAAATCAGAGACATAGCAGAATGGCTGCTCCAATATCATAGCGGAAACACGGGTT
TAAGTACTGACAGCCTTATGGAAGCTGGATATCCAGGTGCTTCAGCTCTTGGTGATTC
AGTTTGTGGGATGGCTGCTGTTAGCGTTACTTCGAGGGATTTCTTTTTTGGTTTAGAT
CTCACACTGCTAAAGAGATCAAGTGGGGCGGTGCAAAACATGATCCTGATGACAAAG
ATGACTTAAGAAAGATGCATCCGAGGTCATCCTTCAAGGCCTTTCTAGAGGTGGTAAA
AATAAATTGACTTATTATACTCCTGACTATGAAACCAAAGATACTGATATCTTGGCAG
CATTCCGAGTAACTCCTCAACCTGGAGTTCCGCCTGAGGAAGCAGGCGCTGCGGTAGC
TGCTGAATCTTCTACTGGTACATGGACAACTGTGTGGACCGATGGACTTACCAGTCTT
GATCGTTATAAAGGGCGATGCTACCACATCGAGCCCGTTGCTGGAGAAGAAAATCAA
TATATTGCTTATGTAGCTTACCCCTTAGACCTTTTTGAAGAAGGTTCTGTTACTAACAT
GTTTACTTCCATTGTGGGTAATGTATTTGGGTTCAAAGCCCTACGCGCTCTCCGTCTGG
AGGATTTGCGAATCCCTCCTGCTTATACGAAAACTTTCCAAGGCCCGCCTCATGGTAT
CCAAGTTGAGAGAGATAAATTGAACAAGTATGGTCGCCCCCTATTGGGCTGTACTATT
AAACCTAAATTGGGGTTATCTGCTAAGAATTACGGTAGAGCTGTTTATGAATGTCTAC
GCGGTGGACTTGATTTTACCAAAGACGACGAGAACGTGAACTCCCAACCATTTATGCG
TTGGAGAGACCGTTTCTTATTTTGTGCCGAAGCAATTTATAAAGCACAGGCGGAAACA
GGTGAAATCAAAGGGCATTATTTGAATGCTACTGCAGGTACATGCGAAGAAATGATC
AAAAGGGCTGTATTTGCCAGAGAATTGGGAGCTCCTATCGTAATGCATGACTACTTAA
CAGGGGGATTCACCGCAAATACTAGCTTGGCTCATTATTGCCGAGATAATGGTTTACT
TCTGCACATCCATCGCGCAATGCATGCAGTTATTGATAGACAGAAGAATCATGGTATG
CACTTTCGTGTACTAGCTAAAGCGTTACGCATGTCTGGTGGAGATCATATTCACGCGG
GTACCGTAGTAGGGAAACTTGAGGGGGAAAGAGAGATCACTTTAGGCTTTGTTGATTT
ACTGCGTGATGATTTTGTTGAAAAAGATAGAAGCCGCGGGATTTATTTCACTCAAGAC
TGGGTCTCCCTACCAGGTGTTATACCCGTGGCTTCGGGAGGTATTCACGTTTGGCATA
TGCCTGCTCTGACCGAGATCTTTGGAGATGATTCCGTACTACAATTCGGTGGGGGAAC
TTTGGGGCACCCTTGGGGAAATGCACCAGGTGCCGTAGCTAATCGAGTAGCTCTAGA
AGCATGTGTACAAGCTCGTAATGAGGGACGTGATCTTGCTCGTGAGGGTAATGAAATT
ATCCGTGAGGCTAGCAAATGGAGTCCTGAACTAGCTGCTGCTTGTGAAGTGTGGAAA
GAGATTAA-
Sphedamnocarpus_poissonii --

-
ATTTAAATTATGTGTCTGATTCACTAATACCTTACCCCATCCATCTAGAAAAATTGGTT
CAAATCCTTCGTTACTGGATGCAAGATCCCTCTTCTTTGCATTTATTACGACTCTTTCTT
CACGAGTATTGGAATTGGAATAGTTTTATTATTTCAACGAAATCCTTTTCCATTTTTTT
TAATTTAAAAAGGAATCTAAGATTATTGTTGTTCCTATATAATTCTCATGTATATGAAT
ACGAATCCATCTTCTTTTTTCTCCGTAACCAATCCTTTTATTTCCAATCAACATTTTCGG
GGGTGCTTCTTGAACGAATATATTTCTATGGAAAAATACAAGATTTTGTAGAATTTTTT
ACTAATG------
ATTTTAAGGCTAACCTACGGTTGTTCAAGGATCCTTTCATGCATTATGTTAGATATCAA
GGAAAAGGCATTCTGGCTTCAAAAAGTACACTTCTTCTGATGAAAAAATGGAAATATT
TCTTTGTCAATTTATGTCAATTTCATTTTTATGTGTGGGTTCATCCAGAACCGATCTAT
ATGAAGTCATTATCCAACCATTCTCTTGACTTTTTGGGTTATCTTTCAAGTATACGAAA
AAATCTTTCAGTGGTACGGAGTCAAATGCTAGAAAATTCATTTCTAATAGATAATATT
ATGAAGAAACTCGATTCAAGAGTTCCAATTATTCCTTTGATTGGATCATTGTCAAAAA
CAAAATTTTGTAACGCAGTAGGGCATCCCATTAGTAAACCGAGCTGGGCGGATTCACC
CGATTCTGATATTATCGACCAATTTTTGCGTATATCCAGAAATCTTTCTCATTACTATA
GCGGGTCCTCAAAAAAAAAAGAGTTTGTCTCGAGTAAAACATATACTTCGACTTTCTTG
TGTTAAAACTTTGGCTCGTAAACACAAAAGTACCGTCCGGGCTTTTTTGAAAAAACTA
GGTCCAGAATTATTGGAAGAATTTTTTACGGAGGAAAAATCGATTCTTGATTTGGTCT
TCCCCACGGCTTATTCTATTTCGCGA?GGTTATACAGAGGGCGGGTTTGGTATTCGGAT
ATTATTTGTATCAATGATCTAGCCAATCATGAATAAGATTAACTGCATTTTATATGTTT
CGGGTTTATTTACTTACTTTTGAAGGACATTTAAATCTTTATTTTCAAAATTACAGTGG
CAAAAAAAACAGTGCATTCTATTCAATATCTCTATGGGGTAAAGAAGGATCAAAAAT
ACTAAAAAAAAAAAATTCCTTTATTA---------------
CCTTTATTAACAATCACTAATAATGAAAAGACTTCTTTTTTTTTGAACAAAATATACCA
AATTGATGGTAATGTAAGAAATATGACGCGTCCCTCTATTACTAT------
TAATCATTTTTTCACGAAAAATATTTTTCCCTATCCCCCTGAAGCGGATAATACTATGT
TATTTCCTATGCTTGTGTTGGTACTATTTACTTTGTTTATTGGAGCTATAGGAATTCCTT
TCAATCAAGAGGGAACTAATTTGGATATATTATCCAAACTGTTAACTCCGTCTTTAAA
TCTTTTGCATCAAAATGAAAATAATTCTTTTGATTGGTATGAATTTATAACAAATGCTA
TTTTTTCAGTCATTATAACTTTTTTTGGAATATTTATAGCGTCTTCCTTCTATAAACCTC
TTTATTCATCGCTACAAAATTTGAACTTACTTAACTCATTTGCTA------AAAAA---------
AGGGGTCGTTGGGACAAAATAATAGGTGCGATATATGATTGGTCCTATAATCGTGGTT
ATATCGATGTTTTTTATGCAATATTGGCTTCAAGGTTTCTCTTCATGAAAAACAAGGTA
CGCATGATATGCGATTGTCTGGCCCCTCCAGTCAAGGTAATCCAAGACGAGAGATTGC
CTCAGCCATTAAGTCTTTGTGGGTCTACATTAAGATCTCCTCATGGCTGCCATTCTCAG
TATATGACAAATATGGGGACAATTGCATCGCTTGTGATGTCTGTGACTATCAACGAAG
ATGATGATACGATGGATGGTGATCAGCAGCAGATGGCAAGAAAATTGTGGGGCTTAG
TTGTTTGTCATCATACTAGTCCTCGGTTTGTTCCGTTTCCTTTGAGGTATGCTTGTGAAT
TTTTGATTCAAGTGTTTGGTGTTCAGATCAACAAGGAGGTAGAATTAGCTGCTCAGGT
GAGGGAAAAACATATTTTACAAATTCAGACTATGCTATGCGACATGCTCCTTAGAGAT
GCTCCAGTTGCAATTATCACACAATCTCCGAATGTGATGGATCTTGTTAAGTCTGATG
GAACTGCCTTATACTTCAAGAACAAAACTTGGTTGCTCGGGGTAACCCCTACAGAGGA
ACAAATCAGAGACATAGCAGAATGGCTGCTCCAATATCATAGCGGAAACACCGGTTT
AAGTACTGACAGCCTTATGGAAGCTGGATATCCAGGTGCTTCAGCTCTTGGTGATTCA
GTTTGTGGGATGGCTGCTGTTAGCATTACTTCGAGGGATTTTCTTTTTTGGTTTAGATC
TCACACTGCTAAAGAGATCAAGTGGGGCGGTGCAAAACATGATCCTGATGACAAAGA
TGACTTAAGAAAGATGCATCCGAGGTCATCCTTCAAGGCCTTTCTAGAGGTGGTAAAA
ATAAATTGACTTATTATACTCCTGACTATGAAACCAAAGATACTGATATCTTGGCAGC
ATTCCGAGTAACTCCTCAACCTGGAGTTCCGCCTGAGGAAGCAGGCGCTGCGGTAGCT
GCTGAATCTTCTACTGGTACATGGACAACTGTGTGGACCGATGGACTTACCAGTCTTG
ATCGTTATAAAGGGCGATGCTACCACATCGAGCCCGTTGCTGGAGAAGAAAATCAAT
ATATTGCTTATGTAGCTTACCCCTTAGACCTTTTTGAAGAAGGTTCTGTTACTAACATG
TTTACTTCCATTGTGGGTAATGTATTTGGGTTCAAAGCCCTACGCGCTCTCCGTCTGGA
GGATTTGCGAATCCCTCCTGCTTATACGAAAACTTTCCAAGGCCCGCCTCATGGTATC
CAAGTTGAGAGAGATAAATTGAACAAATATGGTCGCCCCCTATTGGGCTGTACTATTA
AACCTAAATTGGGGTTATCTGCTAAGAATTACGGTAGAGCTGTTTATGAATGTCTACG
CGGTGGACTTGATTTTACCAAAGACGACGAGAACGTGAACTCCCAACCATTTATGCGT

TGGAGAGACCGTTTCTTATTTTGTGCCGAAGCACTTTATAAAGCACAGGCGGAAACAG
GTGAAATCAAAGGGCATTATTTGAATGCTACTGCAGGTACATGCGAAGAAATGATCA
AAAGGGCTGTATTTGCCAGAGAATTGGGAGCTCCTATCGTAATGCATGACTACTTAAC
AGGGGGATTCACCGCAAATACTAGCTTGGCTCATTATTGCCGAGATAATGGTTTACTT
CTGCACATCCATCGCGCAATGCATGCAGTTATTGATAGACAGAAGAATCATGGTATGC
ACTTTCGTGTACTAGCTAAAGCGTTACGCATGTCTGGTGGAGATCATATTCACGCGGG
TACCGTAGTAGGGAAACTTGAGGGGGAAAGAGACATCACTTTAGGCTTTGTTGATTTA
CTGCGTGATGATTTTGTTGAAAAAGATAGAAGCCGCGGGATTTATTTCACTCAAGACT
GGGTCTCCCTACCAGGTGTTATACCCGTGGCTTCGGGAGGTATTCACGTTTGGCATAT
GCCTGCTCTGACCGAGATCTTTGGAGATGATTCCGTACTACAATTCGGTGGGGGAACT
TTGGGGCACCCTTGGGGAAATGCACCAGGTGCCGTAGCTAATCGAGTAGCTCTAGAA
GCATGTGTACAAGCTCGTAATGAGGGACGTGATCTTGCTCGTGAGGGTAATGAAATTA
TCCGTGAGGCTAGCAAATGGAGTCCTGAACTAGCTGCTGCTTGTGAAGTGTGGAAAG
AGATTAA-
Stigmaphyllon_aberrans --

ATTTAAATTATGTGTCTGATCCACTAATACCTTACCCCATCCATCTAGAAAAATTGGTT
CAAATCCTTCGTTACTGGATGCAAGATCCCTCTTCTTTGCATTTATTACGACTCTTTCTT
CACGAGTATTGGAATTGGAATAGTTTTCTTATTTCAACGAAATCCTTTTCCATTTTTTT
TAATTTAAAAAGGAATCTAAGATTATTGTTGTTCCTATATAATTCTCATGTATATGAAT
ACGAATCCATCTTCTTTTTTCTCCGAAACCAATCCTTTTATTTCCGATCAACATTTTCG
GGGGTGCTTCTTGAACGAATATATTTCTATGGAAAAATACAAGATTTTGTAGAACTTT
TTACTAATG------
ATTTTAAGGCTAACCTACGGTTGTTCAAGGATCCTTTCATGCATTATGTTAGATATCAA
GGAAAAGGCATTCTGGCTTCAAAAAGTACACTTTTTCCGATGAAAAAATGGAAATATT
TCTTTGTCAATTTATGTCAATTTCATTTTTATGTGTGGGTTCATCCAGAACCGATCTAT
ATGAAGTCATTATCCAACAATTCTCTTGACTTTTTGGGTTATCTTTCAAGTATACGAAA
AAATCTTTCAGTGGTACGGAGTCAAATGCTAGAAAATTCATTTCTAATAGATAATATT
ATGAAGAAATTTGATTCAAAAGTTCCAATTATTCCTTTGATTGGGTCATTGTCAAAAA
CAAAATTTTGTAACGCAGTAGGGCATCCCATTAGTAAACCGAGCTGGGCGGATTCACC
CGATTCTGATATTATCGACCAATTTTTGCGTATATCCAGAAATCTTTCTCATTACTATA
GTGGATCCTCAAAAAAAAAGAGTTTGTATCGAGTAAAATATATACTTCGACTTTCTTG
TGTTAAAACTTTGGCTCGTAAACACAAAAGTACCGCCCGGGCTTTTTTGAAAAAACTA
GGTTCAGAATTATTGGAAGAATTTTTT?CGGAGGAAAAATCGATTCTTGATTTGGTCTT
CCCCCCGGCTTATTCTATTTCGCGAAGGTTATACAGAGGG?GCATTTGGTATTTGGATA
TTATTTGTATCAATGATCTAGCCAATCATGAATAAGATTAACTGCATTTTATATGTTTC
GGGTTTATTTACTTACTTTTGAAGGACATTTTAATCTTTATTTTCAAAATTACAGTGGC
AAAAAAAACAGTTCATTCTATTCAATATCTCTATGGGGTAAAGAAGAATCAAAAATA
CTAAAAAAAAAAAATTCCTTTATTA---------------
CCTTTATTAACAATCACTAATAATGAAAAGACTTCTTTTTTTTTGAACAAAATATATCA
AATTGACGGTAATGTAAGAAATATCACGCGGCCCTCTATTACTAT------
TAATCATTTTTTCACGAAAAATATTTTTCCCTATCCCCCTGAAGCGGATAATACTATGT
TATTTCCTATGCTTGTGTTGGTACTATTTACTTTGTTTATTGGAGCTATAGGAGTTCCTT
TCAATCAAGAGGGAACTAATTTGGATATATTATCCAAACTGTTAACTCCGTATTTAAA
TCTTTTGCATCAAAATGAAAATAATTCTTTTGATTGGTATGAATTTATAACAAATGCTA
TTTTTTCAGTCATTATAACTTTTTTTGGAATATTTATAGCGTCTTCCTTCTATAAACCTC
TTTATTCATCGCTACAAAATTTGAACTTACTTAACTCATTTG-TA------
AAAAAGGTCCTAAGAGGGGTCGTTGGGACAAAATAATAGGTGCGATATATGATTGGT
CCTATAATCGTGGTTATATCGATGCTTTTTATGCAATATTGGCTTCAAGGTTTCTCTTC
ATGAAAAACAAGGTACGCATGATATGCGATTGTCTGGCCCCTCCAGTCAAAGTAATCC
AAGACGAGAGATTGCCTCTGCCATTAAGTCTTTGTGGGTCTACATTAAGATCTCCTCA
TGGTTGCCATGCTCAGTATATGACAAATATGGGGACAATTGCATCTCTTGTGATGTCT

GTAACTATCAACGAAGATGATGATACAATGGATGGTGATCAGCAGCAGATGACAAGA
AAATTGTGGGGCTTAGTTGTATGTCATCATACTAGTCCTCGATTTGTGCCGTTTCCTTT
GAGGTATGCTTGTGAATTTTTGATTCAAGTGTTTGGTGTTCAGATCAACAAGGAGGTA
GAGTTAGCTGCTCAGGTGAGGGAAAAACATATTTTACAAATTCAGACAATGCTATGC
GACATGCTCCTTAGAGATGCTCCTGTTGCAATTATCACACAATCTCCGAACGTGGTGG
ATCTTGTTAAGTGTGATGGAGCTGCCTTATACTTCAAGAACAAAACTTGGTTGCTGGG
AGTAACCCCTACAGAGGAACAAATCAGAGACATAGCTCAATGGCTGCTCGAGTATCA
TAGTGGAAACACGGGTTTAAGTACTGACAGCCTTATGGAAGCTGGATATCCAGGTGCT
TCAGCTCTTGGTGATGCAGTTTGTGGGATGGCTGCTGTTAGCATTACTTCGAGAGATTT
TCTTTTTTGGTTTAGATCTCACACTGCTAAAGAGGTCAAGTGGGGAGGTGCAAAACAC
GATCCTGATGACAAAGATGACTTAAGAAAGATGCATCCGAGGTCATCCTTCAAGGCC
TTTCTAGAGGTGGTAAAAATAAATTGACTTATTATACTCCTGACTATGAAACCAAAGA
TACTGATATCTTGGCAGCATTCCGAGTAACTCCTCAACCTGGAGTTCCGCCTGAGGAA
GCAGGCGCTGCGGTAGCTGCTGAATCTTCTACTGGTACATGGACAACTGTGTGGACCG
ATGGACTTACCAGTCTTGATCGTTATAAAGGGCGATGCTACCACATCGAGCCCGTTGC
TGGAGAAGAAAATCAATATATTGCTTATGTAGCTTACCCCTTGGACCTTTTTGAAGAA
GGTTCTGTTACTAACATGTTTACTTCCATTGTGGGTAATGTATTTGGGTTCAAAGCCCT
ACGCGCTCTCCGTCTGGAGGATTTGCGAATCCCTCCTGCTTATACGAAAACTTTCCAA
GGCCCGCCTCATGGTATCCAAGTTGAGAGAGATAAATTGAACAAGTATGGTCGCCCC
CTATTGGGCTGTACTATTAAACCTAAATTGGGGTTATCCGCTAAGAATTACGGTAGAG
CTGTTTATGAATGTCTACGCGGTGGACTTGATTTTACCAAAGACGACGAGAACGTGAA
CTCCCAACCATTTATGCGTTGGAGAGACCGTTTCTTATTTTGTGCCGAAGCACTTTATA
AAGCACAGGCGGAAACAGGTGAAATCAAAGGGCATTATTTGAATGCTACTGCAGGTA
CATGCGAAGAAATGATCAAAAGGGCTGTATGTGCCAGAGAATTGGGAGTTCCTATCG
TAATGCATGACTACTTAACAGGGGGATTCACCGCAAATACTAGCTTGGCTCATTATTG
CCGAGATAATGGTTTACTTCTGCACATCCATCGCGCAATGCATGCAGTTATTGATAGA
CAGAAGAATCATGGTATGCACTTTCGCGTACTAGCTAAAGCGTTACGTATGTCTGGTG
GAGATCATATTCACGCGGGTACTGTAGTAGGGAAACTTGAGGGGGAAAGAGAGATCA
CTTTAGGCTTTGTTGATTTACTGCGTGATGATTTTGTTGAAAAAGATAGAAGCCGCGG
GATTTATTTCACTCAAGACTGGGTCTCCCTACCAGGTGTTATACCCGTGGCTTCGGGA
GGTATTCACGTTTGGCATATGCCTGCTCTGACCGAGATCTTTGGAGATGATTCCGTACT
ACAATTCGGTGGAGGAACTTTGGGGCACCCTTGGGGAAATGCACCAGGTGCCGTAGC
TAATCGAGTAGCTCTAGAAGCATGTGTAAAAGCTCGTAATGAGGGACGTGATCTTGCT
CGTGAGGGTAATGAAATTATCCGTGAGGCTAGCAAATGGAGTCCTGAACTAGCTGCT
GCTTGTGAAGTGTGGAAAGAGATTAA-
Stigmaphyllon_ciliatum --

ATTTAAATTATGTGTCTGATCCACTAATACCTTACCCCATCCATCTAGAAAAATTGGTT
CAAATCCTTCGTTACTGGATGCAAGATCCCTCTTCTTTGCATTTATTACGACTCTTTCTT
CACGAGTATTGGAATTGGAATAGTTTTATTATTTCAACGAAATCCTTTTCCATTTTTTT
TCATTTAAAAAGGAATCTAAGATTATTGTTGTTCCTATATAATTCTCATGTATATGAAT
ACGAATCCATCTTCTTTTTTCTCCGAAACCAATCCTTTTATTTCCGATCAACATTTTCG
GGGGTGCTTCTTGAACGAATATATTTCTATGGAAAAATACAAGATTTTGTAGAACTTT
TTACTAATG------
ATTTTAAGGCTAACCTACGGTTGTTCAAGGATCCTTTCATGCATTATGTTAGATATCAA
GGAAAAGGCATTCTGGCTTCAAAAAGTACACTTTTTCCGATGAAAAAATGGAAATATT
TCTTTGTCAATTTATGTCAATTTCATTTTTATGTGTGGGTTCATCCAGAACCGATCCAT
ATGAAGTCATTATCCAACCATTCTCTTGACTTTTTGGGGTATCTTTCAAGTATACGAAA
AAATATTTCAGTGGTACGGAGTCAAATGCTAGAAAATTCAGTTCTAATGGATAATATT
ATGAAGAAATTCGATTCAAAAGTTCCAATTATTCCTTTGATTGGGTCATTGTCAAAAA
TAAAATTTTGTAACGCAGTAGGGCATCCCATTAGTAAACCGAGCTGGGCGGATTCACC
CGATTCTGATATTATCGACCAATTTTTGCGTATATCCAGAAATCTTTCTCATTACTATA

GTGGATCCTCAAAAAAAAAGAGTTTGTATCGAGTAAAATATATACTTCGACTTTCTTG
TGTTAAAACTTTGGCTCGTAAACACAAAAGTACCGCCCGGGCTTTTTTGAAAAAACTA
GGTTCAGAATTATTGGAAGAATTTTTTACGGAGGAAAAATCGATTCTTGATTTGGTCT
TCCCCACGGCTTATTCTATTTCGCGAAAGTTATACAGAGGGCGCGTTTGGTATTTGGA
TATTATTTGTATCAATGATCTAGCCAATCATGAATAAGATTAACTGCATTTTATATGTT
TCGGGTTTATTTACTTACTTTTGAAGGACATTTAAATCTTTATTTTCAAAATTACAGTG
GCAAAAAAAACAGTTCATTCTATTCAATATCTCTATGGGGTAAAGAAGGATCAAAAA
TACTAAAAAAAAAAAATTCCTTTATTA---------------
CCTTTATTAACAATCACTAATAATGAAAAGACTTCTTTTTTTTTGAACAAAATATATCA
AATTGACGGTAATCTAAGAAATATGACGCGGCCCTCTATTACTAT------
TAATCATTTTTTCACGAAAAATATTTTTCCCTATCCCCCTGAAGCGGATAATACTATGT
TATTTCCTATGCTTGTGTTGGTACTGTTTACTTTGTTTATTGGAGCTATAGGAGTTCCTT
TCAATCAAGAGGGAACTAATTTGGATATATTATCCAAACTGTTAACTCCGTCTTTAAA
TCTTTTGCATCAAAATGAAAATAATTCTTTTGATTGGTATGAATTTATAACAAATGCTA
TTTTTTCAGTCATTATAACTTTTTTTGGAATATTTATAGCGTCTTCCTTCTATAAACCTC
TTTATTCATCGCTACAAAATTTGAACTTACTTAACTCATTTGTTA------
AAAAAGGTCCTAAGAGAGGTCGTTGGGACAAAATAATAGGTGCGGTATATGATTGGT
CCTATAATCGTGGTTATATCGATGCTTTTTATGCAATATTAGCTTCAAGGTTTCTCTTC
ATGAAAAACAAGGTACGCATGATATGCGATTGTCTGGCCCCTCCAGTCAAAGTAATCC
AAGACGAGAGATTGCCTCAGCCATTAAGTCTTTGTGGGTCTACATTAAGATCTCCTCA
TGGTTGCCATGCTCAGTATATGACAAATATGGGGACAATTGCATCTCTTGTGATGTCT
GTGACTATCAACGAAGATGATGATACGATGGATGGTGATCAGCAGCAGATGACAAGA
AAATTGTGGGGCTTAGTTGTATGTCATCATAATAGTCCTCGATTTGTGCCGTTTCCTTT
GAGGTATGCTTGTGAATTTTTGATTCAAGTGTTTGGTGTTCAGATCAACAAGGAGGTA
GAGTTAGCTGCTCAGGTGAGGGAAAAACATATTTTACAAATTCAGACAATGCTATGC
GACATGCTCCTTAGAGATGCTCCTGTTGCAATTATCACACAATCTCCGAACGTGATGG
ATCTTGTTAAGTCCGATGGAGCTGCCTTATACTTCAAGAACAAAACTTGGTTGCTGGG
AGTAACCCCTACAGAGGAACAAATCAGAGACATAGCTCAATGGCTGCTCGAGTATCA
TAGTGGCAACACGGGTTTAAGTACTGACAGCCTTATGGAAGCTGGATATCCAGGTGCT
TCAGCTCTTGGTGATGCTGTTTGTGGGATGGCTGCTGTTAGCATTACCTCGAGAGATTT
TCTTTTTTGGTTTAGATCTCACACTGCTAAAGAGGTCAAGTGGGGCGGTGCAAAACAT
GATCCTGATGACAAAGATGACCTAAGAAAGATGCATCCGAGGTCATCCTTCAAGGCC
TTTCTAGAGGTGGTAAAAATAAATTGACTTATTATACTCCTGACTATGAAACCAAAGA
TACTGATATCTTGGCAGCATTCCGAGTAACTCCTCAACCTGGAGTTCCGCCTGAGGAA
GCAGGCGCTGCGGTAGCTGCTGAATCTTCTACTGGTACATGGACAACTGTGTGGACCG
ATGGACTTACCAGTCTTGATCGTTATAAAGGGCGATGCTACCACATCGAGCCCGTTGC
TGGAGAAGAAAATCAATATATTGCTTATGTAGCTTACCCCTTAGACCTTTTTGAAGAA
GGTTCTGTTACTAACATGTTTACTTCCATTGTGGGTAATGTATTTGGGTTCAAAGCCCT
ACGCGCTCTCCGTCTGGAGGATTTGCGAATCCCTCCCGCTTATACGAAAACTTTCCAA
GGCCCACCTCATGGTATCCAAGTTGAGAGAGATAAATTGAACAAGTATGGTCGCCCC
CTATTGGGCTGTACTATTAAACCTAAATTGGGGTTATCCGCTAAGAATTACGGTAGAG
CTGTTTATGAATGTCTACGCGGTGGACTCGATTTTACCAAAGACGACGAGAACGTGAA
CTCCCAACCATTTATGCGTTGGAGAGACCGTTTCTTATTTTGTGCCGAAGCACTTTATA
AAGCACAGGCGGAAACAGGTGAAATCAAAGGGCATTATTTGAATGCTACTGCAGGTA
CATGCGAAGAAATGATCAAAAGGGCTGTATGTGCCAGAGAATTGGGAGTTCCTATCG
TAATGCACGACTACTTAACAGGGGGATTCACCGCAAATACTAGCTTGGCTCATTATTG
CCGAGATAATGGTTTACTTCTGCACATCCATCGCGCAATGCATGCAGTTATTGATAGA
CAGAAGAATCATGGTATGCACTTTCGTGTACTAGCTAAAGCGTTACGTATGTCTGGTG
GAGATCATATTCACGCGGGTACTGTAGTAGGGAAACTTGAGGGGGAAAGAGAGATCA
CTTTAGGCTTTGTTGACTTACTGCGTGATGATTTTGTTGAAAAAGATAGAAGCCGCGG
GATTTATTTCACTCAAGACTGGGTCTCCCTACCAGGTGTTATACCCGTGGCTTCGGGA
GGTATTCACGTTTGGCATATGCCTGCTCTGACCGAGATCTTTGGAGATGATTCCGTACT
ACAATTCGGTGGAGGAACTTTGGGGCACCCTTGGGGAAATGCACCTGGTGCCGTAGC
TAATCGAGTAGCTCTAGAAGCGTGTGTAAAAGCTCGTAATGAGGGACGTGATCTTGCT
CGTGAGGGTAATGAAATTATCCGTGAGGCTAGCAAATGGAGTCCTGAACTAGCTGCT
GCTTGTGAAGTGTGGAAAGAGATTAA-

;
end;
