# Supplementary material for: Dated Phylogeny of Banisteriopsis (Malpighiaceae) Suggests an Ancient Colonization of the Cerrado and No Evidence of Human Manipulation in the Origin of B. caapi
Source: Plants (Basel). 2025 Apr 7;14(7):1149. doi: 10.3390/plants14071149 (PMC11990928; doi:10.3390/plants14071149)

**Supplementary information 3**

Maximum likelihood (ML) majority rule consensus trees for each molecular marker.

ETS marker. Bootstrap percentages are shown above branches. Support values below 70 are considered weakly and do not reflect robust phylogenetic relacionships. Bootstrapping ≥70% are considered high.

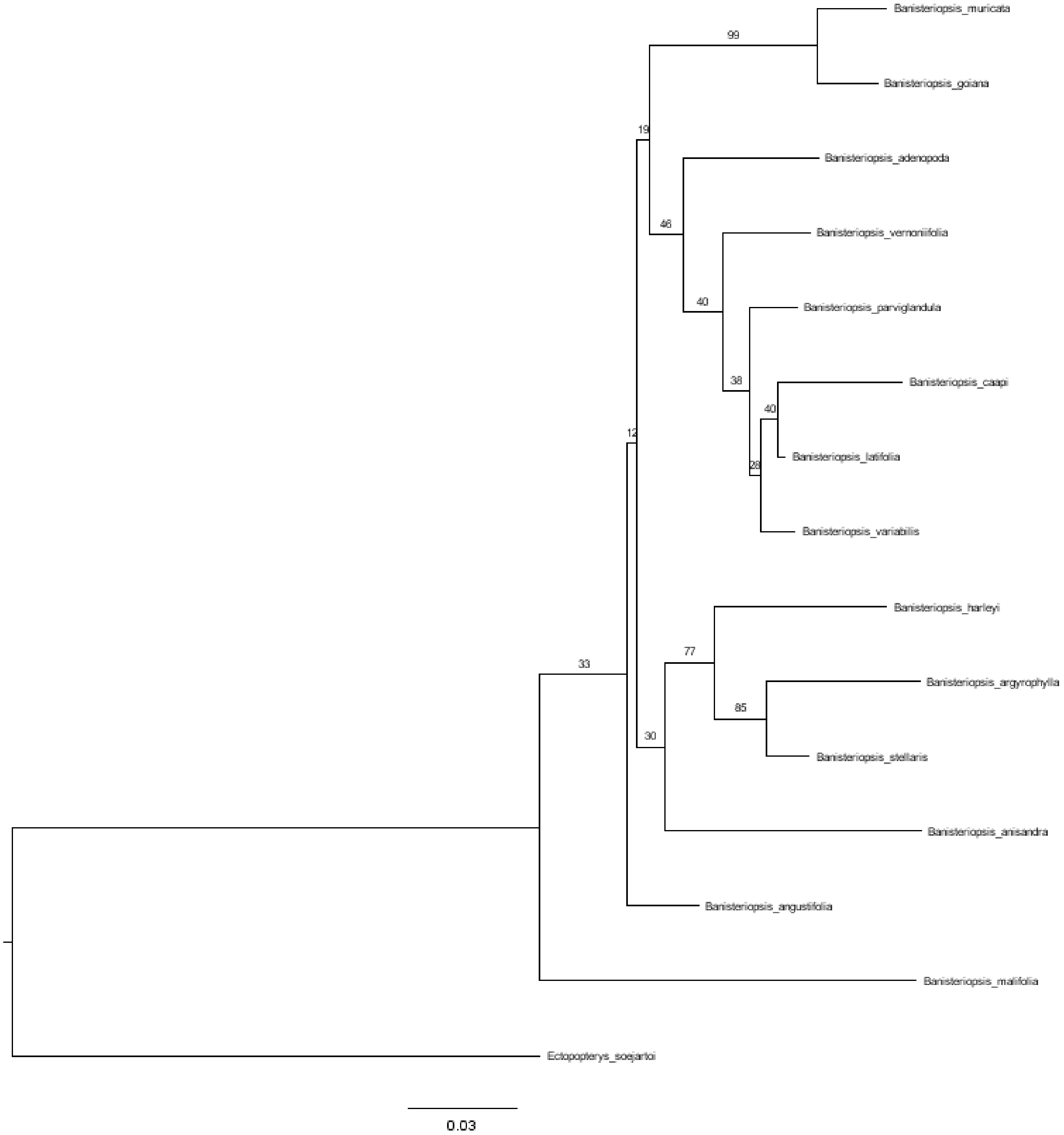

ITS marker. Bootstrap percentages are shown above branches. Support values below 70 are considered weakly and do not reflect robust phylogenetic relacionships. Bootstrapping ≥70% are considered high.

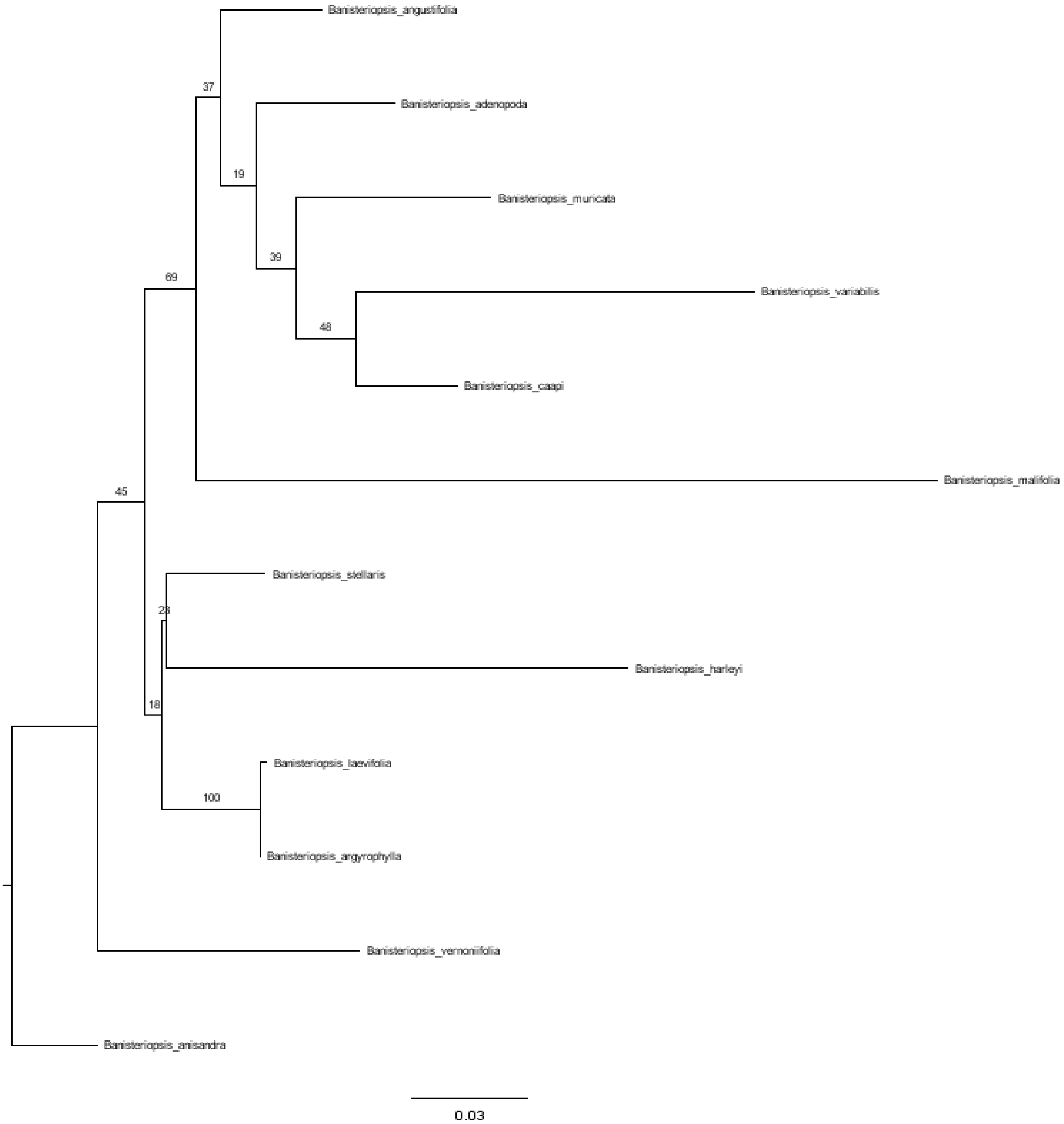

*mat*K marker. Bootstrap percentages are shown above branches. Support values below 70 are considered weakly and do not reflect robust phylogenetic relacionships. Bootstrapping ≥70% are considered high.

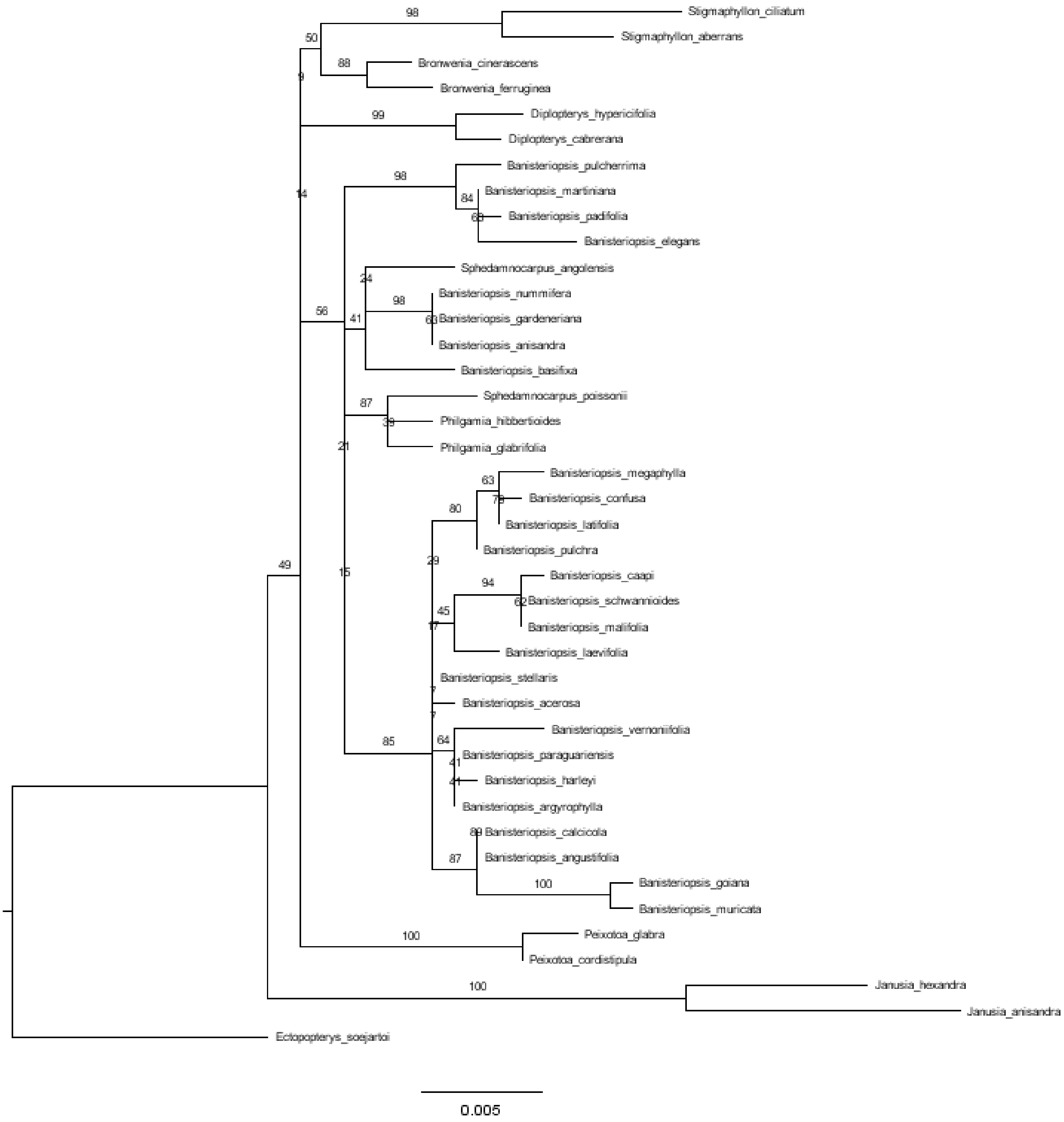

*ndh*F marker. Bootstrap percentages are shown above branches. Support values below 70 are considered weakly and do not reflect robust phylogenetic relacionships. Bootstrapping ≥70% are considered high.

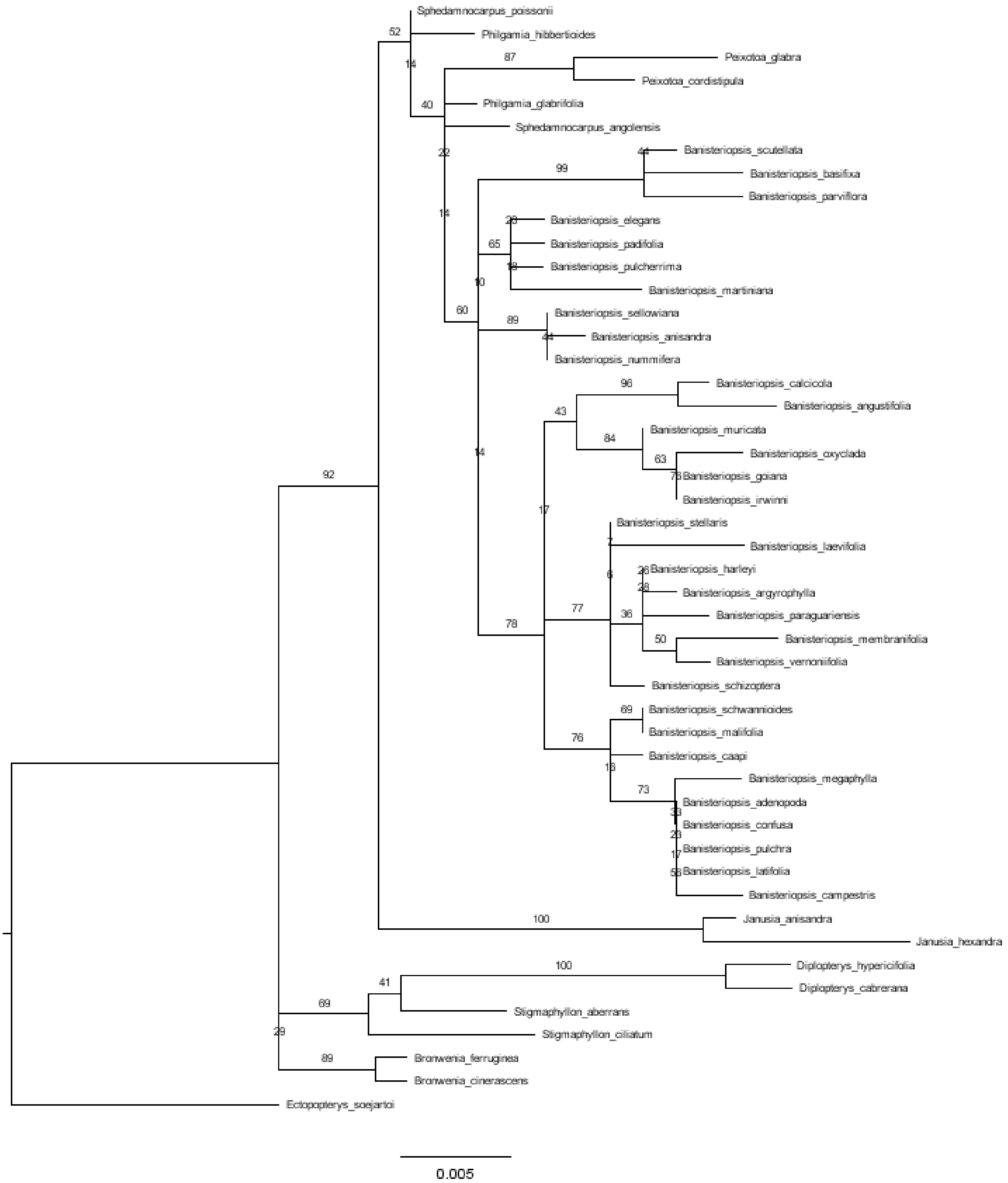

PHYC marker. Bootstrap percentages are shown above branches. Support values below 70 are considered weakly and do not reflect robust phylogenetic relacionships. Bootstrapping ≥70% are considered high.

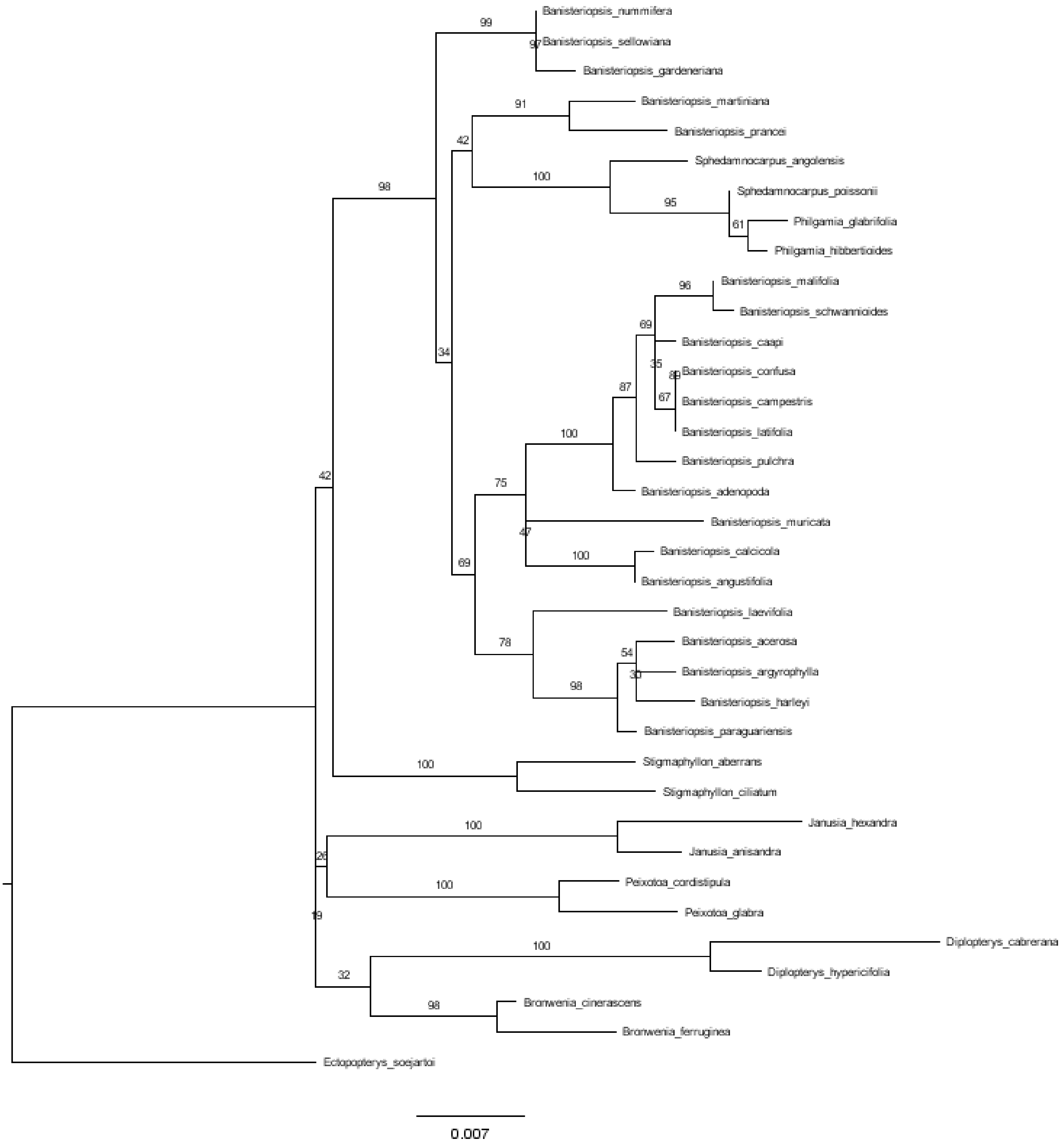

*rbc*L marker. Bootstrap percentages are shown above branches. Support values below 70 are considered weakly and do not reflect robust phylogenetic relacionships. Bootstrapping ≥70% are considered high.

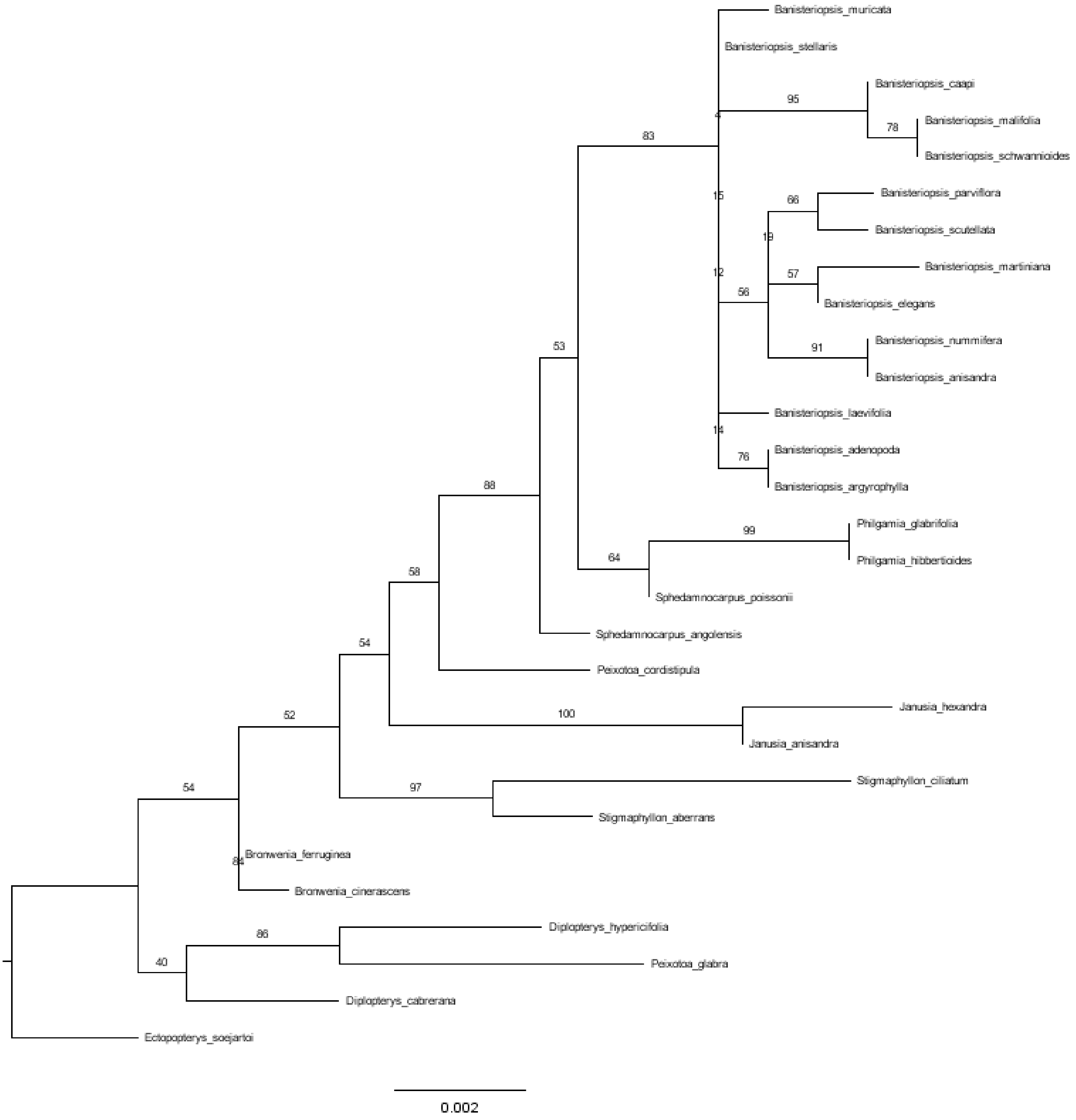

Supplement: Supplementary file 1 [file plants-14-01149-s001.zip › Supplementary information 3.pdf]
